# Primary amine-catalyzed enantioselective 1,4-Michael addition reaction of pyrazolin-5-ones to α,β-unsaturated ketones

**DOI:** 10.3762/bjoc.20.136

**Published:** 2024-07-09

**Authors:** Pooja Goyal, Akhil K Dubey, Raghunath Chowdhury, Amey Wadawale

**Affiliations:** 1 Bio-Organic Division, Bhabha Atomic Research Centre, Trombay, Mumbai 400085, India; 2 Homi Bhabha National Institute, Anushaktinagar, Mumbai 400094, India; 3 Chemistry Division, Bhabha Atomic Research Centre, Trombay, Mumbai 400085, India

**Keywords:** α,β-unsaturated ketones, iminium catalysis, organocatalysis, pyrazoles

## Abstract

The enantioselective 1,4-addition reaction of pyrazolin-5-ones to α,β-unsaturated ketones catalyzed by a cinchona alkaloid-derived primary amine–Brønsted acid composite is reported. Both enantiomers of the anticipated pyrazole derivatives were obtained in good to excellent yields (up to 97%) and high enantioselectivities (up to 98.5% ee) under mild reaction conditions. In addition, this protocol was further expanded to synthesize highly enantioenriched hybrid molecules bearing biologically relevant heterocycles.

## Introduction

**Figure 1:**
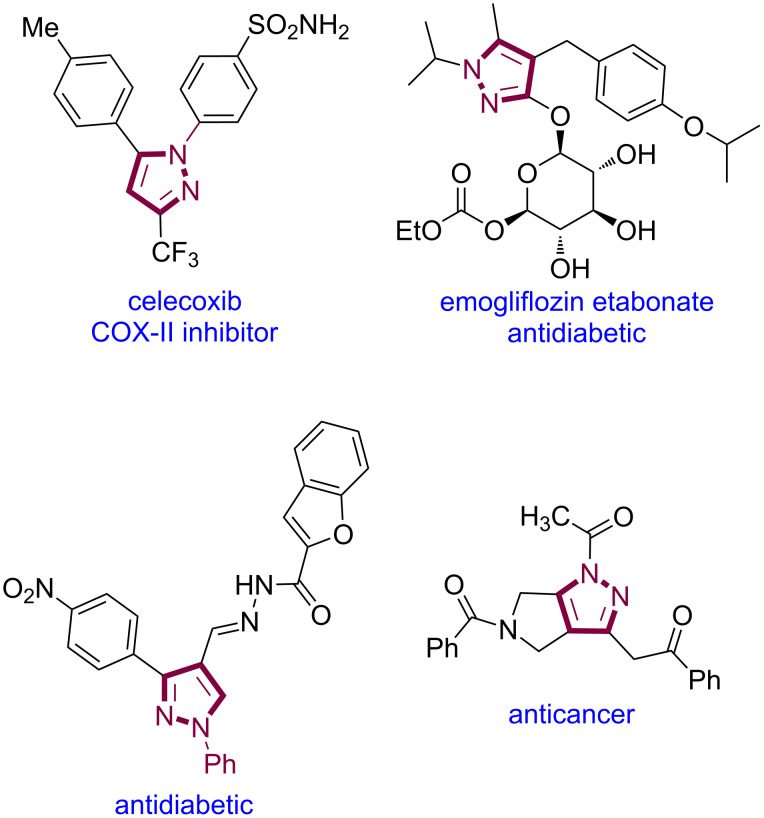
Selected examples of drugs and bioactive molecules bearing a pyrazole core.

*N*-Heterocycles are attractive molecules owing to their extensive applications in small-molecule drugs, natural products, and agrochemical products [1–3]. Among the *N*-heterocycles, pyrazole is an important structural scaffold, found in several marketed drugs and bioactive molecules (Figure 1) [4–7]. In addition, this moiety is an integral part of various agrochemical products and chelating agents [4–9]. Given the importance and widespread applications of pyrazoles, considerable efforts have been devoted to develop new protocols to access structurally diverse pyrazole derivatives [4–7,10–12].

4-Unsubstituted pyrazolin-5-ones are well known precursors for the construction of optically active structurally diverse pyrazoles [10–12]. In this context, the organocatalyzed asymmetric Michael addition of 4-unsubstituted pyrazolin-5-ones to a variety of Michael acceptors has emerged as one of the most powerful strategies to access enantioenriched pyrazole derivatives [10–21]. In the majority of these cases, the reactivities of the pyrazolin-5-one derivatives were harnessed under non-covalent catalysis via bifunctional hydrogen-bonding organocatalysts. The C-4 nucleophilicity of pyrazolin-5-ones was also explored in enantioselective reactions with α,β-unsaturated carbonyl compounds through covalent catalysis with chiral amine-based catalysts; however, it has achieved limited success [10–21].

**Scheme 1:**
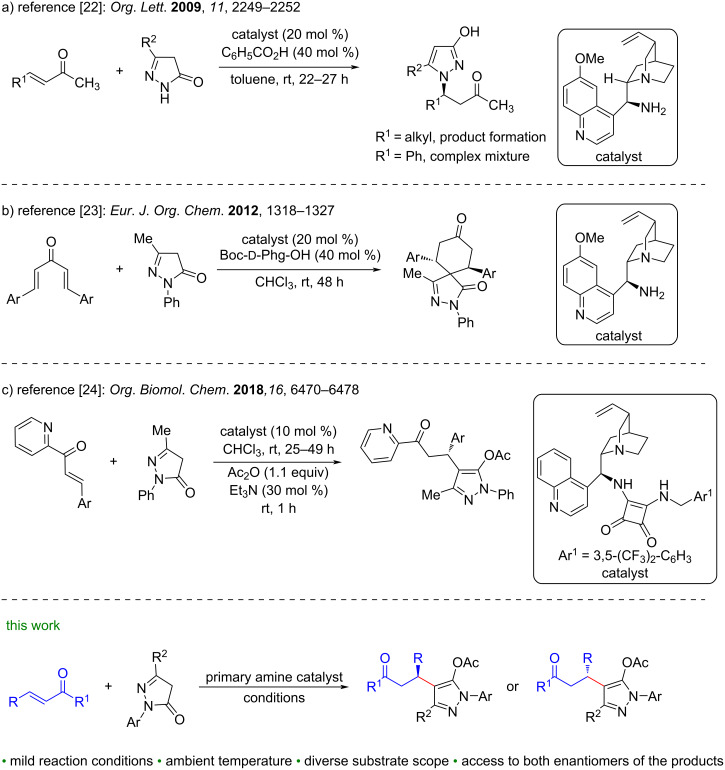
Representative examples of asymmetric organocatalytic conjugate addition of pyrazolin-5-ones to α,β-unsaturated ketones and present study.

Among the developed organocatalyzed enantioselective 1,4-addition reactions of pyrazolin-5-ones, the catalytic asymmetric reactions of pyrazolin-5-ones with α,β-unsaturated ketones are comparatively less studied. In 2009, Zhao’s group were the first who reported a chiral amine-catalysed aza-Michael addition reaction of pyrazolin-5-ones with α,β-unsaturated ketones to access β-(3-hydroxypyrazol-1-yl)ketones (Scheme 1a) [22]. The developed reaction was restricted to α,β-unsaturated ketones with aliphatic substituents (Scheme 1a) [22]. Ji and Wang disclosed organocatalyzed [5 + 1] double Michael additions between pyrazolones and dienones (Scheme 1b) [23]. Very recently, the Chimni group reported a cinchona-derived squaramide-catalyzed 1,4-Michael addition reaction of pyrazolin-5-ones with 2-enoylpyridines (Scheme 1c) [24]. Recently, we developed an organocatalyzed asymmetric Michael addition reaction of 4-monosubstituted pyrazol-5-ones to simple enones for the synthesis of pyrazolone derivatives [25]. Despite these progresses, arylidene/heteroarylideneacetones have remained untapped by 4-unsubstituted pyrazolin-5-ones under asymmetric organocatalytic or metal catalytic conditions. In continuation of our work in the field of organocatalysis [26–29], herein, we present the Michael addition reaction of 4-unsubstituted pyrazolin-5-ones with arylidene/heteroarylideneacetones using cinchona alkaloid-derived primary amine catalysts. The developed protocol delivered both enantiomers of the desired products in good to excellent yields and enantioselectivities.

## Results and Discussion

At the outset, the reaction between commercially available benzylideneacetone (**1a**) and 3-methyl-1-phenyl-2-pyrazolin-5-one (**2a**) was studied in the presence of a panel of primary amine catalysts (see Table S1 in Supporting Information File 1) in toluene at room temperature (30–32 °C). When the test reaction was conducted in the presence of 15 mol % of 9-amino-9-deoxy-epicinchonidine (**I**) as catalyst [30] for 12 h and treated with Ac_2_O followed by DABCO, the reaction gave the conjugate addition product **3aa** in 58–62% yield with 74% ee (Table 1, entry 1). On the other hand, 9-amino-9-deoxyepicinchonine (**II**) [30] furnished the opposite enantiomer *ent*-**3aa** in 62% yield and 66% ee (Table 1, entry 2). Among the screened organocatalysts (see Table S1 in Supporting Information File 1), the catalyst **I** imparted the highest enantioselectivity (74% ee) of the Michael product **3aa** (Table 1, entry 1). Different solvents (see details in Supporting Information File 1) were screened for the test reaction using 15 mol % of catalyst **I**. Among them, CHCl_3_ turned out to be the optimal solvent, as the product **3aa** was isolated in reproducible yield (77%) and enantioselectivity 74% ee (Table 1, entry 3). Next, we explored a variety of achiral and/or chiral Brønsted acids **A1**–**6** as additives in order to increase the yield and the enantioselectivity of the reaction (Table 1, entries 4–9). A marked increase in both the yield and enantioselectivity of the product **3aa** were observed. Among the screened Brønsted acids **A1**–**6**, the combination of 15 mol % of the catalyst **I** and 30 mol % of (±)-mandelic acid (**A5**) was found to be superior in terms of enantioselectivity (92% ee) of the product **3aa** (Table 1, entry 8). When the catalyst loading was lowered (10 mol % of **I**/20 mol % of **A5**), the desired product **3aa** was obtained in 71% yield and 91% ee (Table 1, entry 10). Lowering the temperature (−20 °C) of the reaction improved the enantioselectivity of the product **3aa** slightly but decreased its yield (Table 1, entry 11). Subsequently, the effect of concentration on the reaction outcome was also studied. In dilute conditions, both the yield and enantioselectivity of the product **3aa** were improved to 80% and 94%, respectively, at room temperature (Table 1, entry 12).

**Table 1:** Optimization of reaction conditions.^a^

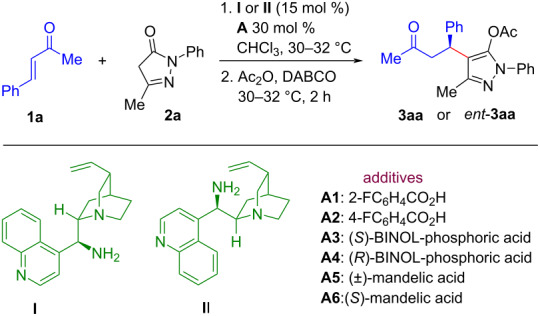				

Entry	Cat.	Additive	Yield (%)^b^	ee (%)^c^

1	**I**	–	58^d^	74
2	**II**	–	62	−66
3	**I**	–	77	74
4	**I**	**A1**	80	90
5	**I**	**A2**	76	88.5
6	**I**	**A3**	86	83
7	**I**	**A4**	83	87
8	**I**	**A5**	77	92
9	**I**	**A6**	77	90
10^e^	**I**	**A5**	71	91
11^f^	**I**	**A5**	58	94
12^g^	**I**	**A5**	80	94
13^g^	**II**	**A5**	76	−87.5

^a^Reaction conditions: **1a** (0.3 mmol), **2a** (0.2 mmol), 15 mol % of catalyst **I** or **II** in 0.5 mL toluene (entries 1 and 2) or catalyst **I** (15 mol %) and 30 mol % **A** in 0.5 mL CHCl_3_ (entries 3–9) for 12–14 h. Next, Ac_2_O (0.52 mmol, 50 µL) was added followed by DABCO (0.1 mmol, 11 mg) and the reaction mixture was further stirred for 2 h at 30–32 °C. ^b^Isolated yield of **3aa** or *ent*-**3aa** after column chromatography. ^c^Enantiomeric excess (ee) was measured by HPLC analysis using a chiralcel OD-H column. ^d^The yield of the reaction product varied from 58–62%. ^e^The reaction was performed in the presence of 10 mol % **I** and 20 mol % **A5**. ^f^The reaction was performed at −20 °C using 15 mol % of catalyst **I** in combination with 30 mol % **A5** in 0.5 mL CHCl_3_ for 24 h. ^g^The reaction was carried out using 15 mol % of catalyst **I** or **II** in combination with 30 mol % **A5** in 1.0 mL CHCl_3_ for 14 h at 30–32 °C. Next, Ac_2_O (0.52 mmol, 50 µL) was added followed by DABCO (0.1 mmol, 11 mg) and the reaction mixture was further stirred for 2 h at 30–32 °C.

Taking into account the results of the optimization studies mentioned above, the catalytic system **I** (15 mol %)/**A5** (30 mol %) in CHCl_3_ (1 mL) at room temperature (30–32 °C) was selected as the optimum reaction conditions (Table 1, entry 12). Under identical optimized reaction conditions, the catalytic system **II** (15 mol %)/**A5** (30 mol %) furnished *ent*-**3aa** in 76% yield and 87.5% ee (Table 1, entry 13).

**Scheme 2:**
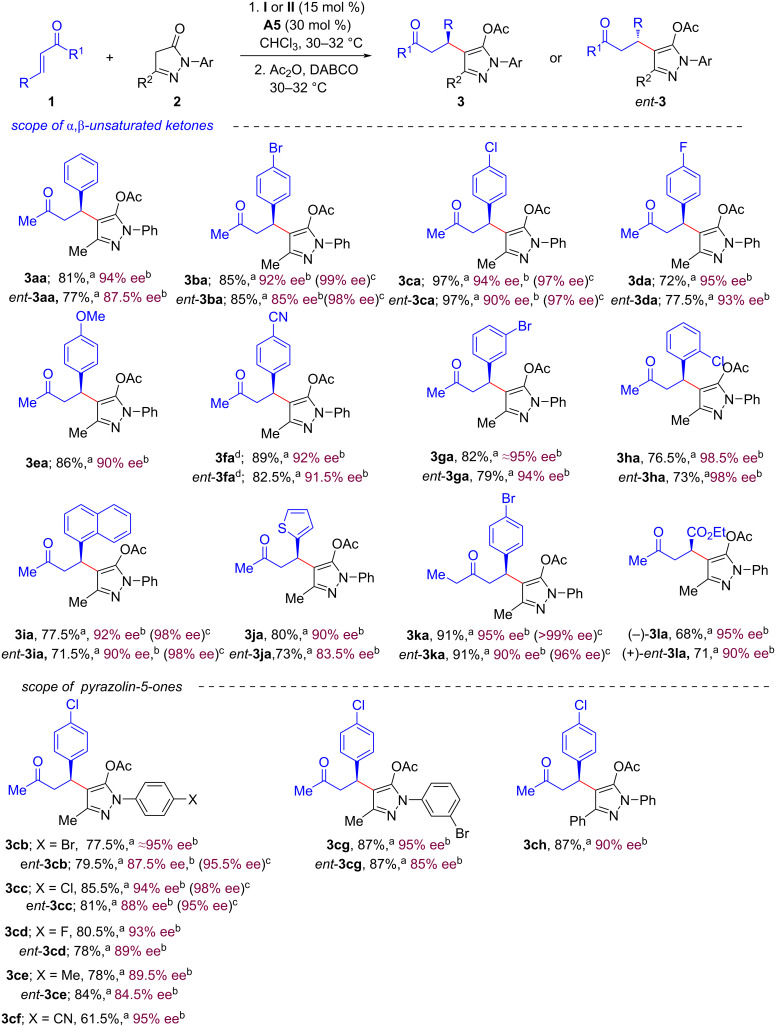
Scope of substrates. Reaction conditions: **1** (0.3 mmol), **2** (0.2 mmol), 15 mol % of catalyst **I**, 30 mol % **A5 (**for **3**) or 15 mol % catalyst **II**, 30 mol % **A5** (for *ent*-**3**) in 1.0 mL CHCl_3_ for 4–14 h. Next, Ac_2_O (0.52 mmol, 50 µL) was added followed by DABCO (0.1 mmol, 11 mg) and the reaction mixture was further stirred for 2 h at 30–32 °C. ^a^Isolated yield of **3** or *ent*-**3** after column chromatography. ^b^Enantiomeric excess (ee) was measured by HPLC analysis using a stationary phase chiral column. ^c^Values in parentheses represent % ee after single recrystallization. ^d^Reaction time for the first step was 4 h.

With the optimal reaction conditions at hands, the 1,4-conjugate addition reaction of a series of α,β-unsaturated ketones **1** with pyrazolin-5-one (**2a**) were studied next (Scheme 2). Aryl α,β-unsaturated ketones bearing a halogen, electron-withdrawing, or electron-donating group at the *para*-position of the benzene ring were compatible and led to the corresponding products **3ba**–**fa** in good to excellent yields (72–97%) and enantioselectivities (90–95% ee). The α,β-unsaturated ketone **1f** with a strong electron-withdrawing group (cyano) in the *para*-position of the benzene ring, was found to be more reactive as the reaction was completed within 4 h and the desired Michael adduct **3fa** was isolated in 89% yield and 92% ee. Notably, the α,β-unsaturated ketone with a substituent in the *meta-*position of the benzene ring was also tolerated and the desired product **3ga** was isolated in good yield (82%) and excellent enantioselectivity (95% ee). To our delight, the α,β-unsaturated ketone with a substituent in the *ortho-*position of the benzene ring, led to the product **3ha** in good yield (76.5%) and highest enantioselectivity (98.5% ee). Moreover, 1-naphthyl-substituted and 2-thienyl-substituted α,β-unsaturated ketones also took part in the reaction and the desired products (**3ia** and **3ja**) were isolated in good yields (77.5% and 80%, respectively) and enantioselectivities (92% ee and 90% ee, respectively). Interestingly, the reaction also worked well with (*E*)-1-phenylpent-1-en-3-one (**1k**) as α,β-unsaturated ketone. The corresponding product, **3ka** was obtained in 91% yield and 95% ee. Moreover, ethyl (*E*)-5-oxohex-2-enoate (**1l**) also showed good reactivity and the expected product (−)-**3la** was isolated in 68% yield and 95% ee.

Next, we explored the scope of pyrazolin-5-ones **2** (Scheme 2, lower part) with diverse substituents (Br, Cl, F, Me or CN) in the *para*-position of the *N*-aryl group. These substrates reacted smoothly with α,β-unsaturated ketone **1c** and the corresponding products **3cb**–**cf** were obtained in good yields (61–85.5%) and good to excellent enantioselectivities (84.5–95% ee). In addition, pyrazolone **2g** with a substituent in the *meta*-position of the *N*-aryl group also participated in the reaction and the desired product **3cg** was isolated in 87% yield and 95% ee. Notably, a phenyl substituent at the C3 position of pyrazolone **2h** was found to be compatible, and the desired product **3ch** was obtained in 87% yield and 90% ee.

In general, enantiomers of a bioactive molecule have different biological activities. Therefore, there is a huge demand to develop methods to access both enantiomers of a chiral compound. We turned our attention to the synthesis of enantiomeric products *ent*-**3**. Under identical optimized reaction conditions (Table 1, entry 12), a panel of aryl/heteroaryl α,β-unsaturated ketones **1** and pyrazolin-5-ones **2** were studied (Scheme 2) using the catalytic system **II** (15 mol %)/**A5** (30 mol %). To our delight, the enantiomeric products *ent*-**3aa–***ent*-**3cg** (Scheme 2) were obtained in good to excellent yields (71–97%) and enantioselectivities (83.5–98% ee).

Molecules containing two or more biologically relevant heterocycle motifs are receiving attention in drug discovery research [31–33]. The enantioselective synthesis of such hybrid molecules is fascinating but at the same time challenging. Pyrazoles [4–7], benzofurans [34], and indoles [35–36] are popular scaffolds as they are prevalent in many bioactive molecules. Compounds bearing both pyrazole and indole moieties or pyrazole and benzofuran moieties (Figure 1) are highly attractive since such compounds might be endowed with potent biological activities.

**Scheme 3:**
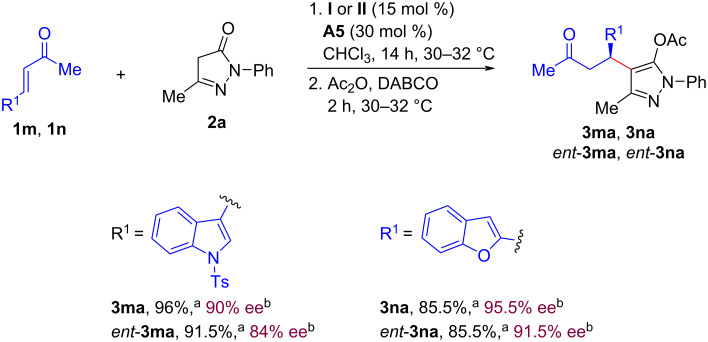
Synthesis of pyrazole-benzofuran and pyrazole–indole hybrid molecules. Reaction conditions: **1m** or **1n** (0.3 mmol), **2a** (0.2 mmol), 15 mol % of catalyst **I**, 30 mol % **A5** (for **3**) or 15 mol % catalyst **II**, 30 mol % **A5** (for *ent*-**3**) in 1.0 mL CHCl_3_ for 14 h. Next, Ac_2_O (0.52 mmol, 50 µL) was added followed by DABCO (0.1 mmol, 11 mg) and the reaction mixture was further stirred for 2 h at 30–32°C. ^a^Isolated yield of **3** or *ent*-**3** after column chromatography. ^b^Enantiomeric excess (ee) was measured by HPLC analysis using a stationary phase chiral column.

Under the disclosed optimized reaction conditions, the reaction between pyrazolin-5-one (**2a**) and indole-derived α,β-unsaturated ketone **1m** was performed. The resulting hybrid molecule **3ma** was isolated in 96% yield and 90% ee (Scheme 3). On the other hand, the reaction of pyrazolin-5-one (**2a**) with benzofuran-derived α,β-unsaturated ketone **1n** delivered the product **3na** in 85.5% yield and 95.5% ee (Scheme 3). Moreover, by employing the catalytic composite **II** (15 mol %) and **A5** (30 mol %) under otherwise identical optimized reaction conditions, the corresponding enantiomeric products (*ent*-**3ma** and *ent*-**3na**) were obtained (Scheme 3) in good yields (91.5% and 85.5%, respectively) and enantioselectivities (84% ee and 91.5% ee, respectively).

**Scheme 4:**
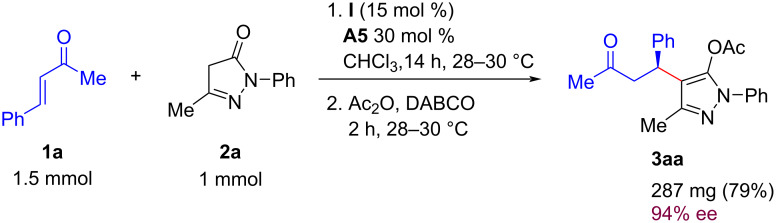
Synthesis of **3aa** on preparative scale.

The practical utility of the developed method was demonstrated by carrying out the synthesis of **3aa** on a 1 mmol scale under the optimized reaction conditions (Scheme 4). The product **3aa** was isolated in slightly lower yield and similar enantioselectivity compared to the 0.2 mmol scale reaction.

**Figure 2:**
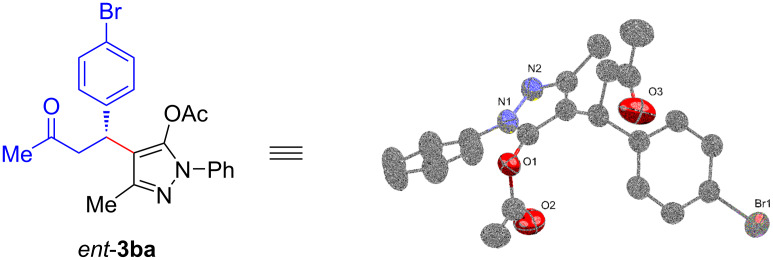
Single crystal X-ray structure of *ent*-**3ba** (CCDC 2234286).

Subsequently, we turned our attention to determine the absolute configuration of the newly formed chiral center. Under the disclosed optimized conditions, the product *ent*-**3ba** was isolated as white solid with 85% ee and the enantiopurity of the product could be enriched to 98% ee by single recrystallization. The absolute stereochemistry was determined to be “*R*” on the basis of single-crystal X-ray crystallography data of *ent*-**3ba** (Figure 2) [37]. The stereochemistry of the products in this series was assigned by analogy.

**Scheme 5:**
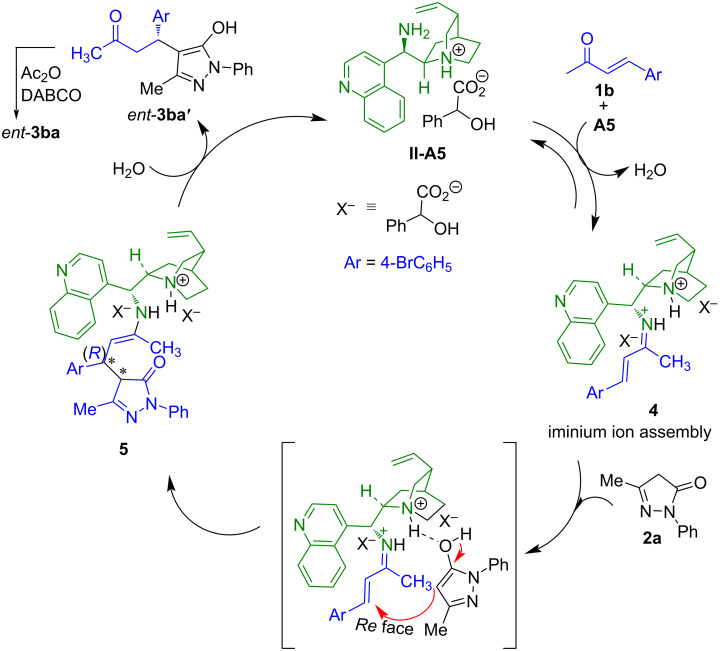
Proposed reaction mechanism.

Based on the observed absolute configuration of product *ent*-**3ba** and preceding literature reports [38,40], a plausible mechanistic pathway is outlined in Scheme 5. Initially, in the presence of one equivalent Brønsted acid additive **A5**, the catalyst **II** generates the monoprotonated diamine **II**-**A5**. The condensation of the primary amine moiety in **II**-**A5** with the carbonyl group of the α,β-unsaturated ketone **1b** in presence of the Brønsted acid leads to the formation of the iminium ion assembly **4** (Scheme 5). It is known that Brønsted acids facilitate the iminium ion formation step [38–39] and the counteranion of the acid plays an important role in the stereocontrolling event [38,40]. On the other hand, the protonated quinuclidine nitrogen atom of the catalyst **II** (in the iminium ion assembly) activates the pyrazol-5-one **2a** through hydrogen bonding and forms the corresponding enol. Simultaneously, the enol form of the pyrazol-5-one attacks the *Re*-face of the α,β-unsaturated ketone **1b** to provide the intermediate **5** (Scheme 5), which after hydrolysis leads to product *ent*-**3ba'**. In situ acetylation of the *ent*-**3ba'** using acetic anhydride and DABCO, furnishes the desired product *ent*-**3ba**.

## Conclusion

In summary, we have realized the Michael addition reaction of 4-unsubstituted pyrazolin-5-ones to α,β-unsaturated ketones under organocatalytic conditions. The developed protocol was efficiently applied to diverse α,β-unsaturated ketones and a pool of pyrazolin-5-ones. The formed Michael adducts were isolated in good to excellent yields and enantioselectivities. The method also led to enantioenriched hybrid molecules bearing pyrazole–indole moieties and pyrazole–benzofuranone moieties. It is worth mentioning that the current protocol delivers both enantiomers of the Michael adducts.

## Experimental

### General procedure for the synthesis of **3** and *ent*-**3**.

In an oven-dried 4 mL glass vial fitted with a magnetic stirring bar, the mixture of catalyst **I** (15 mol %, ≈9.0 mg) and (±)-mandelic acid (30 mol %, 9.0 mg) or catalyst **II** (15 mol %, ≈9.0 mg) and (±)-mandelic acid (30 mol %) in CHCl_3_ (1.0 mL) was stirred at room temperature (30–32 °C) for 5 min. Next, α,β-unsaturated ketone (0.3 mmol, 1.5 equiv) was added in one portion and the reaction mixture was further stirred for 5 min. Then, the pyrazolin-5-one **2** (0.2 mmol, 1.0 equiv) was added to the mixture and stirred for 4–14 h. Once the pyrazolin-5-one **2** was consumed (monitored by TLC), Ac_2_O (50 µL, ≈0.52 mmol, 2.6 equiv) and DABCO (11 mg, 50 mol %) were sequentially added. The resulting reaction mixture was further stirred for 2 h at room temperature. The crude reaction mixture was purified by silica gel (230–400 mesh) column chromatography (petroleum ether/EtOAc as the eluent) to give the product **3** or *ent*-**3**.

## Supporting Information

File 1 Additional optimization studies, characterization data of compounds **3aa**–**na** and *ent*-**3aa-***ent*-**3na**, ^1^H, ^13^C NMR spectra of **3aa**–**na**, ^1^H NMR of *ent*-**3aa–***ent*-**3na** and their HPLC traces and single crystal data of *ent*-**3ba**.

## Data Availability

All data that supports the findings of this study is available in the published article and/or the supporting information to this article.
