# Supplementary material for: Primary amine-catalyzed enantioselective 1,4-Michael addition reaction of pyrazolin-5-ones to α,β-unsaturated ketones
Source: Beilstein J Org Chem. 2024 Jul 9;20:1518–26. doi: 10.3762/bjoc.20.136 (PMC11250233; doi:10.3762/bjoc.20.136)
Supplement: File 1 — Additional optimization studies, characterization data of compounds 3aa–na and ent-3aa-ent-3na, 1H, 13C NMR spectra of 3aa–na, 1H NMR of ent-3aa–ent-3na and their HPLC traces and single crystal data of ent-3ba. [file Beilstein_J_Org_Chem-20-1518-s001.pdf]

BEILSTEIN JOURNAL OF ORGANIC CHEMISTRY

Supporting Information

for

Primary amine-catalyzed enantioselective 1,4-Michael addition reaction of pyrazolin-5-ones to α,β-unsaturated ketones

Pooja Goyal, Akhil K. Dubey, Raghunath Chowdhury and Amey Wadawale

Beilstein J. Org. Chem. **2024,** *20,* 1518–1526. doi:10.3762/bjoc.20.136

Additional optimization studies, characterization data of compounds 3aa–na and *ent*-3aa-*ent*-3na, ^{1}H, ^{13}C NMR spectra of 3aa–na, ^{1}H NMR of *ent*-3aa–*ent*-3na and their HPLC traces and single crystal data of *ent*-3ba

License and Terms: This is a supporting information file under the terms of the Creative Commons Attribution License (https://creativecommons.org/licenses/by/4.0). Please note that the reuse, redistribution and reproduction in particular requires that the author(s) and source are credited and that individual graphics may be subject to special legal provisions.
The license is subject to the *Beilstein Journal of Organic Chemistry* terms and conditions: (https://www.beilstein-journals.org/bjoc/terms)

Table of contents

1	General experimental	S2
2	Reaction optimization	S3
3	General procedure for the synthesis of *racemic* products **3**	S4
4	General procedure for the synthesis of products **3** and *ent*-**3**	S4
5	Characterization data	S5
6	Single crystal X-ray diffraction analysis of *ent*-**3ba**	S30
7	HPLC traces	S35
8	Copies of NMR spectra	S105

1. General experimental

Solvent removal was performed with a rotary evaporator that was connected to a dry ice condenser. TLC (0.5 mm) was carried out using Merck TLC plate. Column chromatography was performed on SRL make silica gel (230–400 mesh). The ^{1}H and ^{13}C NMR spectroscopic data were recorded with a 500 MHz (^{1}H NMR: 500 MHz, ^{13}C NMR: 125 MHz) Varian spectrometer, ^{19}F spectroscopic data were recorded with 300 MHz (^{19}F NMR: 282 MHz). The ^{1}H and ^{13}C chemical shifts are given in ppm (δ scale) and are measured relative to $CHCl_3$ (7.27 ppm) and $CDCl_3$ (77.0 ppm), respectively, as internal standards. High resolution mass spectra were recorded at 60–70 eV with a Micromass Q-TOF spectrometer (ESI, Ar) or on an XEVO G2-XS QTOF. Enantiomeric excess (ee) values were determined by HPLC analysis with a Water instrument fitted with a Daicel Chiralpak AD-H column, Daicel Chiralcel OD-H column and Daicel Chiralpak OJ-H with UV detector (λ fixed at 220 nm). Melting points (mp) were measured on a Büchi B-540 apparatus. The single-crystal X-ray diffraction data of *ent*-**3ba** was collected from were collected using a XtaLAB Synergy, Dualflex, HyPix diffractometer, operating at T = 298(2) K , equipped with a micro-focus sealed X-ray Cu Kα radiation (λ = 1.54184 Å). All the solvents were distilled out prior to use.

2. Reaction optimzations

Table S1: Catalyst and solvent screening[a]

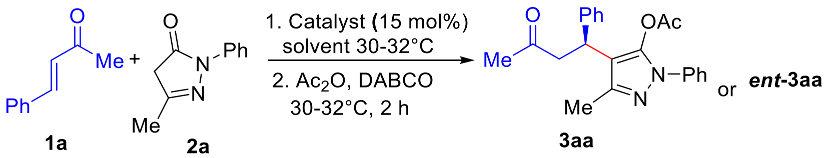

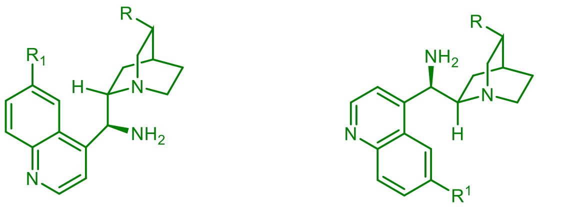

I, R = CH=CH$_2$, R^1 = H,
III, R = CH=CH$_2$, R^1 = OMe,
IV, R = CH$_2$CH$_3$, R^1 = OMe,

II R = CH=CH$_2$, R^1 = H,
V, R = CH=CH$_2$, R^1 = OMe,
VI, R = CH$_2$CH$_3$, R^1 = OMe,

Entry	Cat.	Solvent	Yield[%]of **3aa** [b]	[%]ee[c] of **3aa**
1	**I**	Toluene	58-62	74
2	**II**	Toluene	62	-66
3	**III**	Toluene	75	64
4	**IV**	Toluene	76	58
5	**V**	Toluene	69	-63
6	**VI**	Toluene	66	-59
7	**I**	$C_6H_5CF_3$	ND	69
8	**I**	DCE	70	64
9	**I**	DCM	69	69
10	**I**	CH_3CN	58	42
11	**I**	EtOAc	70	60
12	**I**	$CHCl_3$	77	74

[a] Reaction conditions: **1a** (0.3 mmol), **2a** (0.2 mmol), 15 mol% of catalyst **I-VI** in 0.5 mL solvent for 12h-14h. Next, Ac_2O (0.52 mmol, 50 μL) and DABCO (0.1mmol, 11 mg) were added and stirred for 2h at 30-32°C.

3. General procedure for the synthesis of *rac*-3

An oven-dried 4 mL glass vial equipped with a magnetic stirring bar was charged with catalyst (±)-*trans*-1,2-diaminocyclohexane (20 mol %, ≈ 4.6 mg). Next, $CHCl_3$ (1.0 mL) was added to the mixture and stirred for 5 min at room temperature (30–32 °C). The α,β-unsaturated ketone **1** (0.3 mmol, 1.5 equiv) and pyrazolone **2** were added sequentially, and the reaction mixture was stirred for 12 h. Ac_2O (50 μL, 0.52 mmol, 2.6 equiv) followed by DABCO (12 mg, 50 mol %) were added. The resulting reaction mixture was further stirred for 2 h at room temperature. The crude reaction mixture was directly loaded on the column and purified by column chromatography on silica gel (petroleum ether/EtOAc as the eluent) to give *rac*-**3**.

4. General procedure for the synthesis of 3 and *ent*-3

An oven-dried 4 mL glass vial equipped with a magnetic stirring bar was charged with catalyst **I** (15 mol %, ≈ 9.0 mg) and (±)-mandelic acid (30 mol %, 9.0 mg) or catalyst **II** (15 mol %, ≈ 9.0 mg) and (±)-mandelic acid (30 mol %). Next, $CHCl_3$ (1.0 mL) was added to the mixture and stirred for 5 min at room temperature (30–32 °C). The α,β-unsaturated ketone (0.3 mmol, 1.5 equiv) was added in one portion and the reaction mixture was further stirred for 5 min. Pyrazolin-5-one **2** (0.2 mmol, 1.0 equiv) was added to the reaction mixture and stirred for 4–14 h. Once the pyrazolone **2** was consumed (monitored by TLC), Ac_2O (50 μL, ≈ 0.52 mmol, 2.6 equiv) and DABCO (11 mg, 50 mol %) were sequentially added. The resulting reaction mixture was further stirred for 2 h at room temperature. The crude reaction mixture was directly loaded on the column and purified by column chromatography on silica gel (petroleum ether/EtOAc as the eluent) to give the product **3** or *ent*-**3**.

5. Characterization data

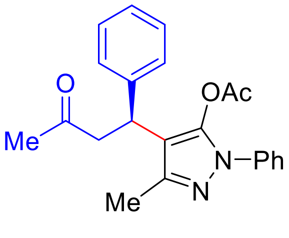

3aa

(*S*)-3-Methyl-4-(3-oxo-1-phenylbutyl)-1-phenyl-1*H*-pyrazol-5-yl acetate (3aa)

Purified by column chromatography on silica gel (230-400 mesh) using eluent 15% EtOAc/ petroleum ether-20% EtOAc/ petroleum ether. The above titled compound was isolated as yellow liquid (59 mg, 81%). **^{1}H NMR (500 MHz, $CDCl_3$)**: δ 7.46-7.44 (m, 2 H), 7.39 (t, *J* = 7.7 Hz, 2 H), 7.28 (t, *J* = 7.2 Hz, 3 H), 7.23-7.22 (m, 2 H), 7.19 (t, *J* = 7.0, 1 H), 4.51 (t, *J* = 7.4 Hz, 1 H), 3.19 (dd, *J* = 17.0, 7.7 Hz, 1 H), 3.06 (dd, *J* = 17.0, 7.0 Hz, 1 H), 2.17 (s, 3 H), 2.14 (s, 3 H), 2.10 (s, 3 H); **^{13}C NMR (125 MHz, $CDCl_3$)**: δ 206.4, 167.4, 147.5, 141.8, 141.3, 137.8, 129.1 (2 C), 128.4 (2 C), 127.4, 127.2 (2 C), 126.4, 122.8 (2 C), 110.8, 47.3, 34.8, 30.4, 20.3, 13.5; **IR** (ATR): 2968, 1788, 1713, 1594, 1501, 1368,1164, 754, 698 cm^{-1}; The ee was determined by HPLC using a Daicel CHIRALCEL® OD-H [hexane/*i*-PrOH (80/20)]; flow rate 1.0 mL/min; λ = 220 nm; τ_{minor} = 7.77 min, τ_{major} = 9.0 min, $[\alpha]_D^{22}$ = -7.5 (*c* 3.0, $CHCl_3$, 94% ee); **HRMS (ESI)** calcd for $C_{22}H_{22}N_2NaO_3$ $[M + Na]^+$: 385.1528, found: 385.1541.

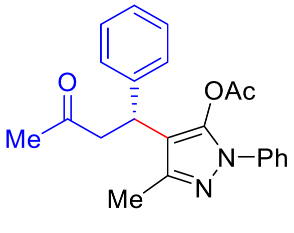

***ent*-3aa**

(*R*)-3-Methyl-4-(3-oxo-1-phenylbutyl)-1-phenyl-1*H*-pyrazol-5-yl acetate (*ent*-3aa)

Purified by column chromatography on silica gel (230-400 mesh) using eluent 15% EtOAc/ petroleum ether-20% EtOAc/petroleum ether. The above titled compound was isolated as yellow

liquid (55 mg, 77%). The ee was determined by HPLC using a Daicel CHIRALCEL® OD-H [hexane/*i*-PrOH (80/20)]; flow rate 1.0 mL/min; λ = 220 nm; τ_{major} = 7.49 min , τ_{minor} = 8.96 min, $[\alpha]_D^{23}$ = +5.0 (*c* 2.1, 87.5% ee $CHCl_3$).

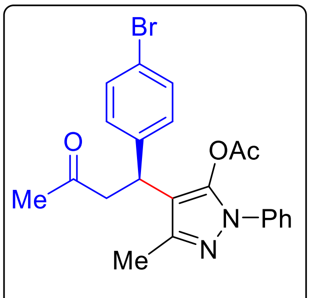

3ba

(*S*)-4-(1-(4-Bromophenyl)-3-oxobutyl)-3-methyl-1-phenyl-1*H*-pyrazol-5-yl acetate (**3ba**)

Purified by column chromatography on silica gel (230-400 mesh) using eluent 15% EtOAc/ petroleum ether-20% EtOAc/petroleum ether. The above titled compound was isolated as colourless liquid (75 mg, ~85%) which solidify on standing in refrigerator. M.p.: 105.5-106.5 °C (n-hexane/ EtOAC : 99/1); **^{1}H NMR (500 MHz, $CDCl_3$)**: *δ* 7.44-7.43 (m, 2 H), 7.40-7.39 (m, 4 H), 7.29 (t, *J* = 7.3 Hz, 1 H), 7.10 (d, *J* = 8.2 Hz, 2 H), 4.45 (t, *J* = 7.3 Hz, 1 H), 3.14 (dd, *J* = 17.2, 7.9 Hz, 1 H), 3.04 (dd, *J* = 17.2, 6.8 Hz, 1 H), 2.14 (s, 6 H), 2.13 (s, 3 H); **^{13}C NMR (125 MHz, $CDCl_3$)**: *δ* 206.0, 167.4, 147.3, 141.2, 141.0, 137.7, 131.4 (2 C), 129.2 (2 C), 129.1 (2 C), 127.3, 122.8 (2 C), 120.2, 110.2, 47.2, 34.2, 30.3, 20.3, 13.5; **IR** (ATR): 2926, 1790, 1719, 1590, 1502, 1370, 1161, 754, 694 cm^{-1}; **HPLC**: 92% ee, the ee was determined by HPLC using a Daicel CHIRALCEL® OD-H [hexane/*i*-PrOH (80/20)]; flow rate 0.5 mL/min; λ = 220 nm; τ_{minor} = 14.87 min, τ_{major} = 17.41 min, $[\alpha]_D^{23}$ = -5.7 (*c* 1.0, $CHCl_3$, >99% ee); **HRMS (ESI)** calcd for $C_{22}H_{22}BrN_2O_3$ $[M + H]^+$: 441.0813, found: 441.0817.

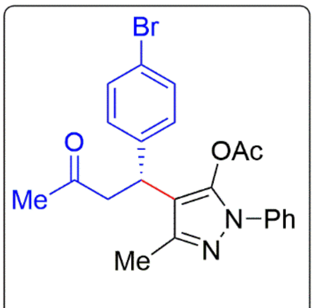

ent-**3ba**

(*R*)-4-(1-(4-Bromophenyl)-3-oxobutyl)-3-methyl-1-phenyl-1*H*-pyrazol-5-yl acetate (*ent*-**3ba**)

Purified by column chromatography on silica gel (230-400 mesh) using eluent 15% EtOAc/ petroleum ether-20% EtOAc/petroleum ether.The above titled compound was isolated as colourless liquid (75 mg, ~85%) which solidify in on standing in refrigerator. Mp. 106-108 °C (n-hexane/ EtOAC : 99/1); The ee was determined by HPLC using a Daicel CHIRALCEL® OD-H [hexane/*i*-PrOH (80/20)]; flow rate 0.5 mL/min; λ = 220 nm; τ_{minor} = 15.12 min, τ_{major} = 18.12 min, $[\alpha]_D^{25}$ = +2.3 (*c* 1.0, $CHCl_3$, 98% ee). **CCDC**: 2234286

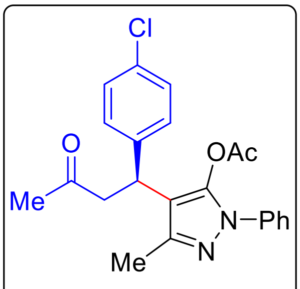

3ca

(*S*)-4-(1-(4-Chlorophenyl)-3-oxobutyl)-3-methyl-1-phenyl-1*H*-pyrazol-5-yl acetate (**3ca**)

Purified by column chromatography on silica gel (230-400 mesh) using 15% EtOAc/petroleum ether-20%EtOAc/petroleum ether. The above titled compound was isolated as colourless liquid (77 mg, 97%) which solidify on standing. M.p.: 101-102 °C (n-hexane/ EtOAC : 99/1); **^{1}H NMR (500 MHz, $CDCl_3$)**: δ 7.45-7.43 (m, 2 H), 7.40 (t, *J* = 7.5 Hz, 2 H), 7.30 (t, *J* = 7.1 Hz, 1 H), 7.25 (d, *J* = 7.1 Hz, 2 H), 7.16 (d, *J* = 8.1 Hz, 2 H), 4.47 (t, *J* = 7.2 Hz, 1 H), 3.15 (dd, *J* = 17.2, 7.9 Hz, 1 H), 3.04 (dd, *J*

= 17.1, 6.8 Hz, 1 H), 2.15 (s, 3 H), 2.14 (s, 3 H), 2.13 (s, 3 H); **^{13}C NMR (125 MHz, CDCl$_3$)**: δ 206.1, 167.5, 147.4, 141.3, 140.5, 137.8, 132.2, 129.1 (2 C), 128.8 (2 C), 128.5 (2 C), 127.3, 122.8 (2 C), 110.4, 47.3, 34.2, 30.4, 20.3, 13.5; The ee was determined by HPLC using a Daicel CHIRALCEL® OD-H [hexane/*i*-PrOH (80/20)]; flow rate 0.5 mL/min; λ = 220 nm; τ_{minor} = 14.31 min, τ_{major} = 17.10 min, $[\alpha]_D^{25}$ = -6.6 (*c* 1.2, CHCl$_3$, 97% ee); **HRMS (ESI)** calcd for $C_{22}H_{22}ClN_2O_3$ $[M + H]^+$: 397.1319, found: 397.1324.

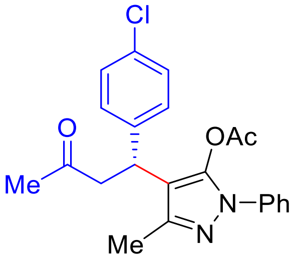

***ent*-3ca**

(*R*)-4-(1-(4-Chlorophenyl)-3-oxobutyl)-3-methyl-1-phenyl-1*H*-pyrazol-5-yl acetate (***ent*-3ca**)

Purified by column chromatography on silica gel (230-400 mesh) using eluent 15%EtOAc/ petroleum ether-20%EtOAc/petroleum ether. The above titled compound was isolated as colourless liquid (77 mg, 97%) which solidify in on standing. M.p.: 99.5-100.5 °C (n-hexane/ IPA: 95/5); The ee was determined by HPLC using a Daicel CHIRALCEL® OD-H [hexane/*i*-PrOH (80/20)]; flow rate 0.5 mL/min; λ = 220 nm; τ_{major} = 14.26 min, τ_{minor} = 17.49 min, $[\alpha]_D^{23}$ = +6.7 (*c* 1.2, CHCl$_3$, 97.5% ee)

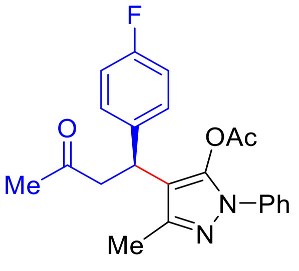

3da

(*S*)-4-(1-(4-Fluorophenyl)-3-oxobutyl)-3-methyl-1-phenyl-1*H*-pyrazol-5-yl acetate (**3da**)

Purified by column chromatography on silica gel (230-400 mesh) using eluent 15% EtOAc/ petroleum ether-20% EtOAc/petroleum ether. The above titled compound was isolated as light-yellow liquid (55 mg, 72%). **^{1}H NMR (500 MHz, CDCl$_3$)**: δ 7.46-7.44 (m, 2 H), 7.40 (t, J = 7.6 Hz, 2 H), 7.29 (t, J = 7.3 Hz, 1 H), 7.21-7.18 (m, 2 H), 6.97 (t, J = 8.6 Hz, 2 H), 4.48 (t, J = 7.4 Hz, 1 H), 3.14 (dd, J = 17.1, 7.9 Hz, 1 H), 3.05 (dd, J = 17.1, 6.9 Hz, 1 H), 2.14 (s, 3 H), 2.139 (s, 3 H), 2.132 (s, 3 H); **^{13}C NMR (125 MHz, CDCl$_3$)**: δ 206.2, 167.6, 161.4 ($J_{C\text{-}F}$ = 243.6 Hz), 147.4, 141.2, 137.8, 137.7, 137.6, 129.2 (2 C), 129.0, 128.9, 127.3, 122.8 (2 C), 115.2 ($J_{C\text{-}F}$ = 21.2 Hz), 110.7, 47.5, 34.1, 30.4, 20.3,13.5; The ee was determined by HPLC using a Daicel CHIRALCEL® OD-H [hexane/*i*-PrOH (80/20)]; flow rate 0.5 mL/min; λ = 220 nm; τ_{minor} = 13.50 min, τ_{major} = 16.34 min, $[\alpha]_D^{21}$ = -8.6 (*c* 2.4, CHCl$_3$, 95%ee); **HRMS (ESI)** calcd for $C_{22}H_{22}FN_2O_3$ [M + H]$^+$: 381.1614, found: 381.1613.

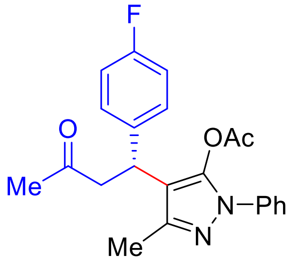

***ent*-3da**

(*R*)-4-(1-(4-Fluorophenyl)-3-oxobutyl)-3-methyl-1-phenyl-1*H*-pyrazol-5-yl acetate (***ent*-3da**)

Purified by column chromatography on silica gel (230-400 mesh) using eluent 15% EtOAc/ petroleum ether-20% EtOAc/petroleum ether. The above titled compound was isolated as light yellow liquid (59 mg, 77.5%). The ee was determined by HPLC using a Daicel CHIRALCEL® OD-H [hexane/*i*-PrOH (80/20)]; flow rate 0.5 mL/min; λ = 220 nm; τ_{major} = 13.55 min, τ_{minor} = 16.73 min, $[\alpha]_D^{22}$ = +6.9 (*c* 1.6, CHCl$_3$, >93% ee).

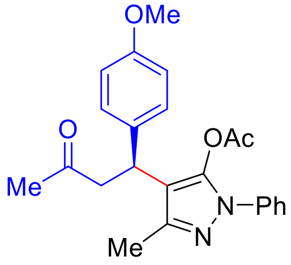

3ea

(*S*)-4-(1-(4-Methoxyphenyl)-3-oxobutyl)-3-methyl-1-phenyl-1*H*-pyrazol-5-yl acetate (**3ea**)

Purified by column chromatography on silica gel (230-400 mesh) using eluent 15% EtOAc/ petroleum ether-25% EtOAc/petroleum ether. The above titled compound was isolated as light yellow liquid (68 mg, 86%).**^{1}H NMR (500 MHz, CDCl$_3$)**: δ 7.46-7.44 (m, 2 H), 7.39 (t, J = 7.9 Hz, 2 H), 7.28 (t, J = 7.1 Hz, 1 H), 7.14 (d, J = 8.4 Hz, 2 H), 6.81 (d, J = 8.6 Hz, 2 H), 4.44 (t, J = 7.4 Hz, 1 H), 3.77 (s, 3 H), 3.14 (dd, J = 16.8, 7.8 Hz, 1 H), 3.02 (dd, J = 16.8, 6.9 Hz, 1 H), 2.15 (s, 3 H), 2.13 (s, 3 H), 2.12 (s, 3 H); **^{13}C NMR (125 MHz, CDCl$_3$)**: δ 206.5, 167.5, 158.0, 147.5, 141.2, 137.9, 133.9, 129.1 (2 C), 128.4 (2 C), 127.2, 122.8 (2 C), 113.8 (2 C), 111.0, 55.2, 47.6, 34.1, 30.4, 20.3, 13.5; The ee was determined by HPLC using a Daicel CHIRALCEL® OD-H [hexane/*i*-PrOH (80/20)]; flow rate 0.5 mL/min; λ = 220 nm; τ_{minor} = 17.64 min, τ_{major} = 19.79 min, $[\alpha]_D^{25}$ = -3.7 (*c* 2.1, CHCl$_3$, 90% ee); **HRMS (ESI)** calcd for $C_{23}H_{25}N_2O_4$ [M + H]$^+$: 393.1814, found: 393.1825.

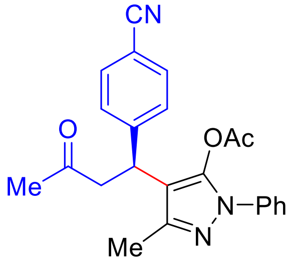

3fa

(*S*)-4-(1-(4-Cyanophenyl)-3-oxobutyl)-3-methyl-1-phenyl-1*H*-pyrazol-5-yl acetate (**3fa**)

Purified by column chromatography on silica gel (230-400 mesh) using eluent 20% EtOAc/ petroleum ether-35% EtOAc/petroleum ether. The above titled compound was isolated as white solid

(69 mg, 89%).^{1}**H NMR (500 MHz, CDCl$_3$)**: δ 7.57 (d, J = 8.2 Hz, 2 H), 7.44-7.38 (m, 4 H), 7.34 (d, J = 8.1 Hz, 2 H), 7.30 (t, J = 7.0 Hz, 1 H), 4.54 (t, J = 7.1 Hz, 1 H), 3.17 (dd, J = 17.6, 7.8 Hz, 1 H), 3.08 (dd, J = 17.6, 6.6 Hz, 1 H), 2.16 (s, 3 H), 2.14 (s, 3 H), 2.10 (s, 3 H); ^{13}**C NMR (125 MHz, CDCl$_3$)**: δ 205.6, 167.4, 147.6, 147.2, 141.3, 137.6, 132.2 (2 C), 129.2 (2 C), 128.3 (2 C), 127.4, 122.8 (2 C), 118.7, 110.3, 109.7, 46.8, 34.6, 30.3, 20.3, 13.5; The ee was determined by HPLC using a Daicel CHIRALCEL® OD-H [hexane/*i*-PrOH (80/20)]; flow rate 0.5 mL/min; λ = 220 nm; τ_{minor} = 27.65 min, τ_{major} = 30.29 min; $[\alpha]_D^{26}$ = -9.9 (*c* 1.8, CHCl$_3$, 92% ee); **HRMS (ESI)** calcd for $C_{23}H_{22}N_3O_3$ $[M + H]^+$: 388.1661, found: 388.1662.

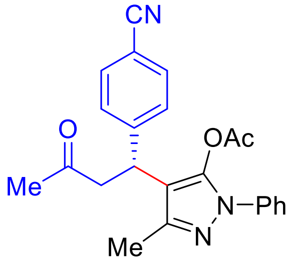

***ent*-3fa**

(*R*)-4-(1-(4-Cyanophenyl)-3-oxobutyl)-3-methyl-1-phenyl-1*H*-pyrazol-5-yl acetate (***ent*-3fa**)

Purified by column chromatography on silica gel (230-400 mesh) using eluent 20% EtOAc/ petroleum ether-35% EtOAc/petroleum ether. The above titled compound was isolated as white solid (64 mg, 82.5%). The ee was determined by HPLC using a Daicel CHIRALCEL® OD-H [hexane/*i*-PrOH (80/20)]; flow rate 0.5 mL/min; λ = 220 nm; τ_{major} = 27.02 min, τ_{minor} = 30.84 min. ; $[\alpha]_D^{26}$ = +10.8 (*c* 3.6, CHCl$_3$, 91.5% ee);

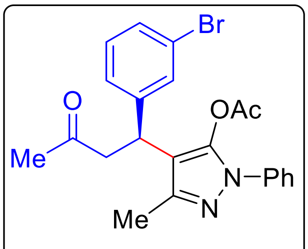

3ga

(*S*)-4-(1-(3-Bromophenyl)-3-oxobutyl)-3-methyl-1-phenyl-1*H*-pyrazol-5-yl acetate (**3ga**)

Purified by column chromatography on silica gel (230-400 mesh) using eluent 15% EtOAc/ petroleum ether-20% EtOAc/petroleum ether. The above titled compound was isolated as yellow liquid (73 mg, 82%). **^{1}H NMR (500 MHz, CDCl$_3$)**: δ 7.44-7.43 (m, 2 H), 7.41-7.37 (m, 3 H), 7.33-7.32 (m, 1 H), 7.29 (t, J = 7.3 Hz, 1 H),7.16-7.14 (m, 2 H), 4.48 (t, J = 7.3 Hz, 1 H), 3.15 (dd, J = 17.3, 7.7 Hz, 1 H), 3.05 (dd J = 17.3, 7.0 Hz, 1 H), 2.18 (s, 3 H), 2.15 (s, 3 H), 2.13 (s, 3 H); **^{13}C NMR (125 MHz, CDCl$_3$)**: δ 205.8, 167.4, 147.3, 144.3, 141.3, 137.8, 130.3, 130.0, 129.6, 129.1 (2 C), 127.3, 126.4, 123.8 (2 C), 122.5, 110.2, 47.1, 34.4, 30.4, 20.3, 13.5; The ee was determined by HPLC using a Daicel CHIRALCEL® OJ-H [hexane/*i*-PrOH (70/30)]; flow rate 1.0 mL/min; λ = 220 nm; τ_{major} = 34.14 min, τ_{minor} = 55.56 min, $[\alpha]_D^{25}$ = -26.3 (*c* 2.0, CHCl$_3$, 94.5% ee); **HRMS (ESI)** calcd for $C_{22}H_{22}BrN_2O_3$ $[M + H]^+$: 441.0814, found: 441.0813.

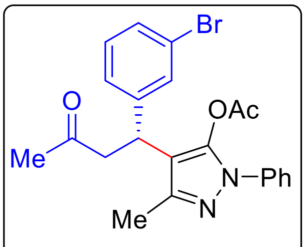

***ent*-3ga**

(*R*)-4-(1-(3-Bromophenyl)-3-oxobutyl)-3-methyl-1-phenyl-1*H*-pyrazol-5-yl acetate (***ent*-3ga**)

Purified by column chromatography on silica gel (230-400 mesh) using eluent 15% EtOAc/ petroleum ether-20% EtOAc/petroleum ether. The above titled compound was isolated as yellow liquid (70 mg, 79%). The ee was determined by HPLC using a Daicel CHIRALCEL® OJ-H

[hexane/*i*-PrOH (70/30)]; flow rate 1.0 mL/min; λ = 220 nm; τ_{minorr} = 41.63 min, τ_{majo} = 51.72 min, $[\alpha]_D^{25}$ = +23.26(*c* 2.8, $CHCl_3$, 94% ee);

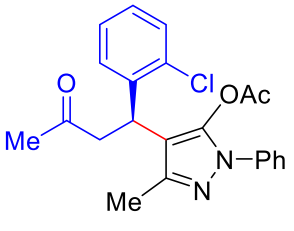

3ha

(*R*)-4-(1-(2-Chlorophenyl)-3-oxobutyl)-3-methyl-1-phenyl-1*H*-pyrazol-5-yl acetate (**3ha**)

Purified by column chromatography on silica gel (230-400 mesh) using eluent 15% EtOAc/ petroleum ether-20%EtOAc/petroleum ether. The above titled compound was isolated as yellow liquid (61 mg, >76.5%). **^{1}H NMR (500 MHz, $CDCl_3$)**: *δ* 7.44-7.42 (m, 2 H), 7.40-7.35 (m, 3 H), 7.29-7.26 (m, 2 H), 7.21 (t, *J* = 7.4 Hz, 1 H), 7.16 (t, *J* = 7.5 Hz, 1 H), 4.86 (t, *J* = 7.6 Hz, 1 H), 3.11 (dd *J* = 17.1, 7.3 Hz, 1 H), 3.06 (dd *J* = 17.4, 8.1 Hz, 1 H), 2.23 (s, 3 H), 2.17 (s, 3 H), 2.06 (s, 3 H); **^{13}C NMR (125 MHz, $CDCl_3$)**: *δ* 205.8, 167.3, 147.8, 141.5, 139.1, 137.8, 133.9, 130.0, 129.1 (2 C), 128.1, 127.9, 127.3, 126.7, 122.9 (2 C), 108.7, 46.8, 32.6, 29.9, 20.3, 13.4; The ee was determined by HPLC using a Daicel CHIRALCEL® OJ-H [hexane/*i*-PrOH (70/30)]; flow rate 1.0 mL/min; λ = 220 nm; τ_{minor} = 34.77 τ_{major} = 50.3 min, $[\alpha]_D^{24}$ = +18.3 (*c* 1.1, $CHCl_3$, >98.5% ee); **HRMS (ESI)** calcd for $C_{22}H_{22}ClN_2O_3$ $[M + H]^+$: 397.1319, found: 397.1331.

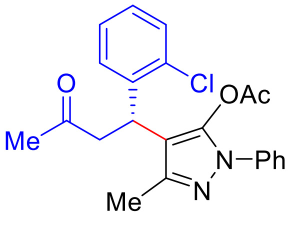

***ent*-3ha**

(*S*)-4-(1-(2-Chlorophenyl)-3-oxobutyl)-3-methyl-1-phenyl-1*H*-pyrazol-5-yl acetate (***ent*-3ha**)

Purified by column chromatography on silica gel (230-400 mesh) using eluent 15% EtOAc/ petroleum ether-20%EtOAc/petroleum ether. The above titled compound was isolated as yellow liquid (58 mg, 73%). The ee was determined by HPLC using a Daicel CHIRALCEL® OJ-H [hexane/*i*-PrOH (70/30)]; flow rate 1.0 mL/min; λ = 220 nm; τ_{major} = 31.61 τ_{minor} = 58.62 min, $[\alpha]_D^{24}$ = -15.0 (*c* 1.5, $CHCl_3$, 98% ee).

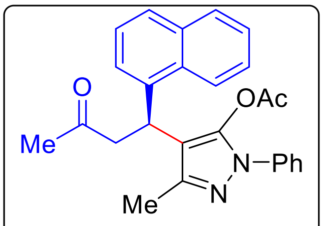

3ia

(*S*)-3-Methyl-4-(1-(naphthalen-1-yl)-3-oxobutyl)-1-phenyl-1*H*-pyrazol-5-yl acetate (3ia)

Purified by column chromatography on silica gel (230-400 mesh) using eluent 15% EtOAc/ petroleum ether-20% EtOAc/petroleum ether. The above titled compound was isolated as white solid (64 mg, >77.5%). M.p.: 130°C –131.0°C; **^{1}H NMR (500 MHz, $CDCl_3$)**: *δ* 8.03 (d, *J* = 8.3 Hz, 1 H), 7.85 (d, *J* = 7.6 Hz, 1 H), 7.75 (d, *J* = 8.0 Hz, 1 H), 7.52-7.46 (m, 2 H), 7.43-7.35 (m, 6 H), 7.28-7.25 (m, 1 H), 5.27 (t, *J* = 7.4 Hz, 1 H), 3.33 (dd, *J* = 17.4, 7.5 Hz, 1 H), 3.19 (dd, *J* = 17.4, 7.5 Hz, 1 H), 2.24 (s, 3 H), 2.20 (s, 3 H), 1.98 (s, 3 H); **^{13}C NMR (125 MHz, $CDCl_3$)**: *δ* 206.4, 167.5, 147.4, 141.4, 137.8, 137.0, 134.0, 131.3, 129.1 (2 C), 128.9, 127.6, 127.2, 126.3, 125.6, 124.9, 123.7, 123.3, 122.8 (2 C), 110.1, 47.4, 31.3, 30.4, 20.2, 13.5; The ee was determined by HPLC using a Daicel CHIRALCEL® OD-H [hexane/*i*-PrOH (80/20)]; flow rate 0.5 mL/min; λ = 220 nm; τ_{minor} = 21.02min, τ_{major} = 27.22min, $[\alpha]_D^{26}$ = -17.2 (*c* 1.2, $CHCl_3$, 98%ee); **HRMS (ESI)** calcd for $C_{26}H_{24}N_2O_3K$ $[M + K]^+$: 451.1424, found: 441.1436.

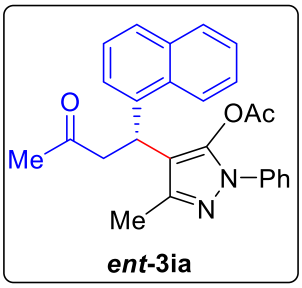

(*R*)-3-Methyl-4-(1-(naphthalen-1-yl)-3-oxobutyl)-1-phenyl-1*H*-pyrazol-5-yl acetate (*ent*-3ia)

Purified by column chromatography on silica gel (230-400 mesh) using eluent 15% 15% EtOAc/ petroleum ether-20% EtOAc/petroleum ether. The above titled compound was isolated as white solid (59 mg, >71.5%). M.p.: 129.0°C –130°C; The ee was determined by HPLC using a Daicel CHIRALCEL® OD-H [hexane/*i*-PrOH (80/20)]; flow rate 0.5 mL/min; $\lambda = 220$ nm; $\tau_{major} = 20.12$min, $\tau_{minor} = 28.35$min, $[\alpha]_D^{26} = +15.8$ (*c* 1.2, $CHCl_3$, 98% ee).

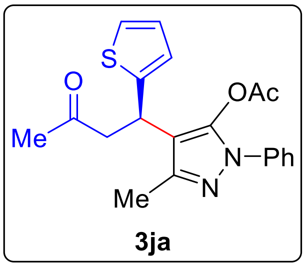

(*R*)-3-Methyl-4-(3-oxo-1-(thiophen-2-yl)butyl)-1-phenyl-1*H*-pyrazol-5-yl acetate (3ja)

Purified by column chromatography on silica gel (230-400 mesh) using eluent 15% EtOAc/ petroleum ether-20% EtOAc/petroleum ether. The above titled compound was isolated as light-yellow liquid (59 mg, 80%). **^{1}H NMR (500 MHz, $CDCl_3$)**: δ 7.47-7.46 (m, 2 H), 7.40 (t, J = 8.1 Hz, 2 H), 7.29 (t, J = 7.3 Hz, 1 H), 7.14 (d, J = 5.1 Hz, 1 H), 6.90 (dd, J = 5.0, 3.6 Hz, 1 H), 6.80-6.79 (m, 1 H), 4.72 (t, J = 7.2 Hz, 1 H), 3.25 (dd, J = 17.2, 7.5 Hz, 1 H), 3.08 (dd, J = 17.2, 6.9 Hz, 1 H), 2.22 (s, 3 H), 2.17 (s, 3 H), 2.15 (s, 3 H); **^{13}C NMR (125 MHz, $CDCl_3$)**: δ 205.8, 167.3, 147.3, 146.3, 141.2, 137.9, 129.1 (2 C), 127.3, 126.6, 124.1, 123.8, 122.8 (2 C), 110.7, 48.7, 30.4, 30.3, 20.3, 13.5; The ee was determined by HPLC using a Daicel CHIRALCEL® OD-H [hexane/*i*-PrOH (80/20)];

flow rate 0.5 mL/min; λ = 220 nm; τ_{minor} = 15.36 min, τ_{major} = 17.71 min, $[\alpha]_D^{25}$ = -22.7 (*c* 2.7, $CHCl_3$, 90% ee); **HRMS (ESI)** calcd for $C_{20}H_{20}N_2O_3SNa$ $[M + Na]^+$: 391.1092, found: 391.1102.

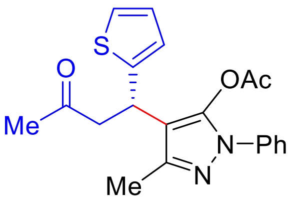

***ent*-3ja**

(*S*)-3-Methyl-4-(3-oxo-1-(thiophen-2-yl)butyl)-1-phenyl-1*H*-pyrazol-5-yl acetate (*ent*-3ja)

Purified by column chromatography on silica gel (230-400 mesh) using eluent 15% EtOAc/ petroleum ether-20% EtOAc/petroleum ether. The above titled compound was isolated as light-yellow liquid (54 mg, 73%). The ee was determined by HPLC using a Daicel CHIRALCEL® OD-H [hexane/*i*-PrOH (80/20)]; flow rate 0.5 mL/min; λ = 220 nm; τ_{major} = 15.60 min, τ_{minor} = 18.2 min, $[\alpha]_D^{25}$ = +16.2 (*c* 1.0, $CHCl_3$, 83.5% ee).

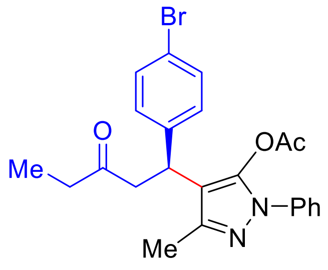

3ka

(*S*)-4-(1-(4-Bromophenyl)-3-oxopentyl)-3-methyl-1-phenyl-1*H*-pyrazol-5-yl acetate (3ka)

Purified by column chromatography on silica gel (230-400 mesh) using eluent 15% EtOAc/ petroleum ether-20%EtOAc/petroleum ether. The above titled compound was isolated as colourless liquid (83 mg, 91%) which solidify in refrigerator on standing. M.p.: 100°C–101°C (hexane/*i*-PrOH :95/5); **^{1}H NMR (500 MHz, $CDCl_3$)**: *δ* 7.45-7.43 (m, 2 H), 7.42-7.39 (m, 4 H), 7.30 (d, *J* = 7.2 Hz, 1 H), 7.10 (d, *J* = 9.2 Hz, 2 H), 4.48 (t, *J* = 7.4 Hz, 1 H), 3.11 (dd, *J* = 16.9, 7.9 Hz, 1 H), 3.01 (dd, *J* = 16.9, 6.7 Hz, 1 H), 2.40 (q, *J* = 7.1 Hz, 2 H), 2.14 (s, 3 H), 2.13 (s, 3 H), 1.02 (t, *J* = 7.3 Hz, 3 H);

^{13}C NMR (125 MHz, $CDCl_3$): δ 208.8, 167.5, 147.4, 141.3, 141.1, 137.7, 131.5 (2 C), 129.2 (2 C), 129.2 (2 C), 127.4, 122.8 (2 C), 120.3, 110.4, 46.0, 36.5, 34.3, 20.3, 13.5, 7.6; The ee was determined by HPLC using a Daicel CHIRALCEL® OD-H [hexane/*i*-PrOH (80/20)]; flow rate 0.5 mL/min; λ = 220 nm; τ_{minor} = 11.55 min, τ_{major} = 12.73 min, $[\alpha]_D^{24}$ = -9.4 (*c* 1.0, $CHCl_3$, >99.5% ee); **HRMS (ESI)** calcd for $C_{23}H_{24}BrN_2O_3$ $[M + H]^+$: 455.0971, found: 455.0977.

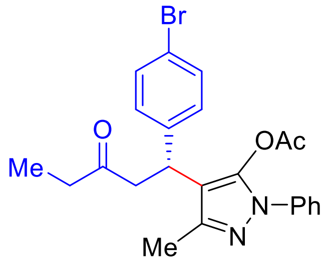

***ent*-3ka**

(*R*)-4-(1-(4-Bromophenyl)-3-oxopentyl)-3-methyl-1-phenyl-1*H*-pyrazol-5-yl acetate (*ent*-3ka)

Purified by column chromatography on silica gel (230-400 mesh) using eluent 15% EtOAc/ petroleum ether-20% EtOAc/petroleum ether.The above titled compound was isolated as colourless liquid (83 mg, 91%) which solidify in refrigerator on standing. M.p.: 100°C–101°C (hexane/*i*-PrOH :95/5); The ee was determined by HPLC using a Daicel CHIRALCEL® OD-H [hexane/*i*-PrOH (80/20)]; flow rate 0.5 mL/min; λ = 220 nm; τ_{major} = 11.46 min, τ_{minor} = 12.77 min, $[\alpha]_D^{25}$ = +6.6 (*c* 1.0, $CHCl_3$, 96% ee);

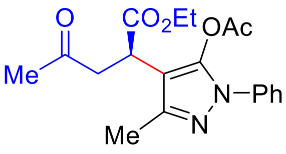

(–)3la

Ethyl (*R*)-2-(5-acetoxy-3-methyl-1-phenyl-1*H*-pyrazol-4-yl)-4-oxopentanoate (-)3la

Purified by column chromatography on silica gel (230-400 mesh) using eluent 15% EtOAc/ petroleum ether-25% EtOAc/petroleum ether. The above titled compound was isolated as light-

yellow liquid (49 mg, >68%). **^{1}H NMR (500 MHz, $CDCl_3$)**: δ 7.46-7.44 (m, 2 H), 7.39 (t, J = 7.8 Hz, 2 H), 7.29 (t, J = 7.3 Hz, 1 H), 4.15 (dq, J = 14.3, 7.2 Hz, 1 H), 4.06 (dq, J = 14.3, 7.1 Hz, 1 H), 3.98 (dd, J = 10.2, 4.0 Hz, 1 H), 3.35 (dd, J = 18.1, 10.2, Hz, 1 H), 2.59 (dd, J = 18.1, 4.0, Hz, 1 H), 2.28 (s, 3 H), 2.20 (s, 3 H), 2.18 (s, 3 H), 1.22 (t, J = 7.2 Hz, 3 H); **^{13}C NMR (125 MHz, $CDCl_3$)**: δ 206.3, 172.1, 167.4, 147.3, 141.6, 137.6, 129.1 (2 C), 127.4, 122.7 (2 C), 105.8, 61.2, 44.2, 35.1, 29.9, 20.2, 14.0, 13.0; The ee was determined by HPLC using a Daicel CHIRALCEL® OD-H [hexane/*i*-PrOH (80/20)]; flow rate 0.5 mL/min; λ = 220 nm; τ_{minor} = 12.27 min, τ_{major} = 14.08 min, $[\alpha]_D^{23}$ = -58.1 (c 3.2, CH_2Cl_2, 95% ee); **HRMS (ESI)** calcd for $C_{19}H_{22}NaN_2O_5$ $[M + Na]^+$: 381.1426, found: 381.1442.

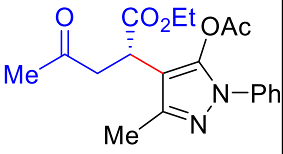

(+)*ent*-3la

Ethyl (*S*)-2-(5-acetoxy-3-methyl-1-phenyl-1*H*-pyrazol-4-yl)-4-oxopentanoate (+)3la

Purified by column chromatography on silica gel (230-400 mesh) using eluent 15% EtOAc/ petroleum ether-20% EtOAc/petroleum ether. The above titled compound was isolated as light-yellow liquid (51 mg, 71%). The ee was determined by HPLC using a Daicel CHIRALCEL® OD-H [hexane/*i*-PrOH (80/20)]; flow rate 0.5 mL/min; λ = 220 nm; τ_{major} = 12.21 min, τ_{minor} = 14.32 min, $[\alpha]_D^{22}$ = +59.1 (c 4.1, CH_2Cl_2, 90% ee);

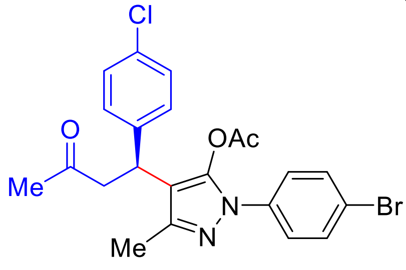

3cb

(*S*)-1-(4-Bromophenyl)-4-(1-(4-chlorophenyl)-3-oxobutyl)-3-methyl-1*H*-pyrazol-5-yl acetate (3cb)

Purified by column chromatography on silica gel (230-400 mesh) using eluent 15% EtOAc/ petroleum ether-20% EtOAc/petroleum ether. The above titled compound was isolated as white solid (74 mg, 77.5 %). M.p.: 130.0°C –131.0°C ([n-hexane/*i*-PrOH (95/5)]; **^{1}H NMR (500 MHz, $CDCl_3$)**: δ 7.52 (d, J = 8.6 Hz, 2 H), 7.34 (d, J = 8.5 Hz, 2 H), 7.24 (d, J = 8.5 Hz, 2 H), 7.14 (d, J = 8.3 Hz, 2 H), 4.46 (t, J = 7.2 Hz, 1 H), 3.14 (dd, J = 17.3, 7.8 Hz, 1 H), 3.02 (dd, J = 17.2, 7.0 Hz, 1 H), 2.16 (s, 3 H), 2.14 (s, 3 H), 2.12 (s, 3 H); **^{13}C NMR (125 MHz, $CDCl_3$)**: δ 205.9, 167.3, 147.9, 141.2, 140.3, 136.9, 132.3 (2 C), 132.26, 128.8 (2 C), 128.6 (2 C), 124.1 (2 C), 120.8, 110.8, 47.2, 34.1, 30.4, 20.4, 13.6; The ee was determined by HPLC using a Daicel CHIRALCEL® OD-H [hexane/*i*-PrOH (80/20)]; flow rate 0.5 mL/min; λ = 220 nm; τ_{minor} = 12.77 min, τ_{major} = 13.78 min, $[\alpha]_D^{25}$ = -6.5 (*c* 1.0, $CHCl_3$, 95% ee); **HRMS (ESI)** calcd for $C_{22}H_{21}BrClN_2O_3$ $[M + H]^+$: 475.0424, found: 475.0432.

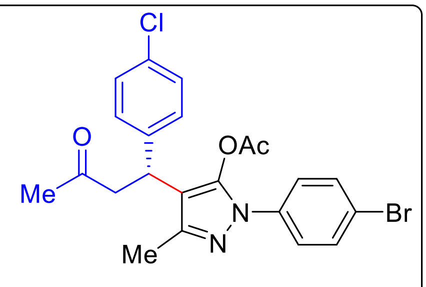

***ent*-3cb**

(*S*)-1-(4-Bromophenyl)-4-(1-(4-chlorophenyl)-3-oxobutyl)-3-methyl-1*H*-pyrazol-5-yl acetate (*ent*-3cb)

Purified by column chromatography on silica gel (230-400 mesh) using eluent 15% EtOAc/ petroleum ether-20% EtOAc/petroleum ether. The above titled compound was isolated as white solid (76 mg, >79.5%). M.p.: 130.0°C –131.0°C ([hexane/*i*-PrOH (95/5)]; The ee was determined by

HPLC using a Daicel CHIRALCEL® OD-H [hexane/*i*-PrOH (80/20)]; flow rate 0.5 mL/min; λ = 220 nm; τ_{minor} = 12.76 min, τ_{major} = 13.87 min, $[\alpha]_D^{25}$ = +6.0 (*c* 1.0, $CHCl_3$, >95.5% ee);

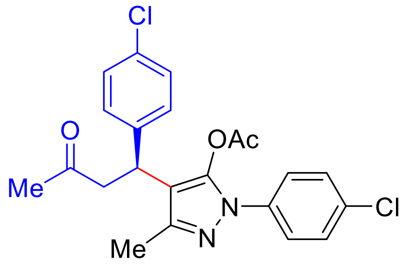

3cc

(*S*)-1-(4-Chlorophenyl)-4-(1-(4-chlorophenyl)-3-oxobutyl)-3-methyl-1*H*-pyrazol-5-yl acetate (3cc)

Purified by column chromatography on silica gel (230-400 mesh) using eluent 15% EtOAc/ petroleum ether-20% EtOAc/petroleum ether. The above titled compound was isolated as white solid (74 mg, >85.5 %). M.p.: 129.0°C –130.0°C ([hexane/*i*-PrOH (95/5)]; **^{1}H NMR (500 MHz, $CDCl_3$)**: *δ* 7.40-7.36 (m, 4 H), 7.24 (d, *J* = 8.5 Hz, 2 H), 7.14 (d, *J* = 8.4 Hz, 2 H), 4.46 (t, *J* = 7.3 Hz, 1 H), 3.13 (dd, *J* = 17.3, 7.8 Hz, 1 H), 3.02 (dd, *J* = 17.3, 7.0 Hz, 1 H), 2.15 (s, 3 H), 2.14 (s, 3 H), 2.12 (s, 3 H); **^{13}C NMR (125 MHz, $CDCl_3$)**: *δ* 205.8, 167.3, 147.8, 141.3, 140.3, 136.4, 132.8, 132.3, 129.3 (2 C), 128.8 (2 C), 128.5 (2 C), 123.9 (2 C), 110.7, 47.2, 34.1, 30.3, 20.3, 13.5; The ee was determined by HPLC using a Daicel CHIRALCEL® OD-H [hexane/*i*-PrOH (80/20)]; flow rate 0.5 mL/min; λ = 220 nm; τ_{minor} = 12.51 min, τ_{major} = 13.47 min, $[\alpha]_D^{25}$ = -15 (*c* 1.0, $CHCl_3$, 98% ee); **HRMS (ESI)** calcd for $C_{22}H_{21}Cl_2N_2O_3$ [M + H]$^+$: 431.0925, found: 431.0914.

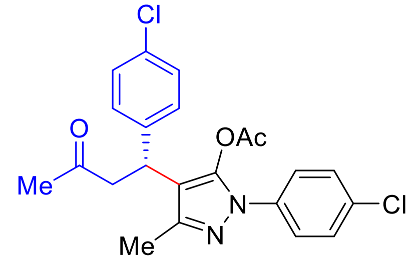

***ent*-3cc**

(*R*)-1-(4-Chlorophenyl)-4-(1-(4-chlorophenyl)-3-oxobutyl)-3-methyl-1*H*-pyrazol-5-yl acetate (*ent*-3cc)

Purified by column chromatography on silica gel (230-400 mesh) using eluent 15% EtOAc/ petroleum ether-20% EtOAc/petroleum ether. The above titled compound was isolated as white solid (70 mg, 81%). M.p.: 129.5°C –130.0°C ([hexane/*i*-PrOH (95/5)]; The ee was determined by HPLC using a Daicel CHIRALCEL® OD-H [hexane/*i*-PrOH (80/20)]; flow rate 0.5 mL/min; λ = 220 nm; τ_{major} = 12.53 min, τ_{minor} = 13.59 min, $[\alpha]_D^{25}$ = +13.1 (*c* 1.0, $CHCl_3$, 95% ee).

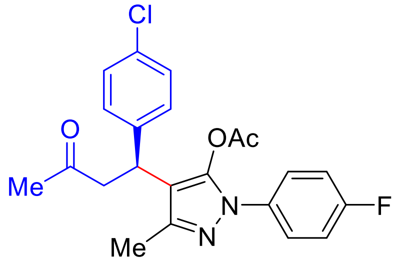

3cd

(*S*)-4-(1-(4-Chlorophenyl)-3-oxobutyl)-1-(4-fluorophenyl)-3-methyl-1*H*-pyrazol-5-yl acetate (3cd)

Purified by column chromatography on silica gel (230-400 mesh) using eluent 15% EtOAc/ petroleum ether-25% EtOAc/petroleum ether. The above titled compound was isolated as yellow thick liquid (67 mg, 80.5%).**^{1}H NMR (500 MHz, $CDCl_3$)**: δ 7.42-7.39 (m, 2 H), 7.24 (d, *J* = 8.5 Hz, 2 H), 7.15 (d, *J* = 8.4 Hz, 2 H), 7.08 (t, *J* = 8.6 Hz, 2 H), 4.46 (t, *J* = 7.3 Hz, 1 H), 3.13 (dd, *J* = 17.2, 7.8 Hz, 1 H), 3.03 (dd, *J* = 17.2, 6.9 Hz, 1 H), 2.14 (s, 3 H), 2.12 (s, 6 H); **^{13}C NMR (125 MHz,**

CDCl3): δ 205.9, 167.4, 161.5 (d, $J_{C\text{-}F}$ = 246.2 Hz), 147.5, 141.3, 140.4, 133.9 (d, $J_{C\text{-}F}$ = 12.1 Hz), 132.2, 128.8 (2 C), 128.5 (2 C), 124.9 (d, $J_{C\text{-}F}$ = 34.2 Hz, 2 C), 116.0 (d, $J_{C\text{-}F}$ = 22.8 Hz, 2 C), 110.3, 42.2, 34.1, 30.4, 20.3, 13.5; The ee was determined by HPLC using a Daicel CHIRALCEL® OD-H [hexane/*i*-PrOH (80/20)]; flow rate 0.5 mL/min; λ = 220 nm; τ_{minor} = 12.49 min, τ_{major} = 13.68 min, $[\alpha]_D^{22}$ = -4.8 (*c* 2.0, $CHCl_3$, 93% ee); **HRMS (ESI)** calcd for $C_{22}H_{21}ClFN_2O_3$ $[M + H]^+$: 415.1224, found: 415.1229.

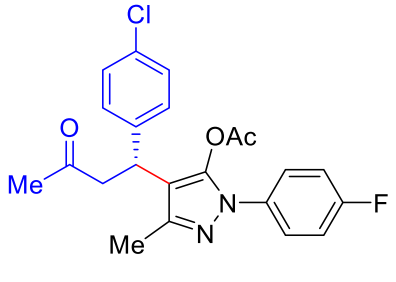

***ent*-3cd**

(*R*)-4-(1-(4-Chlorophenyl)-3-oxobutyl)-1-(4-fluorophenyl)-3-methyl-1*H*-pyrazol-5-yl acetate (*ent*-3cd)

Purified by column chromatography on silica gel (230-400 mesh) using eluent 15% EtOAc/ petroleum ether-25% EtOAc/petroleum ether. The above titled compound was isolated as yellow thick liquid (65 mg, 78%). The ee was determined by HPLC using a Daicel CHIRALCEL® OD-H [hexane/*i*-PrOH (80/20)]; flow rate 0.5 mL/min; λ = 220 nm; τ_{major} = 12.52 min, τ_{minor} = 13.86 min, $[\alpha]_D^{21}$ = +4.6 (*c* 2.3, $CHCl_3$, 89% ee);

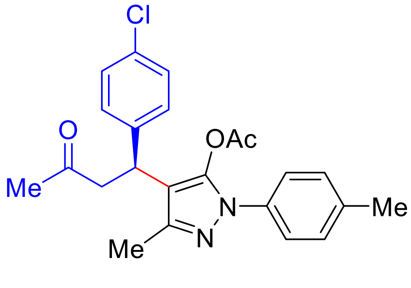

3ce

(*S*)-4-(1-(4-Chlorophenyl)-3-oxobutyl)-3-methyl-1-(*p*-tolyl)-1*H*-pyrazol-5-yl acetate (3ce)

Purified by column chromatography on silica gel (230-400 mesh) using eluent 15% EtOAc/ petroleum ether-20% EtOAc/petroleum ether. The above titled compound was isolated as yellow thick liquid (64 mg, 78%).**^{1}H NMR (500 MHz, $CDCl_3$)**: δ 7.30 (d, J = 8.4 Hz, 2 H), 7.24 (d, J = 8.5 Hz, 2 H), 7.19 (d, J = 8.2 Hz, 2 H), 7.15 (d, J = 8.4 Hz, 2 H), 4.46 (t, J = 7.3 Hz, 1 H), 3.14 (dd, J = 17.2, 8.0 Hz, 1 H), 3.03 (dd, J = 17.2, 6.8 Hz, 1 H), 2.35 (s, 3 H), 2.14 (s, 3 H), 2.13 (s, 3 H), 2.12 (s, 3 H); **^{13}C NMR (125 MHz, $CDCl_3$)**: δ 206.1, 167.5, 147.1, 141.2, 140.5, 137.2, 135.3, 132.1, 129.7 (2 C), 128.8 (2 C), 128.5 (2 C), 122.7 (2 C), 110.0, 47.3, 34.1, 30.4, 21.0, 20.3, 13.5; The ee was determined by HPLC using a Daicel CHIRALCEL® OD-H [hexane/*i*-PrOH (80/20)]; flow rate 0.5 mL/min; λ = 220 nm; τ_{minor} = 13.37 min, τ_{major} = 16.02 min, $[\alpha]_D^{24}$ = -1.8 (*c* 1.4, $CHCl_3$, 89.5% ee); **HRMS (ESI)** calcd for $C_{23}H_{24}ClN_2O_3$ $[M + H]^+$: 411.1475, found: 411.1478.

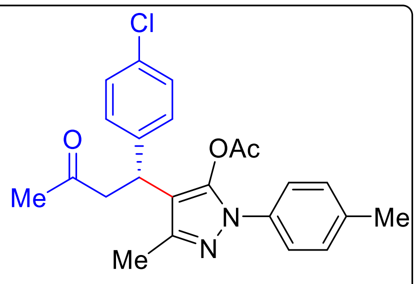

***ent*-3ce**

(*R*)-4-(1-(4-Chlorophenyl)-3-oxobutyl)-3-methyl-1-(*p*-tolyl)-1*H*-pyrazol-5-yl acetate (*ent*-3ce)

Purified by column chromatography on silica gel (230-400 mesh) using eluent 15% EtOAc/ petroleum ether-20%EtOAc/petroleum ether. The above titled compound was isolated as yellow thick liquid (69 mg, 84%). The ee was determined by HPLC using a Daicel CHIRALCEL® OD-H [hexane/*i*-PrOH (80/20)]; flow rate 0.5 mL/min; λ = 220 nm; τ_{major} = 13.25 min, τ_{minor} = 16.15 min, $[\alpha]_D^{24}$ = +1.8 (*c* 1.4, $CHCl_3$, 84.5% ee).

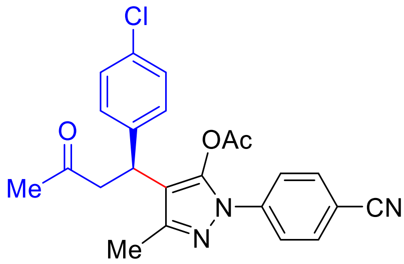

3cf

(*S*)-4-(1-(4-Chlorophenyl)-3-oxobutyl)-1-(4-cyanophenyl)-3-methyl-1*H*-pyrazol-5-ylacetate (3cf)

Purified by column chromatography on silica gel (230-400 mesh) using eluent 25% EtOAc/ petroleum ether-35% EtOAc/petroleum ether. The above titled compound was isolated as off-white amorphous solid (52 mg, 61.5%).**^{1}H NMR (500 MHz, $CDCl_3$)**: δ 7.69 (d, *J* = 8.2 Hz, 2 H), 7.63 (d, *J* = 8.7 Hz, 2 H), 7.25 (d, *J* = 8.4 Hz, 2 H), 7.13 (d, *J* = 8.5 Hz, 2 H), 4.46 (t, *J* = 7.3 Hz, 1 H), 3.15 (dd, *J* = 17.4, 7.5 Hz, 1H), 3.03 (dd, *J* = 17.4, 7.1 Hz, 1 H), 2.21 (s, 3 H), 2.15 (s, 3 H), 2.13 (s, 3 H); **^{13}C NMR (125 MHz, $CDCl_3$)**: δ 205.7, 167.1, 149.1, 141.5, 141.3, 139.9, 133.2 (2 C), 132.4, 128.7 (2 C), 128.6 (2 C), 121.9 (2 C), 118.2, 111.9, 110.1, 46.9, 33.8, 30.4, 20.4, 13.6; The ee was determined by HPLC using a Daicel CHIRALCEL® OD-H [hexane/*i*-PrOH (80/20)]; flow rate 0.5 mL/min; λ = 220 nm; τ_{minor} = 22.61 min, τ_{major} = 24.17 min, $[\alpha]_D^{23}$ = -11.0 (*c* 2.8, $CHCl_3$, 95% ee); **HRMS (ESI)** calcd for $C_{23}H_{21}ClN_3O_3$ $[M + H]^+$: 422.1271, found: 422.1268.

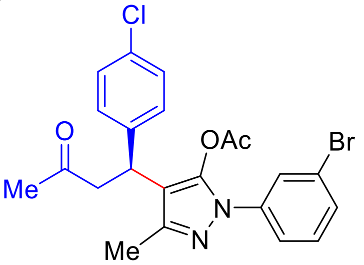

3cg

(*S*)-1-(3-Bromophenyl)-4-(1-(4-chlorophenyl)-3-oxobutyl)-3-methyl-1*H*-pyrazol-5-yl acetate (3cg)

Purified by column chromatography on silica gel (230-400 mesh) using eluent 15% EtOAc/ petroleum ether-20%EtOAc/petroleum ether. The above titled compound was isolated as off-white amorphous solid (83 mg, 87%).**^{1}H NMR (500 MHz, CDCl$_3$)**: δ 7.66 (t, J = 1.8 Hz, 1H), 7.42-7.40 (m, 2 H), 7.26-7.24 (m, 3 H), 7.14 (d, J = 8.4 Hz, 2 H), 4.46 (t, J = 7.3 Hz, 1H), 3.14 (dd, J = 17.3, 7.7 Hz, 1H), 3.03 (dd, J = 17.2, 7.0 Hz, 1H), 2.18 (s, 3 H), 2.14 (s, 3 H), 2.12 (s, 3 H); **^{13}C NMR (125 MHz, CDCl$_3$)**: δ 205.8, 167.3, 148.0, 141.3, 140.2, 138.9, 132.2, 130.4, 130.0, 128.8 (2 C), 128.5 (2 C), 125.4, 122.6, 120.7, 111.0, 47.1, 34.0, 30.3, 20.3, 13.5; The ee was determined by HPLC using a Daicel CHIRALCEL® OD-H [hexane/*i*-PrOH (80/20)]; flow rate 0.5 mL/min; λ = 220 nm; τ_{minor} = 12.95 min, τ_{major} = 13.99 min, $[\alpha]_D^{21}$ = -4.2 (*c* 2.0, CHCl$_3$, 95.5% ee); **HRMS (ESI)** calcd for $C_{22}H_{21}BrClN_2O_3$ [M + H]$^+$: 475.0424, found: 475.0432.

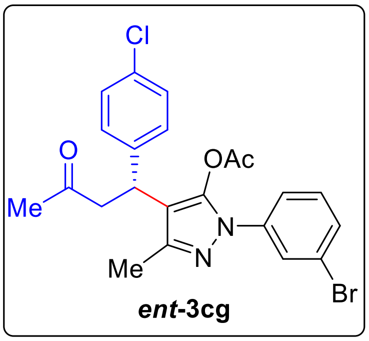

***ent*-3cg**

(*R*)-1-(3-Bromophenyl)-4-(1-(4-chlorophenyl)-3-oxobutyl)-3-methyl-1*H*-pyrazol-5-yl acetate (ent-3cg)

Purified by column chromatography on silica gel (230-400 mesh) using eluent 15% EtOAc/ petroleum ether-20% EtOAc/petroleum ether. The above titled compound was isolated as off-white amorphous solid (83 mg, 87%). The ee was determined by HPLC using a Daicel CHIRALCEL® OD-H [hexane/*i*-PrOH (80/20)]; flow rate 0.5 mL/min; λ = 220 nm; τ_{major} = 12.91 min, τ_{minor} = 14.00 min, $[\alpha]_D^{24}$ = +2.5 (*c* 1.7, CHCl$_3$, 85% ee);

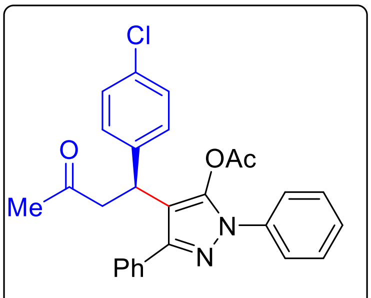

3ch

(*S*)-4-(1-(4-Chlorophenyl)-3-oxobutyl)-1,3-diphenyl-1*H*-pyrazol-5-yl acetate (3ch)

Purified by column chromatography on silica gel (230-400 mesh) using eluent 10% EtOAc/ petroleum ether-20%EtOAc/petroleum ether. The above titled compound was isolated as off-white amorphous solid (80 mg, 87%).**^{1}H NMR (500 MHz, $CDCl_3$)**: δ 7.55 (d, J = 8.0 Hz, 2 H), 7.50 (dd, J = 7.7, 1.8 Hz, 2 H), 7.44 (t, J = 8.0 Hz, 2 H), 7.41-7.37 (m, 3 H), 7.33(t, J = 7.4 Hz, 1 H), 7.24 (d, J = 8.5 Hz, 2 H), 7.14 (d, J = 8.4 Hz, 2 H), 4.73 (t, J = 7.3 Hz, 1H), 3.10 (dd, J = 17.4, 8.2 Hz, 1H), 3.02 (dd, J = 17.3, 6.6 Hz, 1H), 2.09 (s, 3 H), 2.05 (s, 3 H); **^{13}C NMR (125 MHz, $CDCl_3$)**: δ 205.8, 167.1, 150.5, 141.9, 140.9, 137.7, 133.2, 132.1, 129.2 (2 C), 128.9 (2 C), 128.5 (2 C), 128.48 (2 C), 128.4 (2 C), 128.2, 127.7, 123.03 (2 C), 110.1, 47.9, 34.3, 30.0, 20.2; The ee was determined by HPLC using a Daicel CHIRALCEL® OD-H [hexane/*i*-PrOH (80/20)]; flow rate 0.5 mL/min; λ = 220 nm; τ_{minor} = 12.05 min, τ_{major} = 13.44 min, $[\alpha]_D^{20}$= -8.4 (*c* 1.0, $CHCl_3$, 90% ee); **HRMS (ESI)** calcd for $C_{27}H_{24}ClN_2O_3$ $[M + H]^+$: 459.1475, found: 459.1487.

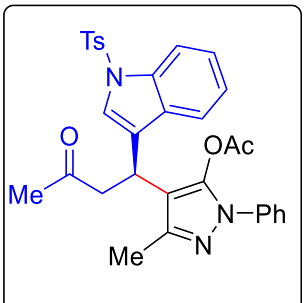

3ma

(*S*)-3-Methyl-4-(3-oxo-1-(1-tosyl-1*H*-indol-3-yl)butyl)-1-phenyl-1*H*-pyrazol-5-yl acetate (3ma)

Purified by column chromatography on silica gel (230-400 mesh) using eluent 25% EtOAc/ petroleum ether-35% EtOAc/petroleum ether. The above titled compound was isolated as colourless amorphous solid (107 mg, 96%).**^{1}H NMR (500 MHz, $CDCl_3$)**: δ 7.95 (d, J = 8.3 Hz, 1 H), 7.70 (d, J = 8.3 Hz, 2 H), 7.42-7.37 (m, 4 H), 7.33-7.27 (m, 4 H), 7.20 (d, J = 8.2 Hz, 2 H), 7.17 (t, J = 7.8 Hz, 1 H), 4.59 (t, J = 7.1 Hz, 1 H), 3.20 (dd, J = 17.1, 6.9 Hz, 1 H), 3.09 (dd, J = 17.0, 6.9 Hz, 1 H), 2.33 (s, 3 H), 2.18 (s, 3 H), 2.13 (s, 3 H), 2.07 (s, 3 H); **^{13}C NMR (125 MHz, $CDCl_3$)**: δ 205.9, 167.4, 147.1, 144.9, 141.3, 137.7, 135.4, 134.9, 129.9, 129.7 (2 C), 129.1 (2 C), 127.2, 126.6 (2 C), 124.9, 123.7, 123.3, 122.7 (2 C), 122.4, 119.9, 113.6, 108.6, 46.7, 30.4, 26.4, 21.4, 20.1, 13.3; The ee was determined by HPLC using a Daicel CHIRALCEL® OD-H [hexane/*i*-PrOH (80/20)]; flow rate 0.5 mL/min; λ = 220 nm; τ_{major} = 29.30 min, τ_{minor} = 33.60 min, $[\alpha]_D^{23}$ = -67.3 (*c* 1.7, $CHCl_3$, 90% ee); **HRMS (ESI)** calcd for $C_{31}H_{30}N_3O_5S$ $[M + H]^+$: 556.1906, found: 556.1908.

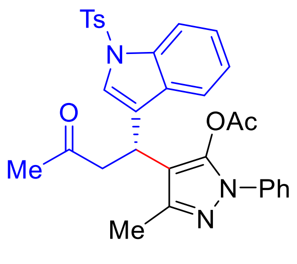

***ent*-3ma**

(*R*)-3-Methyl-4-(3-oxo-1-(1-tosyl-1*H*-indol-3-yl)butyl)-1-phenyl-1*H*-pyrazol-5-yl acetate (ent-3ma)

Purified by column chromatography on silica gel (230-400 mesh) using eluent 25% EtOAc/ petroleum ether-35% EtOAc/petroleum ether. The above titled compound was isolated as colourless amorphous solid (102 mg, 91.5%). The ee was determined by HPLC using a Daicel CHIRALCEL® OD-H [hexane/*i*-PrOH (80/20)]; flow rate 0.5 mL/min; λ = 220 nm; τ_{major} = 30.79 min, τ_{minor} = 34.58 min, $[\alpha]_D^{25}$ = +72.1 (*c* 1.7, $CHCl_3$, 84% ee);

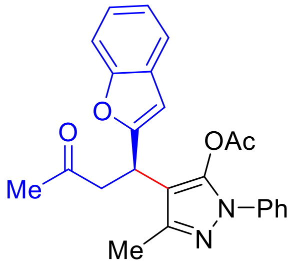

3na

(*R*)-4-(1-(Benzofuran-2-yl)-3-oxobutyl)-3-methyl-1-phenyl-1*H*-pyrazol-5-yl acetate (3na)

Purified by column chromatography on silica gel (230-400 mesh) using eluent 15% EtOAc/ petroleum ether-25% EtOAc/petroleum ether. The above titled compound was isolated as yellowish thick liquid (69 mg, 85.5%).**^{1}H NMR (500 MHz, $CDCl_3$)**: δ 7.47-7.46 (m, 3 H), 7.40 (t, J = 7.8 Hz, 3 H), 7.30 (t, J = 7.5Hz, 1 H), 7.22 (td, J = 7.6, 1.1 Hz, 1 H), 7.18 (t, J = 7.3 Hz, 1 H), 6.42 (s, 1 H), 4.69 (t, J = 7.0 Hz, 1 H), 3.37 (dd, J = 17.5, 7.5 Hz, 1 H), 3.04 (dd, J = 17.5, 6.7 Hz, 1 H), 2.31 (s, 3 H), 2.21 (s, 3 H), 2.08 (s, 3 H); **^{13}C NMR (125 MHz, $CDCl_3$)**: δ 205.6, 167.4, 157.8, 154.7, 147.4, 141.7, 137.8, 129.2 (2 C), 128.4, 127.4, 123.7, 122.8 (2 C), 122.7, 120.6, 110.9, 108.1, 102.9, 45.9, 30.3, 29.4, 20.3, 13.4; The ee was determined by HPLC using a Daicel CHIRALCEL® OD-H [hexane/*i*-PrOH (80/20)]; flow rate 0.5 mL/min; λ = 220 nm; τ_{minor} = 16.24 min, τ_{major} = 17.41min, $[\alpha]_D^{24}$ = -40.3 (*c* 1.3, $CHCl_3$, 95.5% ee); **HRMS (ESI)** calcd for $C_{24}H_{23}N_2O_4$ $[M + H]^+$: 403.1657, found: 403.1657.

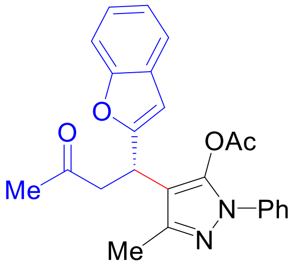

***ent*-3na**

(*S*)-4-(1-(Benzofuran-2-yl)-3-oxobutyl)-3-methyl-1-phenyl-1*H*-pyrazol-5-yl acetate (*ent*-3na)

Purified by column chromatography on silica gel (230-400 mesh) using eluent 15% EtOAc/ petroleum ether-25% EtOAc/petroleum ether. The above titled compound was isolated as yellowish thick liquid (69 mg, 85.5%). The ee was determined by HPLC using a Daicel CHIRALCEL® OD-H [hexane/*i*-PrOH (80/20)]; flow rate 0.5 mL/min; λ = 220 nm; τ_{major} = 16.56 min, τ_{minor} = 18.11 min, $[\alpha]_D^{22}$ = +36.5 (*c* 2.0 $CHCl_3$, 91.5% ee).

Unsuccessful synthetic transformation

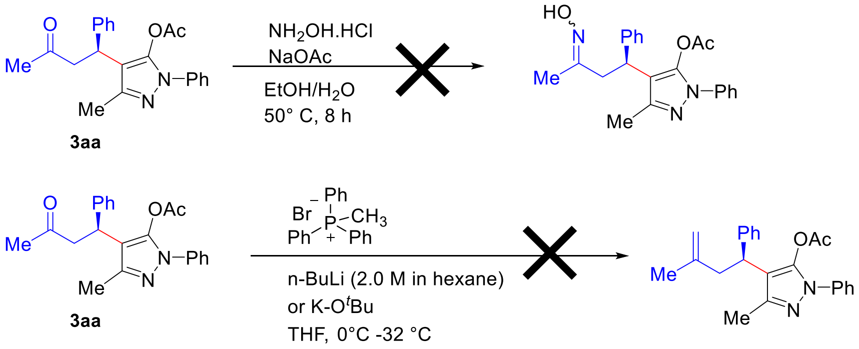

6. Single crystal X-ray diffraction analysis of *ent*-3ba

X-ray structure of *ent*-3ba (CCDC 2234286

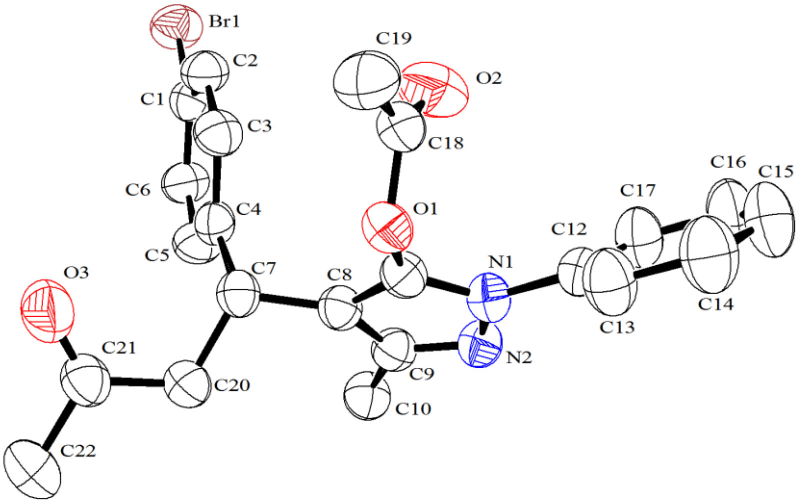

Table 1. Crystal data and structure refinement for BOD-PG-45-new

CCDC number	
Empirical formula	$C_{22}H_{21}BrN_2O_3$
Formula weight	441.32
Temperature [K]	298(2)
Crystal system	monoclinic
Space group (number)	$C2$ (5)
a [Å]	27.4766(3)
b [Å]	6.15110(10)
c [Å]	12.3067(2)
α [Å]	90
β [Å]	100.7590(10)
γ [Å]	90
Volume [Å^3]	2043.41(5)
Z	4
ρ_{calc} [g/cm^3]	1.435
μ [mm^{-1}]	2.946

F(000)	904
Crystal size [mm^3]	0.100×0.050×0.050
Crystal colour	colourless
Crystal shape	needle
Radiation	Cu K_α (λ=1.54184 Å)
2Θ range [°]	6.55 to 154.68<br>(0.79 Å)
Index ranges	-34 ≤ h ≤ 34<br>-7 ≤ k ≤ 7<br>-15 ≤ l ≤ 15
Reflections collected	40865
Independent reflections	4274<br>R_{int} = 0.0848<br>R_{sigma} = 0.0282
Completeness to θ = 67.684°	100.0 %
Data / Restraints / Parameters	4274/1/256
Goodness-of-fit on F^2	1.195
Final R indexes [$I \geq 2\sigma(I)$]	R_1 = 0.0343<br>wR_2 = 0.0993
Final R indexes [all data]	R_1 = 0.0706<br>wR_2 = 0.1550
Largest peak/hole [eÅ^3]	0.64/-0.58
Flack X parameter	-0.031(7)

Table 2. Bond lengths and angles for BOD-PG-45-new.

**Atom–AtomAtom–Atom–Atom**	**Length [Å]Angle [°]**
C1–C6	1.376(8)
C1–C2	1.382(8)
C1–Br1	1.905(5)
C2–C3	1.380(8)
C3–C4	1.396(7)
C4–C5	1.394(7)
C4–C7	1.521(7)
C5–C6	1.388(8)
C7–C8	1.511(6)
C7–C20	1.539(7)
C8–C11	1.371(7)
C8–C9	1.417(8)
C9–N2	1.333(7)
C9–C10	1.489(7)
C11–N1	1.349(7)
C11–O1	1.369(6)
C12–C17	1.373(8)
C12–C13	1.380(8)
C12–N1	1.428(6)
C13–C14	1.388(9)
C14–C15	1.368(11)
C15–C16	1.380(11)
C16–C17	1.380(9)
C18–O2	1.181(9)
C18–O1	1.385(7)
C18–C19	1.488(10)
C20–C21	1.512(8)
C21–O3	1.191(9)
C21–C22	1.507(9)
N1–N2	1.367(7)
C6–C1–C2	121.4(5)
C6–C1–Br1	118.5(4)
C2–C1–Br1	120.0(4)
C3–C2–C1	118.3(5)
C2–C3–C4	122.6(5)
C5–C4–C3	116.8(5)
C5–C4–C7	123.3(5)
C3–C4–C7	119.7(5)
C6–C5–C4	121.8(5)
C1–C6–C5	119.0(5)
C8–C7–C4	109.9(4)
C8–C7–C20	111.3(4)

C4–C7–C20	115.6(4)
C11–C8–C9	103.0(4)
C11–C8–C7	125.1(5)
C9–C8–C7	131.8(5)
N2–C9–C8	112.1(4)
N2–C9–C10	118.3(5)
C8–C9–C10	129.5(5)
N1–C11–O1	122.8(5)
N1–C11–C8	109.4(5)
O1–C11–C8	127.6(5)
C17–C12–C13	121.0(5)
C17–C12–N1	118.5(5)
C13–C12–N1	120.6(5)
C12–C13–C14	118.4(6)
C15–C14–C13	121.3(7)
C14–C15–C16	119.4(6)
C17–C16–C15	120.2(6)
C12–C17–C16	119.7(6)
O2–C18–O1	121.8(6)
O2–C18–C19	129.3(6)
O1–C18–C19	108.9(6)
C21–C20–C7	113.5(4)
O3–C21–C22	121.6(6)
O3–C21–C20	122.9(5)
C22–C21–C20	115.5(6)
C11–N1–N2	110.4(4)
C11–N1–C12	129.1(5)
N2–N1–C12	120.2(4)
C9–N2–N1	105.0(4)
C11–O1–C18	118.2(4)

A colourless block-shaped crystal with dimensions 0.05 × 0.05 × 0.10 mm^3 was mounted. Data were collected using a XtaLAB Synergy, Dualflex, HyPix diffractometer, operating at T = 298(2) K , equipped with a micro-focus sealed X-ray Cu Kα radiation (λ = 1.54184 Å). All data were integrated with CrysAlisPro 1.171.40.57a (Rigaku OD, 2019) and a multi-scan absorption correction using SCALE3 ABSPACK was applied.[1] The structure were solved by iterative methods using SHELXT and refined by full-matrix least-squares methods against F^2 by SHELXL-2017/1.[2,3] All non-hydrogen atoms were refined anisotropically. Hydrogen atom positions were calculated geometrically and refined using the riding model. Hydrogen atom

positions were calculated geometrically and refined using the riding model. ORTEP diagrams were prepared with 50% probability using ORTEP 3 for windows.[4]

Crystallographic data (including structure factors) for the structures reported in this paper have been deposited with the Cambridge Crystallographic Data Centre (CCDC No. 2234286).

[1] CrysAlisPro (Rigaku, V1.171.40.57a, 2019), Rigaku Oxford Diffraction, Poland.

[2] G. M. Sheldrick, *Acta Cryst.***2015**, *A71*, 3–8, doi:10.1107/S2053273314026370.

[3] G. M. Sheldrick, *Acta Cryst.***2015**, *C71*, 3–8, doi:10.1107/S2053229614024218.

[4] L. J. Farrugia, J. Appl. Crystallogr., 1997, 30, 565.

SAMPLE INFORMATION

Sample Name:	PG91-ODH	Acquired By:	
Sample Type:	Unknown	Sample Set Name:	
Vial:	1	Acq. Method Set:	
Injection #:	14	Processing Method	
Injection Volume:	10.00 ul	Channel Name:	
Run Time:	100.0 Minutes	Proc. Chnl. Descr.:	
Date Acquired:	30-11-2022 16:29:04 IST		
Date Processed:	30-11-2022 16:59:44 IST		

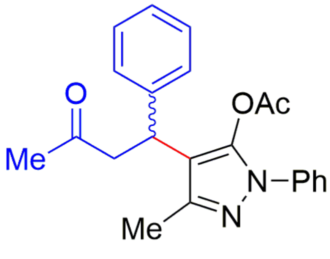

***rac*-3aa**

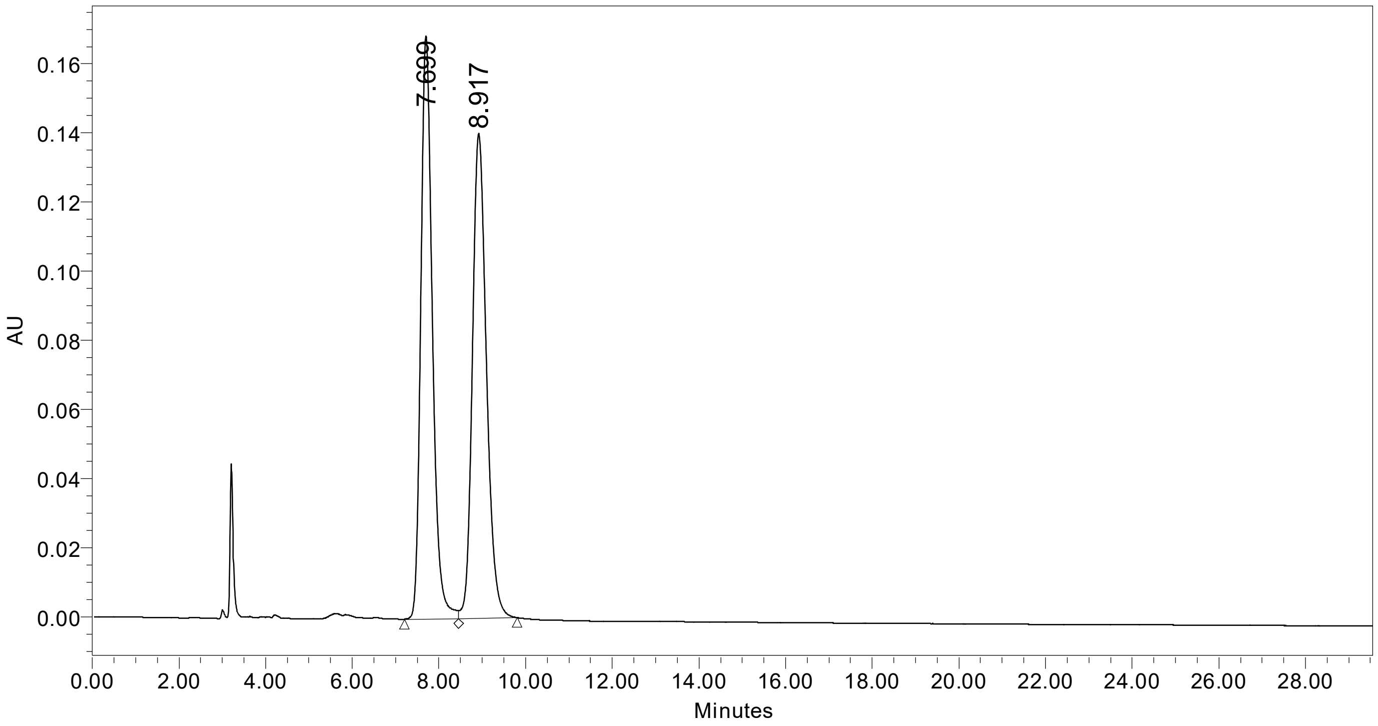

	RT	Area	% Area	Height
1	7.699	3144914	49.91	168804
2	8.917	3156348	50.09	140340

SAMPLE INFORMATION

Sample Name:	PG37-OD-H	Acquired By:	
Sample Type:	Unknown	Sample Set Name:	
Vial:	1	Acq. Method Set:	
Injection #:	4	Processing Method	
Injection Volume:	5.00 ul	Channel Name:	
Run Time:	120.0 Minutes	Proc. Chnl. Descr.:	
Date Acquired:	21-10-2022 19:14:13 IST		
Date Processed:	28-10-2022 14:12:04 IST		

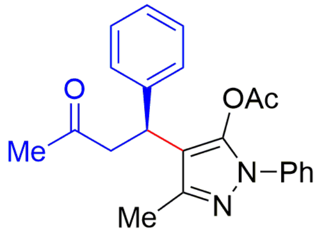

3aa

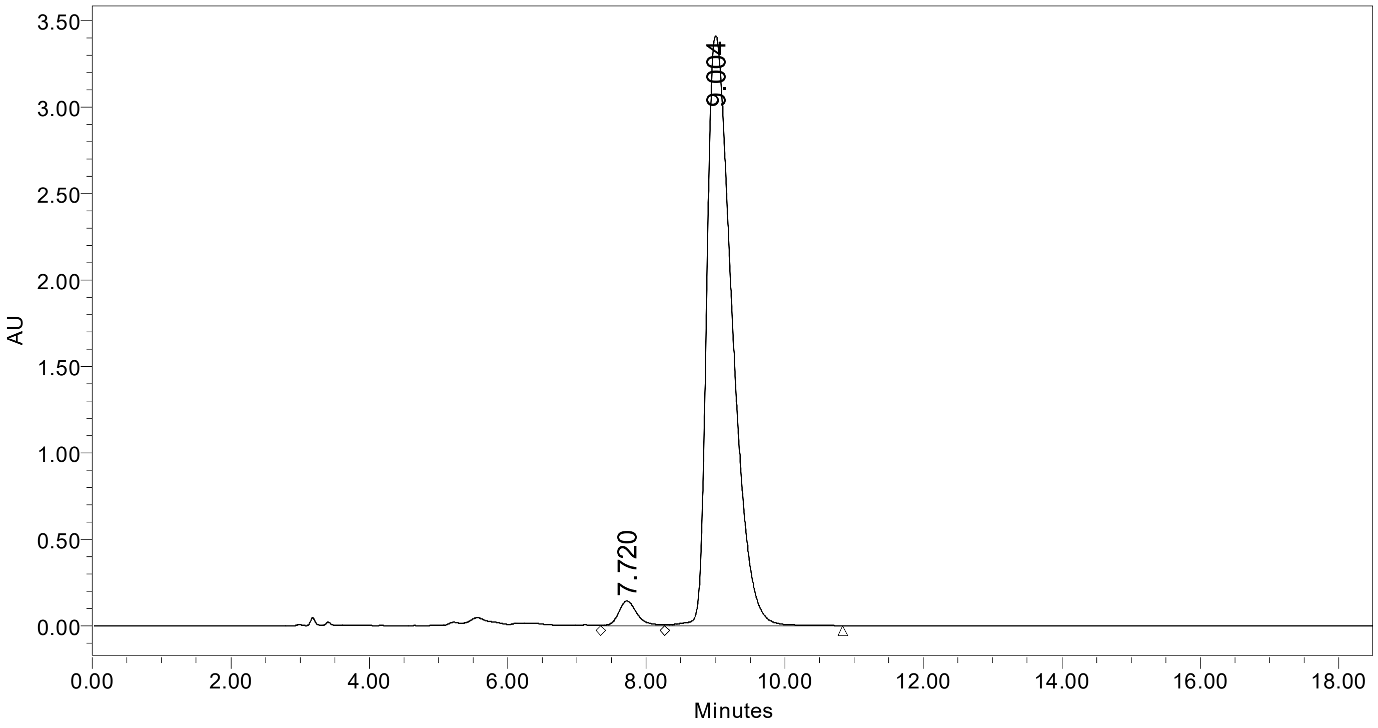

	RT	Area	% Area	Height
1	7.720	2627242	2.96	143104
2	9.004	86150145	97.04	3412864

SAMPLE INFORMATION

Sample Name:	PG-118-ODH	Acquired By:	
Sample Type:	Unknown	Sample Set Name:	
Vial:	1	Acq. Method Set:	
Injection #:	4	Processing Method	
Injection Volume:	10.00 ul	Channel Name:	
Run Time:	100.0 Minutes	Proc. Chnl. Descr.:	
Date Acquired:	05-01-2023 14:58:10 IST		
Date Processed:	21-03-2023 19:23:12 IST		

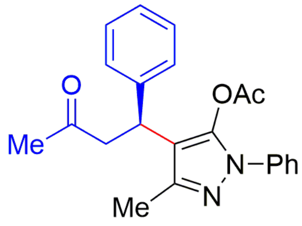

3aa

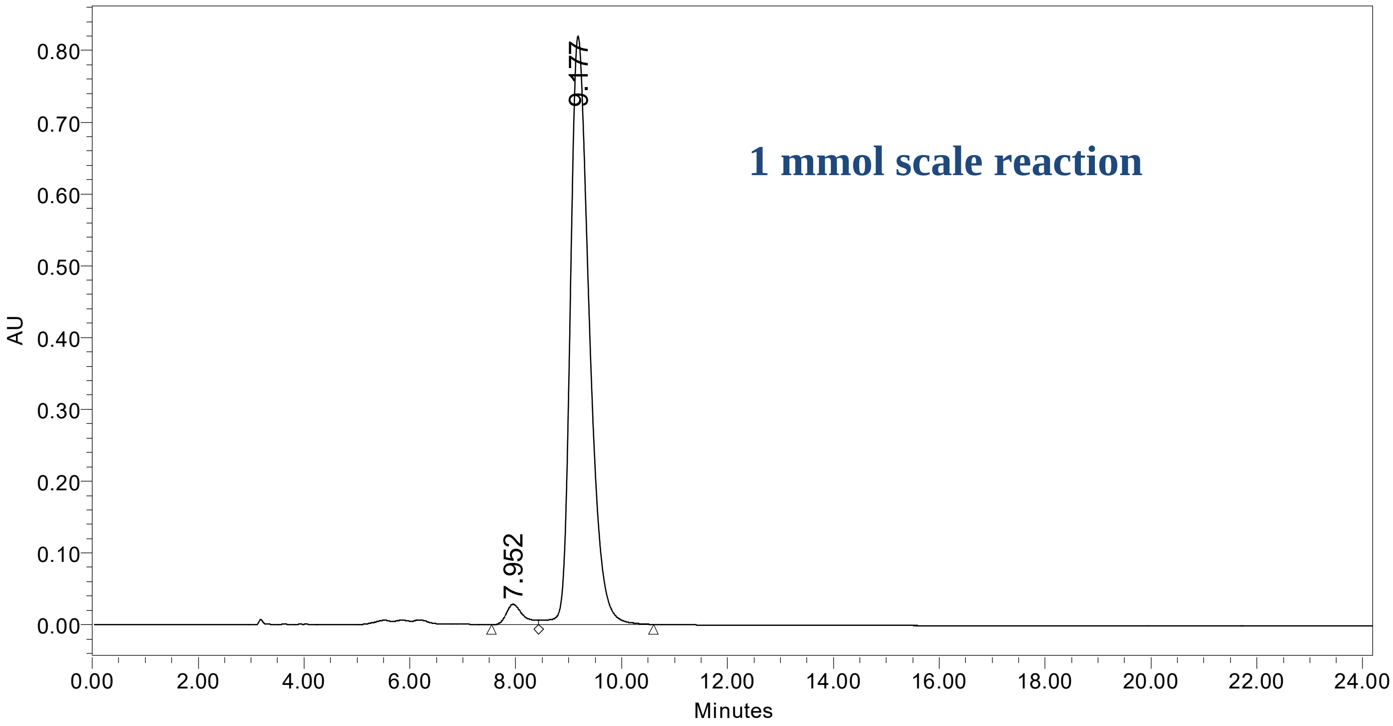

	RT	Area	% Area	Height
1	7.952	631769	3.00	28313
2	9.177	20441814	97.00	820052

SAMPLE INFORMATION

Sample Name:	PG43-OD-H	Acquired By:	
Sample Type:	Unknown	Sample Set Name:	
Vial:	1	Acq. Method Set:	
Injection #:	7	Processing Method	
Injection Volume:	5.00 ul	Channel Name:	
Run Time:	120.0 Minutes	Proc. Chnl. Descr.:	
Date Acquired:	27-10-2022 13:36:52 IST		
Date Processed:	30-11-2022 13:36:32 IST		

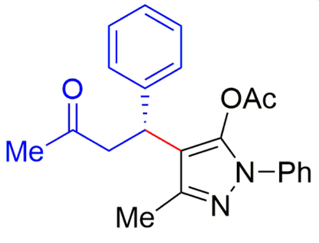

***ent*-3aa**

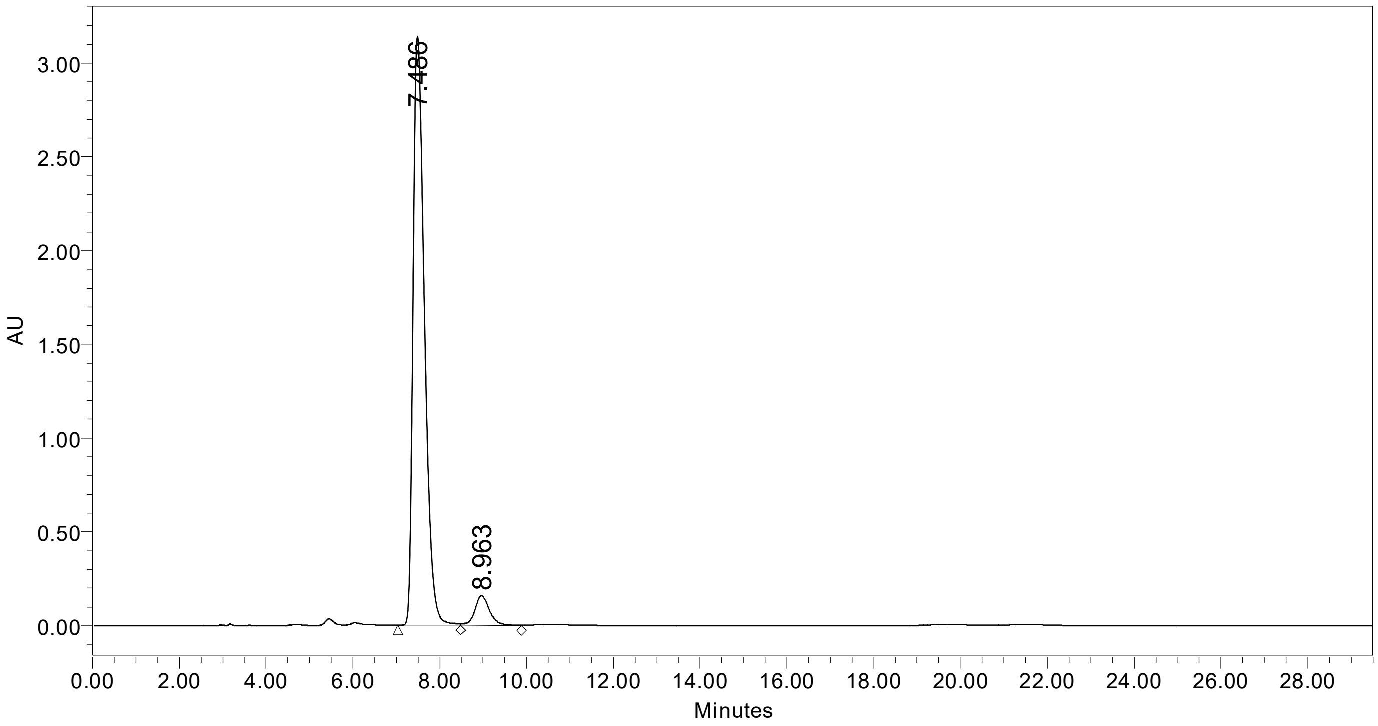

	RT	Area	% Area	Height
1	7.486	57369937	93.83	3143419
2	8.963	3774962	6.17	159145

SAMPLE INFORMATION

Sample Name:	PG93-ODH	Acquired By:	
Sample Type:	Unknown	Sample Set Name:	
Vial:	1	Acq. Method Set:	
Injection #:	5	Processing Method	
Injection Volume:	10.00 ul	Channel Name:	
Run Time:	100.0 Minutes	Proc. Chnl. Descr.:	
Date Acquired:	01-12-2022 12:43:10 IST		
Date Processed:	05-01-2023 18:00:58 IST		

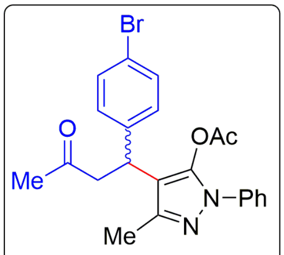

***rac*-3ba**

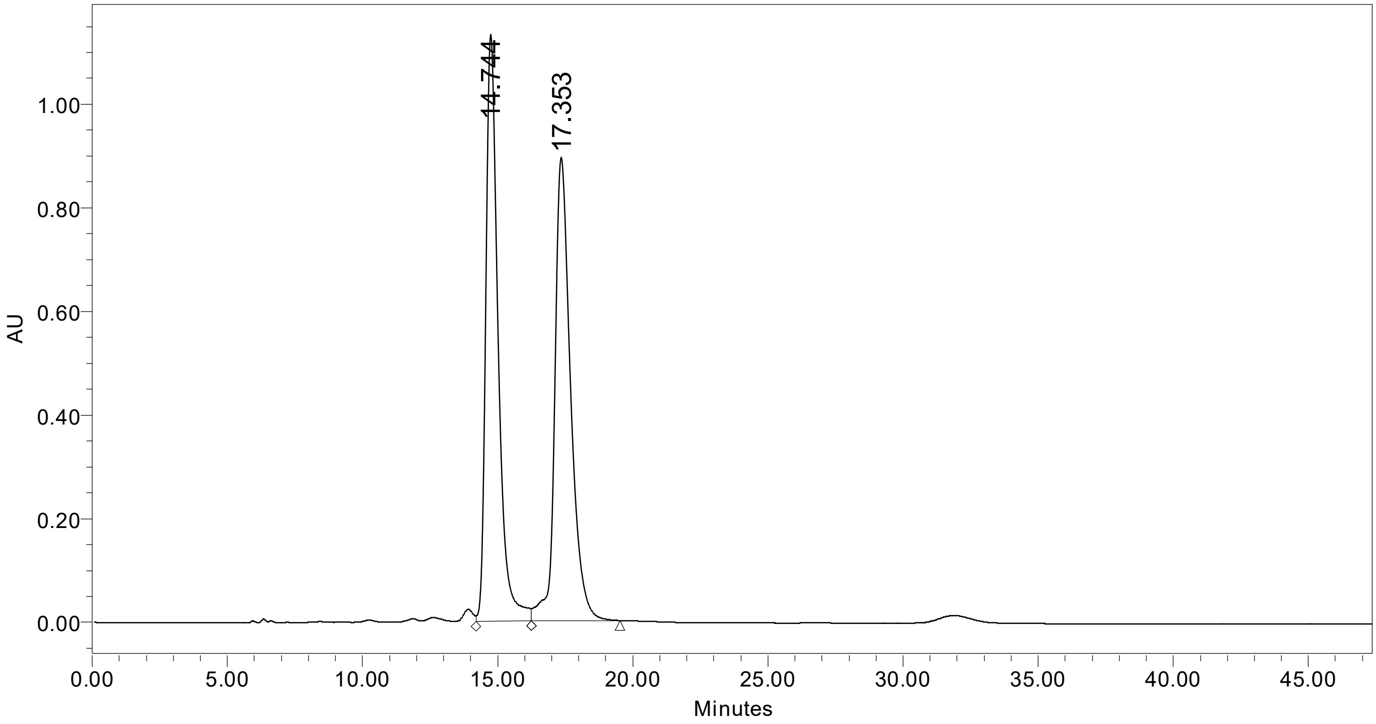

	RT	Area	% Area	Height
1	14.744	36044872	49.66	1133263
2	17.353	36536584	50.34	894938

SAMPLE INFORMATION

Sample Name:	PG44-OD-H	Acquired By:	
Sample Type:	Unknown	Sample Set Name:	
Vial:	1	Acq. Method Set:	
Injection #:	4	Processing Method	
Injection Volume:	5.00 ul	Channel Name:	
Run Time:	120.0 Minutes	Proc. Chnl. Descr.:	
Date Acquired:	28-10-2022 13:49:39 IST		
Date Processed:	28-10-2022 14:17:35 IST		

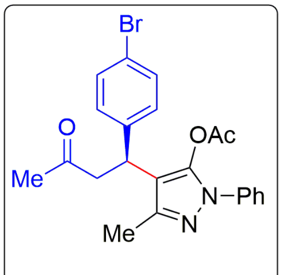

3ba

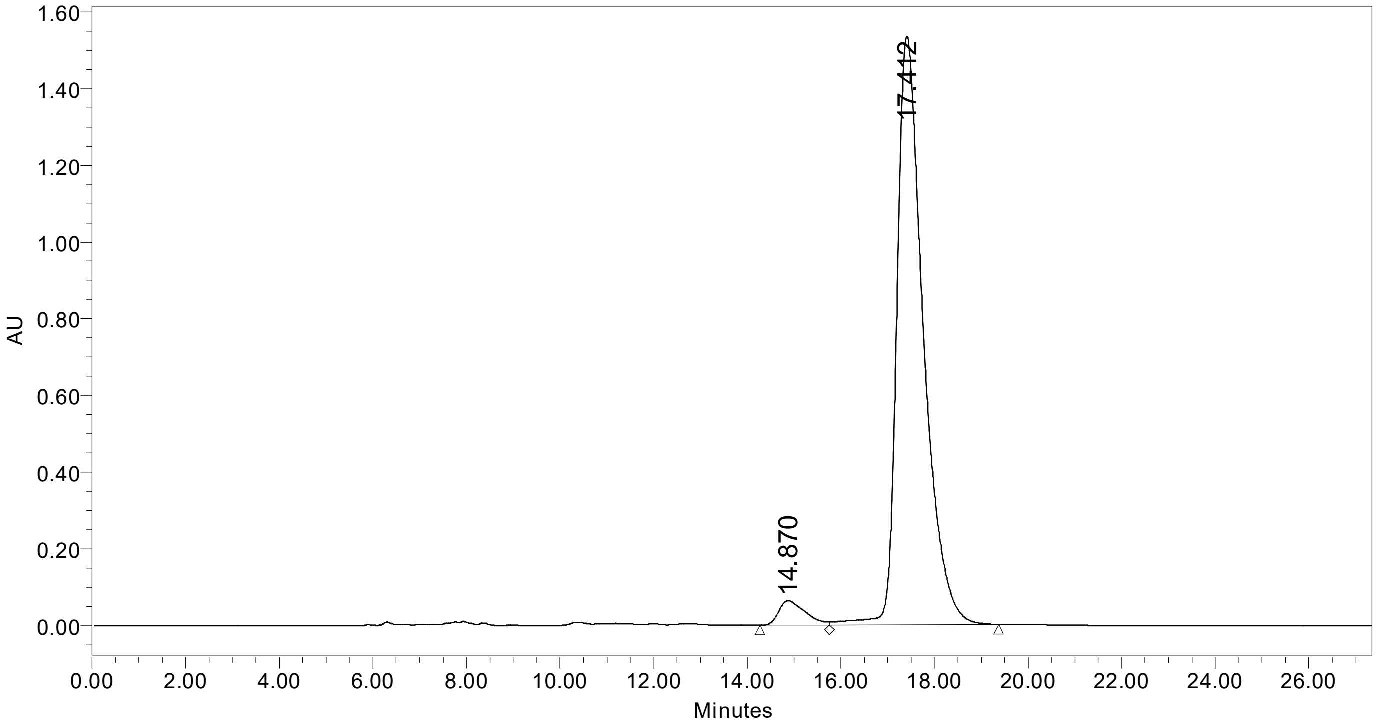

	RT	Area	% Area	Height
1	14.870	2490680	3.96	63884
2	17.412	60478795	96.04	1534578

SAMPLE INFORMATION

Sample Name:	PG44-CRYSTAL	Acquired By:	
Sample Type:	Unknown	Sample Set Name:	
Vial:	1	Acq. Method Set:	
Injection #:	2	Processing Method	
Injection Volume:	10.00 ul	Channel Name:	
Run Time:	100.0 Minutes	Proc. Chnl. Descr.:	
Date Acquired:	11-01-2023 13:15:15 IST		
Date Processed:	13-01-2023 11:30:28 IST		

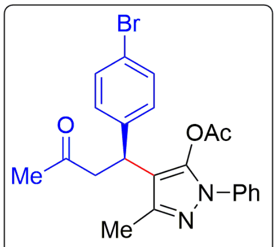

3ba

After single recrystallization

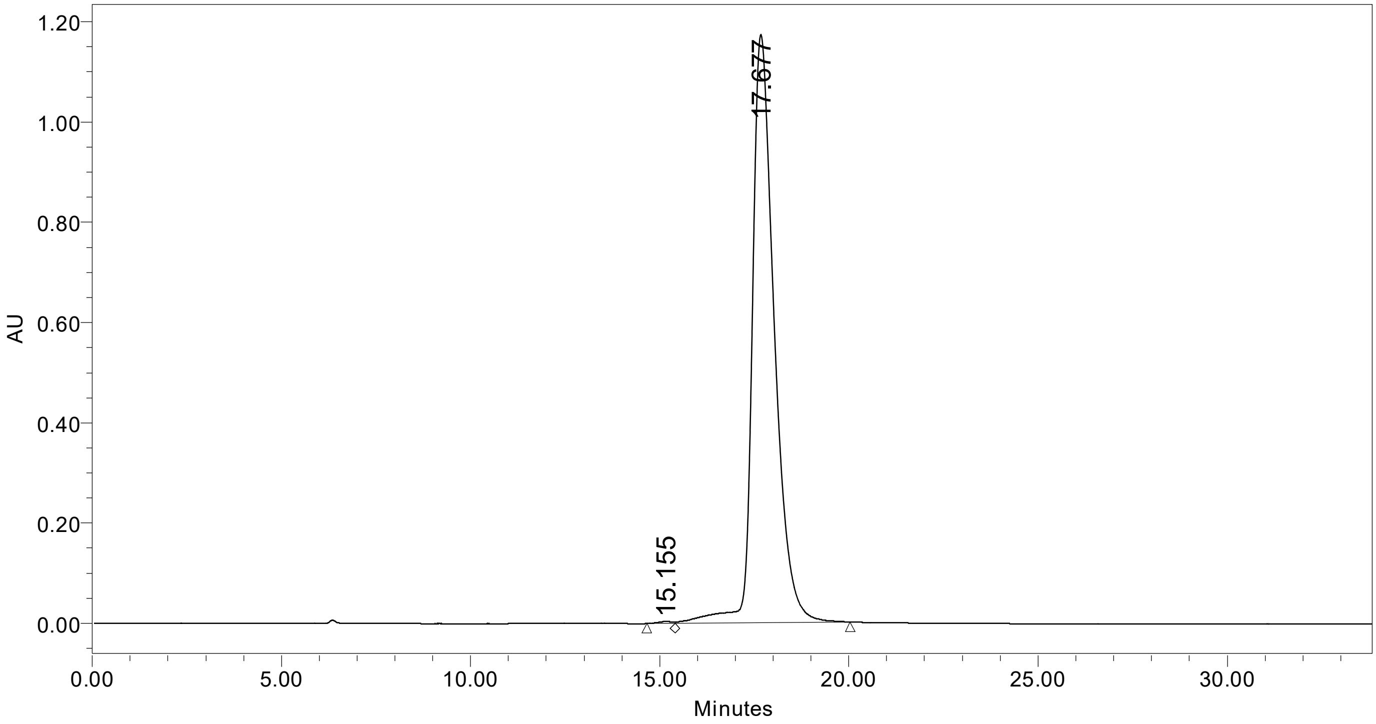

	RT	Area	% Area	Height
1	15.155	101420	0.21	3728
2	17.677	47603373	99.79	1173813

SAMPLE INFORMATION

Sample Name:	PG45-OD-H	Acquired By:	
Sample Type:	Unknown	Sample Set Name:	
Vial:	1	Acq. Method Set:	
Injection #:	6	Processing Method	
Injection Volume:	5.00 ul	Channel Name:	
Run Time:	120.0 Minutes	Proc. Chnl. Descr.:	
Date Acquired:	28-10-2022 14:46:48 IST		
Date Processed:	28-10-2022 15:24:33 IST		

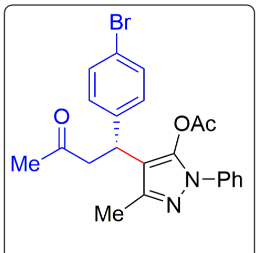

***ent*-3ba**

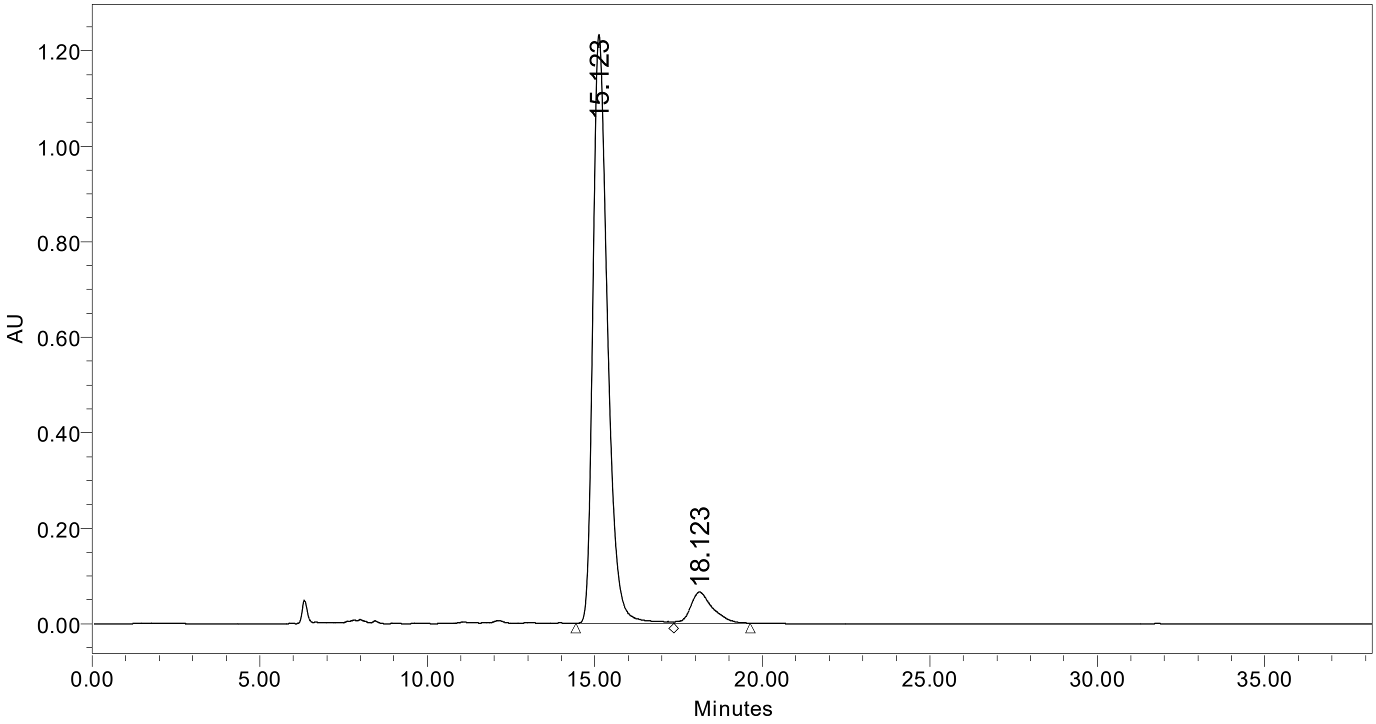

	RT	Area	% Area	Height
1	15.123	37490359	92.65	1233815
2	18.123	2975849	7.35	65127

SAMPLE INFORMATION

Sample Name:	PG92-ODH	Acquired By:	
Sample Type:	Unknown	Sample Set Name:	
Vial:	1	Acq. Method Set:	
Injection #:	3	Processing Method	
Injection Volume:	10.00 ul	Channel Name:	
Run Time:	100.0 Minutes	Proc. Chnl. Descr.:	
Date Acquired:	01-12-2022 11:52:30 IST		
Date Processed:	01-12-2022 18:05:32 IST		

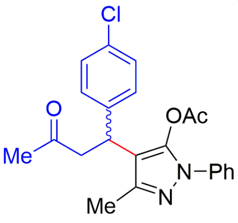

***rac*-3ca**

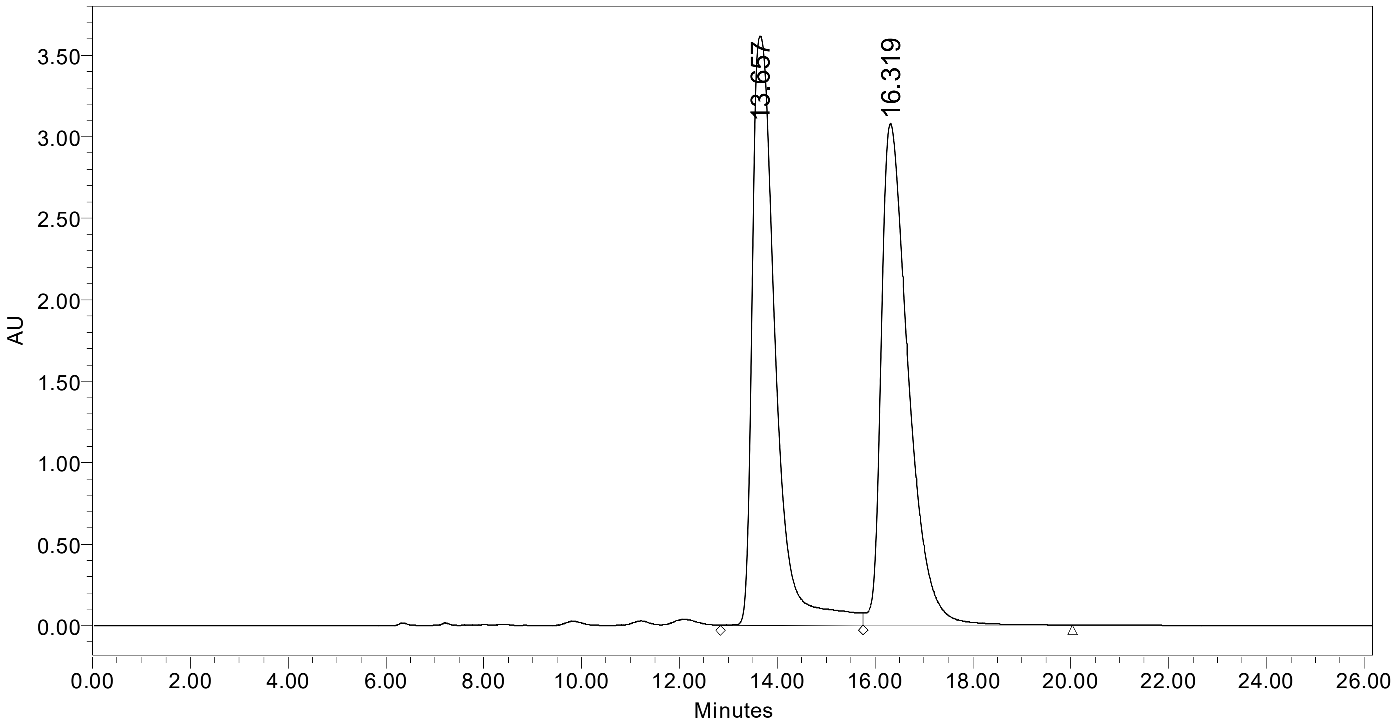

	RT	Area	% Area	Height
1	13.657	120011936	50.29	3616219
2	16.319	118644424	49.71	3078780

SAMPLE INFORMATION

Sample Name:	PG55-OD-H	Acquired By:	
Sample Type:	Unknown	Sample Set Name:	
Vial:	1	Acq. Method Set:	
Injection #:	3	Processing Method	
Injection Volume:	5.00 ul	Channel Name:	
Run Time:	120.0 Minutes	Proc. Chnl. Descr.:	
Date Acquired:	03-11-2022 14:39:20 IST		
Date Processed:	04-11-2022 15:06:54 IST		

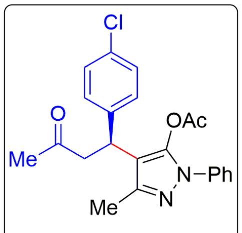

3ca

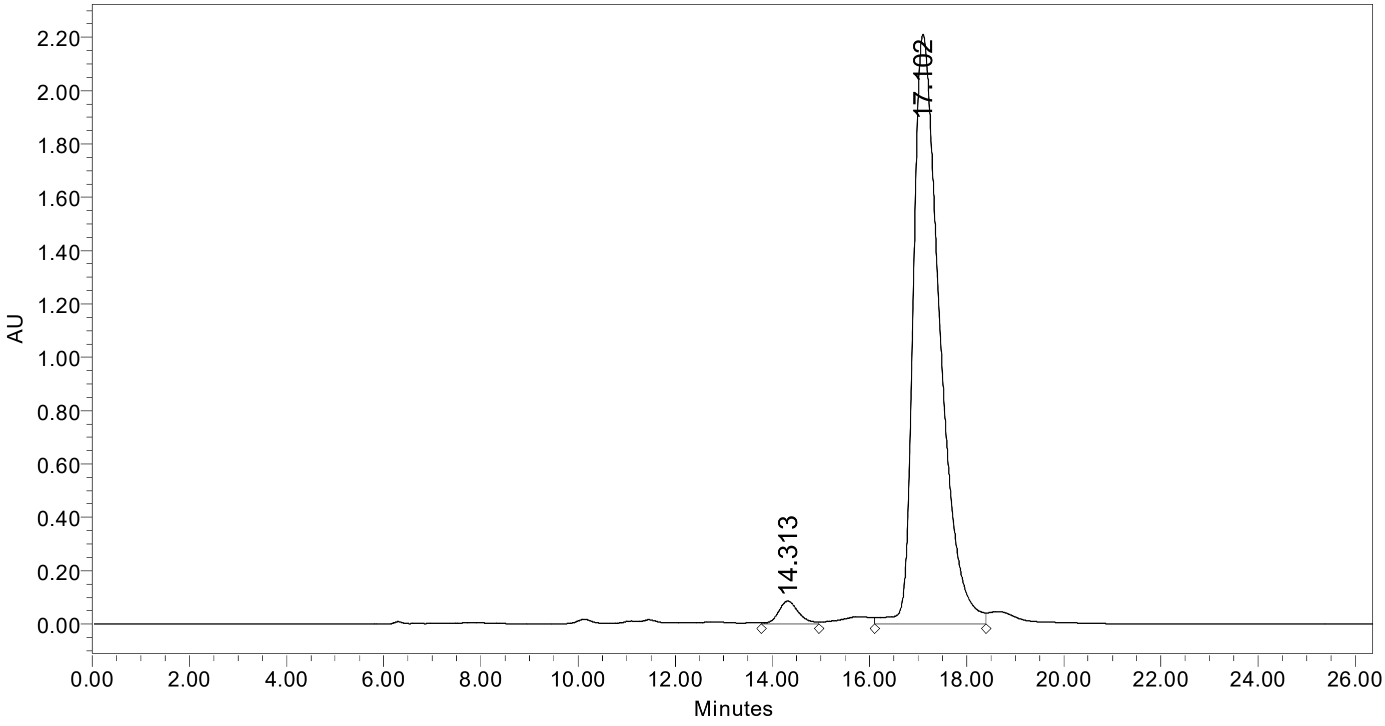

	RT	Area	% Area	Height
1	14.313	2432605	2.83	85500
2	17.102	83518750	97.17	2209887

SAMPLE INFORMATION

Sample Name:	PG56-OD-H	Acquired By:	
Sample Type:	Unknown	Sample Set Name:	
Vial:	1	Acq. Method Set:	
Injection #:	4	Processing Method	
Injection Volume:	5.00 ul	Channel Name:	
Run Time:	120.0 Minutes	Proc. Chnl. Descr.:	
Date Acquired:	03-11-2022 15:27:23 IST		
Date Processed:	11-11-2022 14:41:53 IST		

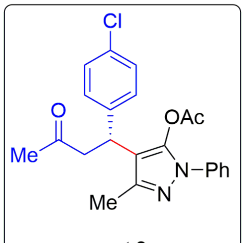

***ent*-3ca**

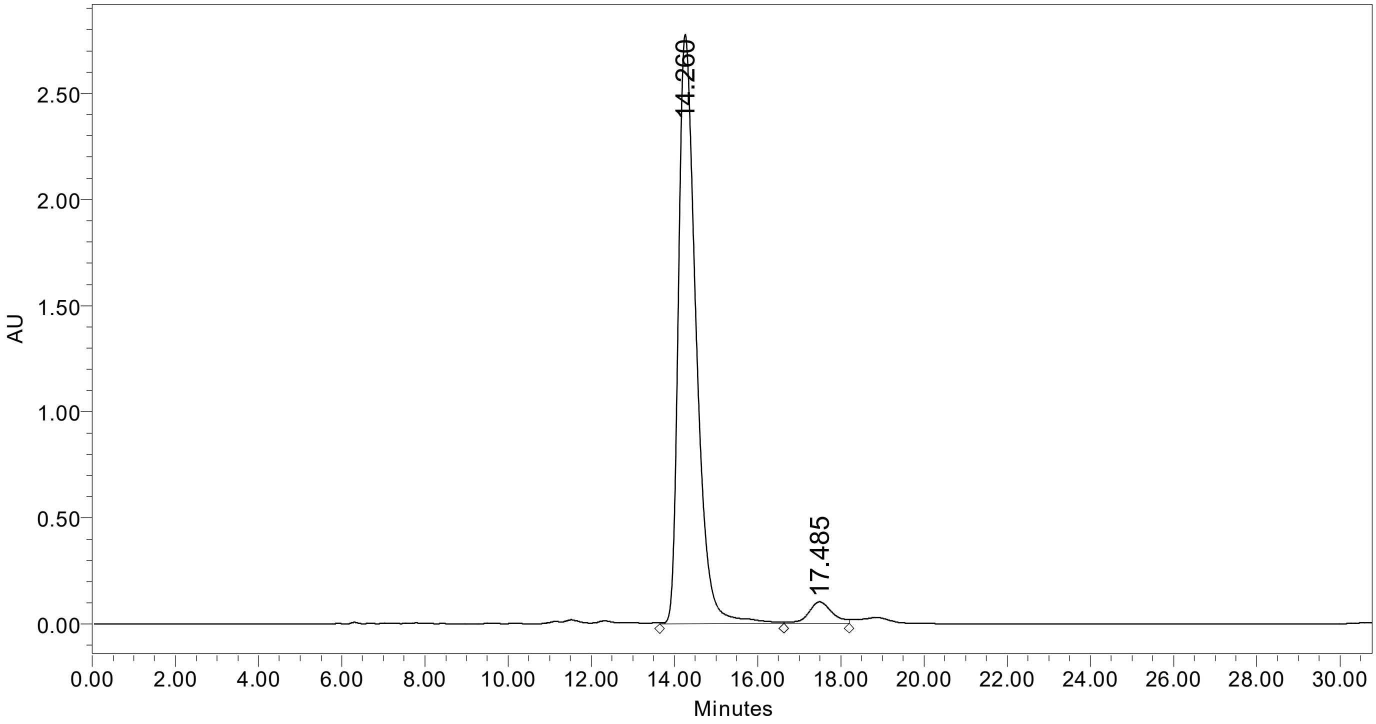

	RT	Area	% Area	Height
1	14.260	82147153	95.26	2776418
2	17.485	4088503	4.74	102285

SAMPLE INFORMATION

Sample Name:	PG100-ODH	Acquired By:	
Sample Type:	Unknown	Sample Set Name:	
Vial:	1	Acq. Method Set:	
Injection #:	5	Processing Method	
Injection Volume:	10.00 ul	Channel Name:	
Run Time:	100.0 Minutes	Proc. Chnl. Descr.:	
Date Acquired:	28-12-2022 12:39:48 IST		
Date Processed:	28-12-2022 17:30:07 IST		

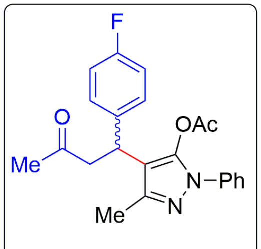

***rac*-3da**

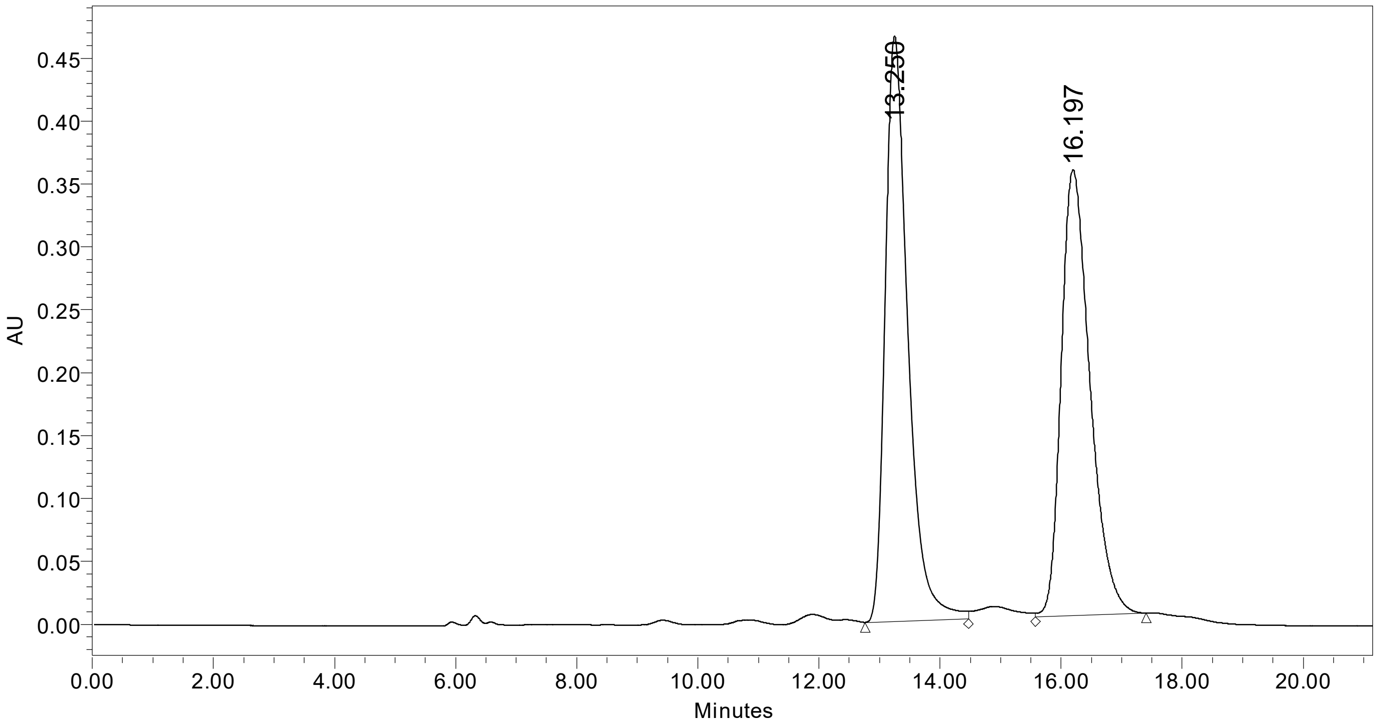

	RT	Area	% Area	Height
1	13.250	12177405	51.01	465531
2	16.197	11696466	48.99	354300

SAMPLE INFORMATION

Sample Name:	PG155	Acquired By:	
Sample Type:	Unknown	Sample Set Name:	
Vial:	1	Acq. Method Set:	
Injection #:	3	Processing Method	
Injection Volume:	10.00 ul	Channel Name:	
Run Time:	100.0 Minutes	Proc. Chnl. Descr.:	
Date Acquired:	08-02-2023 14:49:25 IST		
Date Processed:	21-03-2023 19:38:44 IST		

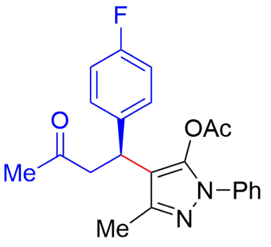

3da

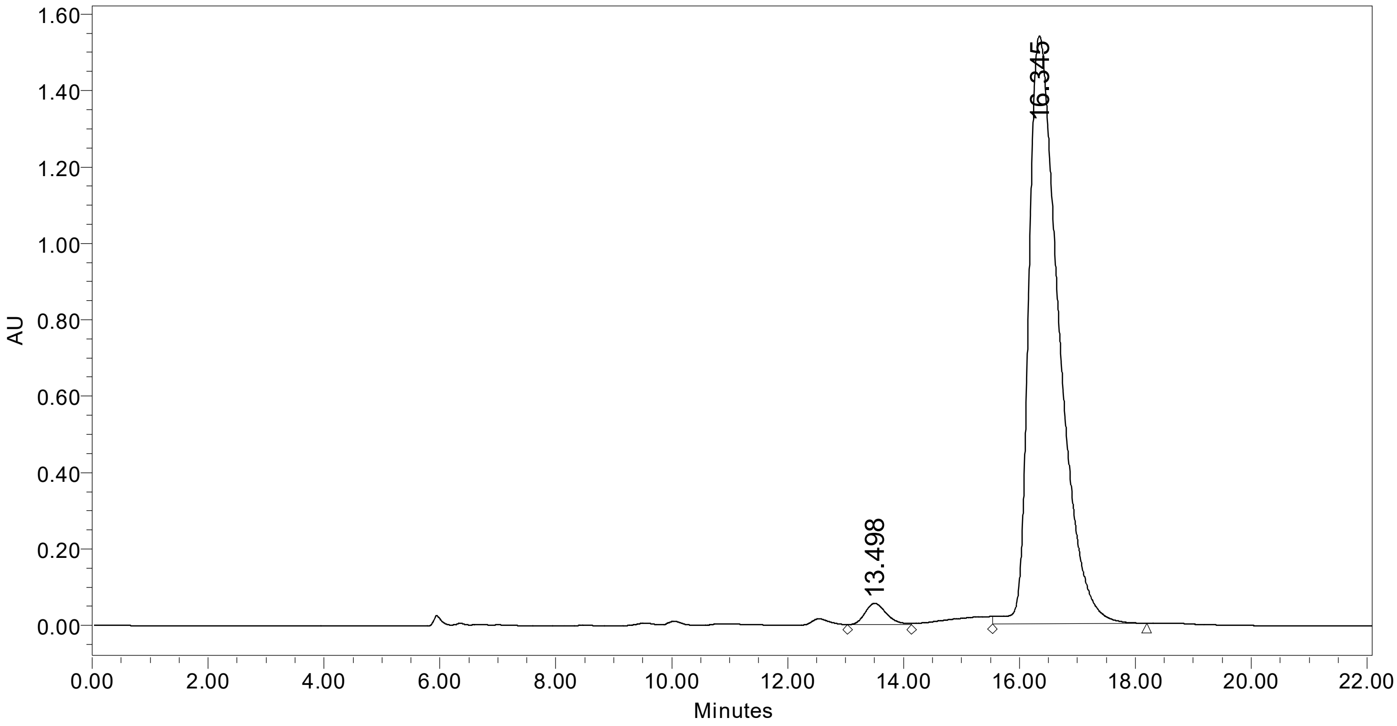

	RT	Area	% Area	Height
1	13.498	1459269	2.56	55988
2	16.345	55510958	97.44	1539264

RC2638PDF

SAMPLE INFORMATION

Sample Name:	PG156	Acquired By:	
Sample Type:	Unknown	Sample Set Name:	
Vial:	1	Acq. Method Set:	
Injection #:	6	Processing Method	
Injection Volume:	10.00 ul	Channel Name:	
Run Time:	100.0 Minutes	Proc. Chnl. Descr.:	
Date Acquired:	08-02-2023 15:54:27 IST		
Date Processed:	16-03-2023 18:56:50 IST		

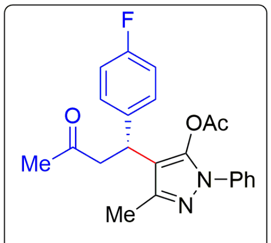

***ent*-3da**

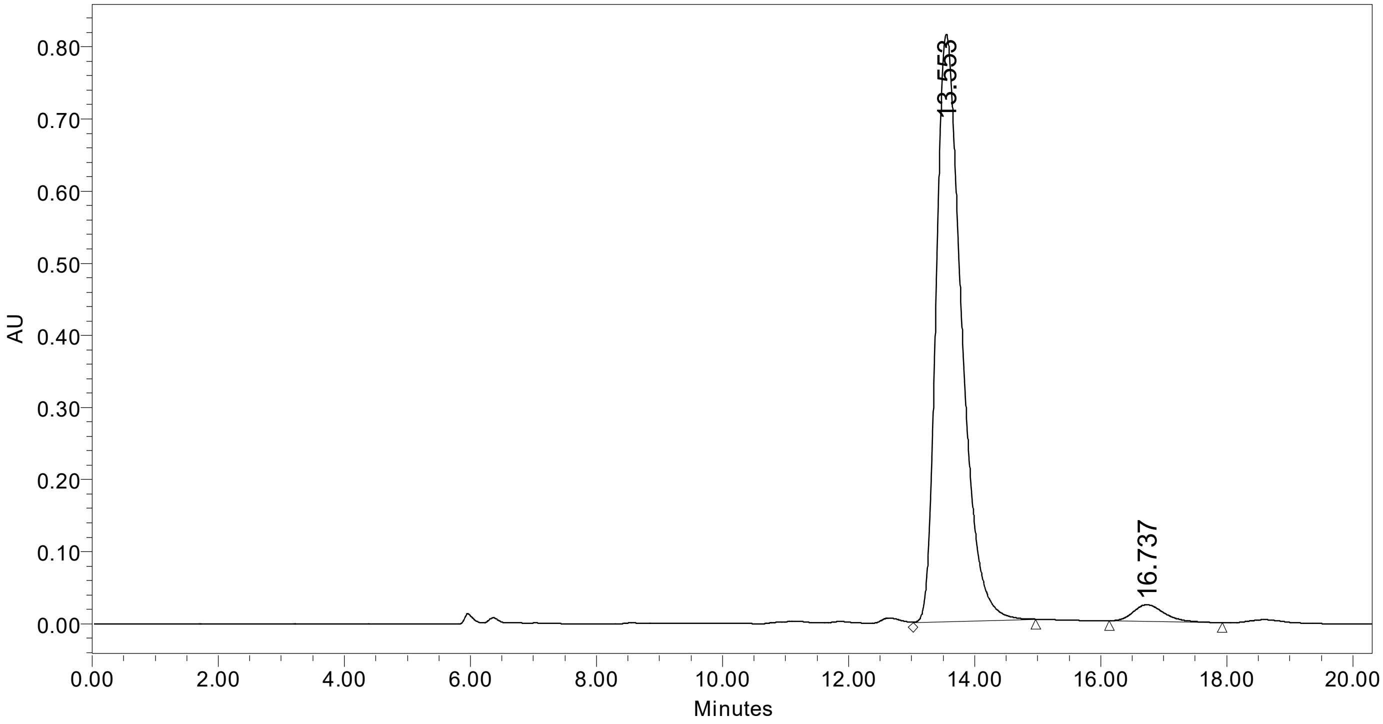

	RT	Area	% Area	Height
1	13.553	22763661	96.71	814289
2	16.737	775139	3.29	23020

SAMPLE INFORMATION

Sample Name:	PG99-ODH	Acquired By:	
Sample Type:	Unknown	Sample Set Name:	
Vial:	1	Acq. Method Set:	
Injection #:	7	Processing Method	
Injection Volume:	10.00 ul	Channel Name:	
Run Time:	100.0 Minutes	Proc. Chnl. Descr.:	
Date Acquired:	28-12-2022 13:46:33 IST		
Date Processed:	28-12-2022 17:29:14 IST		

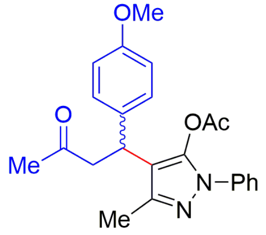

***rac*-3ea**

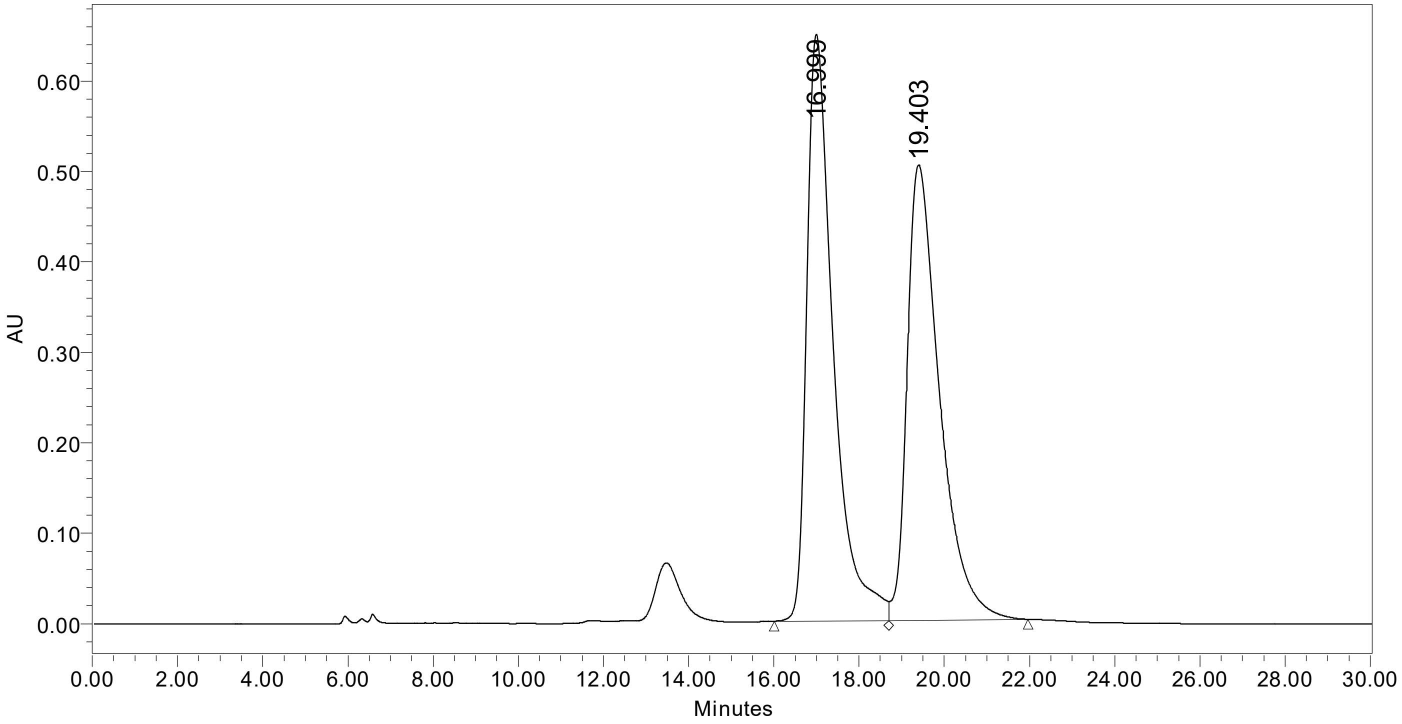

	RT	Area	% Area	Height
1	16.999	27917751	51.15	648798
2	19.403	26664037	48.85	503707

SAMPLE INFORMATION

Sample Name:	PG157	Acquired By:	
Sample Type:	Unknown	Sample Set Name:	
Vial:	1	Acq. Method Set:	
Injection #:	7	Processing Method	
Injection Volume:	10.00 ul	Channel Name:	
Run Time:	100.0 Minutes	Proc. Chnl. Descr.:	
Date Acquired:	09-02-2023 15:44:12 IST		
Date Processed:	09-02-2023 17:17:54 IST		

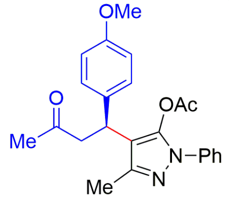

3ea

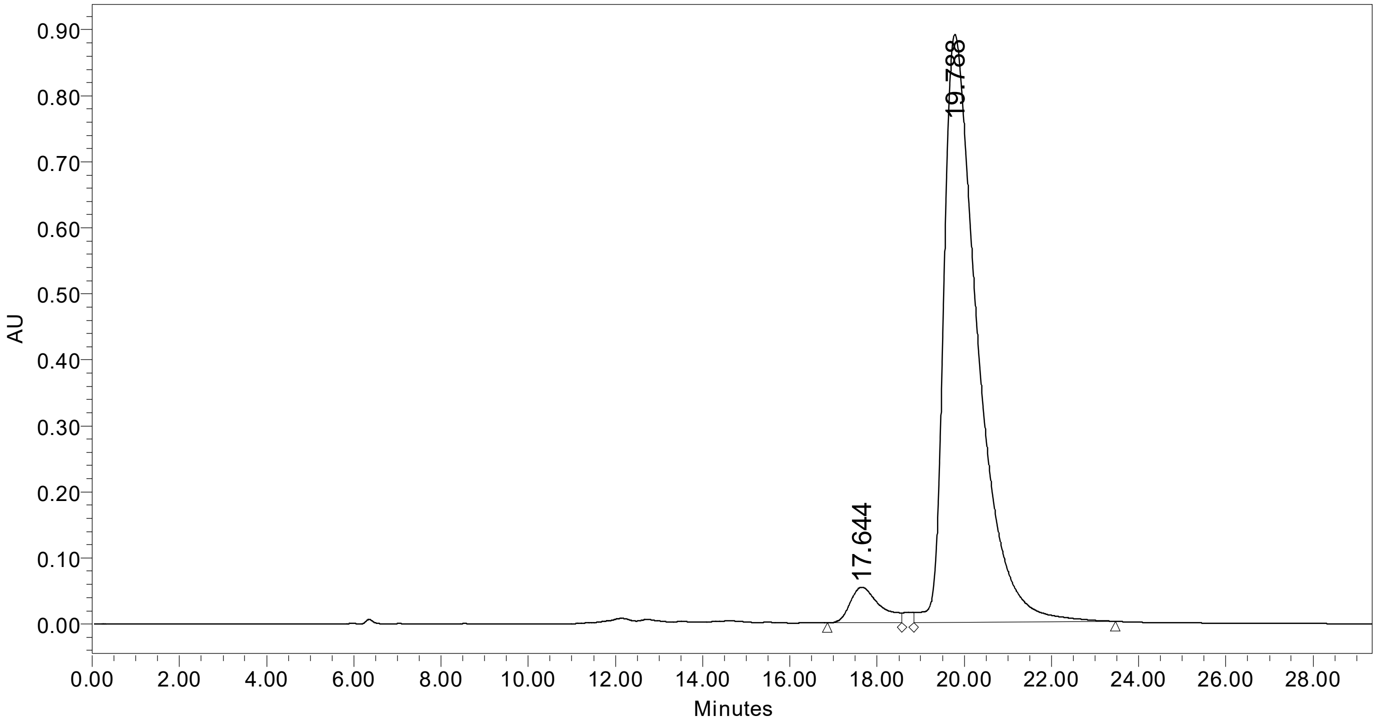

	RT	Area	% Area	Height
1	17.644	2542718	5.04	53729
2	19.788	47892578	94.96	890381

SAMPLE INFORMATION

Sample Name:	PG89-ODH	Acquired By:	
Sample Type:	Unknown	Sample Set Name:	
Vial:	1	Acq. Method Set:	
Injection #:	8	Processing Method	
Injection Volume:	10.00 ul	Channel Name:	
Run Time:	100.0 Minutes	Proc. Chnl. Descr.:	
Date Acquired:	30-11-2022 13:22:55 IST		
Date Processed:	30-11-2022 14:04:46 IST		

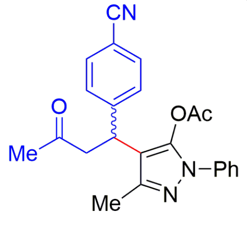

***rac*-3fa**

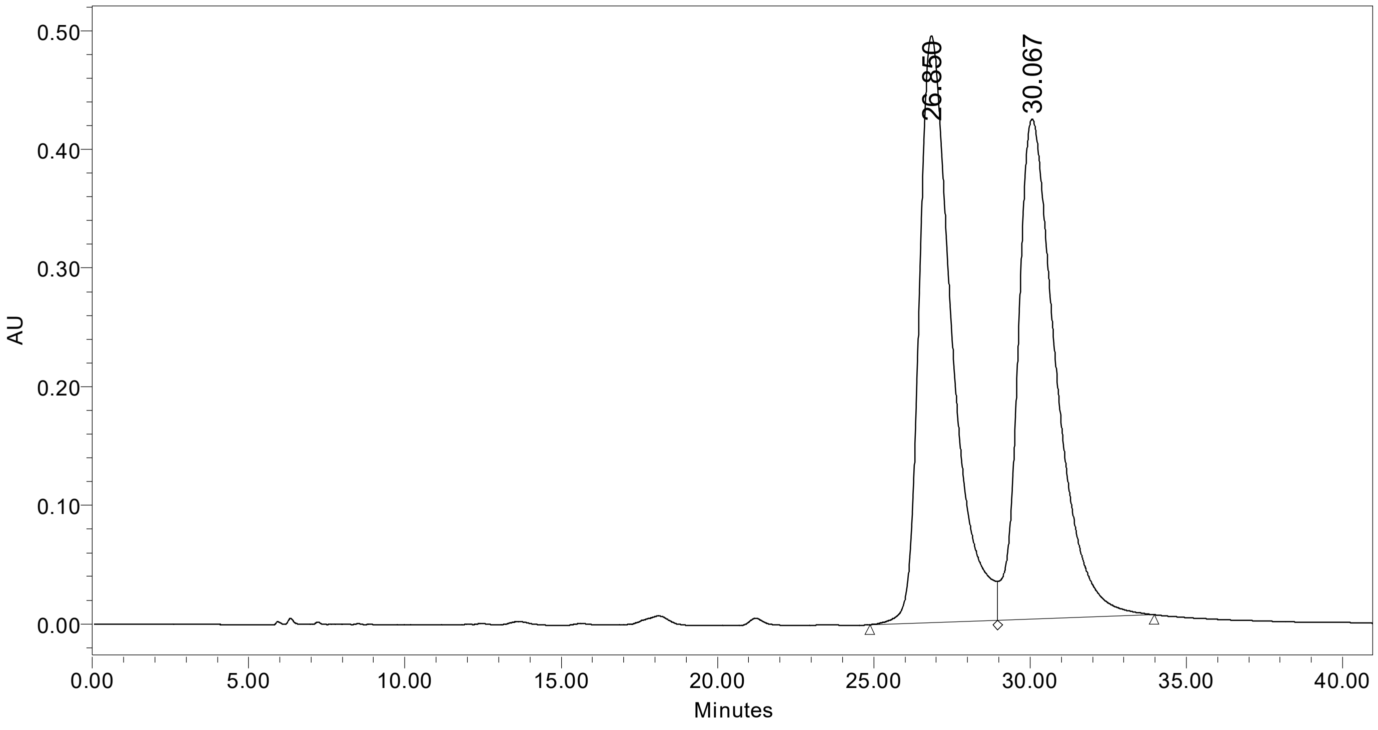

	RT	Area	% Area	Height
1	26.850	35950030	50.24	494515
2	30.067	35599754	49.76	421418

SAMPLE INFORMATION

Sample Name:	PG85-ODH	Acquired By:	
Sample Type:	Unknown	Sample Set Name:	
Vial:	1	Acq. Method Set:	
Injection #:	6	Processing Method	
Injection Volume:	10.00 ul	Channel Name:	
Run Time:	100.0 Minutes	Proc. Chnl. Descr.:	
Date Acquired:	30-11-2022 11:50:54 IST		
Date Processed:	30-11-2022 13:31:53 IST		

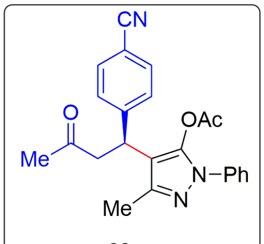

3fa

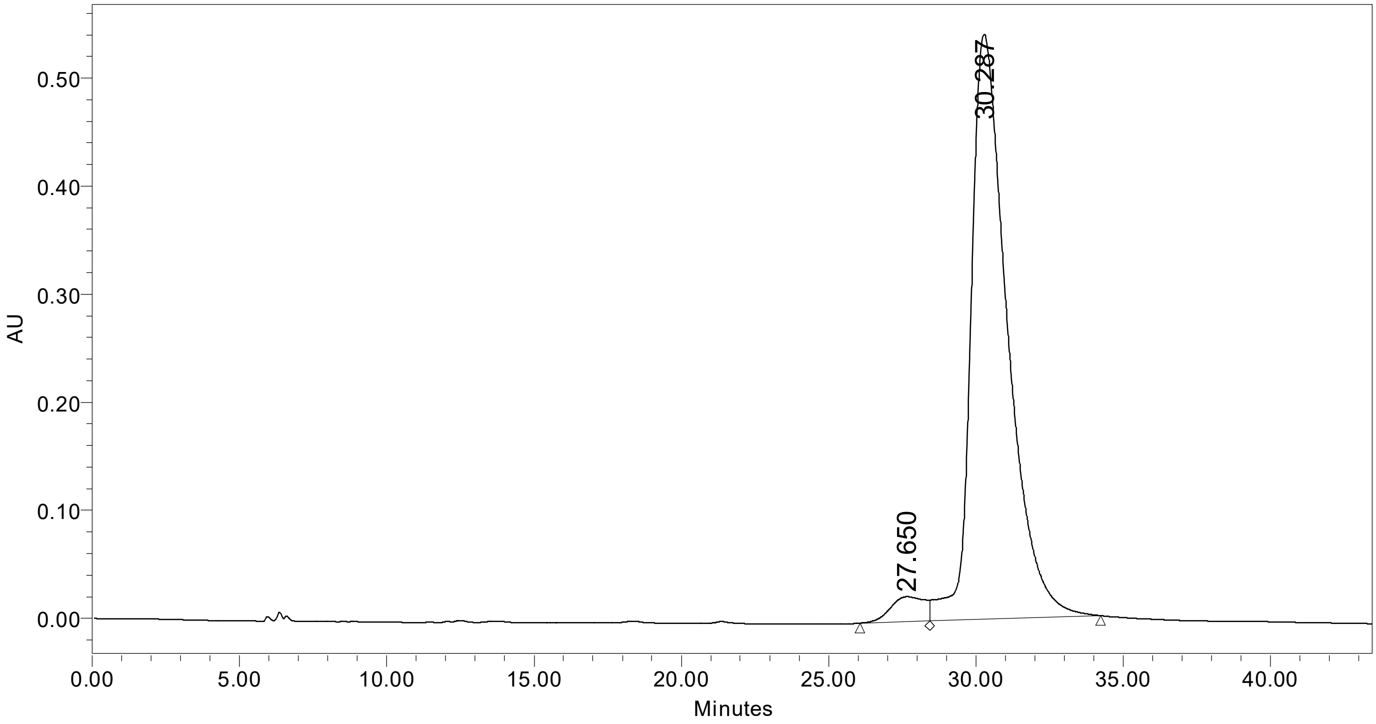

	RT	Area	% Area	Height
1	27.650	1887183	3.89	23163
2	30.287	46649969	96.11	541444

SAMPLE INFORMATION

Sample Name:	PG86-ODH	Acquired By:	
Sample Type:	Unknown	Sample Set Name:	
Vial:	1	Acq. Method Set:	
Injection #:	7	Processing Method	
Injection Volume:	10.00 ul	Channel Name:	
Run Time:	100.0 Minutes	Proc. Chnl. Descr.:	
Date Acquired:	30-11-2022 12:34:42 IST		
Date Processed:	30-11-2022 13:33:17 IST		

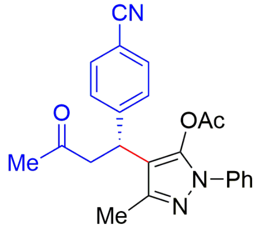

***ent*-3fa**

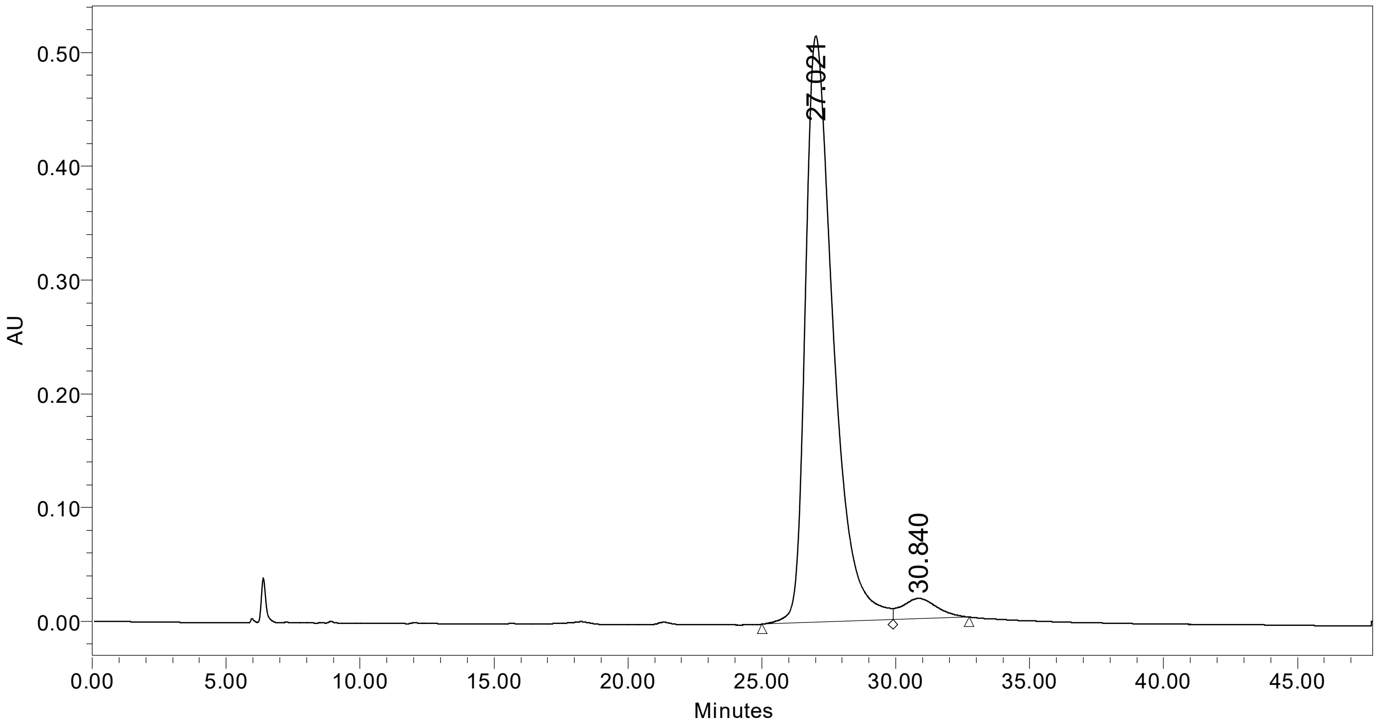

	RT	Area	% Area	Height
1	27.021	37463824	95.76	515567
2	30.840	1657709	4.24	17925

SAMPLE INFORMATION

Sample Name:	PG67-68-OJH	Acquired By:	
Sample Type:	Unknown	Sample Set Name:	
Vial:	1	Acq. Method Set:	
Injection #:	6	Processing Method	
Injection Volume:	10.00 ul	Channel Name:	
Run Time:	100.0 Minutes	Proc. Chnl. Descr.:	
Date Acquired:	17-11-2022 18:05:22 IST		
Date Processed:	21-11-2022 15:47:30 IST		

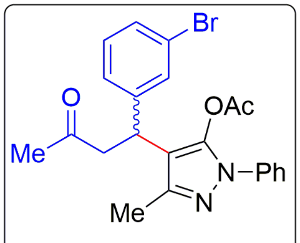

***rac*-3ga**

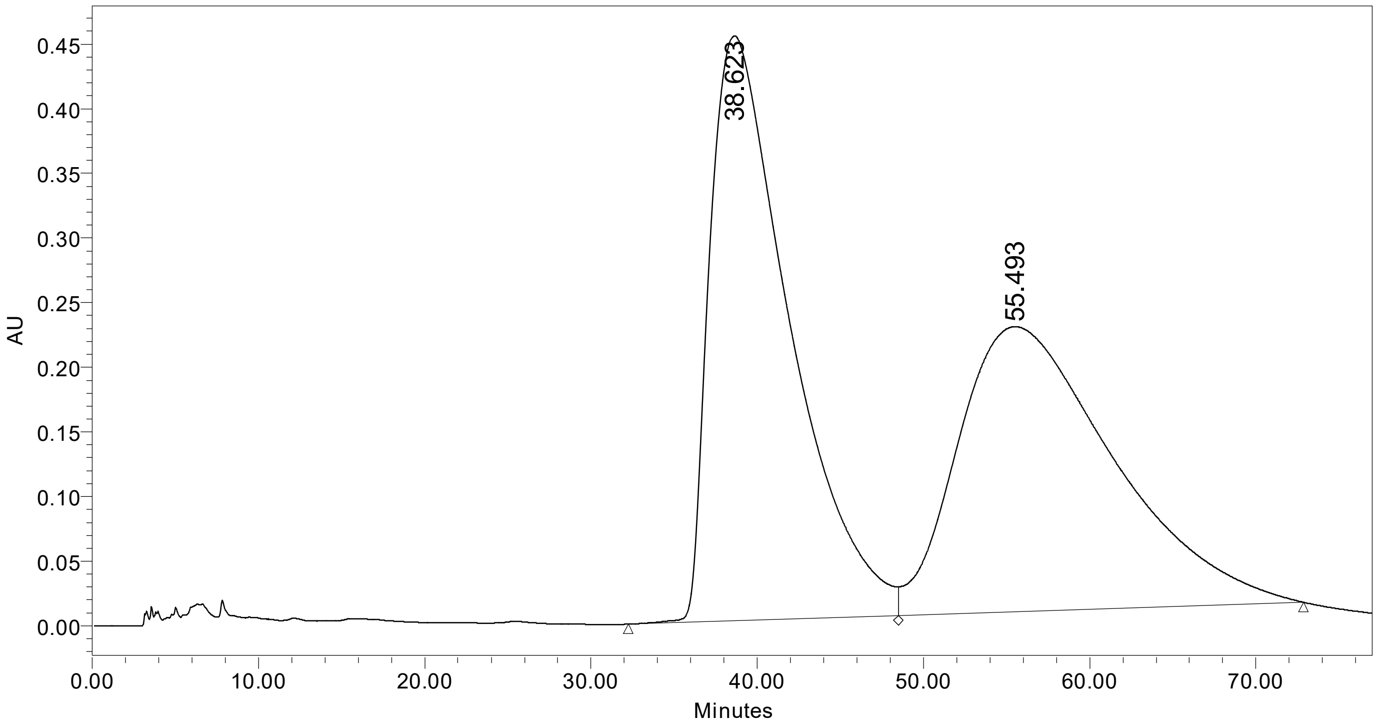

	RT	Area	% Area	Height
1	38.623	151231394	51.37	452384
2	55.493	143190385	48.63	220389

SAMPLE INFORMATION

Sample Name:	PG67-OJH	Acquired By:	System
Sample Type:	Unknown	Sample Set Name:	
Vial:	1	Acq. Method Set:	
Injection #:	3	Processing Method	
Injection Volume:	10.00 ul	Channel Name:	
Run Time:	100.0 Minutes	Proc. Chnl. Descr.:	
Date Acquired:	18-11-2022 14:06:59 IST		
Date Processed:	21-11-2022 15:51:08 IST		

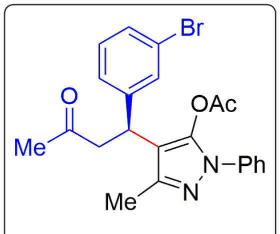

3ga

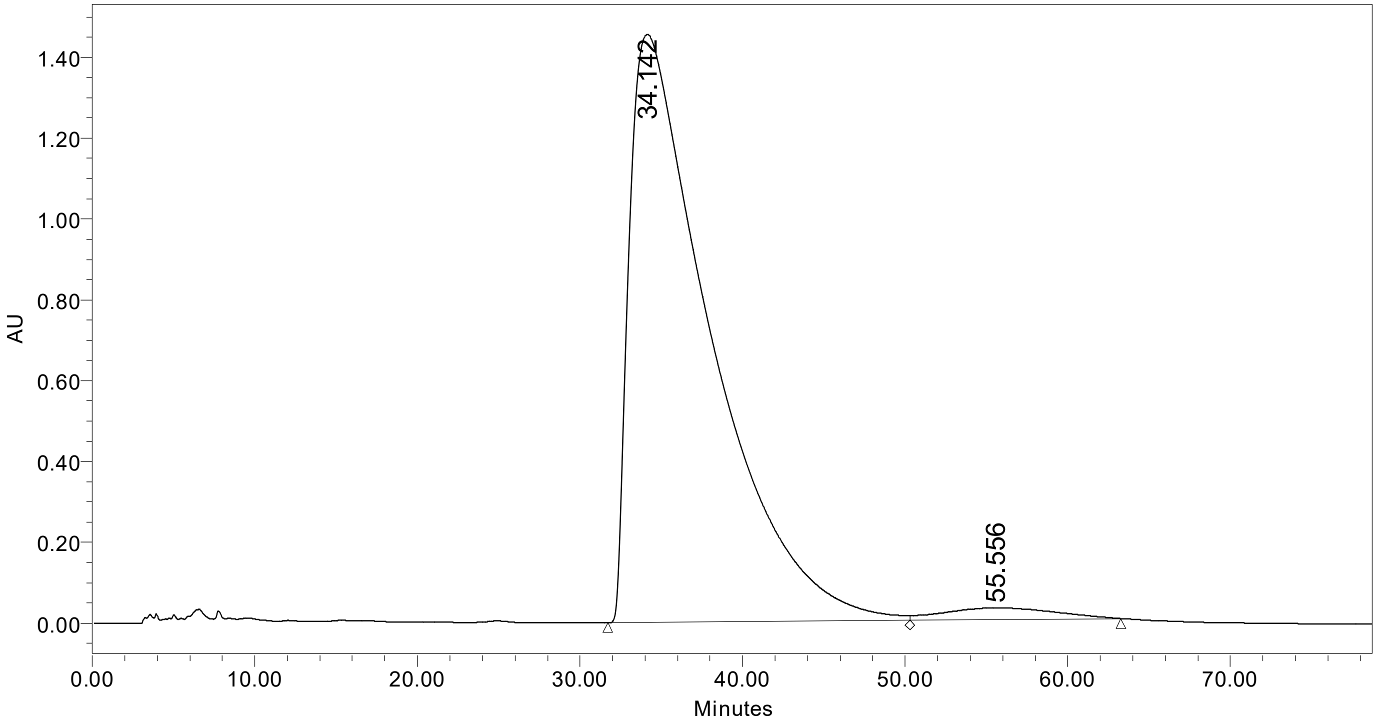

	RT	Area	% Area	Height
1	34.142	507143983	97.34	1455265
2	55.556	13840728	2.66	28942

SAMPLE INFORMATION

Sample Name:	PG68-OJH	Acquired By:	
Sample Type:	Unknown	Sample Set Name:	
Vial:	1	Acq. Method Set:	
Injection #:	2	Processing Method	
Injection Volume:	10.00 ul	Channel Name:	
Run Time:	100.0 Minutes	Proc. Chnl. Descr.:	
Date Acquired:	18-11-2022 12:35:39 IST		
Date Processed:	21-11-2022 15:52:14 IST		

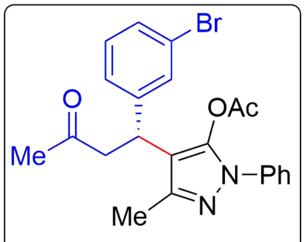

***ent*-3ga**

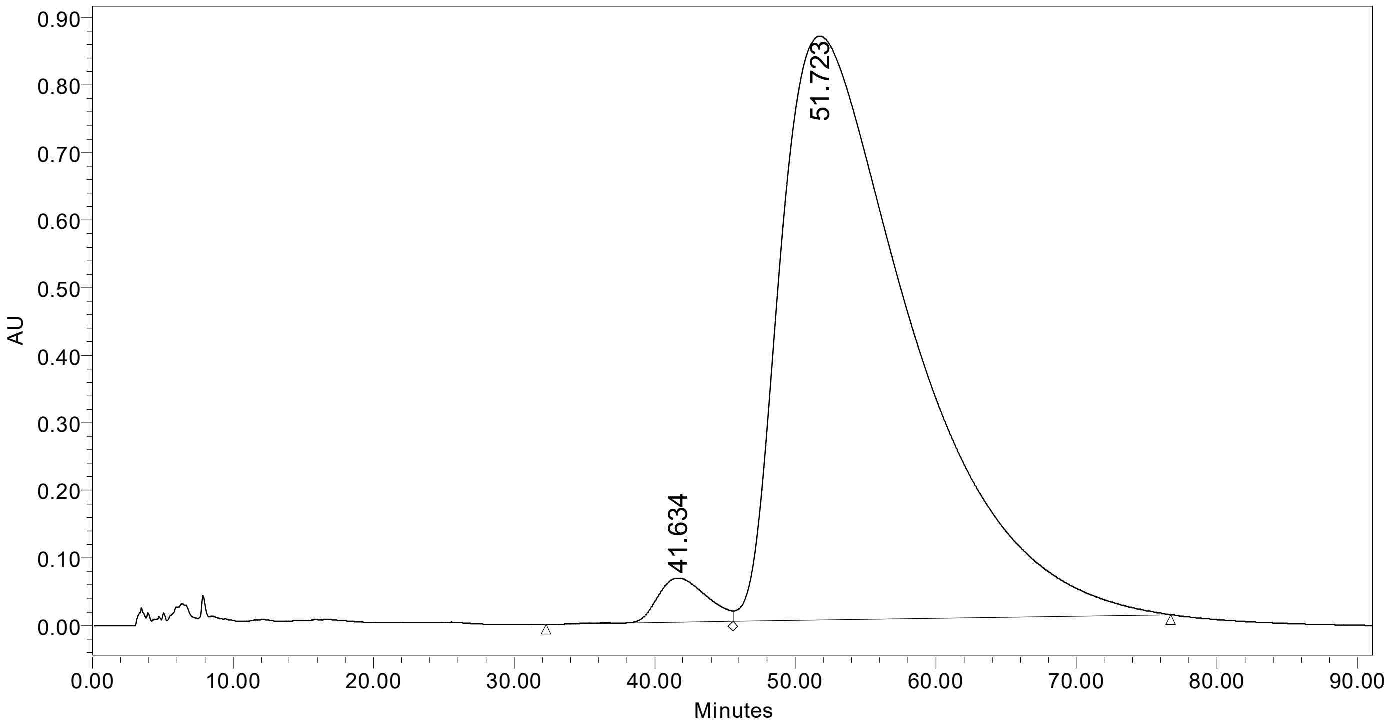

	RT	Area	% Area	Height
1	41.634	16058557	2.77	65761
2	51.723	563389567	97.23	864614

SAMPLE INFORMATION

Sample Name:	PG69-70-OJH	Acquired By:	
Sample Type:	Unknown	Sample Set Name:	
Vial:	1	Acq. Method Set:	
Injection #:	3	Processing Method	
Injection Volume:	10.00 ul	Channel Name:	
Run Time:	100.0 Minutes	Proc. Chnl. Descr.:	
Date Acquired:	17-11-2022 14:02:02 IST		
Date Processed:	21-11-2022 15:53:54 IST		

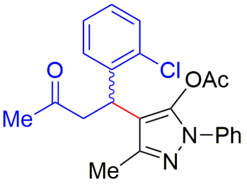

rac-3ha

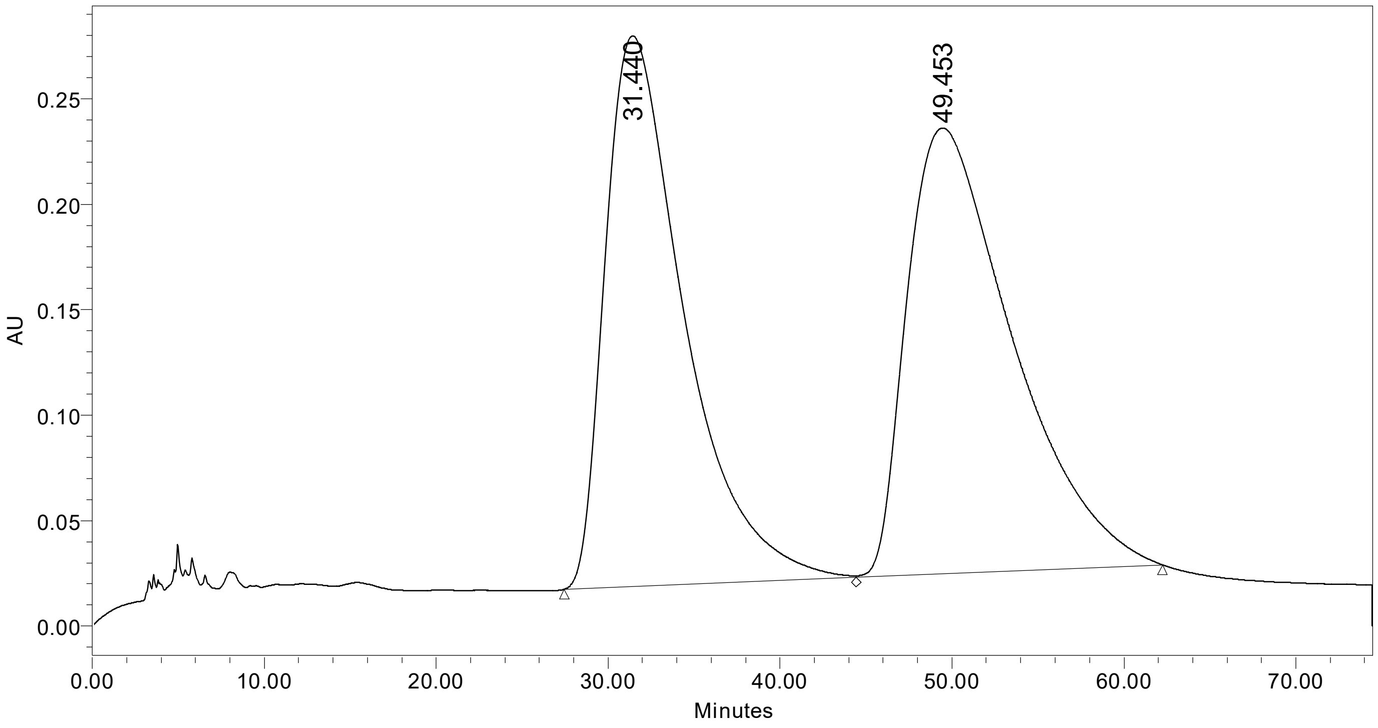

	RT	Area	% Area	Height
1	31.440	83613550	48.08	261211
2	49.453	90276211	51.92	211459

SAMPLE INFORMATION

Sample Name:	PG69-OJH	Acquired By:	
Sample Type:	Unknown	Sample Set Name:	
Vial:	1	Acq. Method Set:	
Injection #:	4	Processing Method	
Injection Volume:	10.00 ul	Channel Name:	
Run Time:	100.0 Minutes	Proc. Chnl. Descr.:	
Date Acquired:	17-11-2022 15:16:54 IST		
Date Processed:	17-11-2022 17:58:24 IST		

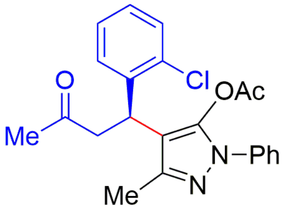

3ha

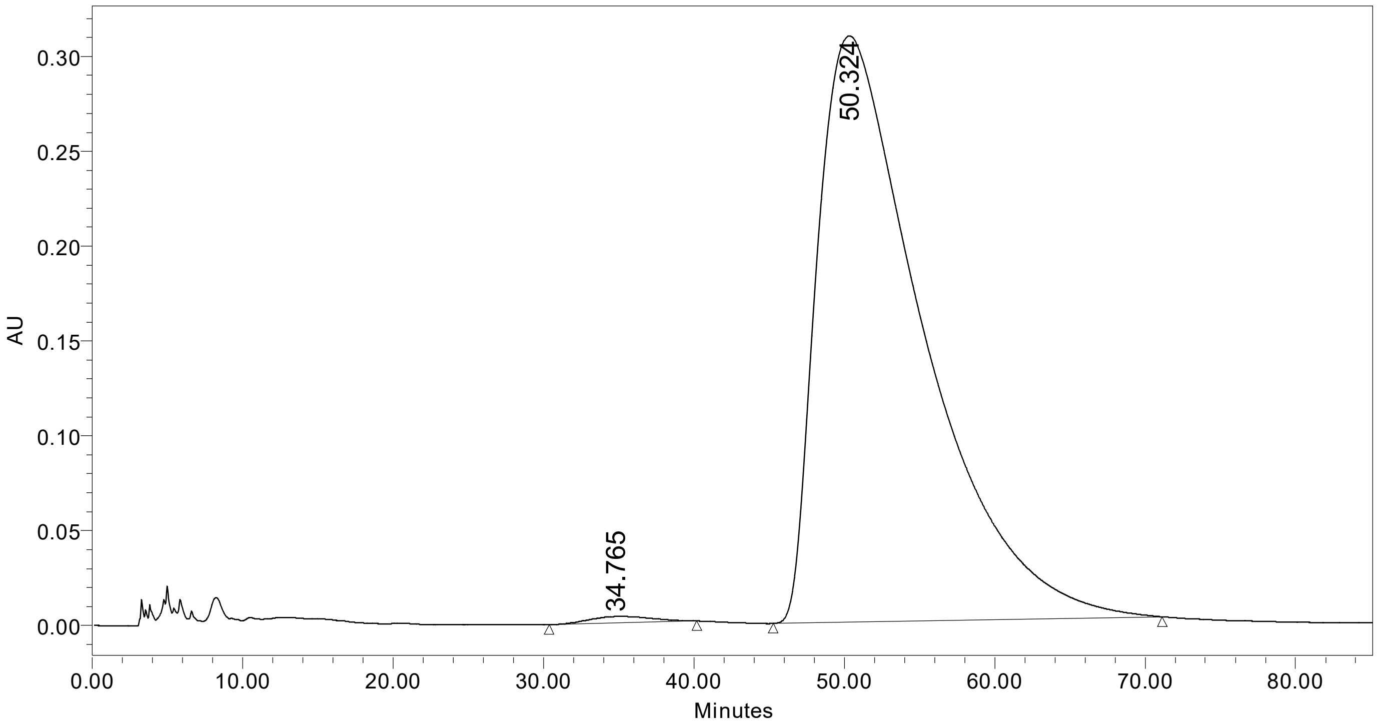

	RT	Area	% Area	Height
1	34.765	996392	0.65	3484
2	50.324	151760560	99.35	309184

SAMPLE INFORMATION

Sample Name:	PG70-OJH	Acquired By:	
Sample Type:	Unknown	Sample Set Name:	
Vial:	1	Acq. Method Set:	
Injection #:	5	Processing Method	
Injection Volume:	10.00 ul	Channel Name:	
Run Time:	100.0 Minutes	Proc. Chnl. Descr.:	
Date Acquired:	17-11-2022 16:43:43 IST		
Date Processed:	05-01-2023 18:07:35 IST		

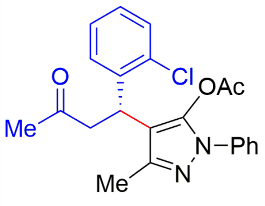

***ent*-3ha**

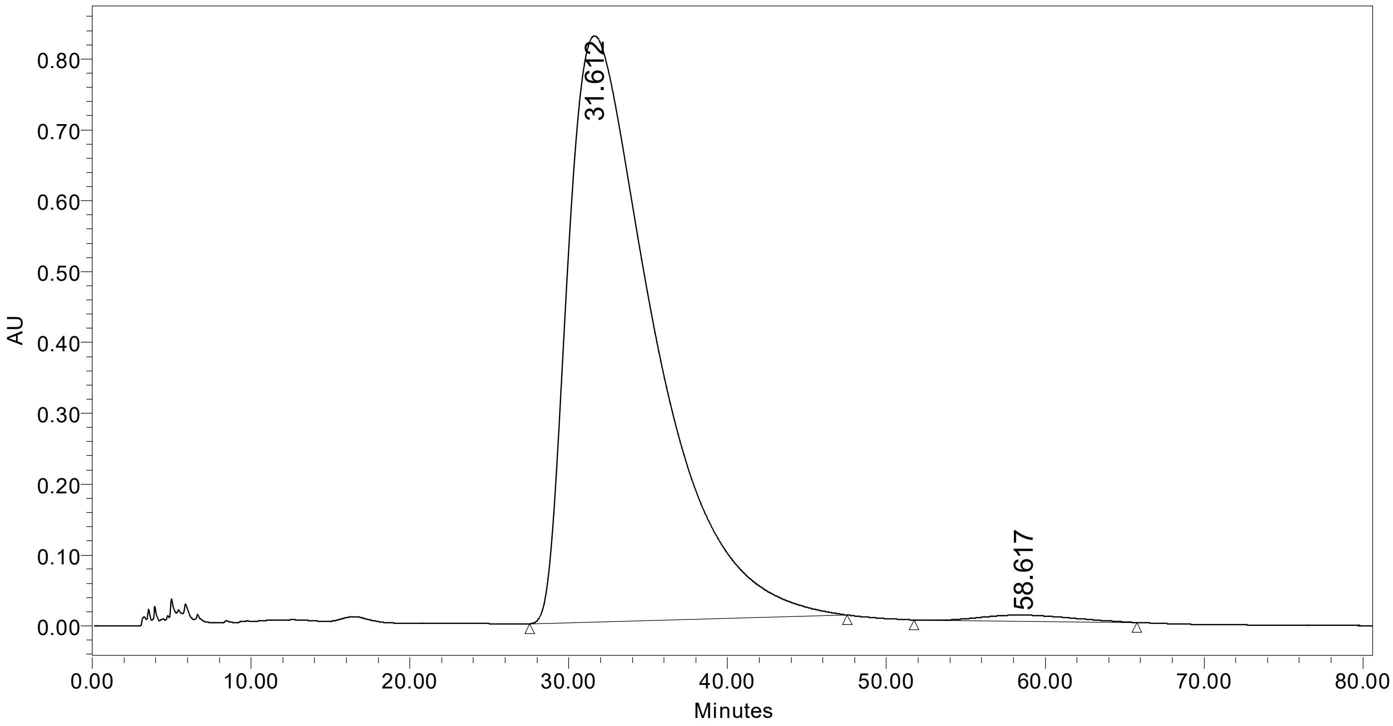

	RT	Area	% Area	Height
1	31.612	313265985	98.93	827224
2	58.617	3376038	1.07	8527

S A M P L E I N F O R M A T I O N

Sample Name:	PG94-ODH	Acquired By:	
Sample Type:	Unknown	Sample Set Name:	
Vial:	1	Acq. Method Set:	
Injection #:	6	Processing Method	
Injection Volume:	10.00 ul	Channel Name:	
Run Time:	100.0 Minutes	Proc. Chnl. Descr.:	
Date Acquired:	01-12-2022 13:30:50 IST		
Date Processed:	05-01-2023 18:11:25 IST		

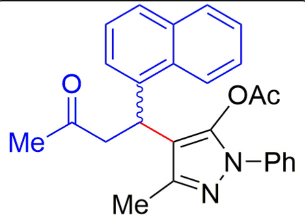

***rac*-3ia**

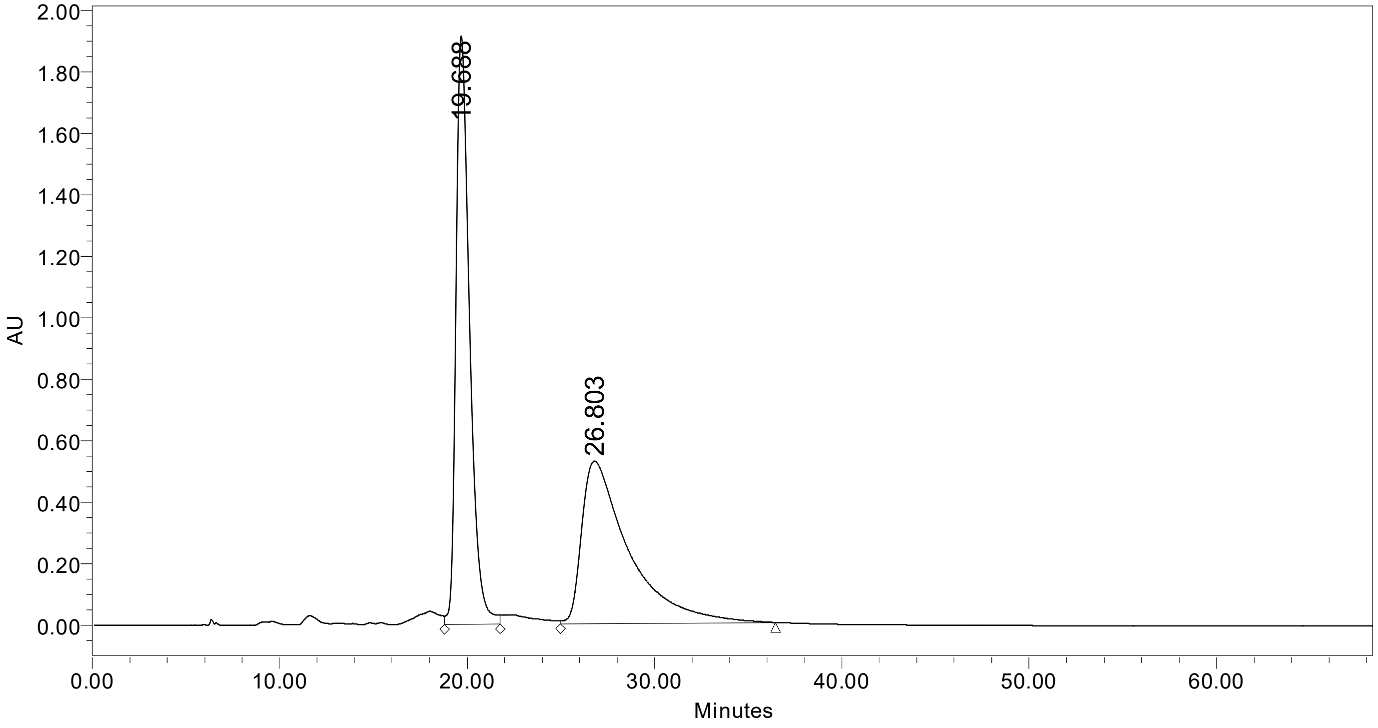

	RT	Area	% Area	Height
1	19.688	97264760	51.30	1914310
2	26.803	92347787	48.70	528374

SAMPLE INFORMATION

Sample Name:	PG71-ODH	Acquired By:	
Sample Type:	Unknown	Sample Set Name:	
Vial:	1	Acq. Method Set:	
Injection #:	8	Processing Method	
Injection Volume:	10.00 ul	Channel Name:	
Run Time:	100.0 Minutes	Proc. Chnl. Descr.:	
Date Acquired:	18-11-2022 18:33:41 IST		
Date Processed:	05-01-2023 18:13:29 IST		

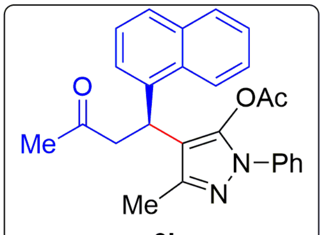

3ia

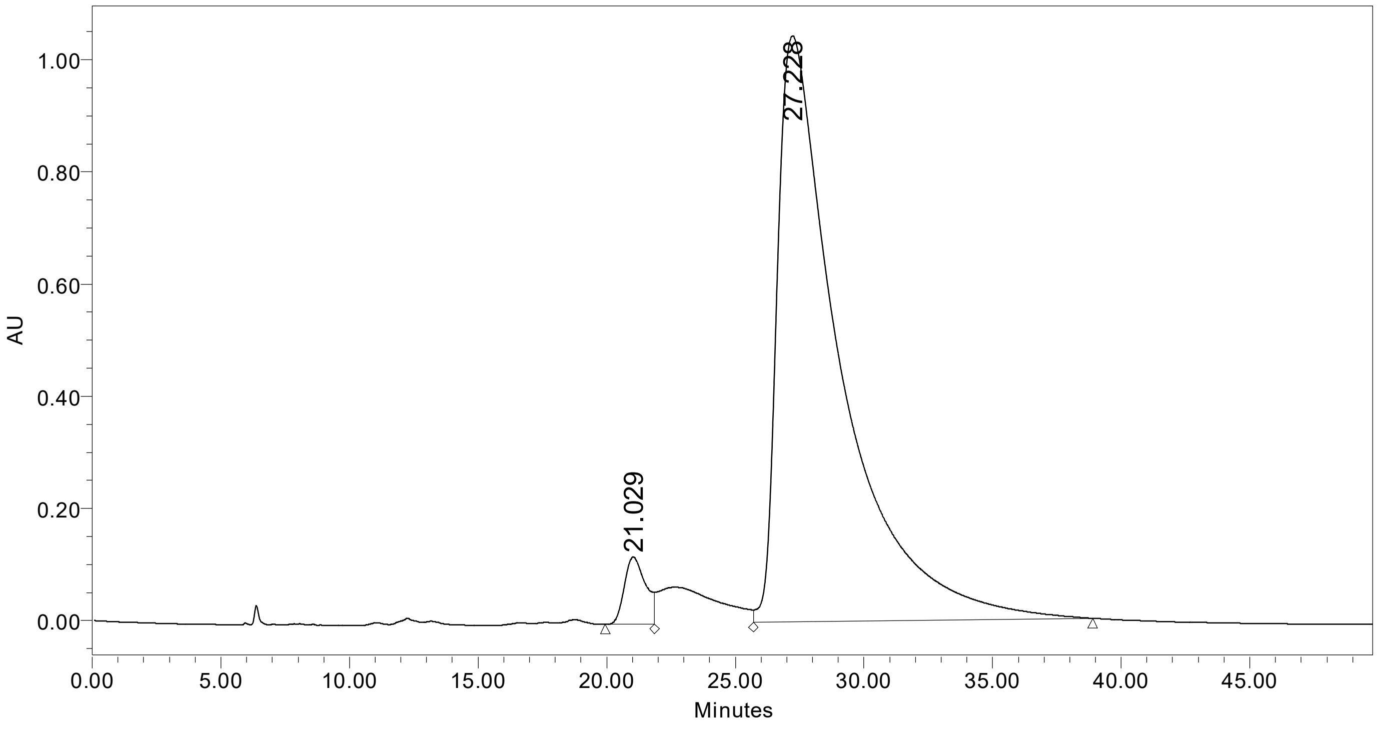

	RT	Area	% Area	Height
1	21.029	6931970	3.72	119451
2	27.228	179338232	96.28	1045594

SAMPLE INFORMATION

Sample Name:	PG71-CRYSTAL	Acquired By:	
Sample Type:	Unknown	Sample Set Name:	
Vial:	1	Acq. Method Set:	
Injection #:	5	Processing Method	
Injection Volume:	10.00 ul	Channel Name:	
Run Time:	100.0 Minutes	Proc. Chnl. Descr.:	
Date Acquired:	10-01-2023 12:52:40 IST		
Date Processed:	10-01-2023 16:47:34 IST		

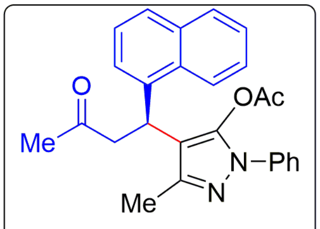

3ia

After single recrystallization

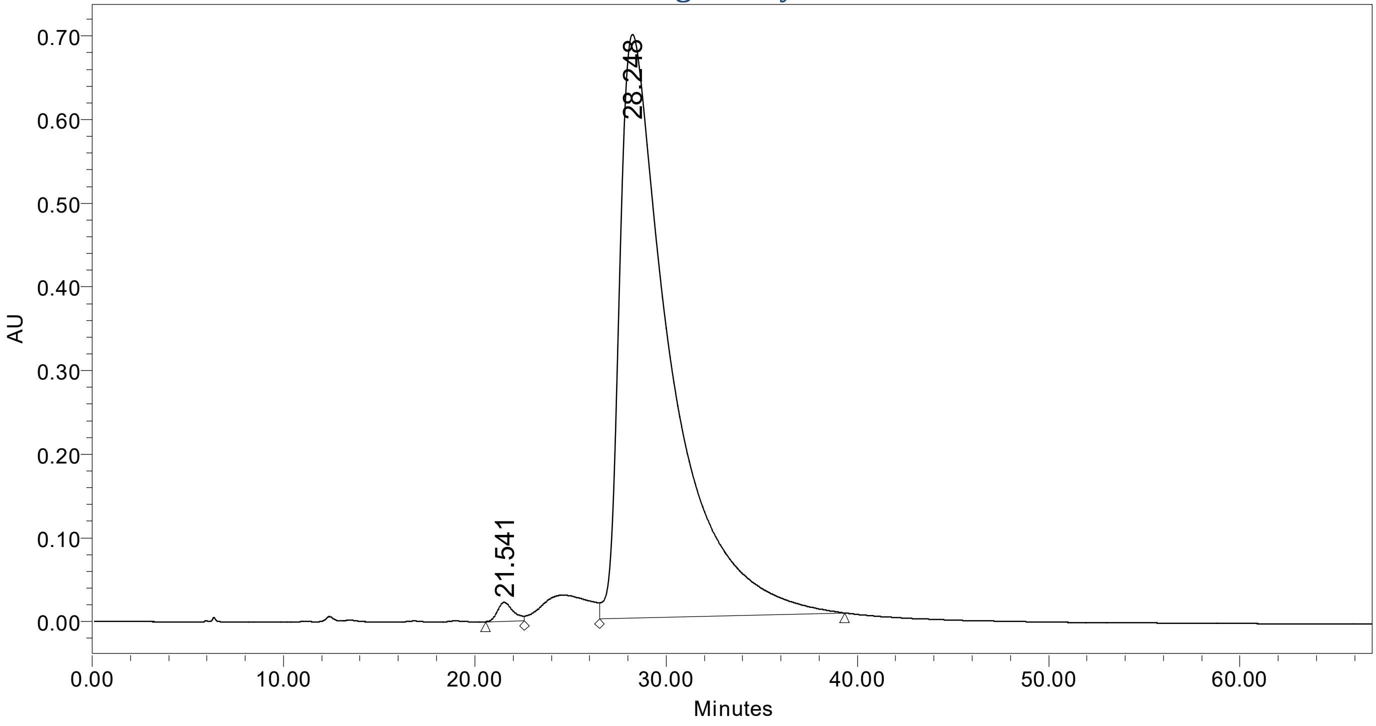

	RT	Area	% Area	Height
1	21.541	1321728	1.01	22629
2	28.248	129626219	98.99	698126

Empower™ 3 SOFTWARE

RC2638PDF

SAMPLE INFORMATION

Sample Name:	PG72-ODH	Acquired By:	
Sample Type:	Unknown	Sample Set Name:	
Vial:	1	Acq. Method Set:	
Injection #:	6	Processing Method	
Injection Volume:	10.00 ul	Channel Name:	
Run Time:	100.0 Minutes	Proc. Chnl. Descr.:	
Date Acquired:	18-11-2022 17:16:34 IST		
Date Processed:	05-01-2023 18:14:30 IST		

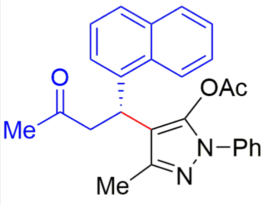

***ent*-3ia**

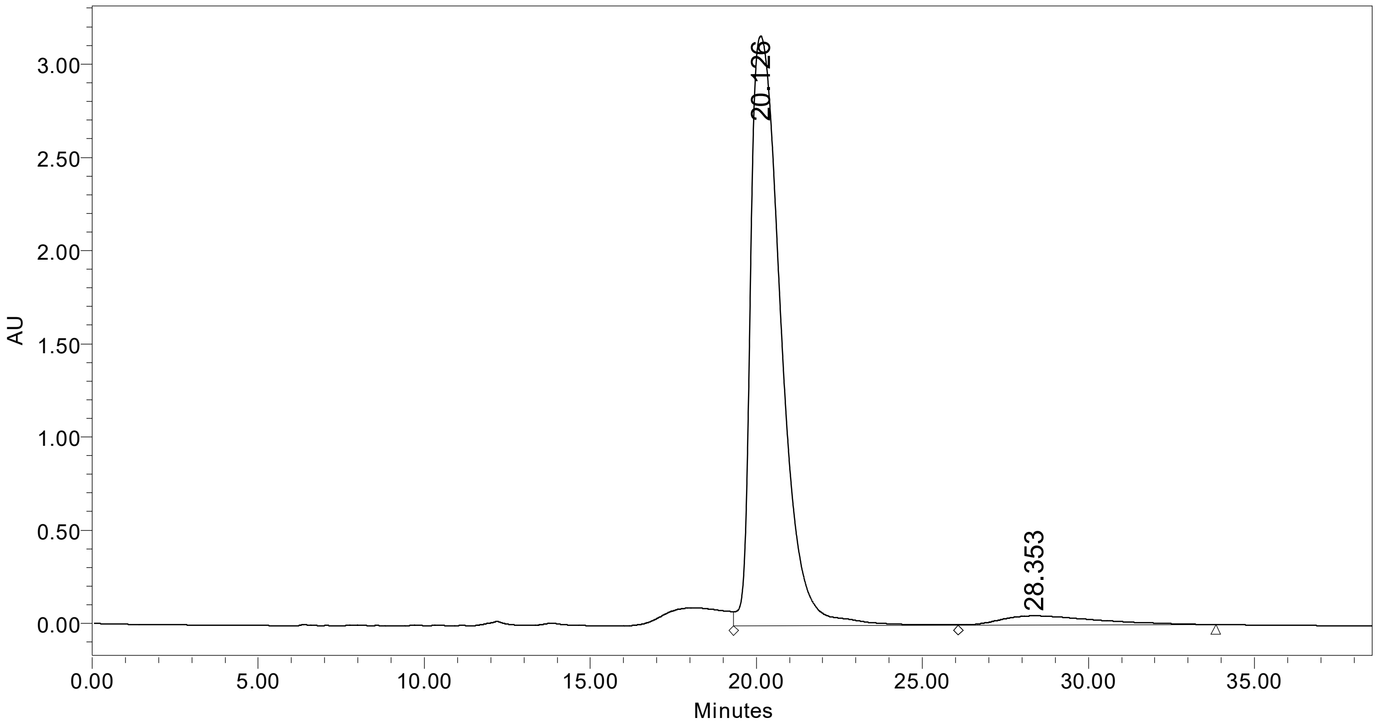

	RT	Area	% Area	Height
1	20.126	200627154	95.30	3163869
2	28.353	9884915	4.70	49079

SAMPLE INFORMATION

Sample Name:	PG72-CRYSTAL	Acquired By:	
Sample Type:	Unknown	Sample Set Name:	
Vial:	1	Acq. Method Set:	
Injection #:	6	Processing Method	
Injection Volume:	10.00 ul	Channel Name:	
Run Time:	100.0 Minutes	Proc. Chnl. Descr.:	
Date Acquired:	10-01-2023 14:04:25 IST		
Date Processed:	10-01-2023 16:48:15 IST		

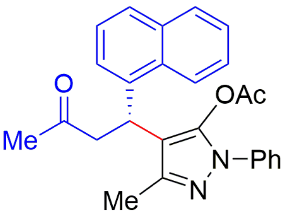

***ent*-3ia**

After single recrystallization

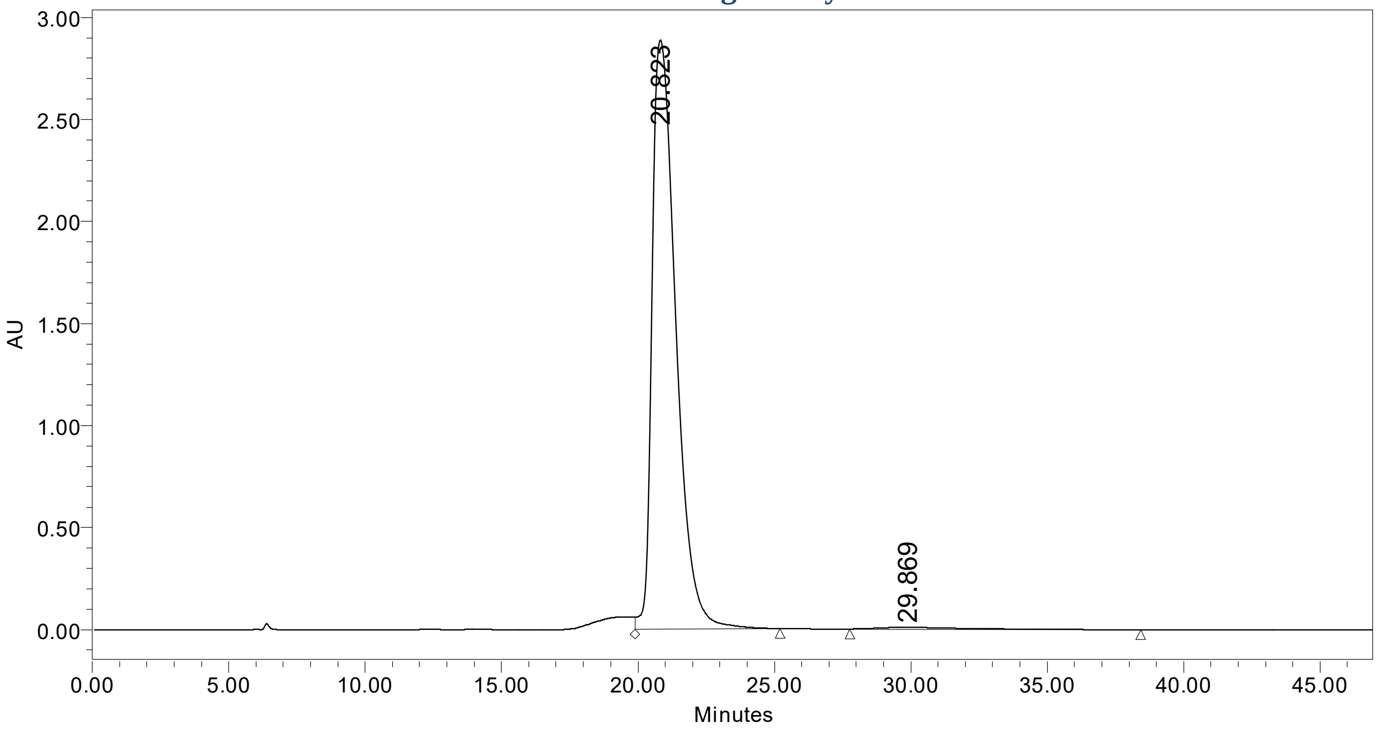

	RT	Area	% Area	Height
1	20.823	175328032	98.88	2887774
2	29.869	1979149	1.12	8729

Empower™ 3 SOFTWARE

RC2638PDF

SAMPLE INFORMATION

Sample Name:	PG90-ODH	Acquired By:	
Sample Type:	Unknown	Sample Set Name:	
Vial:	1	Acq. Method Set:	
Injection #:	2	Processing Method	
Injection Volume:	10.00 ul	Channel Name:	
Run Time:	100.0 Minutes	Proc. Chnl. Descr.:	
Date Acquired:	29-11-2022 18:18:57 IST		
Date Processed:	29-11-2022 18:45:29 IST		

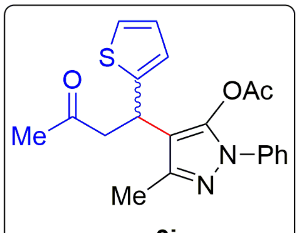

***rac*-3ja**

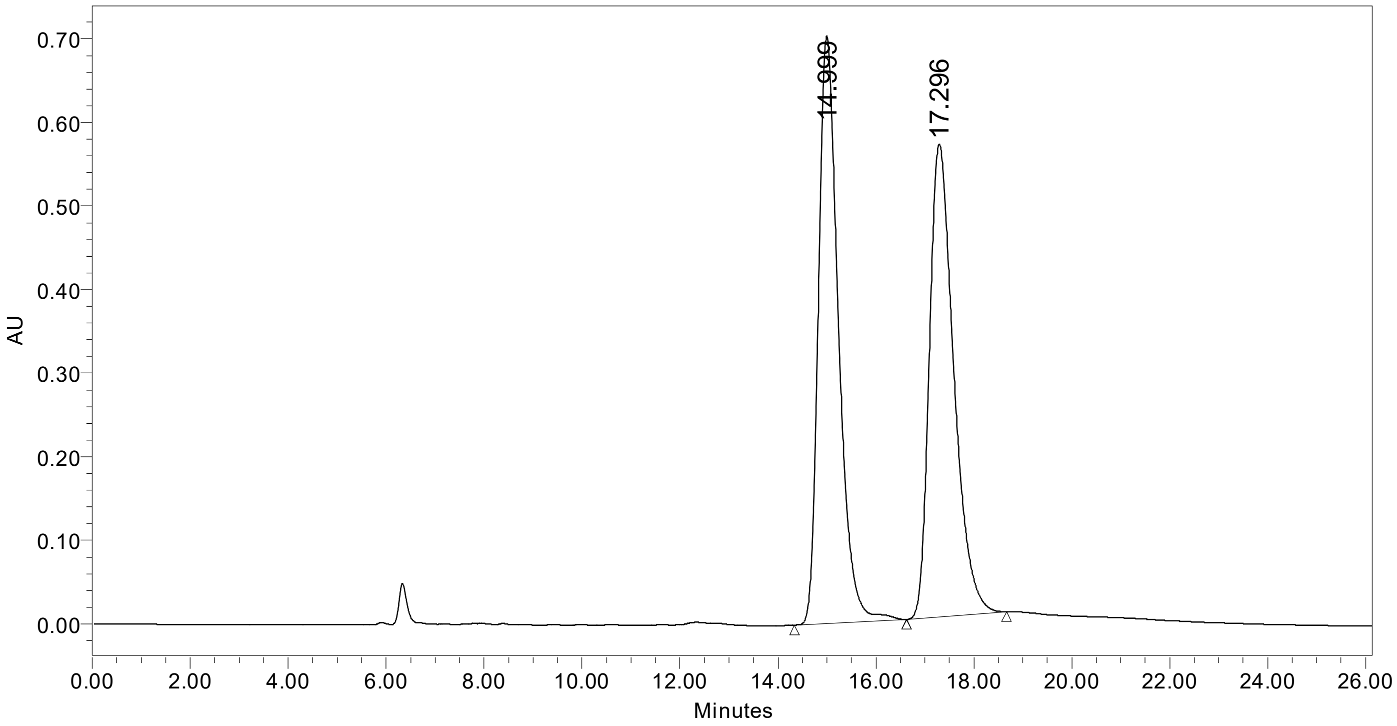

	RT	Area	% Area	Height
1	14.999	20014798	50.51	703234
2	17.296	19614171	49.49	566125

SAMPLE INFORMATION

Sample Name:	PG61-OD-H	Acquired By:	
Sample Type:	Unknown	Sample Set Name:	
Vial:	1	Acq. Method Set:	
Injection #:	3	Processing Method	
Injection Volume:	5.00 ul	Channel Name:	
Run Time:	120.0 Minutes	Proc. Chnl. Descr.:	
Date Acquired:	11-11-2022 11:23:34 IST		
Date Processed:	29-11-2022 18:44:27 IST		

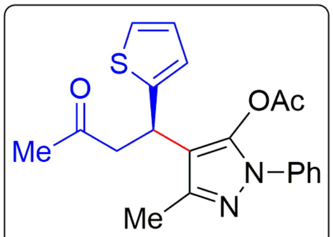

3ja

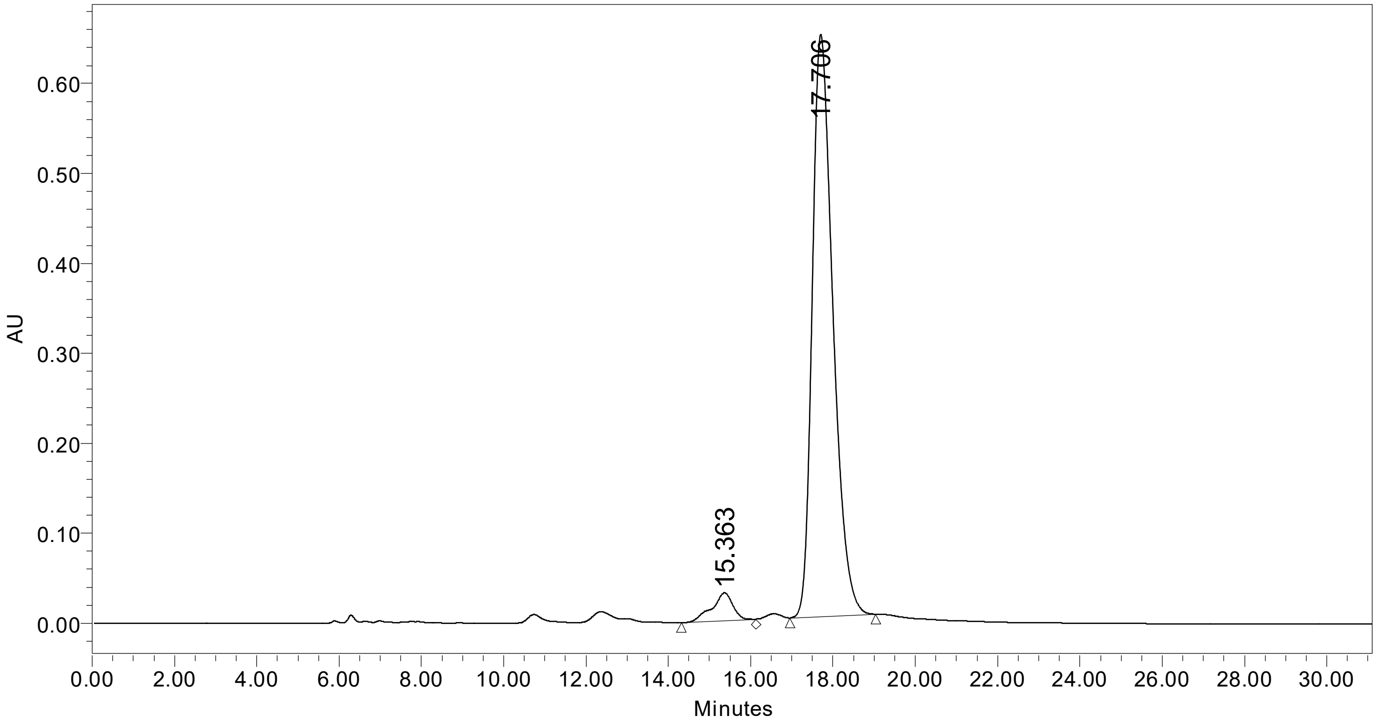

	RT	Area	% Area	Height
1	15.363	1115204	4.70	31144
2	17.706	22595514	95.30	647379

SAMPLE INFORMATION

Sample Name:	PG62-OD-H	Acquired By:	
Sample Type:	Unknown	Sample Set Name:	
Vial:	1	Acq. Method Set:	
Injection #:	4	Processing Method	
Injection Volume:	5.00 ul	Channel Name:	
Run Time:	120.0 Minutes	Proc. Chnl. Descr.:	
Date Acquired:	11-11-2022 11:55:00 IST		
Date Processed:	29-11-2022 18:43:23 IST		

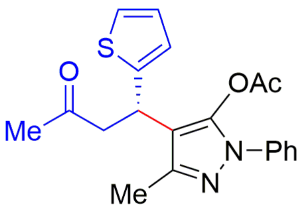

***ent*-3ja**

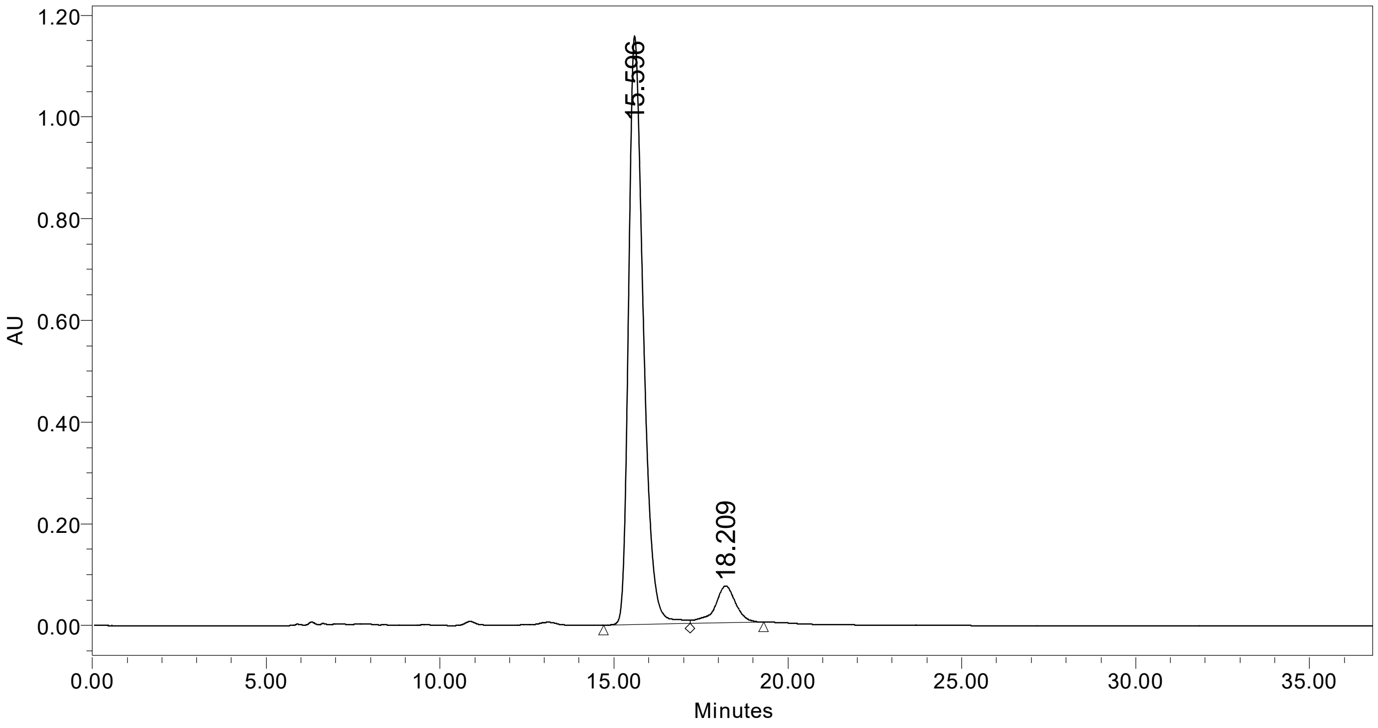

	RT	Area	% Area	Height
1	15.596	34189213	91.75	1157872
2	18.209	3072533	8.25	72535

SAMPLE INFORMATION

Sample Name:	PG73-74-ODH	Acquired By:	
Sample Type:	Unknown	Sample Set Name:	
Vial:	1	Acq. Method Set:	
Injection #:	5	Processing Method	
Injection Volume:	10.00 ul	Channel Name:	
Run Time:	100.0 Minutes	Proc. Chnl. Descr.:	
Date Acquired:	21-11-2022 17:52:34 IST		
Date Processed:	21-11-2022 18:19:56 IST		

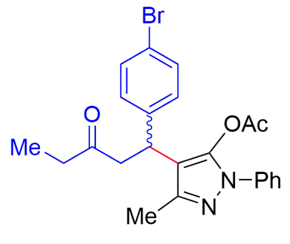

***rac*-3ka**

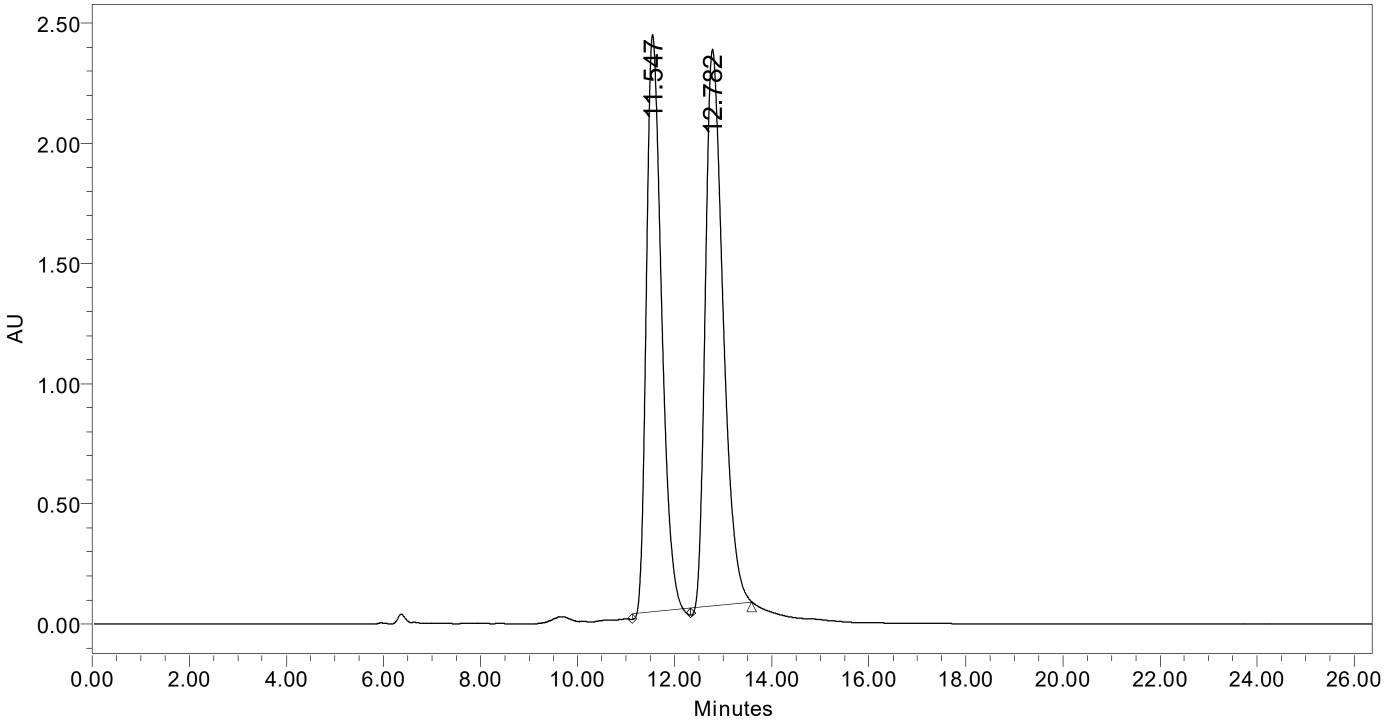

	RT	Area	% Area	Height
1	11.547	52298184	47.06	2402773
2	12.782	58838386	52.94	2314849

SAMPLE INFORMATION

Sample Name:	PG73-ODH	Acquired By:	
Sample Type:	Unknown	Sample Set Name:	
Vial:	1	Acq. Method Set:	
Injection #:	4	Processing Method	
Injection Volume:	10.00 ul	Channel Name:	
Run Time:	100.0 Minutes	Proc. Chnl. Descr.:	
Date Acquired:	21-11-2022 17:09:04 IST		
Date Processed:	21-11-2022 18:20:51 IST		

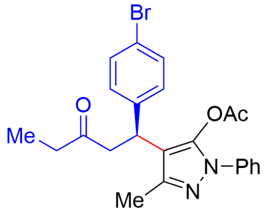

3ka

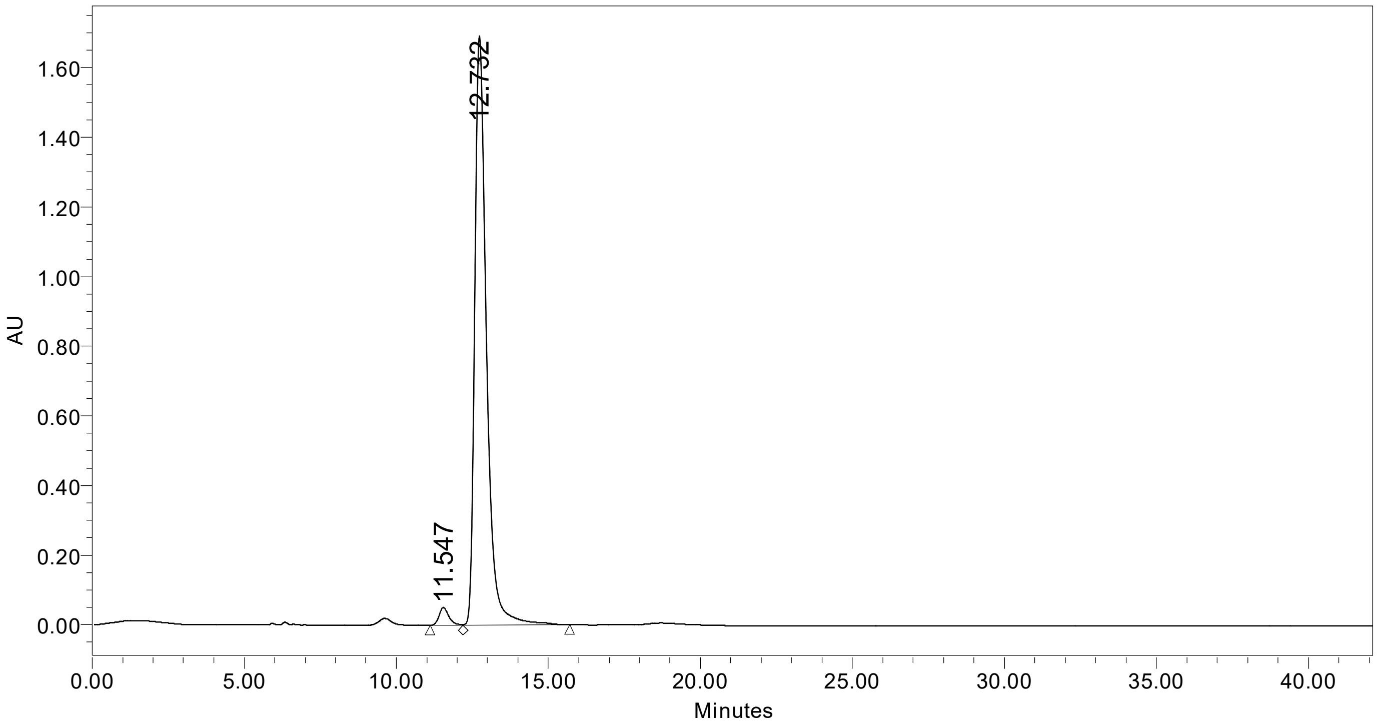

	RT	Area	% Area	Height
1	11.547	1137990	2.44	50589
2	12.732	45443346	97.56	1692533

SAMPLE INFORMATION

Sample Name:	PG73-CRYSTAL	Acquired By:	
Sample Type:	Unknown	Sample Set Name:	
Vial:	1	Acq. Method Set:	
Injection #:	2	Processing Method	
Injection Volume:	10.00 ul	Channel Name:	
Run Time:	100.0 Minutes	Proc. Chnl. Descr.:	
Date Acquired:	10-01-2023 11:23:27 IST		
Date Processed:	10-01-2023 16:45:48 IST		

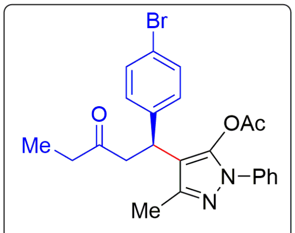

3ka

After single recrystallization

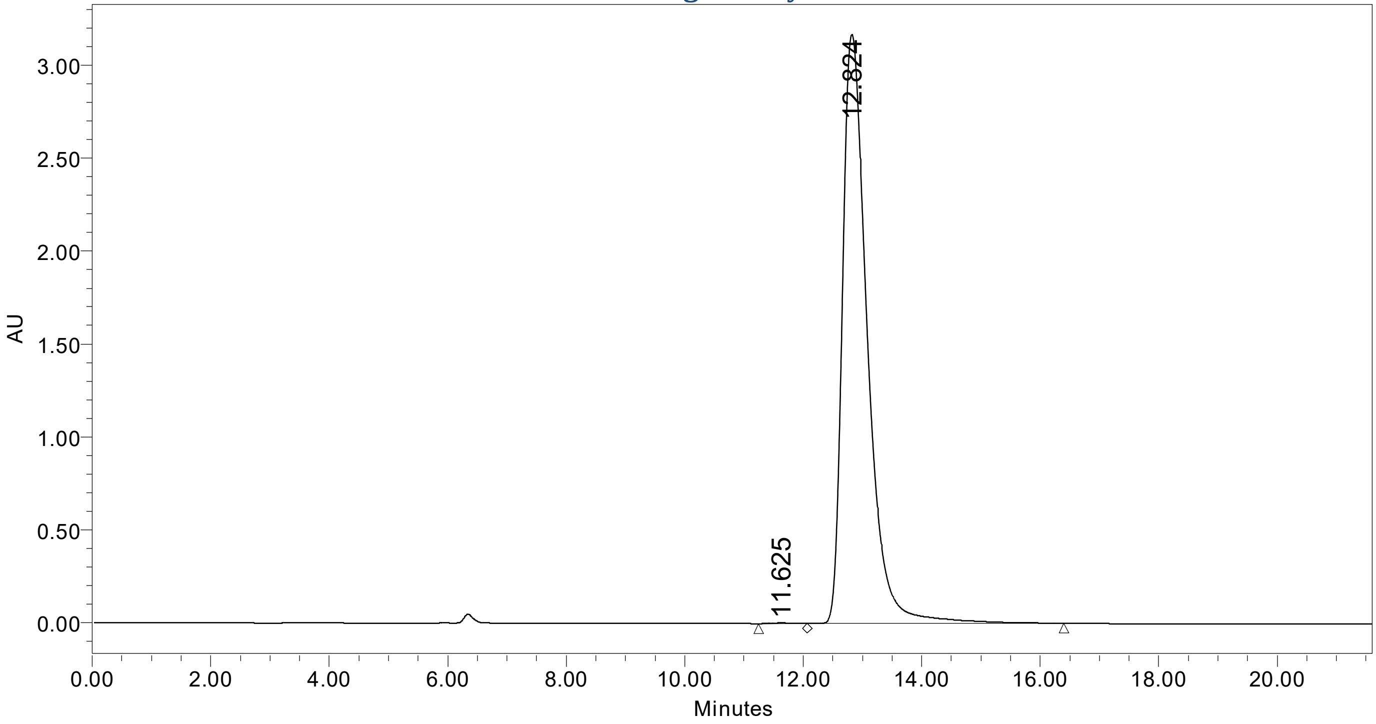

	RT	Area	% Area	Height
1	11.625	70652	0.08	3386
2	12.824	92497025	99.92	3170912

SAMPLE INFORMATION

Sample Name:	PG74-ODH	Acquired By:	
Sample Type:	Unknown	Sample Set Name:	
Vial:	1	Acq. Method Set:	
Injection #:	3	Processing Method	
Injection Volume:	10.00 ul	Channel Name:	
Run Time:	100.0 Minutes	Proc. Chnl. Descr.:	
Date Acquired:	21-11-2022 16:47:28 IST		
Date Processed:	21-11-2022 18:21:58 IST		

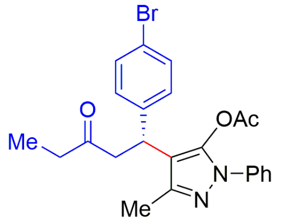

***ent*-3ka**

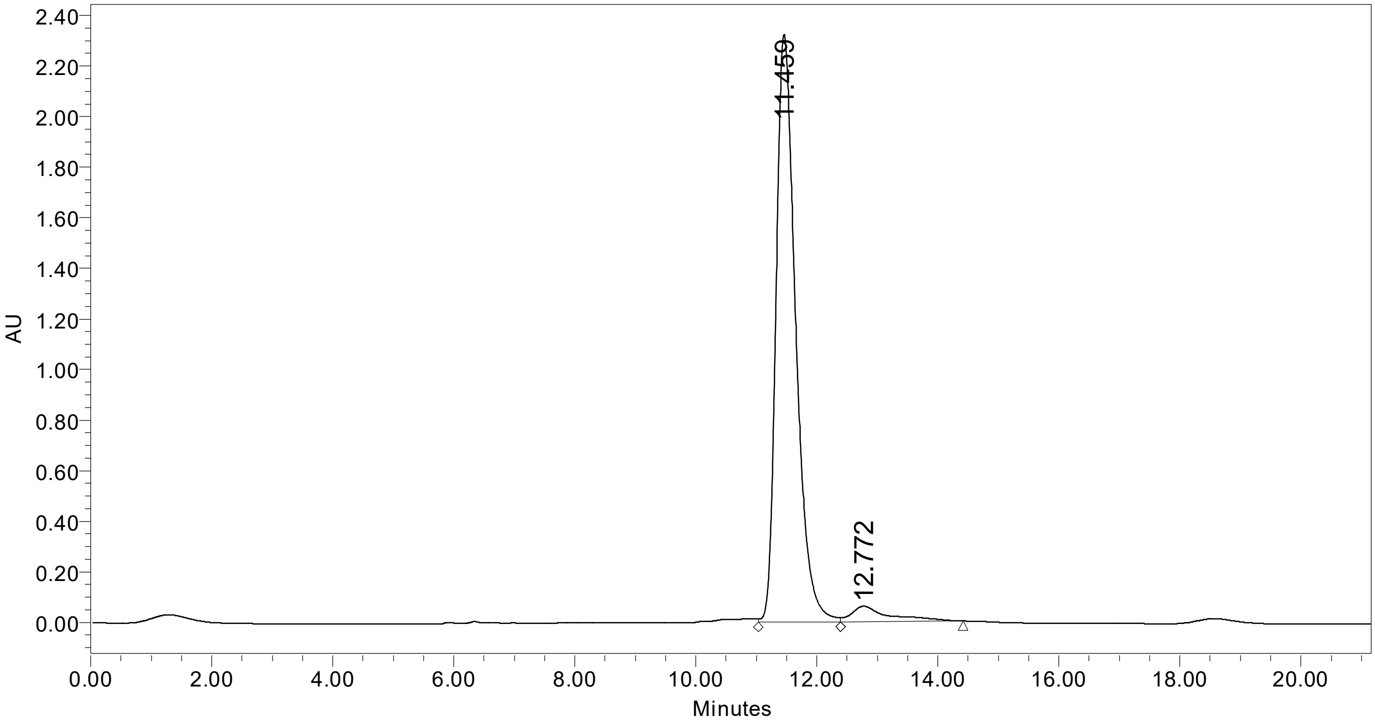

	RT	Area	% Area	Height
1	11.459	52228139	95.15	2323576
2	12.772	2659323	4.85	61064

S A M P L E   I N F O R M A T I O N

Sample Name:	PG74-CRYSTAL	Acquired By:	
Sample Type:	Unknown	Sample Set Name:	
Vial:	1	Acq. Method Set:	
Injection #:	3	Processing Method	
Injection Volume:	10.00 ul	Channel Name:	
Run Time:	100.0 Minutes	Proc. Chnl. Descr.:	
Date Acquired:	10-01-2023 11:45:31 IST		
Date Processed:	10-01-2023 16:46:40 IST		

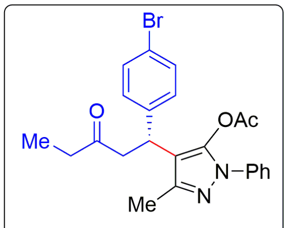

***ent*-3ka**

After single recrystallization

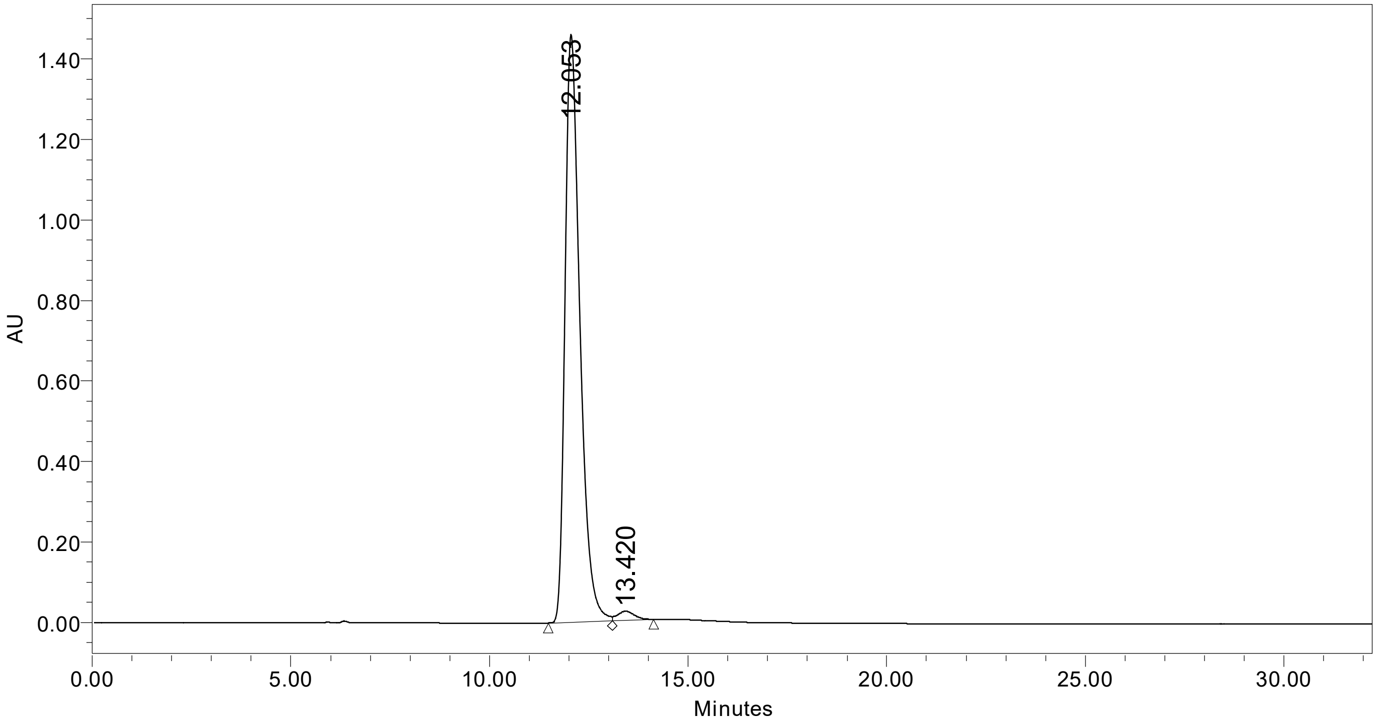

	RT	Area	% Area	Height
1	12.053	38089922	98.23	1461076
2	13.420	685749	1.77	22829

SAMPLE INFORMATION

Sample Name:	PD16-OD-H	Acquired By:	
Sample Type:	Unknown	Sample Set Name:	
Vial:	1	Acq. Method Set:	
Injection #:	5	Processing Method	
Injection Volume:	5.00 ul	Channel Name:	
Run Time:	120.0 Minutes	Proc. Chnl. Descr.:	
Date Acquired:	19-10-2022 17:16:14 IST		
Date Processed:	05-01-2023 18:03:44 IST		

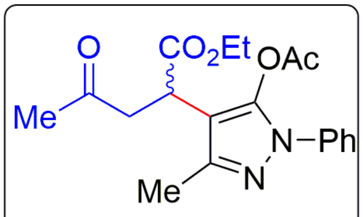

***rac*-3la**

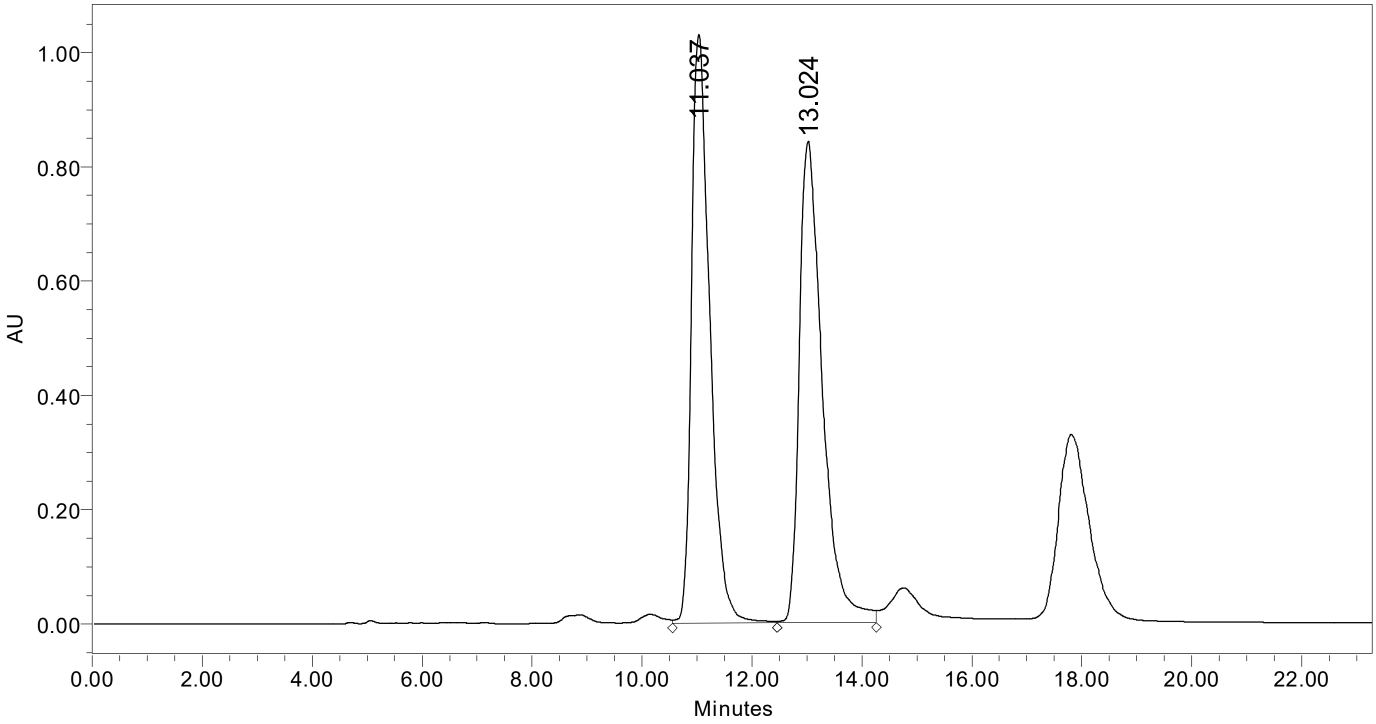

	RT	Area	% Area	Height
1	11.037	23992096	49.30	1030252
2	13.024	24677237	50.70	842260

SAMPLE INFORMATION

Sample Name:	PG46-OD-H	Acquired By:	
Sample Type:	Unknown	Sample Set Name:	
Vial:	1	Acq. Method Set:	
Injection #:	8	Processing Method	
Injection Volume:	5.00 ul	Channel Name:	
Run Time:	120.0 Minutes	Proc. Chnl. Descr.:	
Date Acquired:	28-10-2022 15:55:34 IST		
Date Processed:	28-10-2022 16:23:08 IST		

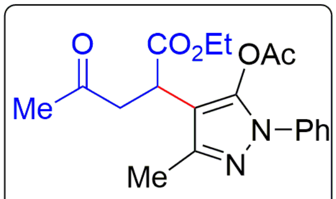

3la

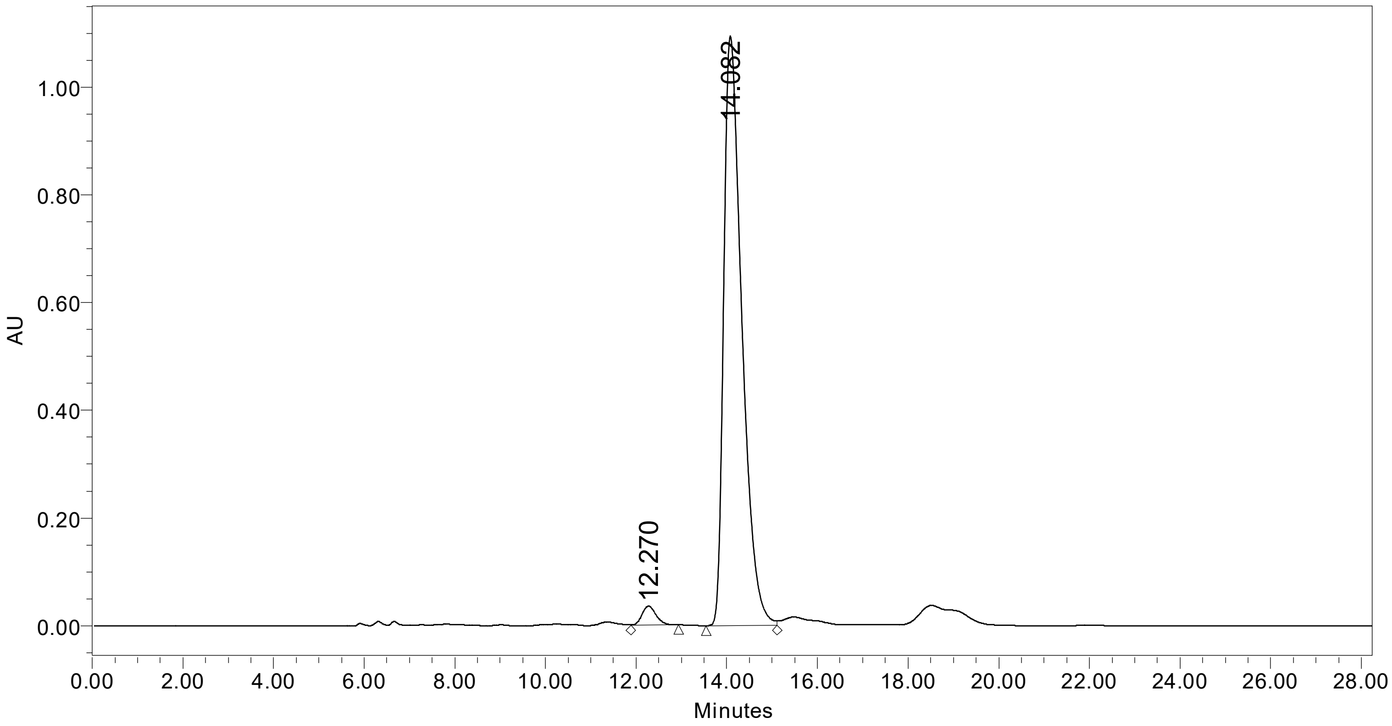

	RT	Area	% Area	Height
1	12.270	767204	2.46	35629
2	14.082	30453488	97.54	1095596

SAMPLE INFORMATION

Sample Name:	PG47-OD-H	Acquired By:	
Sample Type:	Unknown	Sample Set Name:	
Vial:	1	Acq. Method Set:	
Injection #:	10	Processing Method	
Injection Volume:	5.00 ul	Channel Name:	
Run Time:	120.0 Minutes	Proc. Chnl. Descr.:	
Date Acquired:	28-10-2022 16:51:04 IST		
Date Processed:	28-10-2022 17:24:05 IST		

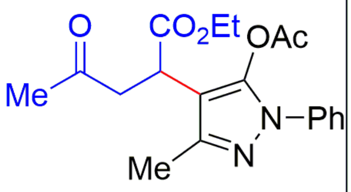

***ent*-3la**

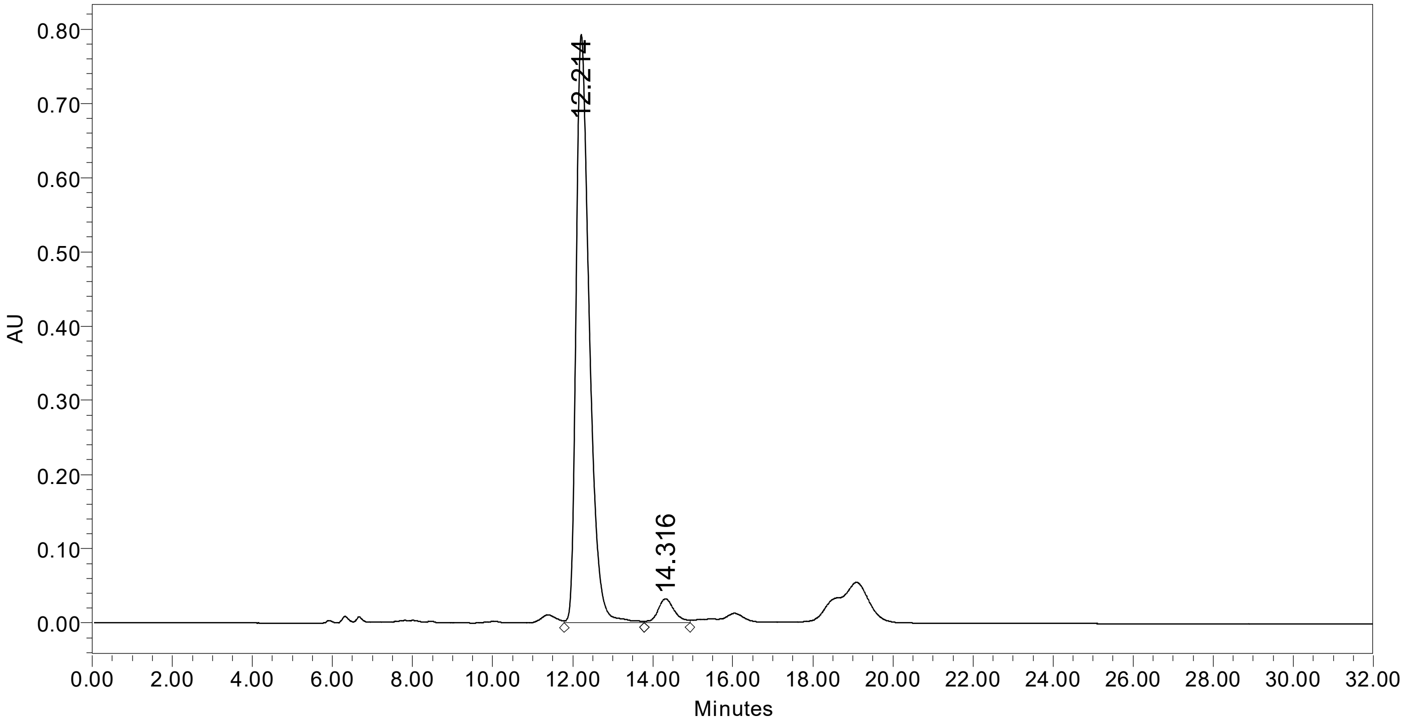

	RT	Area	% Area	Height
1	12.214	18248661	95.32	792879
2	14.316	896587	4.68	31713

SAMPLE INFORMATION

Sample Name:	AKD-640-ODH	Acquired By:	
Sample Type:	Unknown	Sample Set Name:	
Vial:	1	Acq. Method Set:	
Injection #:	1	Processing Method	
Injection Volume:	10.00 ul	Channel Name:	
Run Time:	100.0 Minutes	Proc. Chnl. Descr.:	
Date Acquired:	02-12-2022 16:31:34 IST		
Date Processed:	02-12-2022 18:04:00 IST		

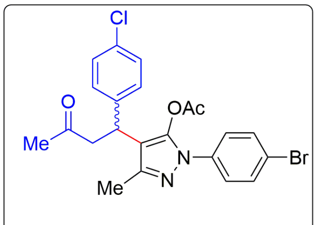

***rac*-3cb**

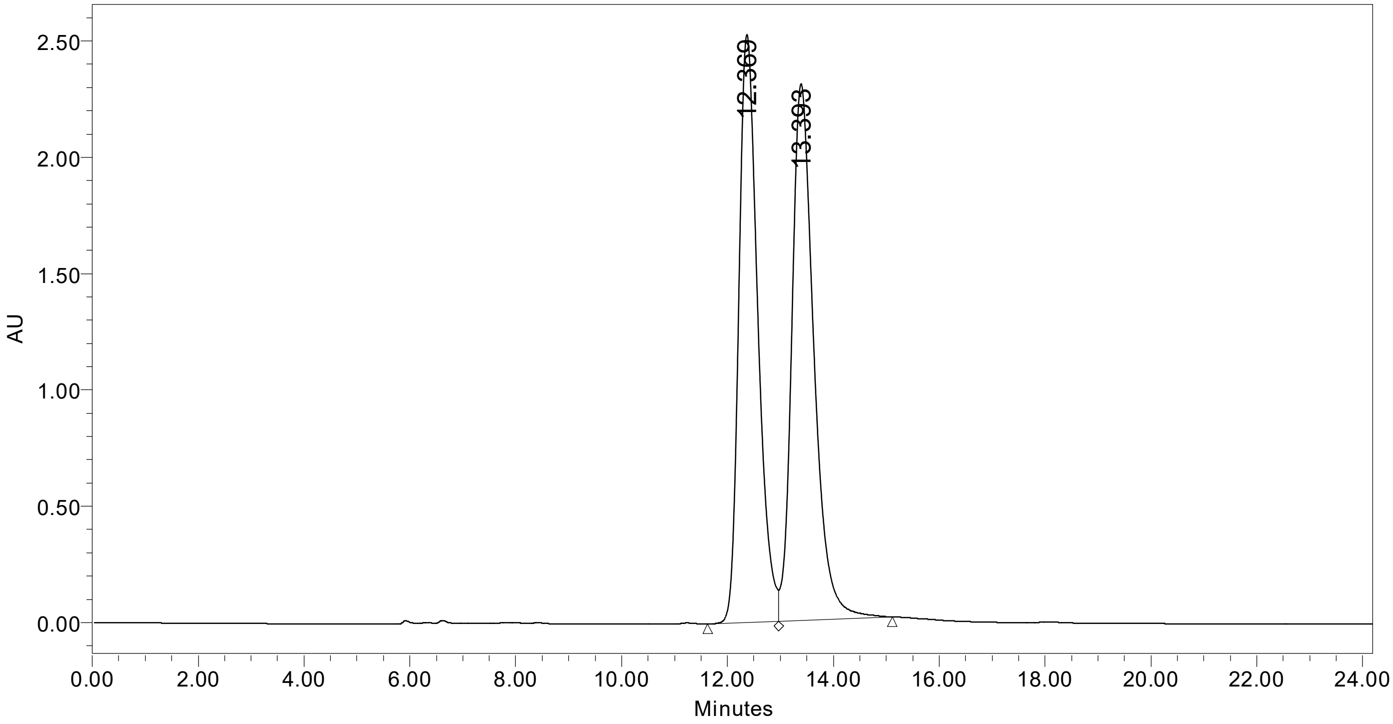

	RT	Area	% Area	Height
1	12.369	62972586	48.48	2528478
2	13.393	66918108	51.52	2306666

SAMPLE INFORMATION

Sample Name:	PG97-ODH	Acquired By:	
Sample Type:	Unknown	Sample Set Name:	
Vial:	1	Acq. Method Set:	
Injection #:	2	Processing Method	
Injection Volume:	10.00 ul	Channel Name:	
Run Time:	100.0 Minutes	Proc. Chnl. Descr.:	
Date Acquired:	02-12-2022 16:56:12 IST		
Date Processed:	02-12-2022 18:04:47 IST		

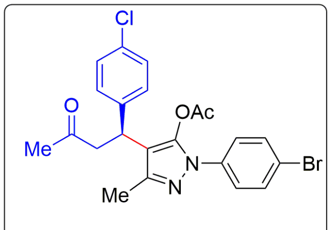

3cb

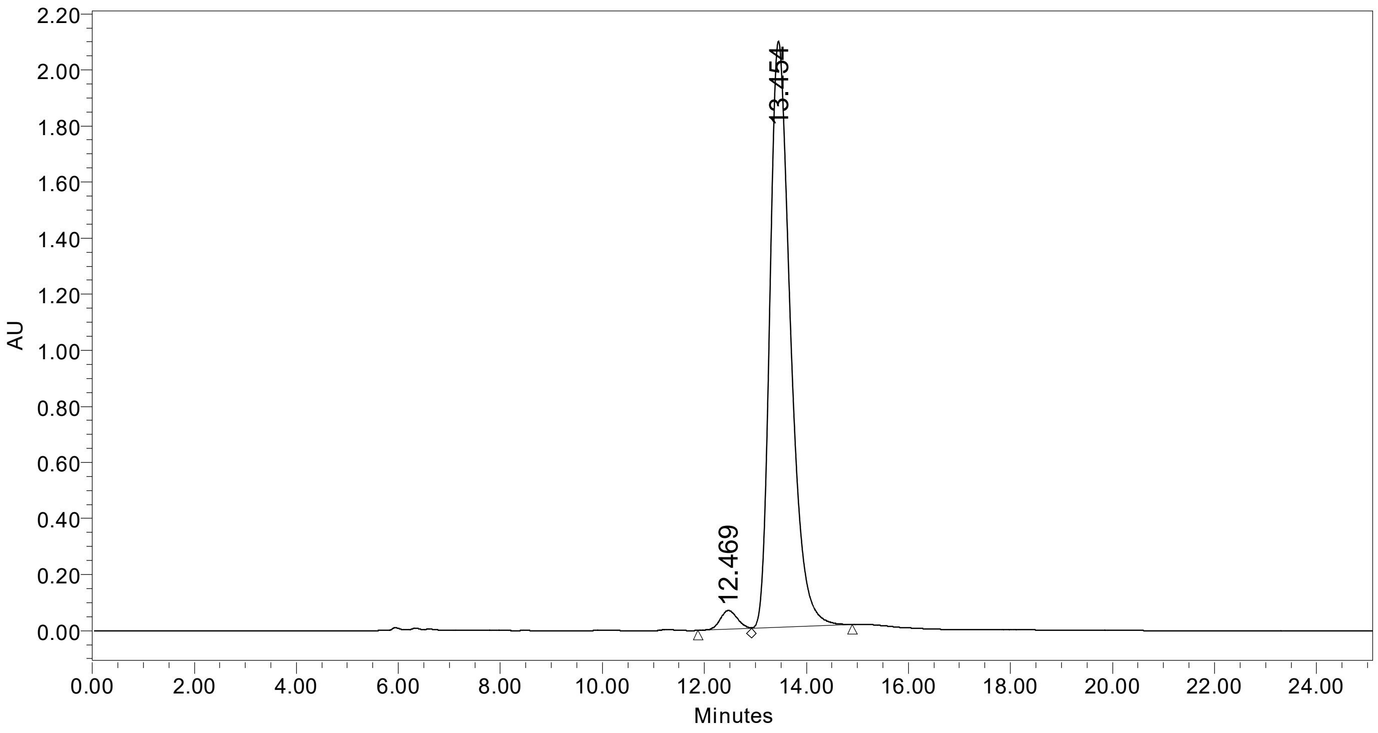

	RT	Area	% Area	Height
1	12.469	1563532	2.60	66887
2	13.454	58606545	97.40	2091950

SAMPLE INFORMATION

Sample Name:	PG97-CRYSTAL	Acquired By:	
Sample Type:	Unknown	Sample Set Name:	
Vial:	1	Acq. Method Set:	
Injection #:	10	Processing Method	
Injection Volume:	10.00 ul	Channel Name:	
Run Time:	100.0 Minutes	Proc. Chnl. Descr.:	
Date Acquired:	10-01-2023 18:12:09 IST		
Date Processed:	13-01-2023 11:32:37 IST		

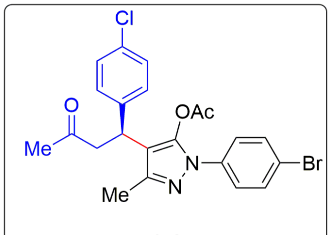

3cb

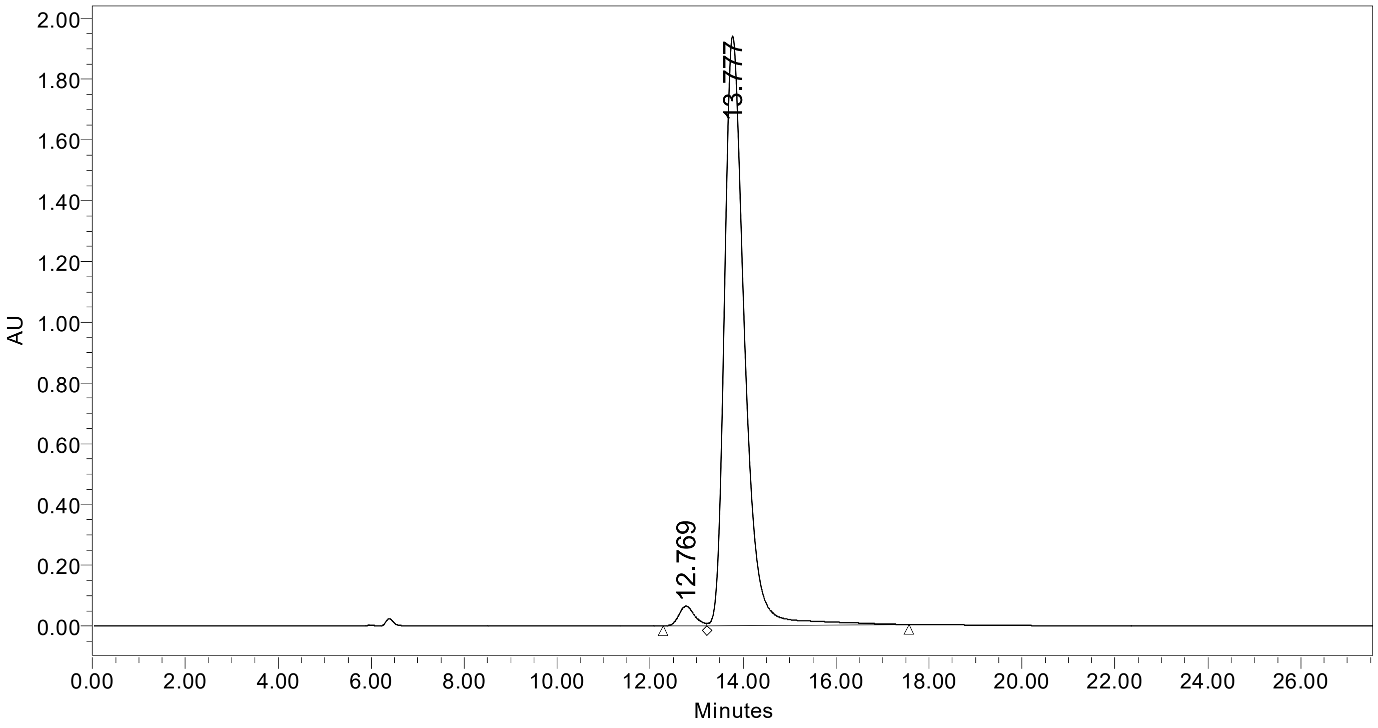

	RT	Area	% Area	Height
1	12.769	1512814	2.52	64702
2	13.777	58514028	97.48	1941482

SAMPLE INFORMATION

Sample Name:	PG98-ODH	Acquired By:	
Sample Type:	Unknown	Sample Set Name:	
Vial:	1	Acq. Method Set:	
Injection #:	4	Processing Method	
Injection Volume:	10.00 ul	Channel Name:	
Run Time:	100.0 Minutes	Proc. Chnl. Descr.:	
Date Acquired:	02-12-2022 17:48:27 IST		
Date Processed:	02-12-2022 18:06:02 IST		

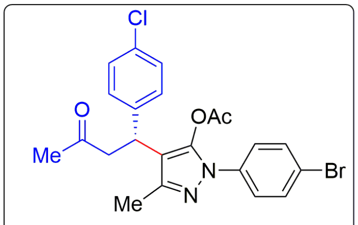

ent-3cb

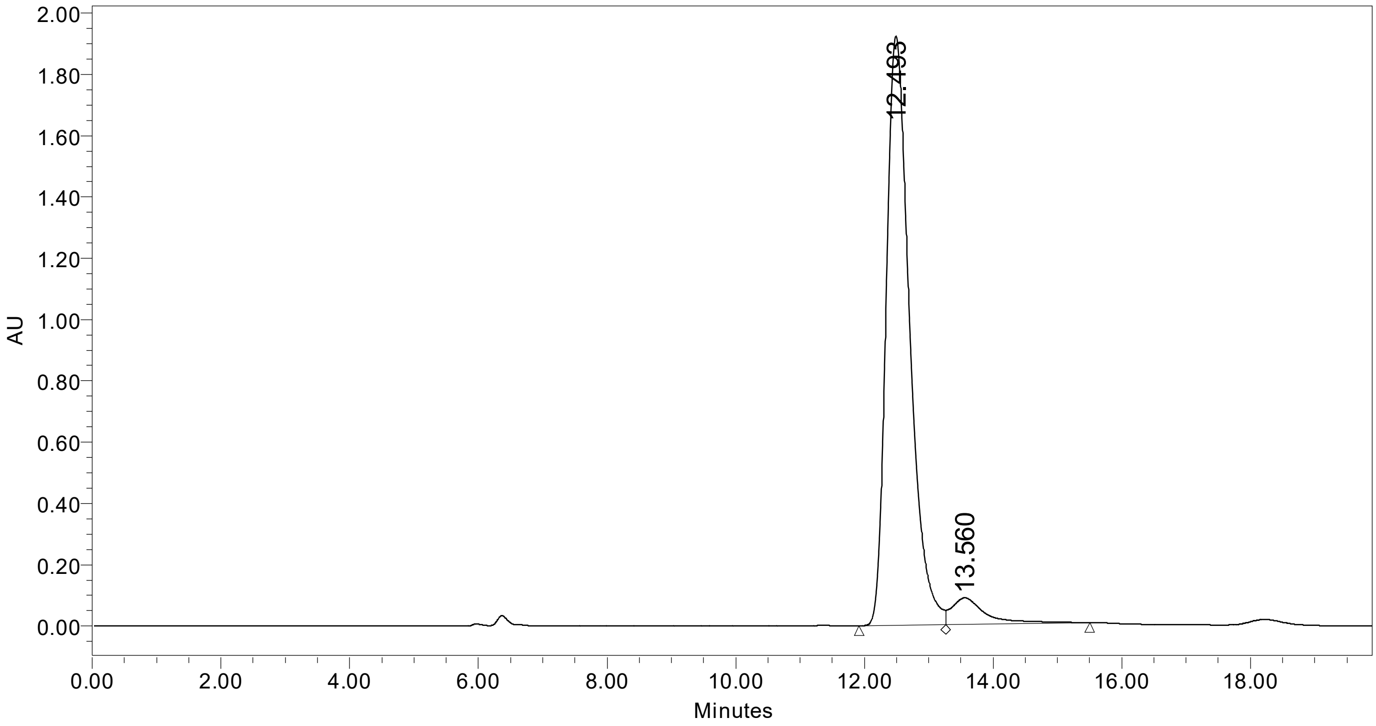

	RT	Area	% Area	Height
1	12.493	48515680	93.82	1923904
2	13.560	3196385	6.18	86538

SAMPLE INFORMATION

Sample Name:	PG98-CRYSTAL	Acquired By:	
Sample Type:	Unknown	Sample Set Name:	
Vial:	1	Acq. Method Set:	
Injection #:	11	Processing Method	
Injection Volume:	10.00 ul	Channel Name:	
Run Time:	100.0 Minutes	Proc. Chnl. Descr.:	
Date Acquired:	10-01-2023 18:40:02 IST		
Date Processed:	13-01-2023 11:31:49 IST		

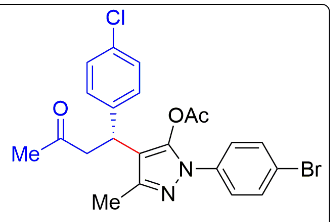

***ent*-3cb**

After single recrystallization

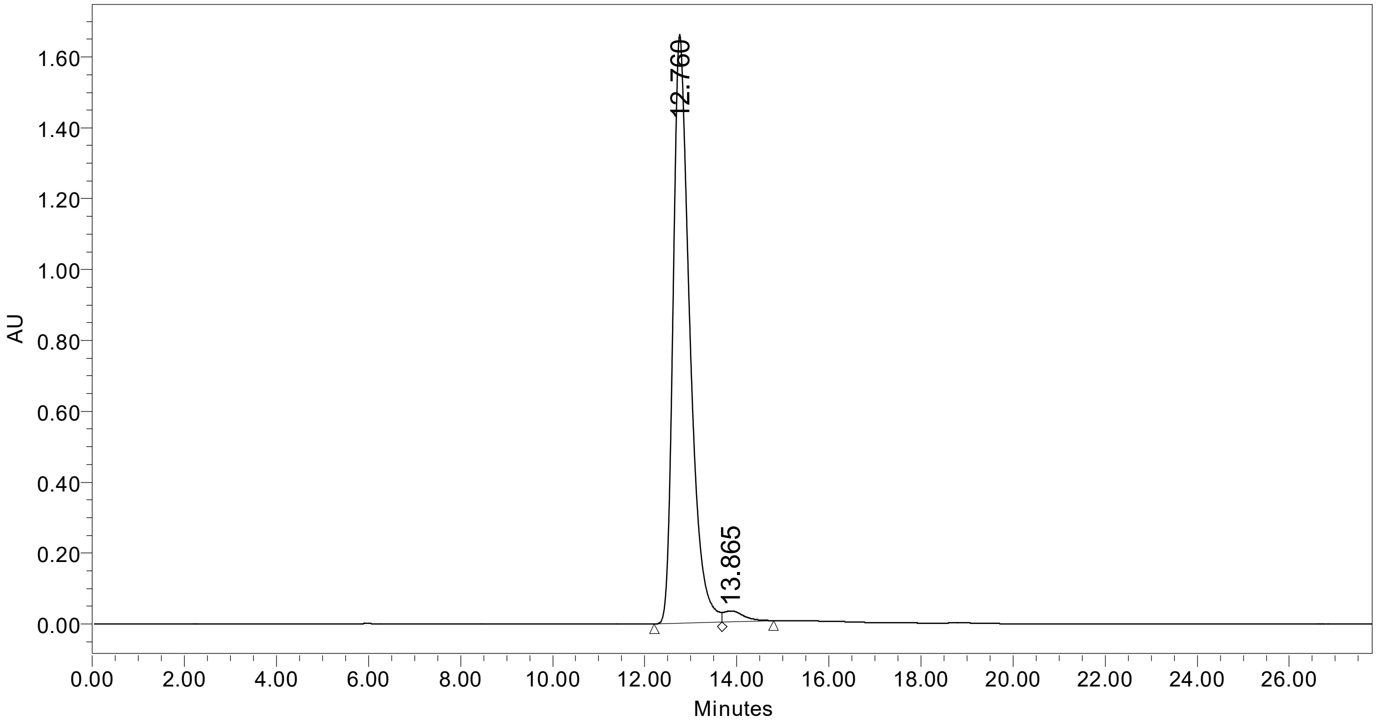

	RT	Area	% Area	Height
1	12.760	41757493	97.85	1661616
2	13.865	915439	2.15	30407

SAMPLE INFORMATION

Sample Name:	AKD-641-ODH	Acquired By:	
Sample Type:	Unknown	Sample Set Name:	
Vial:	1	Acq. Method Set:	
Injection #:	10	Processing Method	
Injection Volume:	10.00 ul	Channel Name:	
Run Time:	100.0 Minutes	Proc. Chnl. Descr.:	
Date Acquired:	01-12-2022 18:22:08 IST		
Date Processed:	05-01-2023 18:16:12 IST		

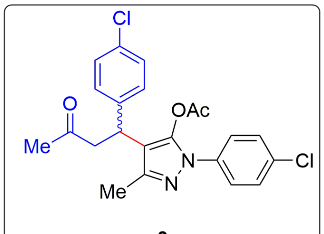

rac-3cc

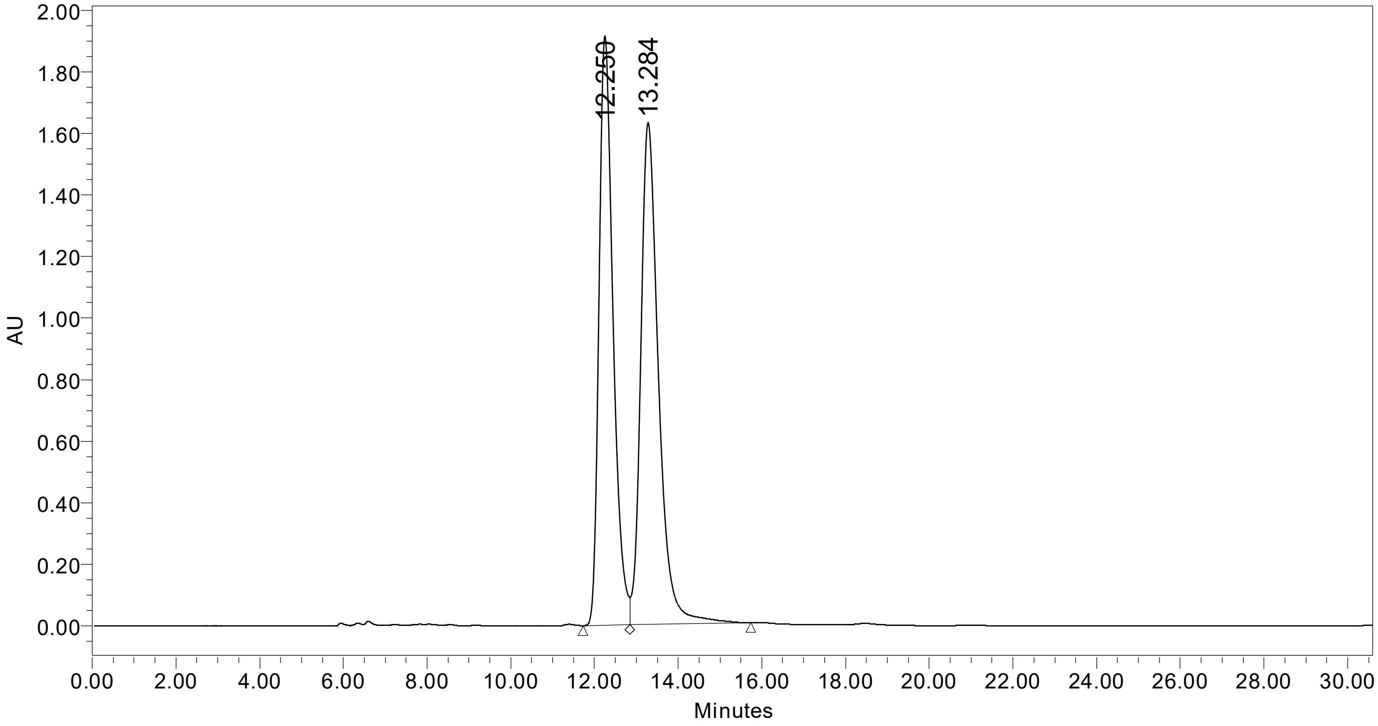

	RT	Area	% Area	Height
1	12.250	44809995	48.17	1914279
2	13.284	48223287	51.83	1630586

SAMPLE INFORMATION

Sample Name:	PG95-ODH	Acquired By:	
Sample Type:	Unknown	Sample Set Name:	
Vial:	1	Acq. Method Set:	
Injection #:	8	Processing Method	
Injection Volume:	10.00 ul	Channel Name:	
Run Time:	100.0 Minutes	Proc. Chnl. Descr.:	
Date Acquired:	01-12-2022 17:33:13 IST		
Date Processed:	02-12-2022 18:01:35 IST		

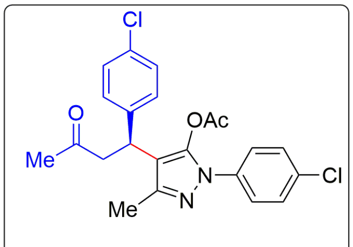

3cc

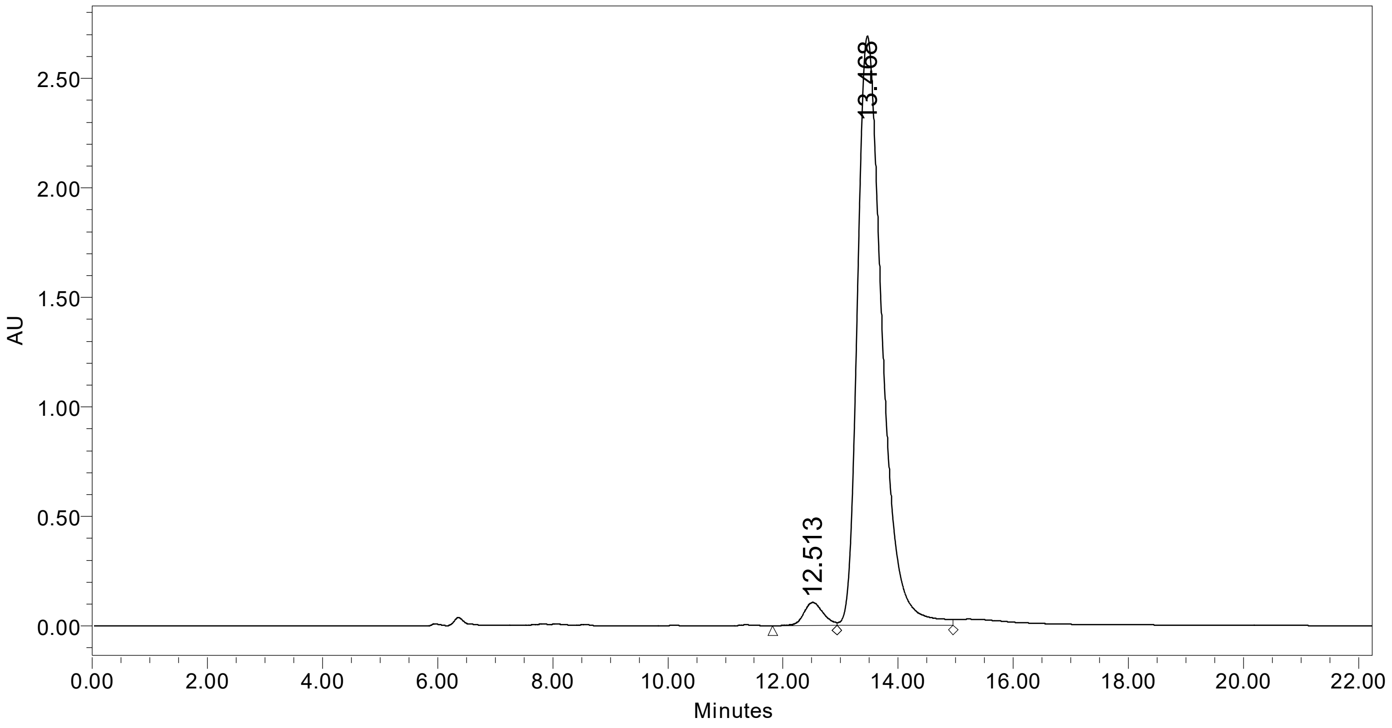

	RT	Area	% Area	Height
1	12.513	2499088	3.04	105584
2	13.468	79695968	96.96	2691451

SAMPLE INFORMATION

Sample Name:	PG95-CRYSTAL	Acquired By:	
Sample Type:	Unknown	Sample Set Name:	
Vial:	1	Acq. Method Set:	
Injection #:	8	Processing Method	
Injection Volume:	10.00 ul	Channel Name:	
Run Time:	100.0 Minutes	Proc. Chnl. Descr.:	
Date Acquired:	10-01-2023 17:06:05 IST		
Date Processed:	10-01-2023 18:07:35 IST		

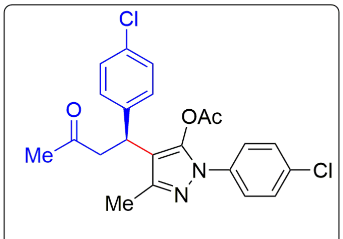

3cc

After single recrystallization

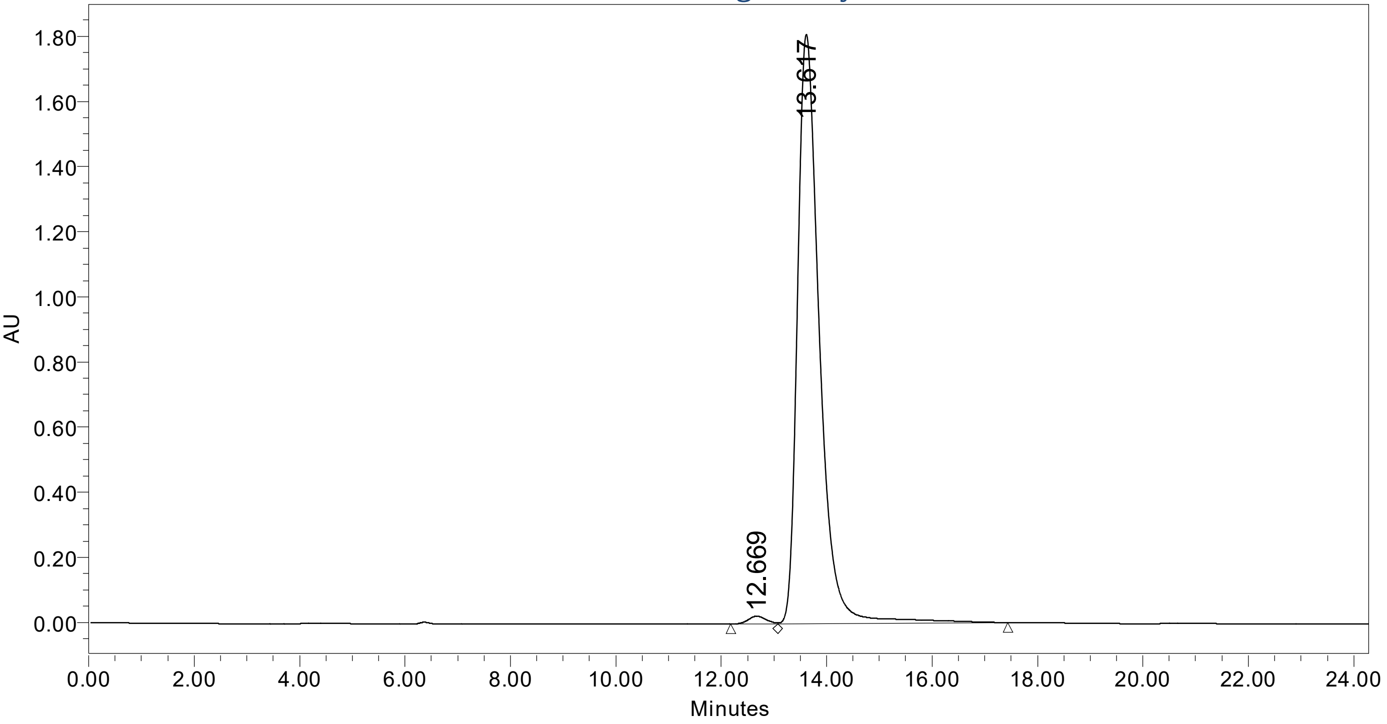

	RT	Area	% Area	Height
1	12.669	553428	1.04	23763
2	13.617	52426960	98.96	1809397

SAMPLE INFORMATION

Sample Name:	PG96-ODH	Acquired By:	
Sample Type:	Unknown	Sample Set Name:	
Vial:	1	Acq. Method Set:	
Injection #:	9	Processing Method	
Injection Volume:	10.00 ul	Channel Name:	
Run Time:	100.0 Minutes	Proc. Chnl. Descr.:	
Date Acquired:	01-12-2022 17:55:46 IST		
Date Processed:	02-12-2022 18:02:41 IST		

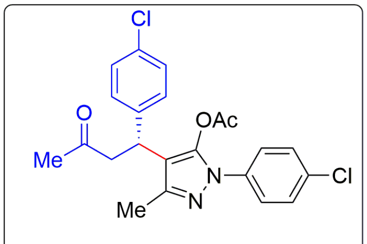

***ent*-3cc**

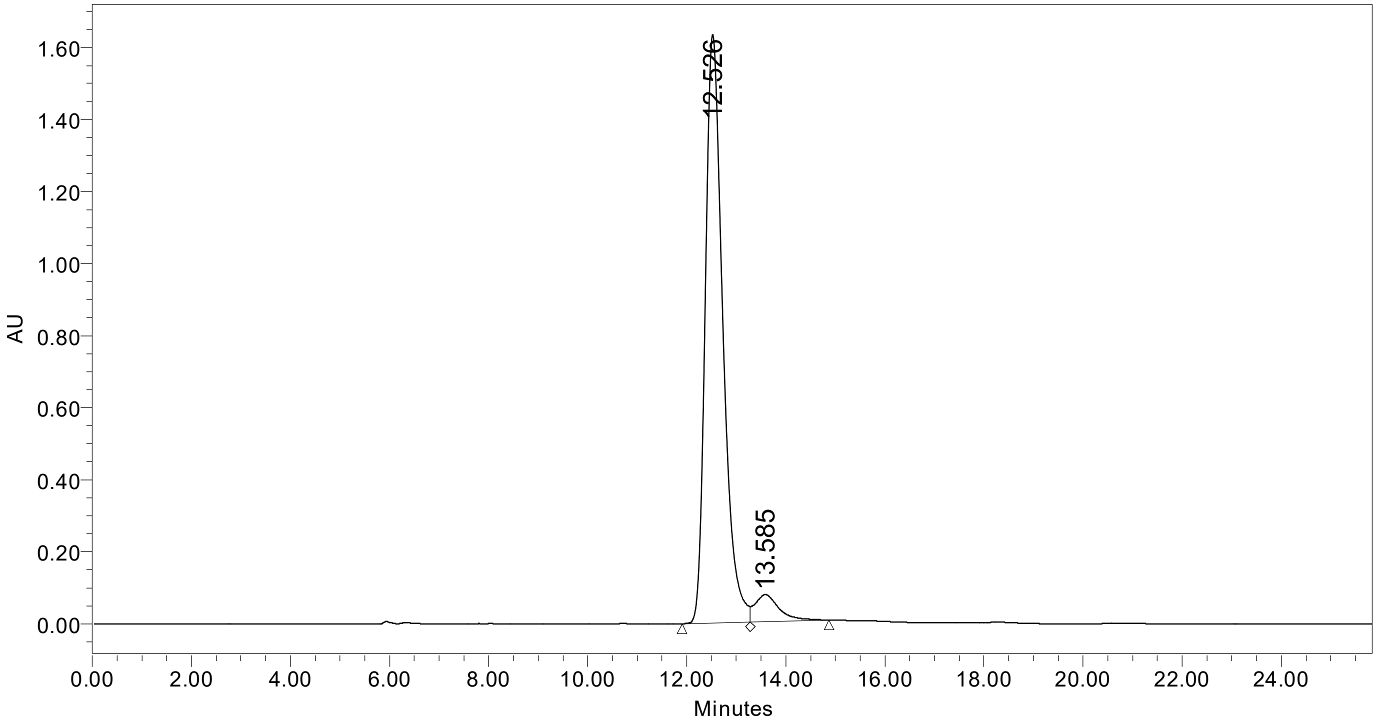

	RT	Area	% Area	Height
1	12.526	40190837	94.04	1634201
2	13.585	2548071	5.96	74790

SAMPLE INFORMATION

Sample Name:	PG96-CRYSTAL	Acquired By:	
Sample Type:	Unknown	Sample Set Name:	
Vial:	1	Acq. Method Set:	
Injection #:	9	Processing Method	
Injection Volume:	10.00 ul	Channel Name:	
Run Time:	100.0 Minutes	Proc. Chnl. Descr.:	
Date Acquired:	10-01-2023 17:30:43 IST		
Date Processed:	10-01-2023 18:08:55 IST		

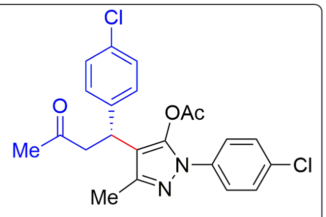

ent-3cc

After single recrystallization

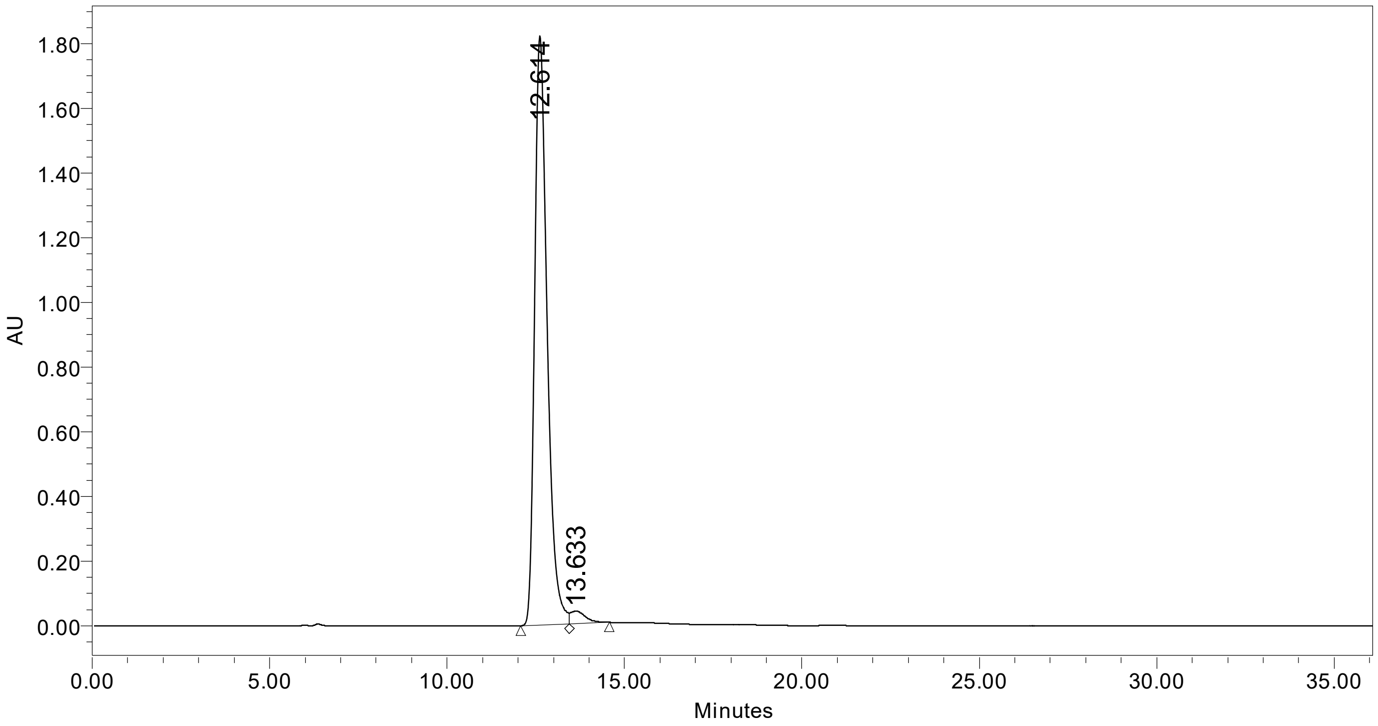

	RT	Area	% Area	Height
1	12.614	44685057	97.54	1822013
2	13.633	1128881	2.46	38441

SAMPLE INFORMATION

Sample Name:	PG-116-ODH	Acquired By:	
Sample Type:	Unknown	Sample Set Name:	
Vial:	1	Acq. Method Set:	
Injection #:	1	Processing Method	
Injection Volume:	10.00 ul	Channel Name:	
Run Time:	100.0 Minutes	Proc. Chnl. Descr.:	
Date Acquired:	04-01-2023 15:55:06 IST		
Date Processed:	04-01-2023 17:38:09 IST		

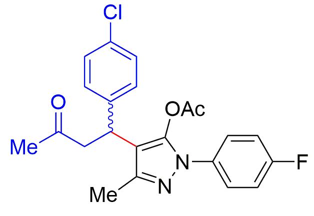

***rac*-3cd**

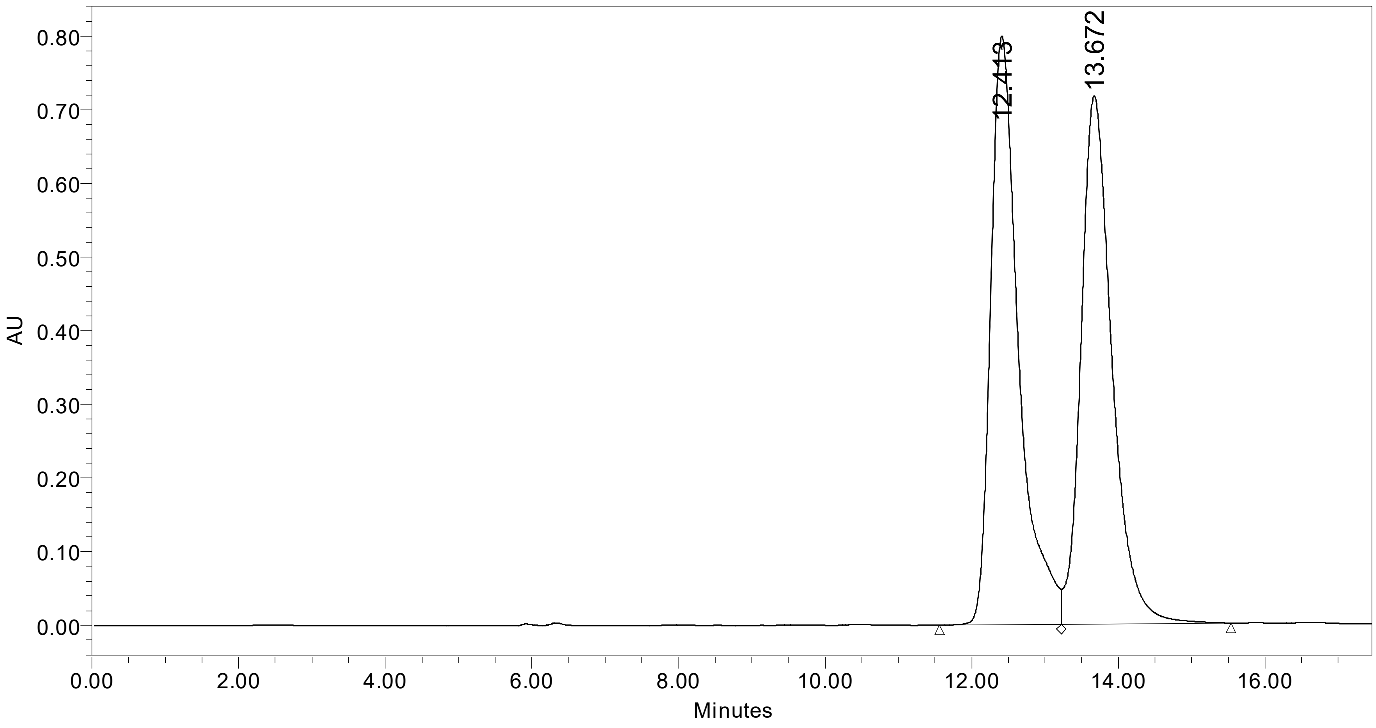

	RT	Area	% Area	Height
1	12.413	21312771	50.31	799331
2	13.672	21048474	49.69	717005

SAMPLE INFORMATION

Sample Name:	PG-114-ODH	Acquired By:	
Sample Type:	Unknown	Sample Set Name:	
Vial:	1	Acq. Method Set:	
Injection #:	2	Processing Method	
Injection Volume:	10.00 ul	Channel Name:	
Run Time:	100.0 Minutes	Proc. Chnl. Descr.:	
Date Acquired:	04-01-2023 16:13:23 IST		
Date Processed:	04-01-2023 17:39:30 IST		

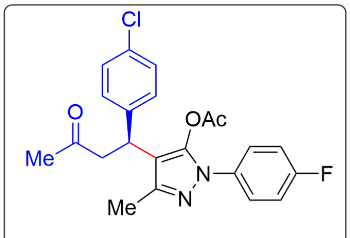

3cd

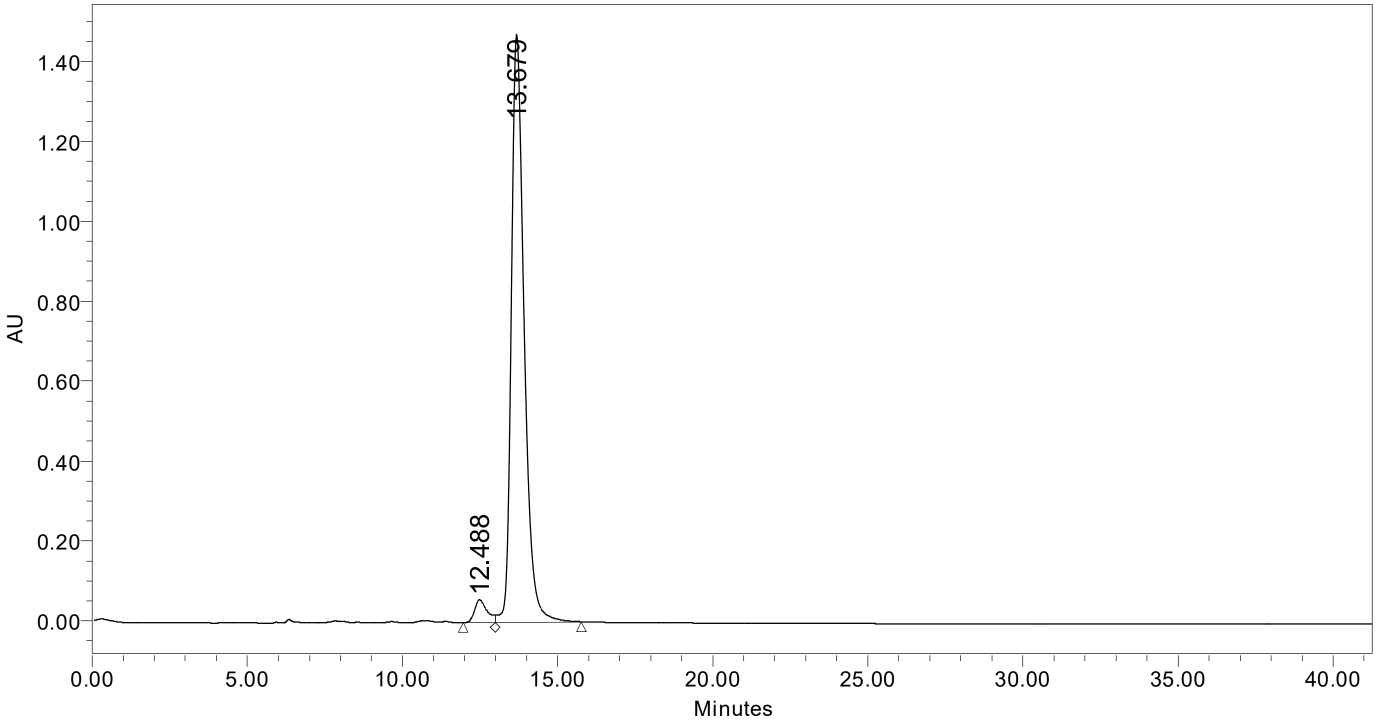

	RT	Area	% Area	Height
1	12.488	1596718	3.64	56505
2	13.679	42309384	96.36	1471717

SAMPLE INFORMATION

Sample Name:	PG-115-ODH	Acquired By:	
Sample Type:	Unknown	Sample Set Name:	
Vial:	1	Acq. Method Set:	
Injection #:	3	Processing Method	
Injection Volume:	10.00 ul	Channel Name:	
Run Time:	100.0 Minutes	Proc. Chnl. Descr.:	
Date Acquired:	04-01-2023 16:55:08 IST		
Date Processed:	04-01-2023 17:40:17 IST		

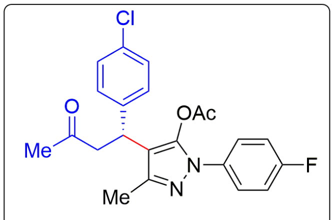

***ent*-3cd**

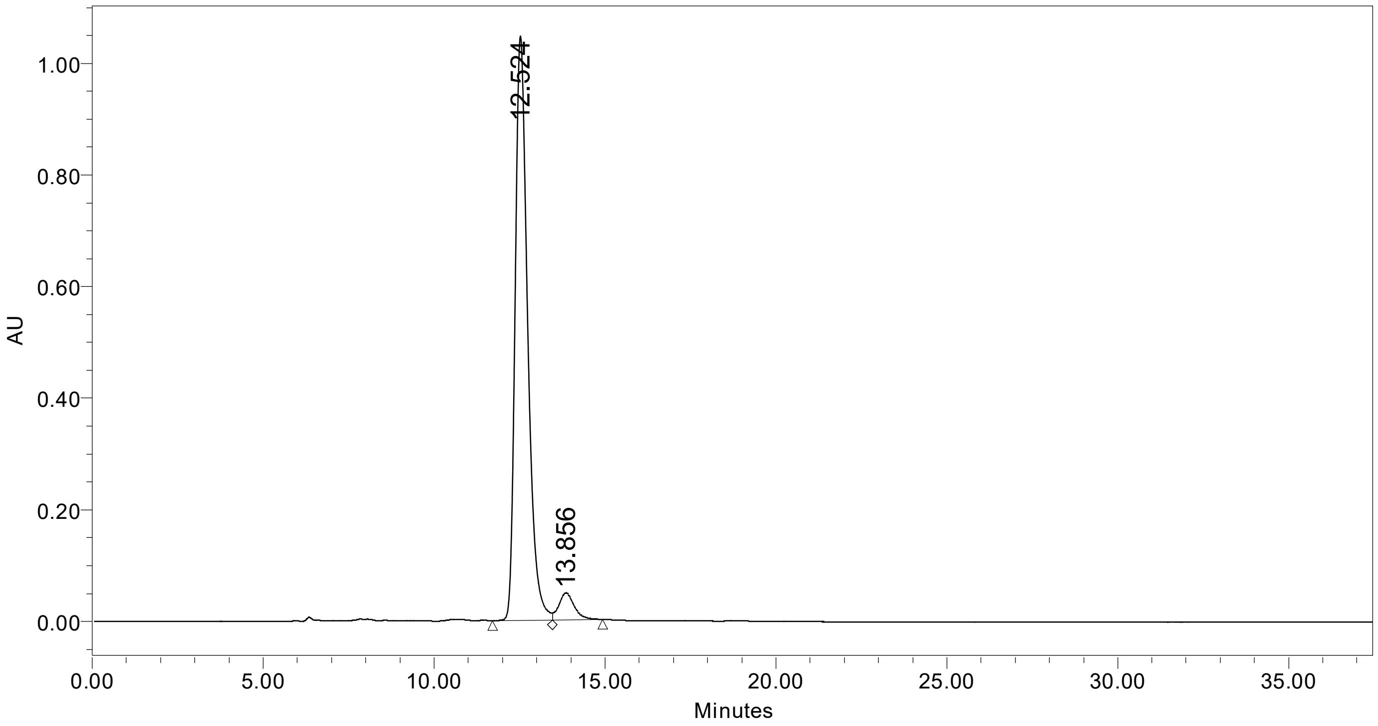

	RT	Area	% Area	Height
1	12.524	26617946	94.53	1047662
2	13.856	1539073	5.47	48556

SAMPLE INFORMATION

Sample Name:	AKD-642	Acquired By:	
Sample Type:	Unknown	Sample Set Name:	
Vial:	1	Acq. Method Set:	
Injection #:	2	Processing Method	
Injection Volume:	10.00 ul	Channel Name:	
Run Time:	100.0 Minutes	Proc. Chnl. Descr.:	
Date Acquired:	29-12-2022 12:13:23 IST		
Date Processed:	29-12-2022 15:05:27 IST		

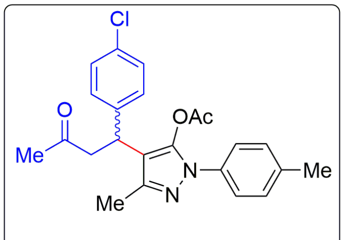

3ce

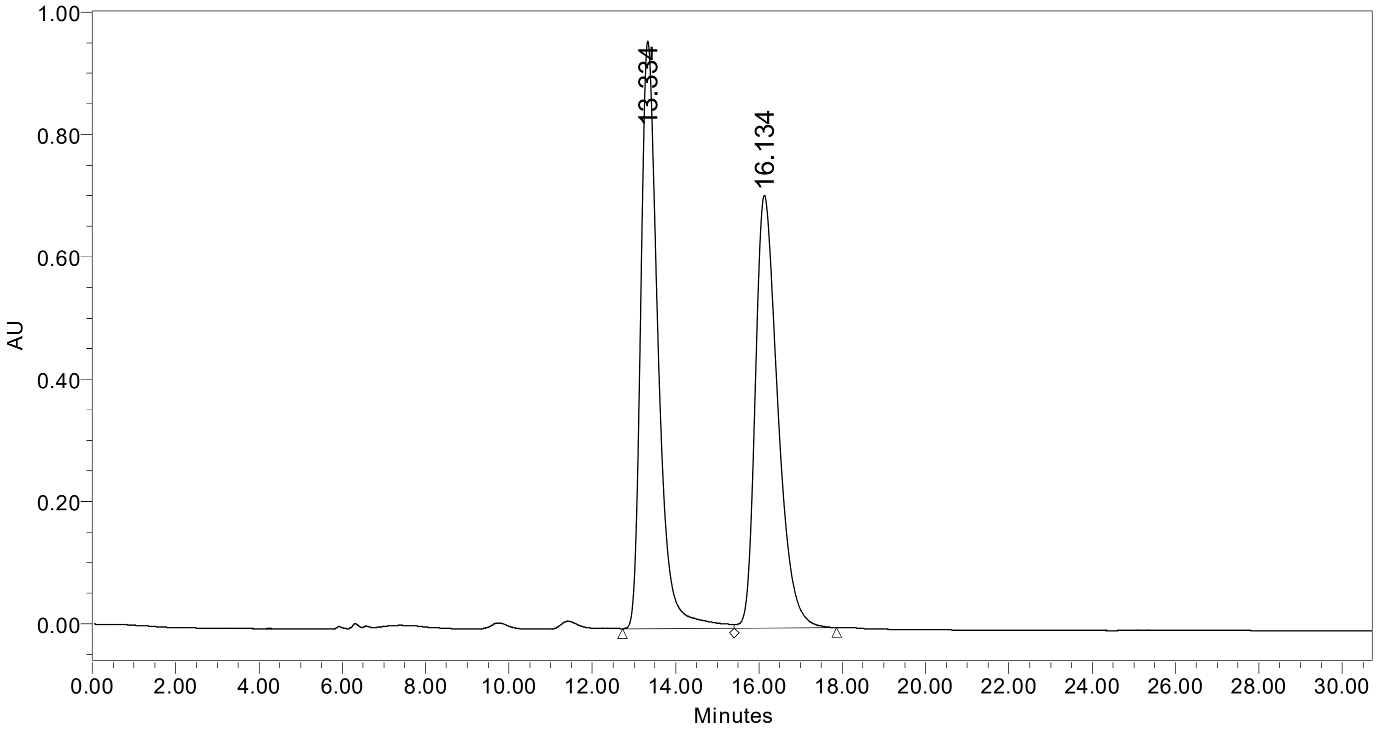

	RT	Area	% Area	Height
1	13.334	27937145	51.35	960801
2	16.134	26464101	48.65	707807

SAMPLE INFORMATION

Sample Name:	PG104-OD-H	Acquired By:	
Sample Type:	Unknown	Sample Set Name:	
Vial:	1	Acq. Method Set:	
Injection #:	4	Processing Method	
Injection Volume:	10.00 ul	Channel Name:	
Run Time:	100.0 Minutes	Proc. Chnl. Descr.:	
Date Acquired:	29-12-2022 13:14:55 IST		
Date Processed:	29-12-2022 15:04:03 IST		

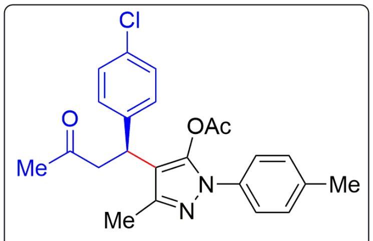

3ce

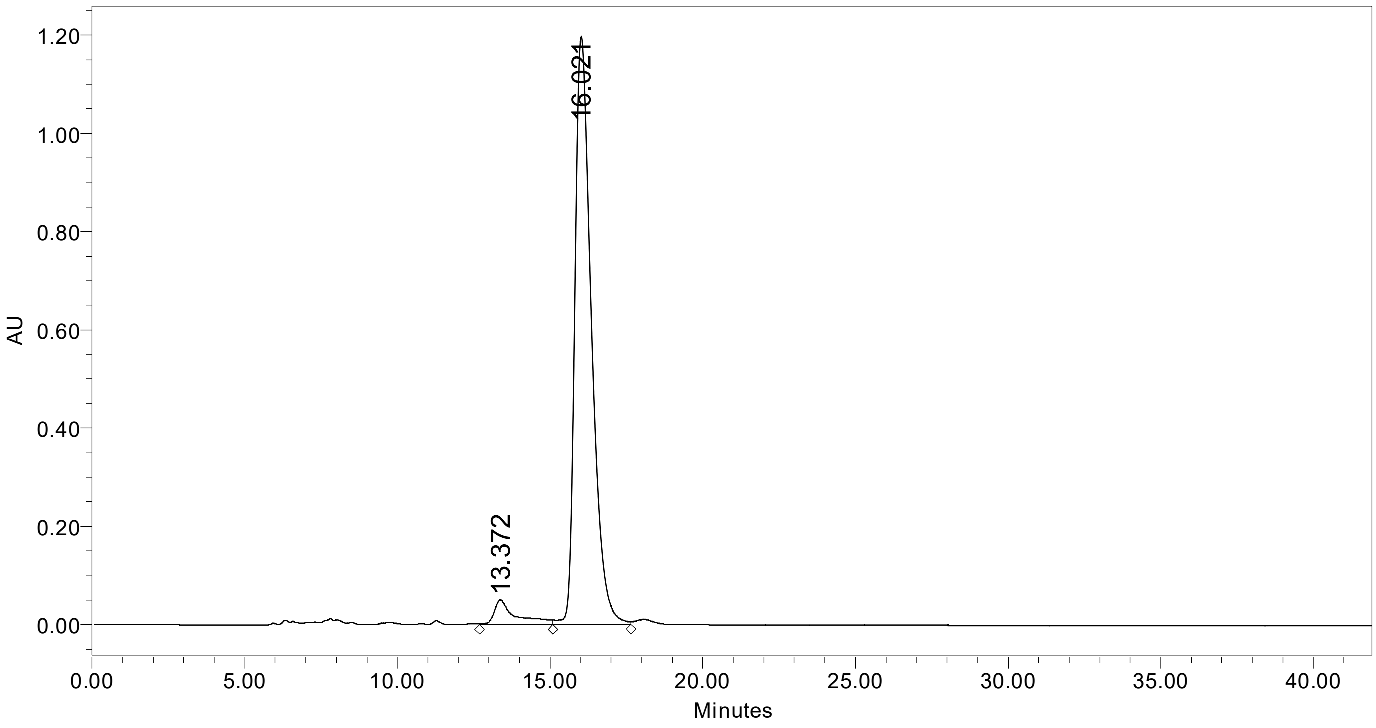

	RT	Area	% Area	Height
1	13.372	2507989	5.25	50485
2	16.021	45227068	94.75	1197961

SAMPLE INFORMATION

Sample Name:	PG105-OD-H	Acquired By:	
Sample Type:	Unknown	Sample Set Name:	
Vial:	1	Acq. Method Set:	
Injection #:	5	Processing Method	
Injection Volume:	10.00 ul	Channel Name:	
Run Time:	100.0 Minutes	Proc. Chnl. Descr.:	
Date Acquired:	29-12-2022 13:57:27 IST		
Date Processed:	29-12-2022 15:00:02 IST		

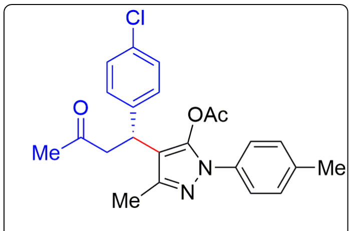

ent-3ce

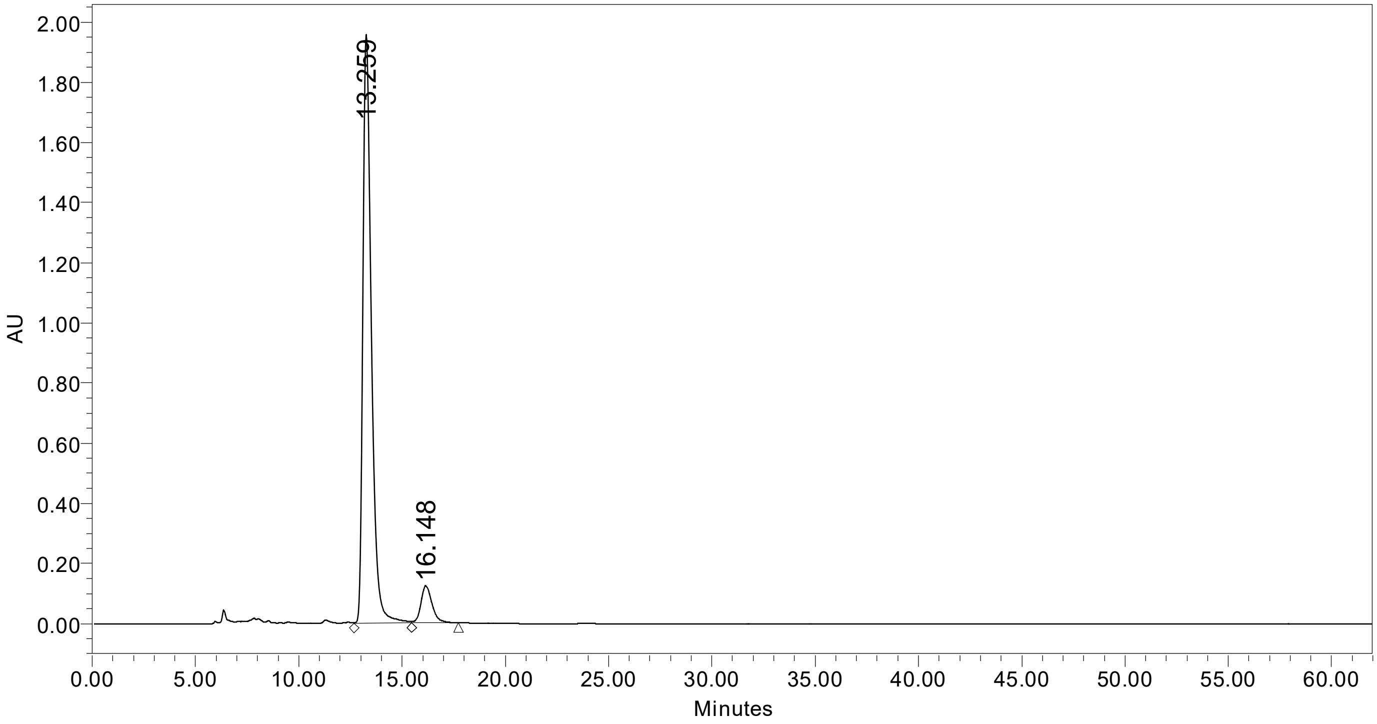

	RT	Area	% Area	Height
1	13.259	56063266	92.33	1957894
2	16.148	4660118	7.67	123711

SAMPLE INFORMATION

Sample Name:	AKD-646	Acquired By:	
Sample Type:	Unknown	Sample Set Name:	
Vial:	1	Acq. Method Set:	
Injection #:	6	Processing Method	
Injection Volume:	10.00 ul	Channel Name:	
Run Time:	100.0 Minutes	Proc. Chnl. Descr.:	
Date Acquired:	17-01-2023 16:04:51 IST		
Date Processed:	17-01-2023 16:36:04 IST		

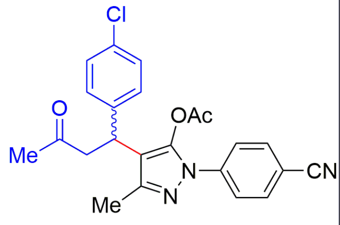

***rac*-3cf**

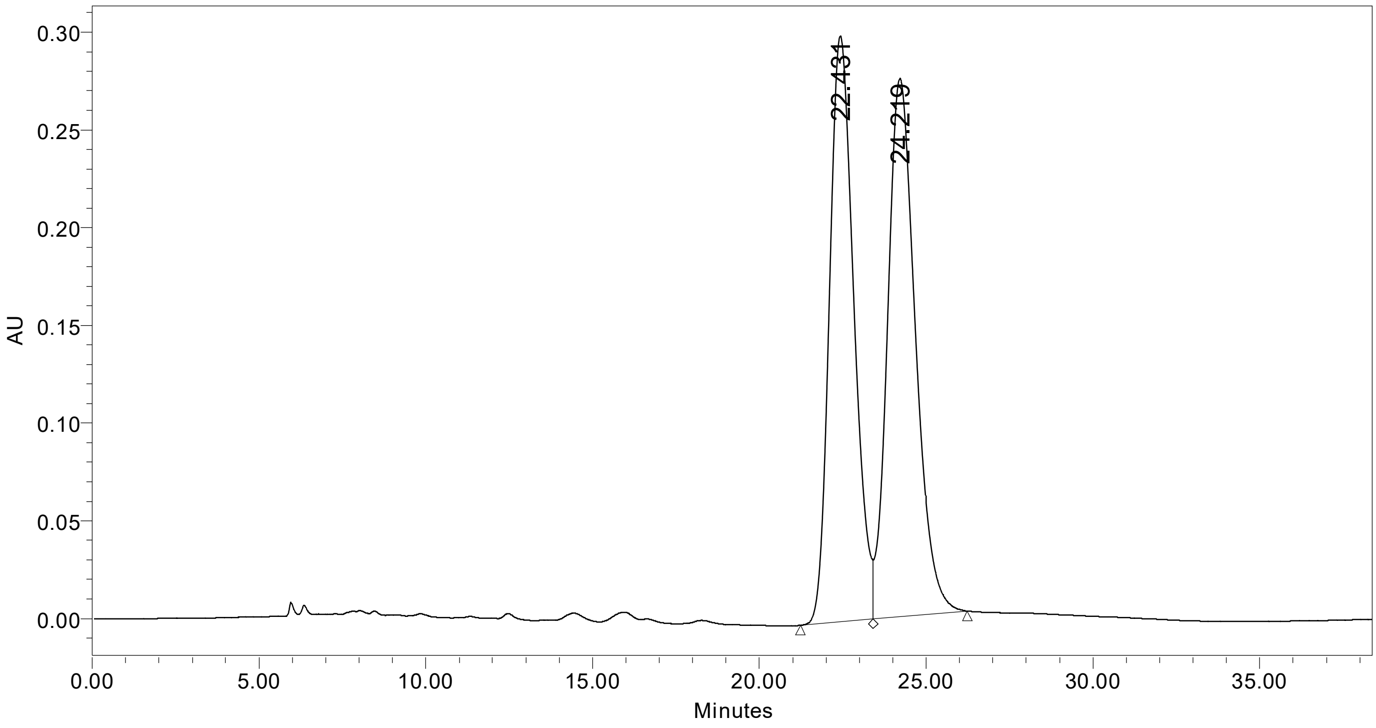

	RT	Area	% Area	Height
1	22.431	15282469	48.83	299864
2	24.219	16016144	51.17	275454

SAMPLE INFORMATION

Sample Name:	PG129	Acquired By:	
Sample Type:	Unknown	Sample Set Name:	
Vial:	1	Acq. Method Set:	
Injection #:	5	Processing Method	
Injection Volume:	10.00 ul	Channel Name:	
Run Time:	100.0 Minutes	Proc. Chnl. Descr.:	
Date Acquired:	17-01-2023 15:31:39 IST		
Date Processed:	17-01-2023 16:44:49 IST		

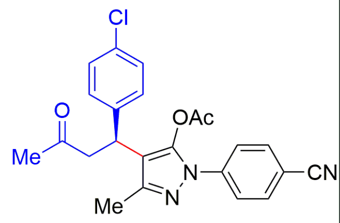

3cf

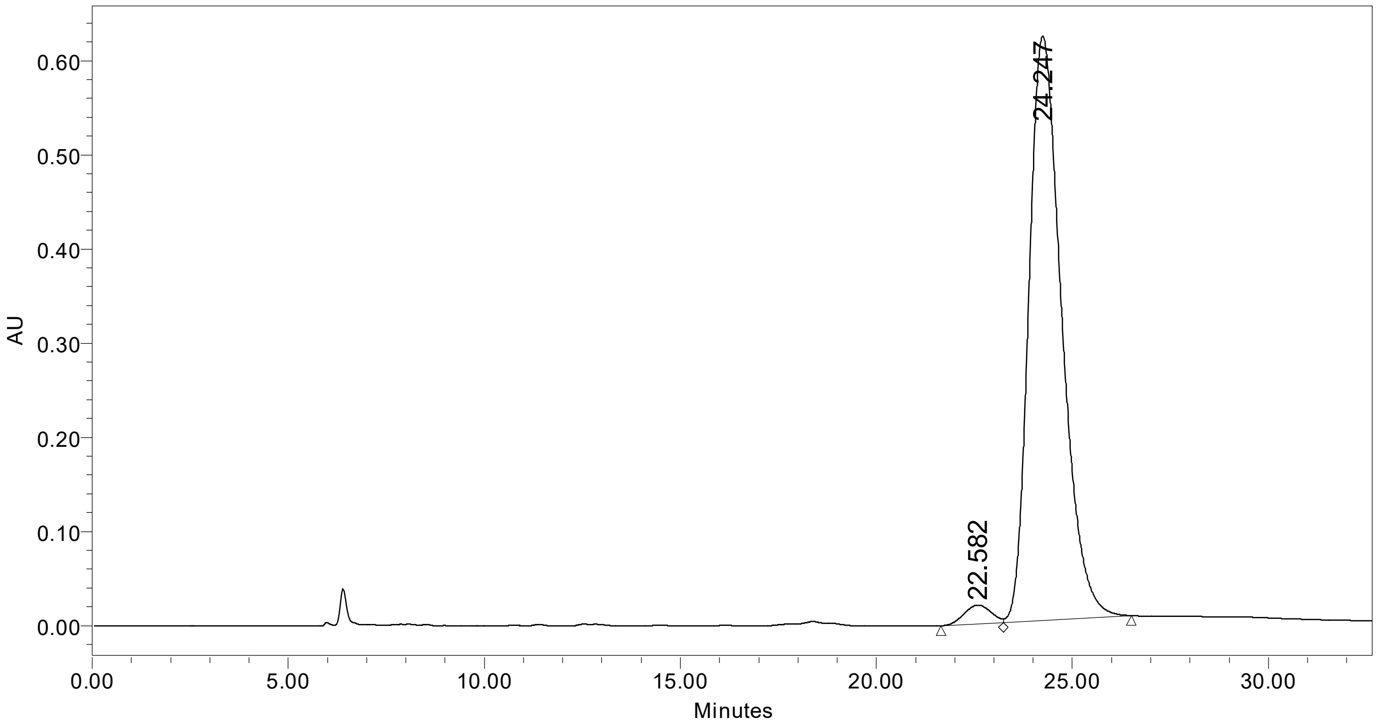

	RT	Area	% Area	Height
1	22.582	948660	2.57	19981
2	24.247	35945660	97.43	621059

SAMPLE INFORMATION

Sample Name:	PG-112-ODH	Acquired By:	System
Sample Type:	Unknown	Sample Set Name:	
Vial:	1	Acq. Method Set:	
Injection #:	1	Processing Method	
Injection Volume:	10.00 ul	Channel Name:	
Run Time:	100.0 Minutes	Proc. Chnl. Descr.:	
Date Acquired:	02-01-2023 15:41:38 IST		
Date Processed:	05-01-2023 18:18:21 IST		

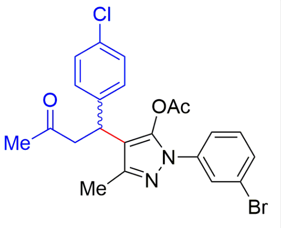

***rac*-3cg**

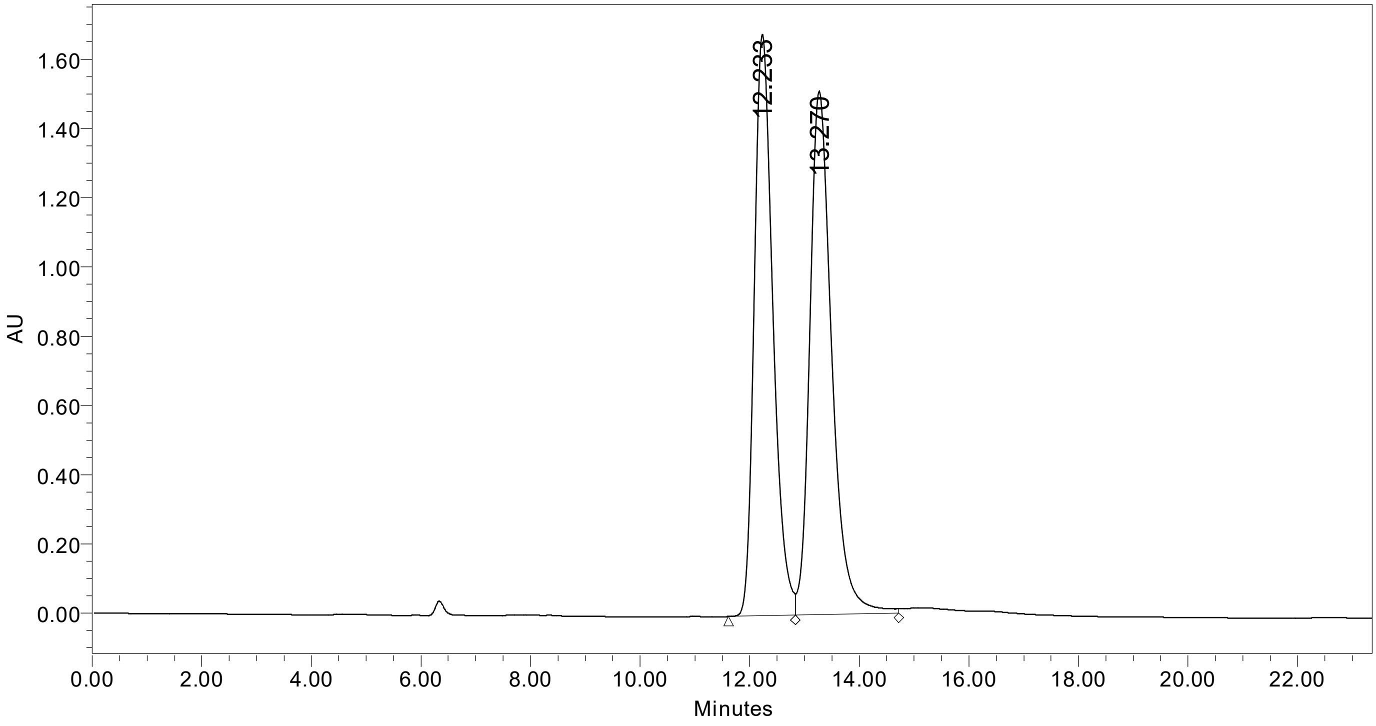

	RT	Area	% Area	Height
1	12.233	39764720	48.59	1678994
2	13.270	42073595	51.41	1511126

SAMPLE INFORMATION

Sample Name:	PG-109-ODH	Acquired By:	
Sample Type:	Unknown	Sample Set Name:	
Vial:	1	Acq. Method Set:	
Injection #:	2	Processing Method	
Injection Volume:	10.00 ul	Channel Name:	
Run Time:	100.0 Minutes	Proc. Chnl. Descr.:	
Date Acquired:	02-01-2023 16:05:20 IST		
Date Processed:	05-01-2023 18:19:06 IST		

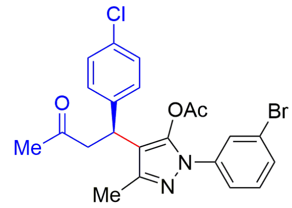

3cg

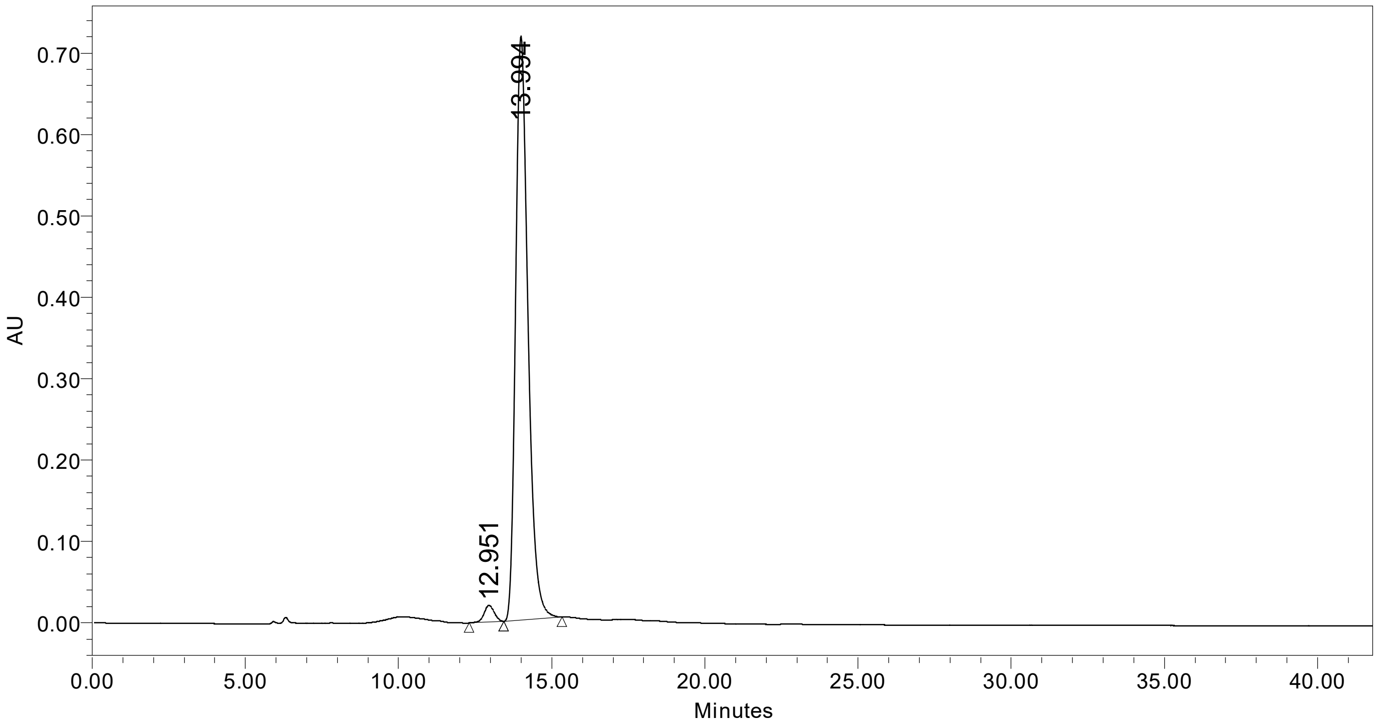

	RT	Area	% Area	Height
1	12.951	482089	2.33	20645
2	13.994	20175078	97.67	717992

SAMPLE INFORMATION

Sample Name:	PG-110-ODH	Acquired By:	
Sample Type:	Unknown	Sample Set Name:	
Vial:	1	Acq. Method Set:	
Injection #:	4	Processing Method	
Injection Volume:	10.00 ul	Channel Name:	
Run Time:	100.0 Minutes	Proc. Chnl. Descr.:	
Date Acquired:	02-01-2023 17:20:18 IST		
Date Processed:	05-01-2023 18:19:59 IST		

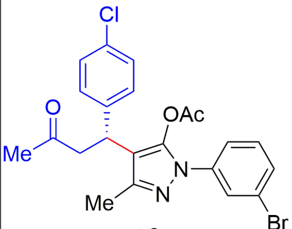

ent-3cg

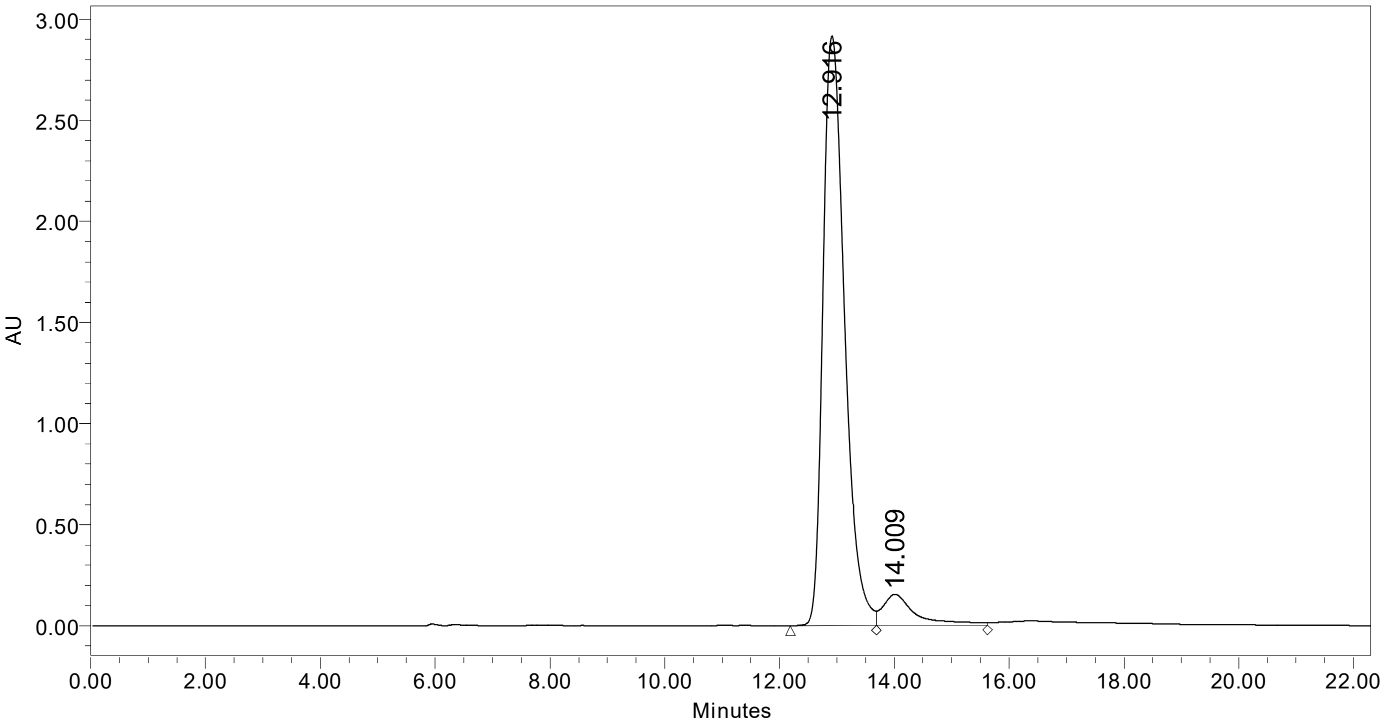

	RT	Area	% Area	Height
1	12.916	76334899	92.54	2916527
2	14.009	6152202	7.46	153622

SAMPLE INFORMATION

Sample Name:	PG-117	Acquired By:	
Sample Type:	Unknown	Sample Set Name:	
Vial:	1	Acq. Method Set:	
Injection #:	1	Processing Method	
Injection Volume:	10.00 ul	Channel Name:	
Run Time:	100.0 Minutes	Proc. Chnl. Descr.:	
Date Acquired:	13-01-2023 11:36:38 IST		
Date Processed:	13-01-2023 12:05:46 IST		

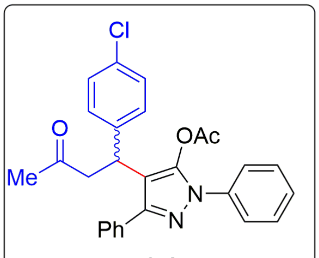

3ch

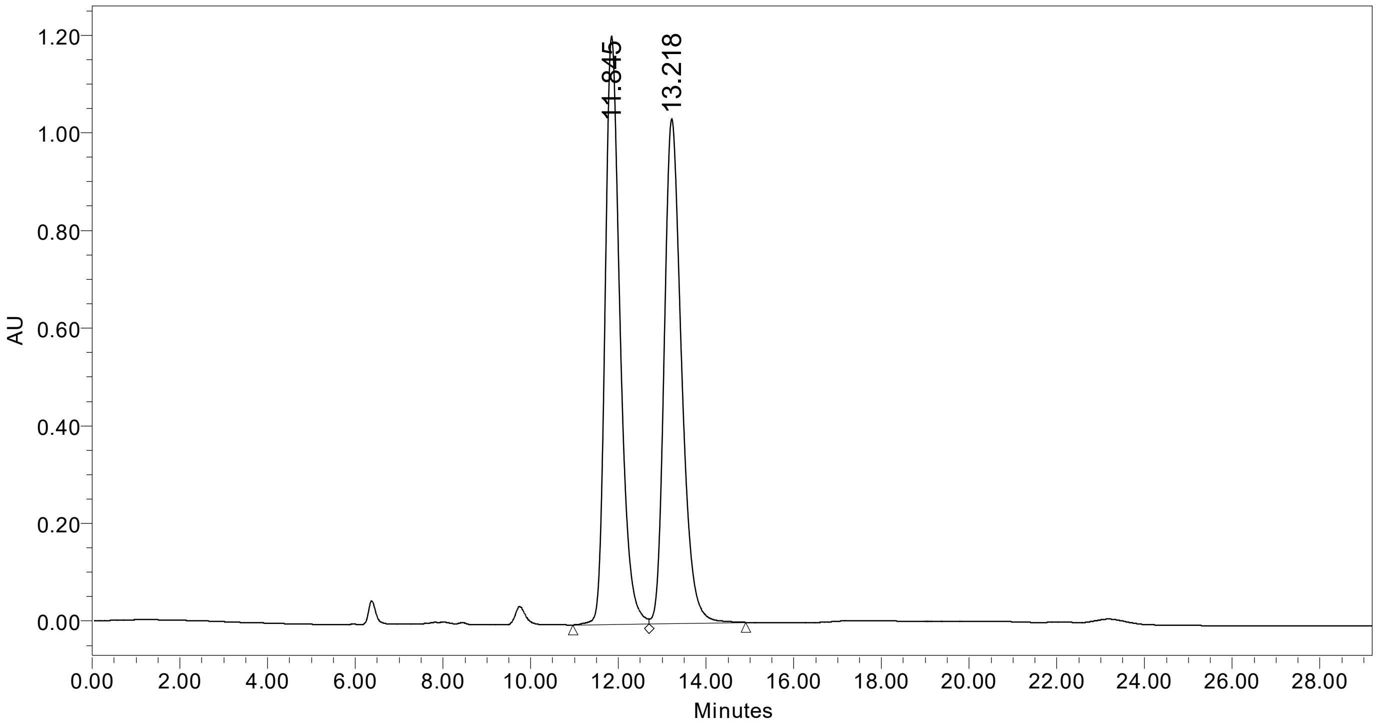

	RT	Area	% Area	Height
1	11.845	28733496	50.30	1205727
2	13.218	28392079	49.70	1034446

SAMPLE INFORMATION

Sample Name:	PG-125	Acquired By:	
Sample Type:	Unknown	Sample Set Name:	
Vial:	1	Acq. Method Set:	
Injection #:	2	Processing Method	
Injection Volume:	10.00 ul	Channel Name:	
Run Time:	100.0 Minutes	Proc. Chnl. Descr.:	
Date Acquired:	13-01-2023 12:08:09 IST		
Date Processed:	16-03-2023 18:37:11 IST		

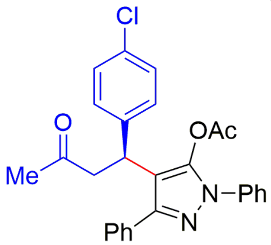

3ch

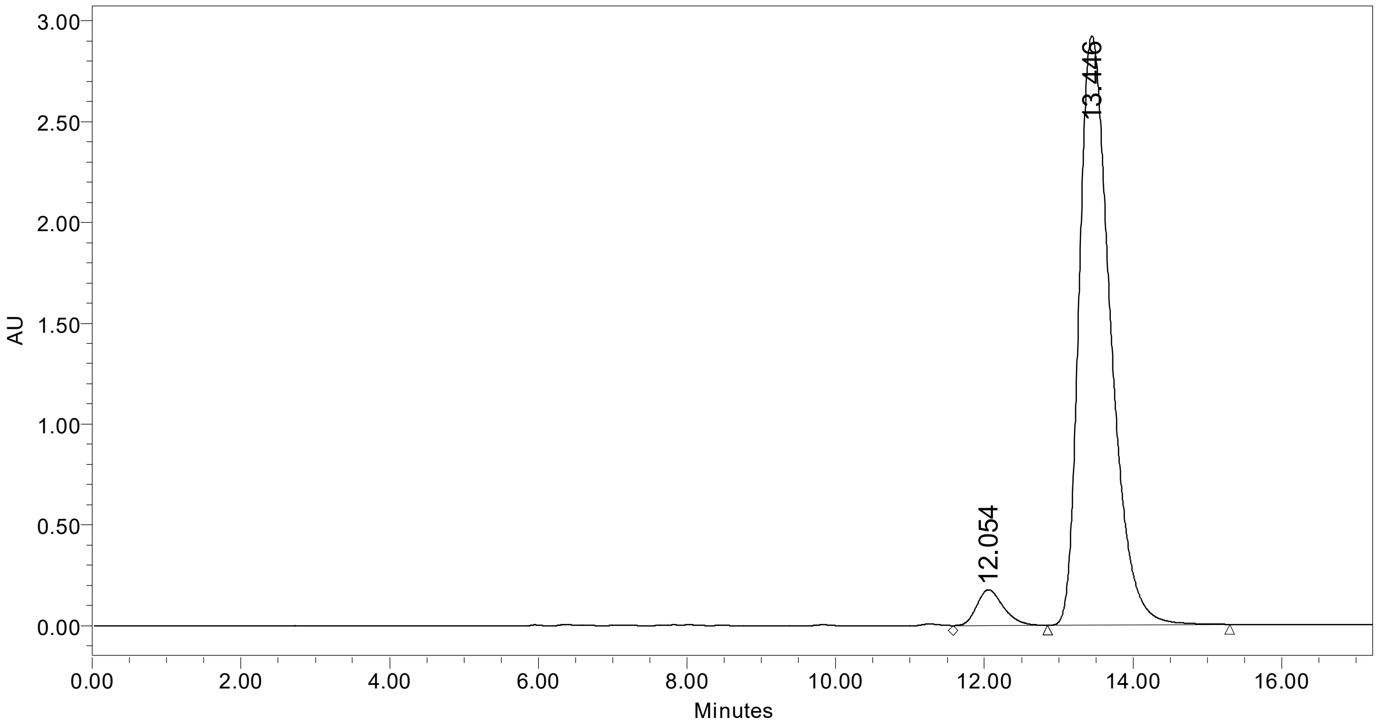

	RT	Area	% Area	Height
1	12.054	4438579	4.95	178103
2	13.446	85167103	95.05	2921835

SAMPLE INFORMATION

Sample Name:	PG126	Acquired By:	
Sample Type:	Unknown	Sample Set Name:	
Vial:	1	Acq. Method Set:	
Injection #:	1	Processing Method	
Injection Volume:	10.00 ul	Channel Name:	
Run Time:	100.0 Minutes	Proc. Chnl. Descr.:	
Date Acquired:	18-01-2023 17:14:50 IST		
Date Processed:	16-03-2023 18:47:49 IST		

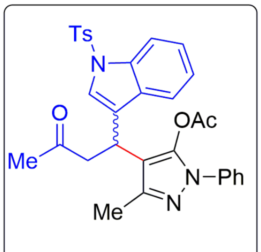

***rac*-3ma**

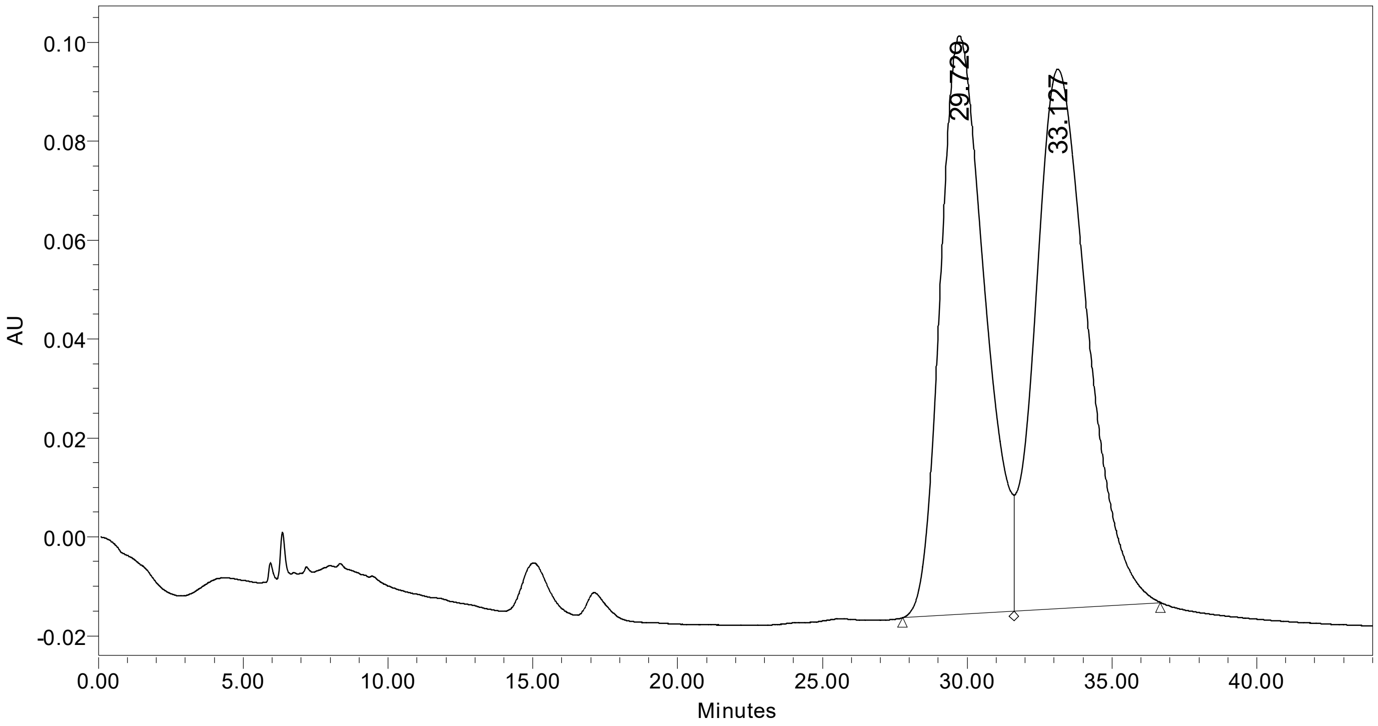

	RT	Area	% Area	Height
1	29.729	12712917	48.09	116985
2	33.127	13720371	51.91	109075

SAMPLE INFORMATION

Sample Name:	PG131	Acquired By:	
Sample Type:	Unknown	Sample Set Name:	
Vial:	1	Acq. Method Set:	
Injection #:	1	Processing Method	
Injection Volume:	10.00 ul	Channel Name:	
Run Time:	100.0 Minutes	Proc. Chnl. Descr.:	
Date Acquired:	19-01-2023 15:04:37 IST		
Date Processed:	16-03-2023 18:50:57 IST		

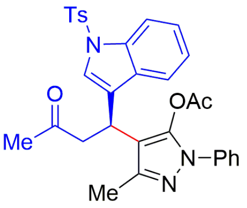

3ma

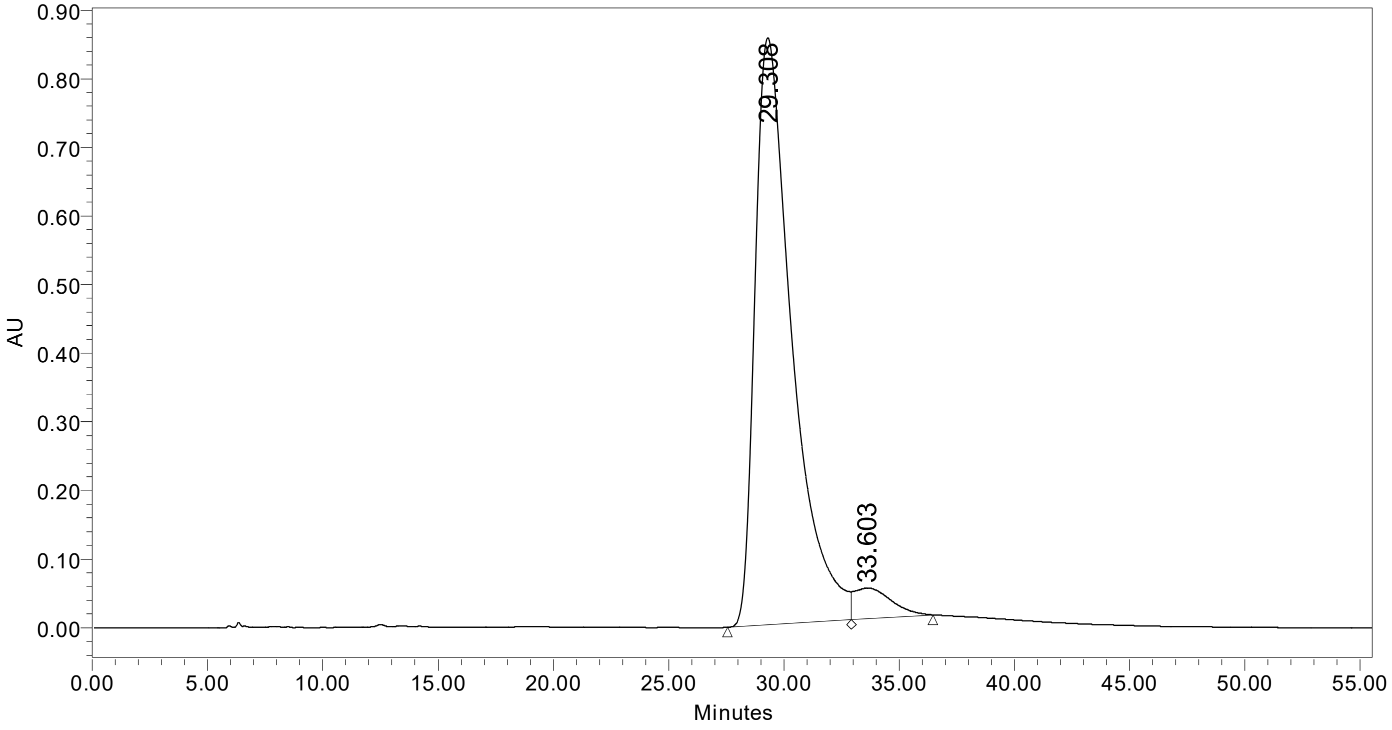

	RT	Area	% Area	Height
1	29.308	95690688	95.02	855863
2	33.603	5012813	4.98	44800

SAMPLE INFORMATION

Sample Name:	PG132	Acquired By:	
Sample Type:	Unknown	Sample Set Name:	
Vial:	1	Acq. Method Set:	
Injection #:	2	Processing Method	
Injection Volume:	10.00 ul	Channel Name:	
Run Time:	100.0 Minutes	Proc. Chnl. Descr.:	
Date Acquired:	18-01-2023 17:59:13 IST		
Date Processed:	16-03-2023 18:48:53 IST		

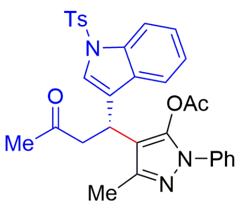

***ent*-3ma**

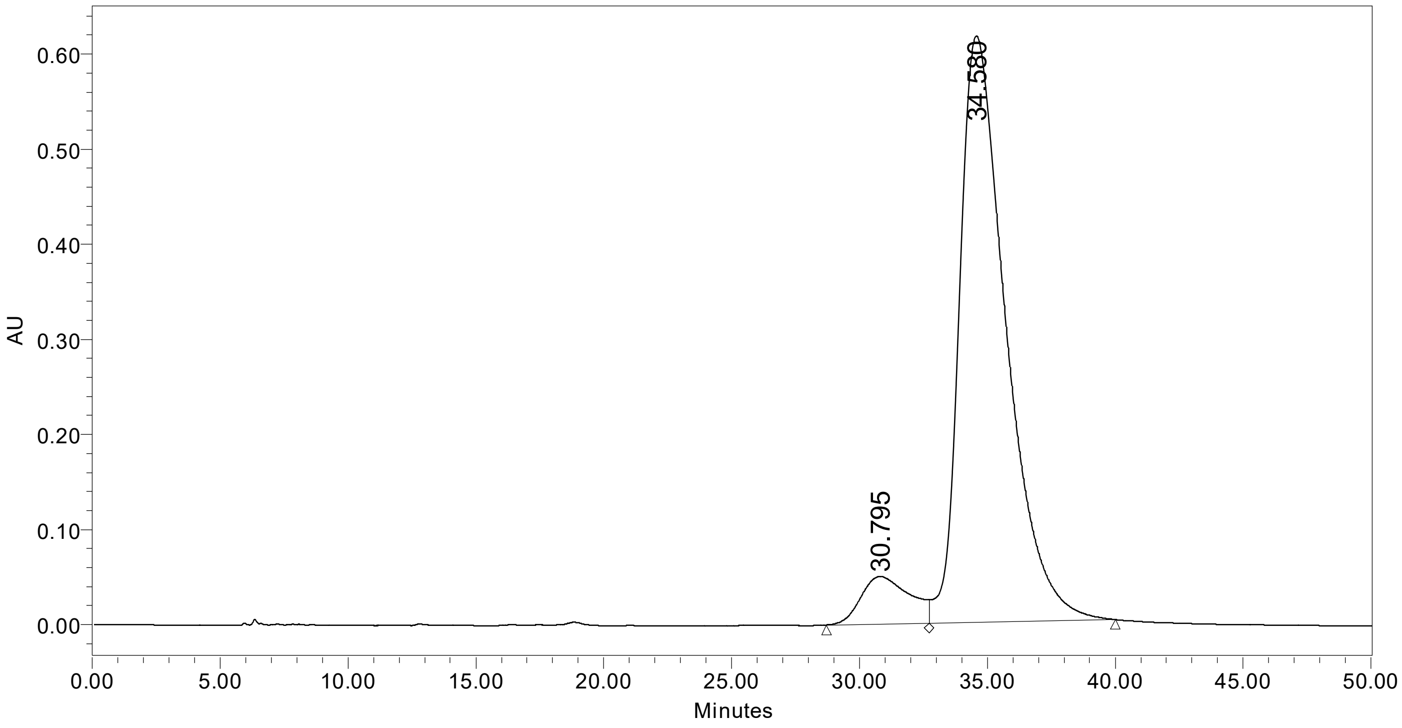

	RT	Area	% Area	Height
1	30.795	6683102	7.91	49908
2	34.580	77843218	92.09	616642

Empower™ 3 SOFTWARE

RC2638PDF

SAMPLE INFORMATION

Sample Name:	PG123	Acquired By:	
Sample Type:	Unknown	Sample Set Name:	
Vial:	1	Acq. Method Set:	
Injection #:	7	Processing Method	
Injection Volume:	10.00 ul	Channel Name:	
Run Time:	100.0 Minutes	Proc. Chnl. Descr.:	
Date Acquired:	13-01-2023 14:33:05 IST		
Date Processed:	16-01-2023 11:56:25 IST		

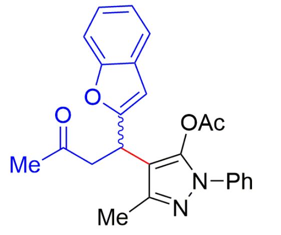

***rac*-3na**

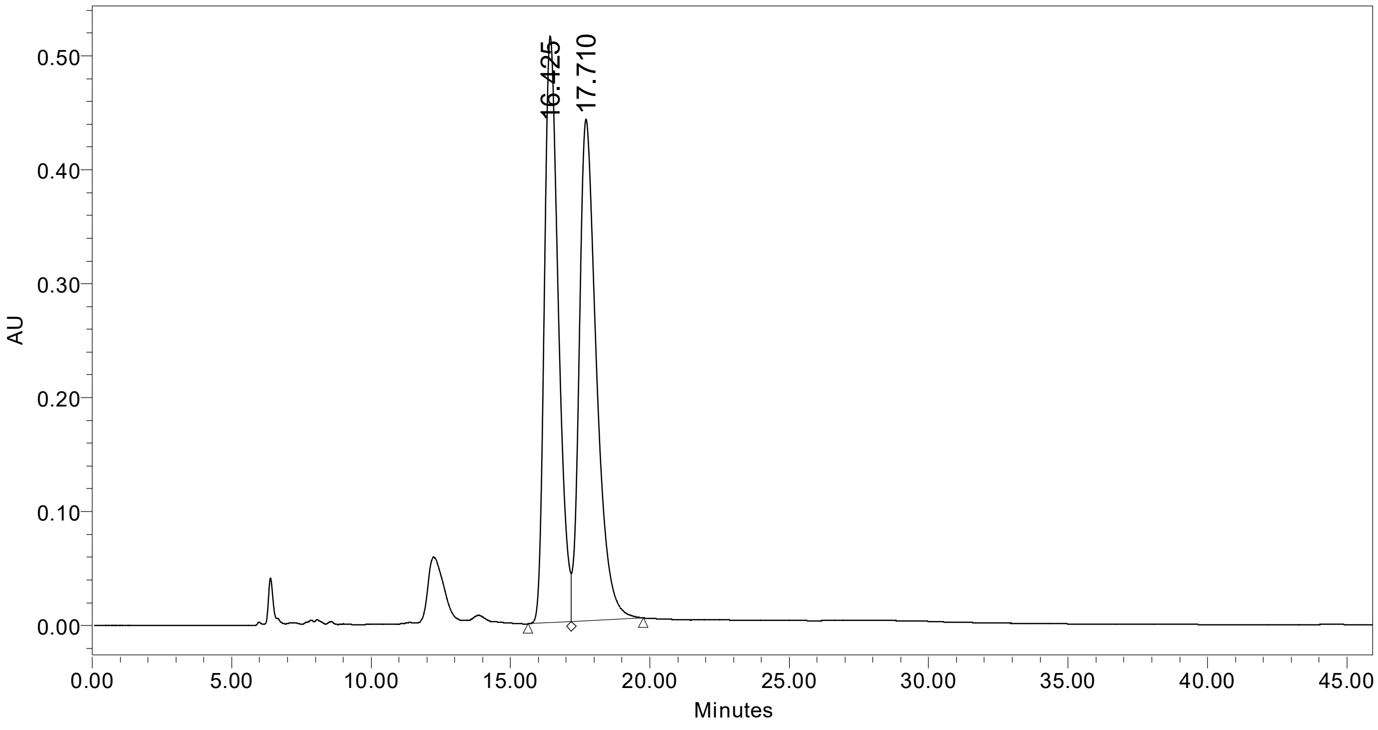

	RT	Area	% Area	Height
1	16.425	18060444	48.79	515128
2	17.710	18958230	51.21	440365

SAMPLE INFORMATION

Sample Name:	PG127	Acquired By:	
Sample Type:	Unknown	Sample Set Name:	
Vial:	1	Acq. Method Set:	
Injection #:	1	Processing Method	
Injection Volume:	10.00 ul	Channel Name:	
Run Time:	100.0 Minutes	Proc. Chnl. Descr.:	
Date Acquired:	16-01-2023 11:56:00 IST		
Date Processed:	16-03-2023 18:41:01 IST		

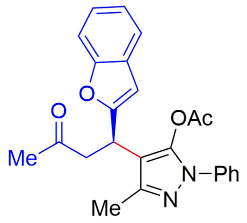

3na

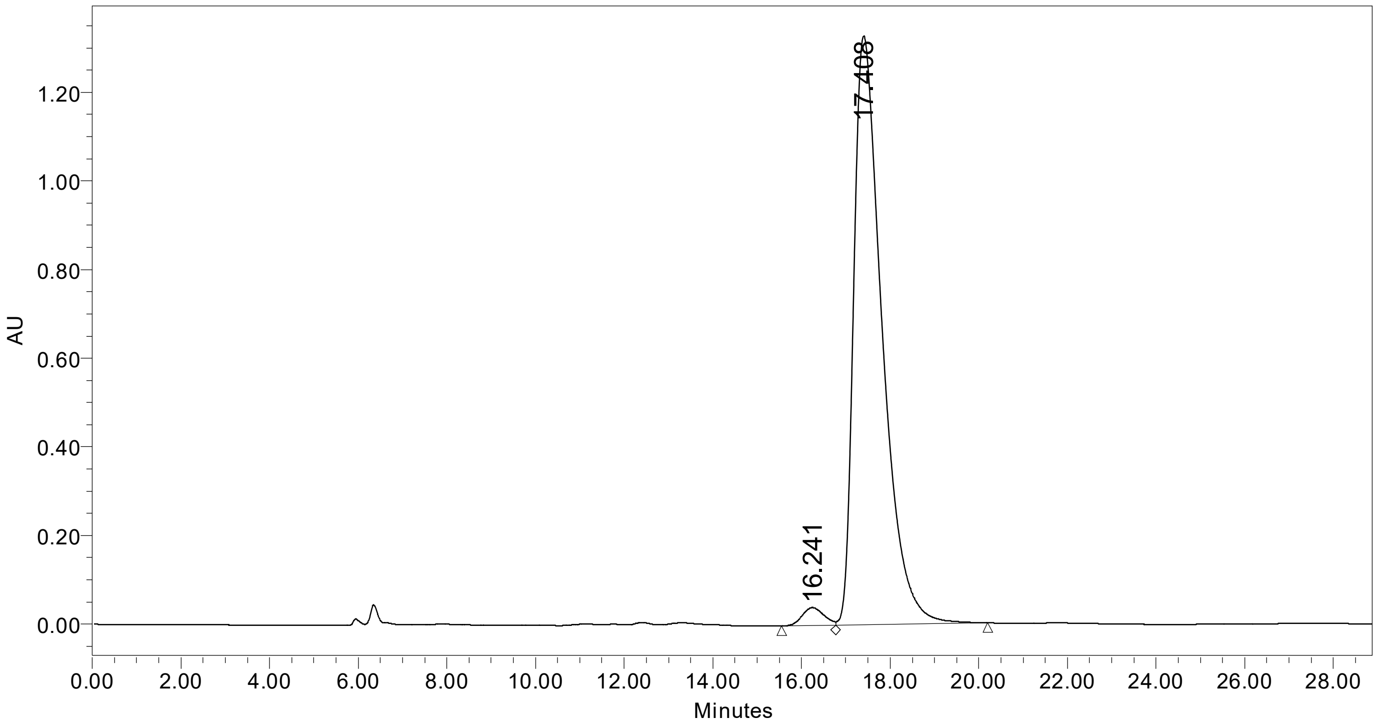

	RT	Area	% Area	Height
1	16.241	1374562	2.28	40318
2	17.408	59041896	97.72	1328807

SAMPLE INFORMATION

Sample Name:	PG128	Acquired By:	
Sample Type:	Unknown	Sample Set Name:	
Vial:	1	Acq. Method Set:	
Injection #:	1	Processing Method	
Injection Volume:	10.00 ul	Channel Name:	
Run Time:	100.0 Minutes	Proc. Chnl. Descr.:	
Date Acquired:	13-01-2023 18:41:08 IST		
Date Processed:	16-01-2023 11:57:27 IST		

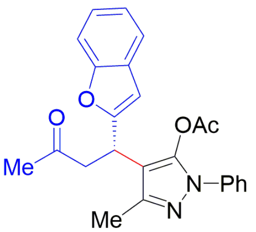

***ent*-3na**

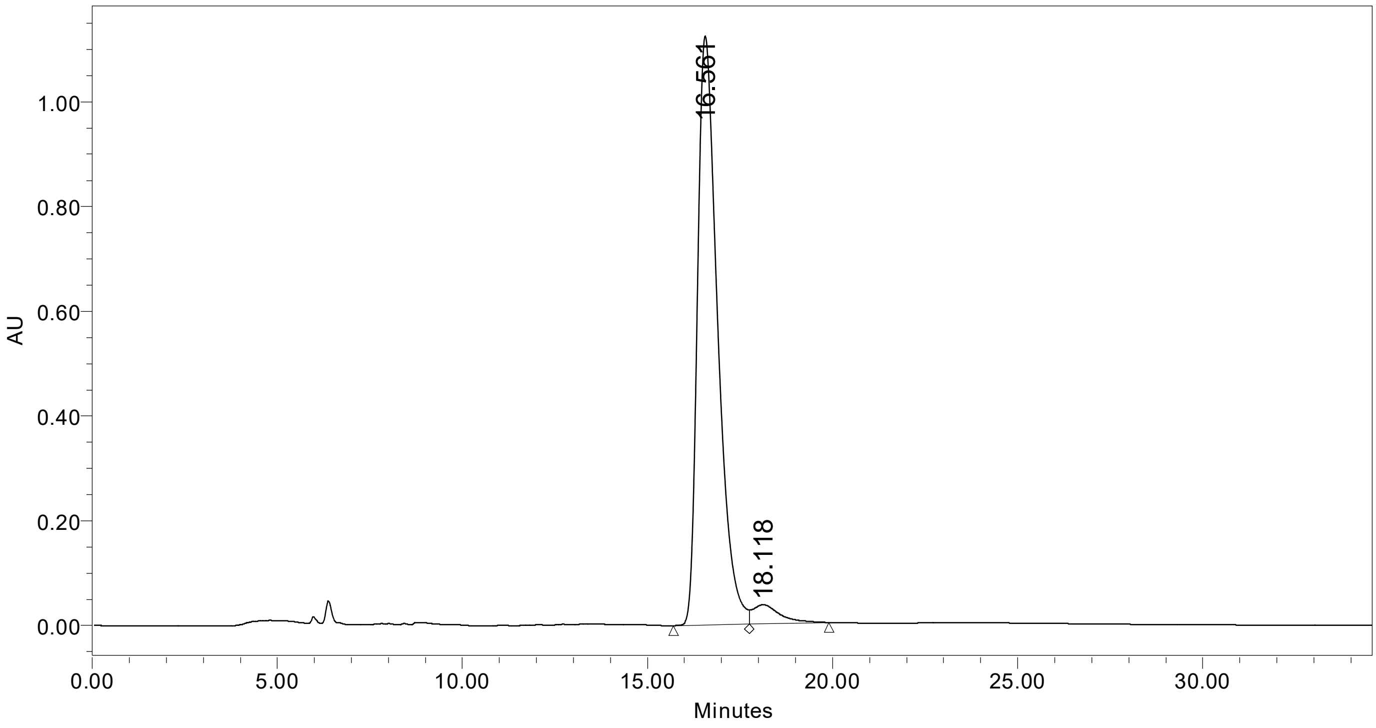

	RT	Area	% Area	Height
1	16.561	42604173	95.76	1125024
2	18.118	1884169	4.24	36487

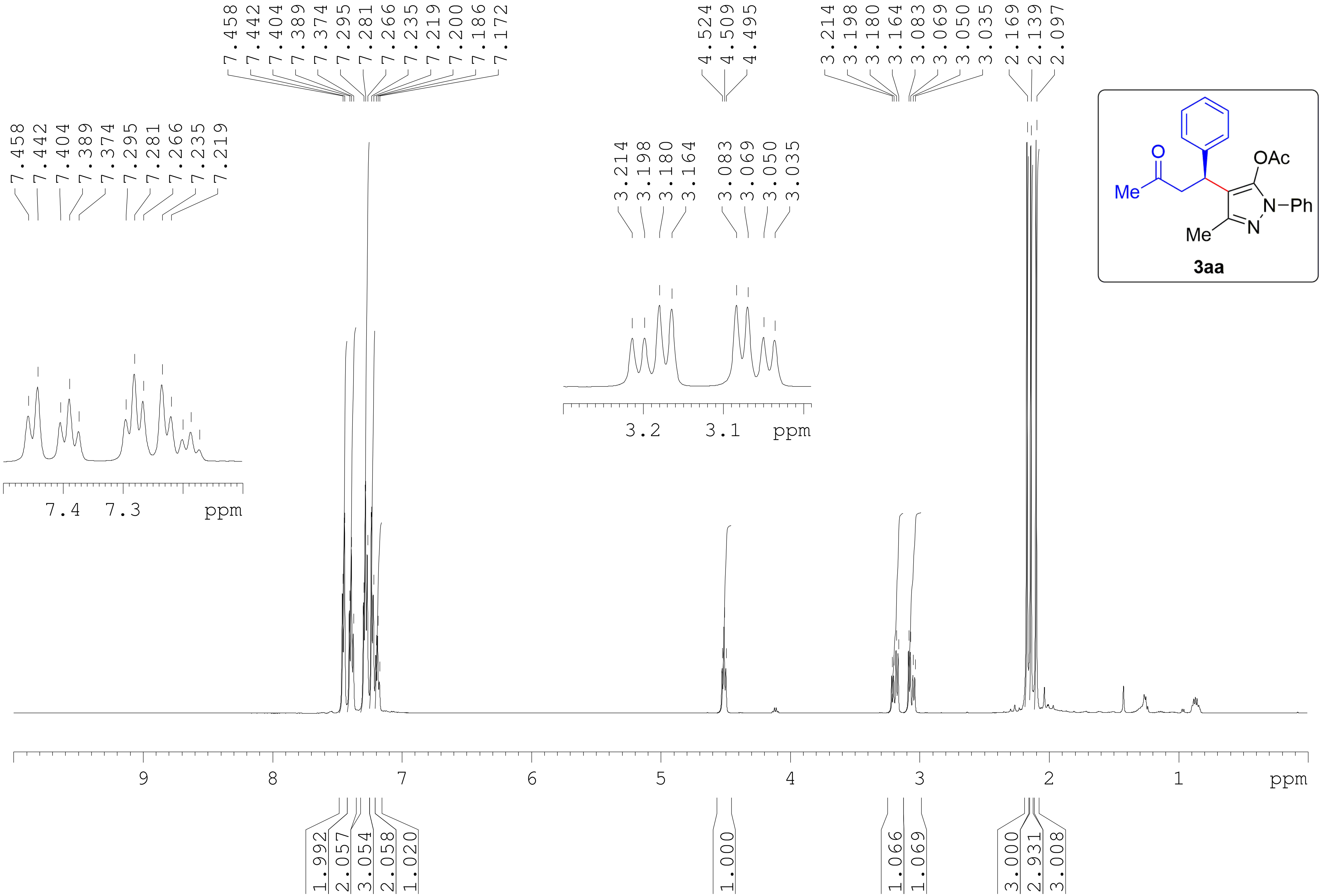
7.458
7.442
7.404
7.389
7.374
7.295
7.281
7.266
7.235
7.219
7.200
7.186
7.172
4.524
4.509
4.495
3.214
3.198
3.180
3.164
3.083
3.069
3.050
3.035
2.169
2.139
2.097
3.2
3.1
ppm
7.4
7.3
ppm
9
8
7
6
5
4
3
2
1
ppm
1.992
2.057
3.054
2.058
1.020
1.000
1.066
1.069
3.000
2.931
3.008
O
Me
OAc
N–Ph
N
Me
3aa

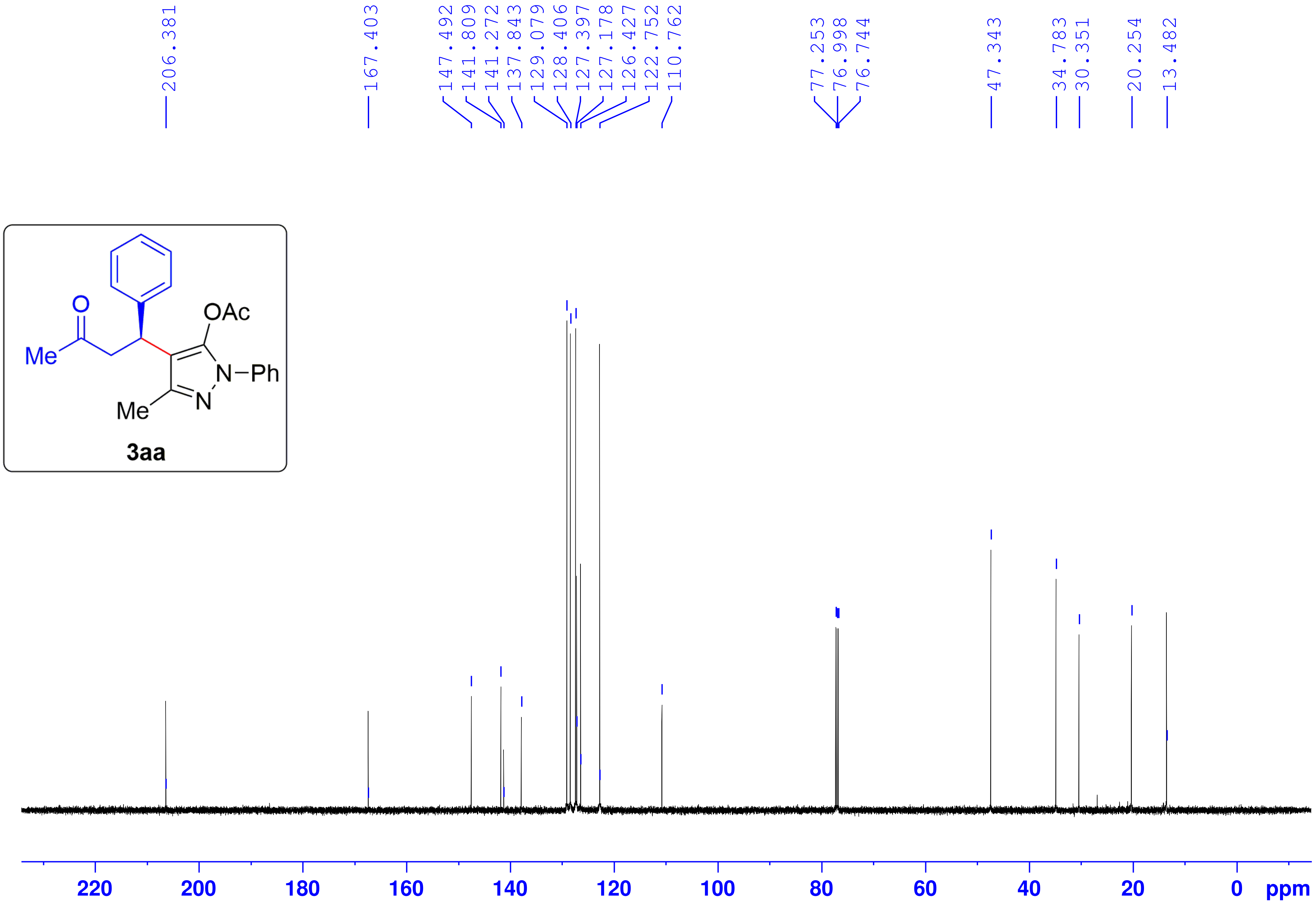
206.381
167.403
147.492
141.809
141.272
137.843
129.079
128.406
127.397
127.178
126.427
122.752
110.762
77.253
76.998
76.744
47.343
34.783
30.351
20.254
13.482
O
Me
OAc
N−Ph
N
Me
3aa
220
200
180
160
140
120
100
80
60
40
20
0
ppm

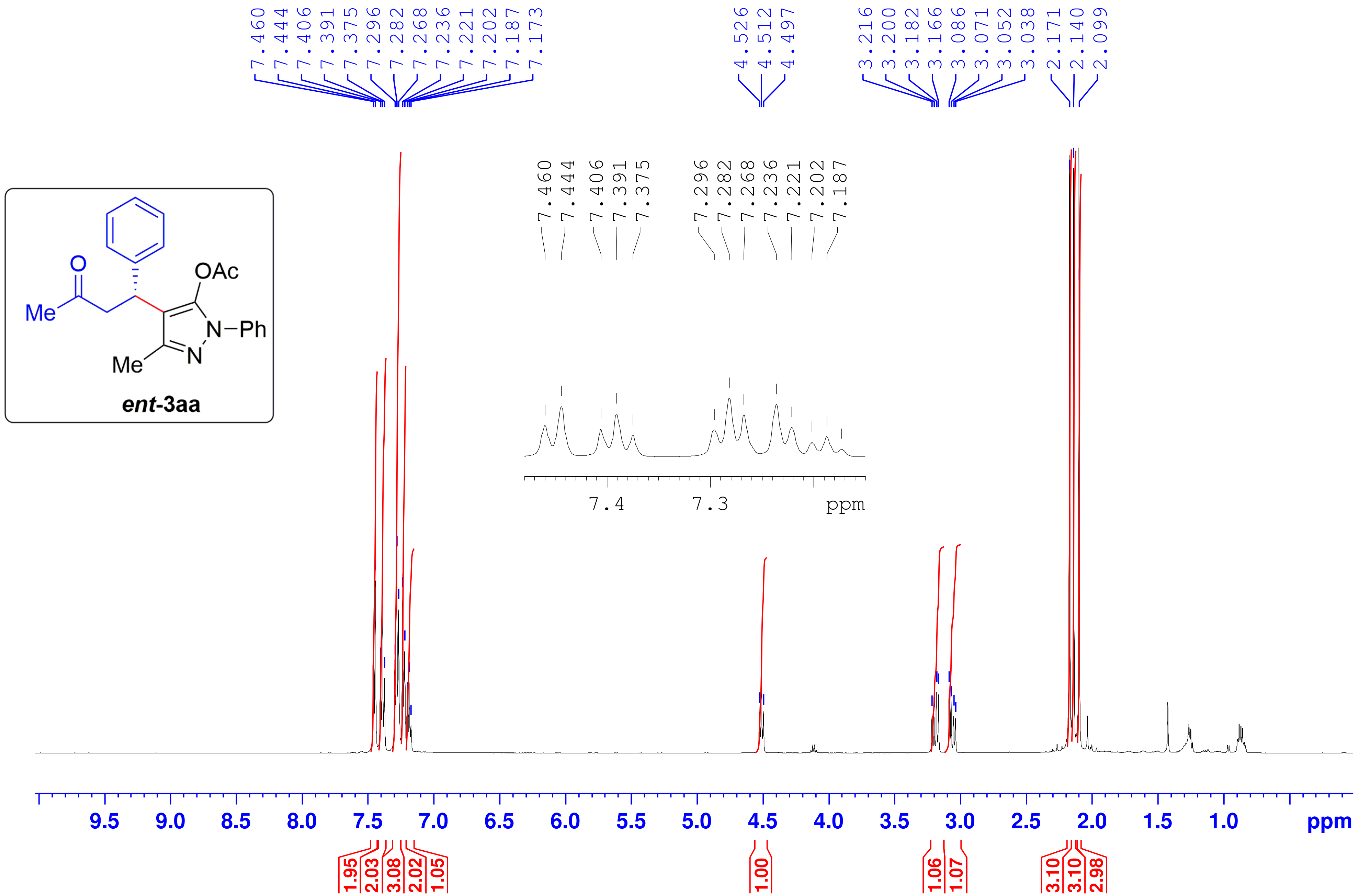
7.460
7.444
7.406
7.391
7.375
7.296
7.282
7.268
7.236
7.221
7.202
7.187
7.173
4.526
4.512
4.497
3.216
3.200
3.182
3.166
3.086
3.071
3.052
3.038
2.171
2.140
2.099
O
OAc
Me
N−Ph
N
Me
ent-3aa
7.4
7.3
ppm
9.5
9.0
8.5
8.0
7.5
7.0
6.5
6.0
5.5
5.0
4.5
4.0
3.5
3.0
2.5
2.0
1.5
1.0
ppm
1.95
2.03
3.08
2.02
1.05
1.00
1.06
1.07
3.10
3.10
2.98

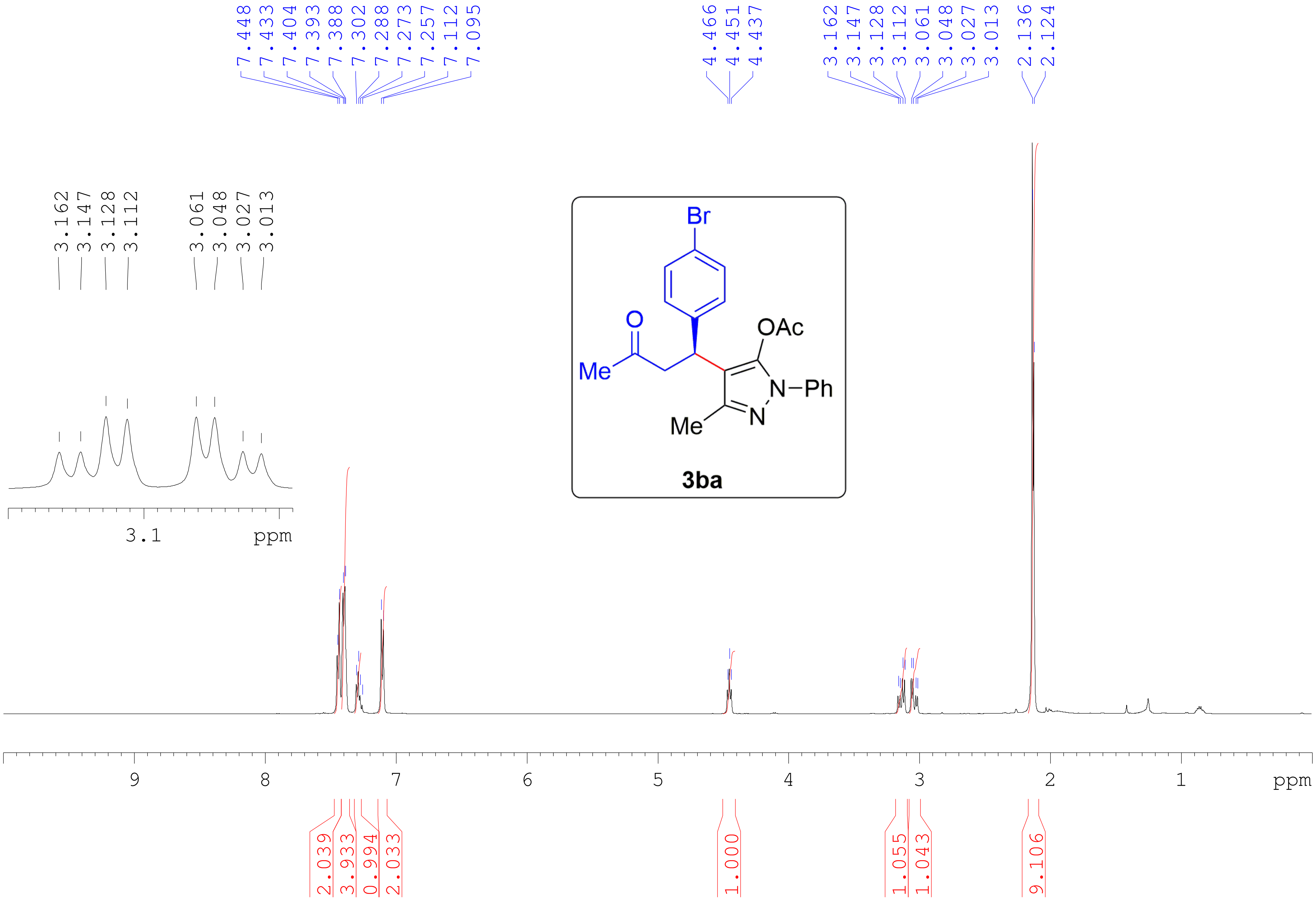
7.448
7.433
7.404
7.393
7.388
7.302
7.288
7.273
7.257
7.112
7.095
4.466
4.451
4.437
3.162
3.147
3.128
3.112
3.061
3.048
3.027
3.013
2.136
2.124
Br
O
OAc
Me
N−Ph
N
Me
3ba
3.1
ppm
9
8
7
6
5
4
3
2
1
ppm
2.039
3.933
0.994
2.033
1.000
1.055
1.043
9.106

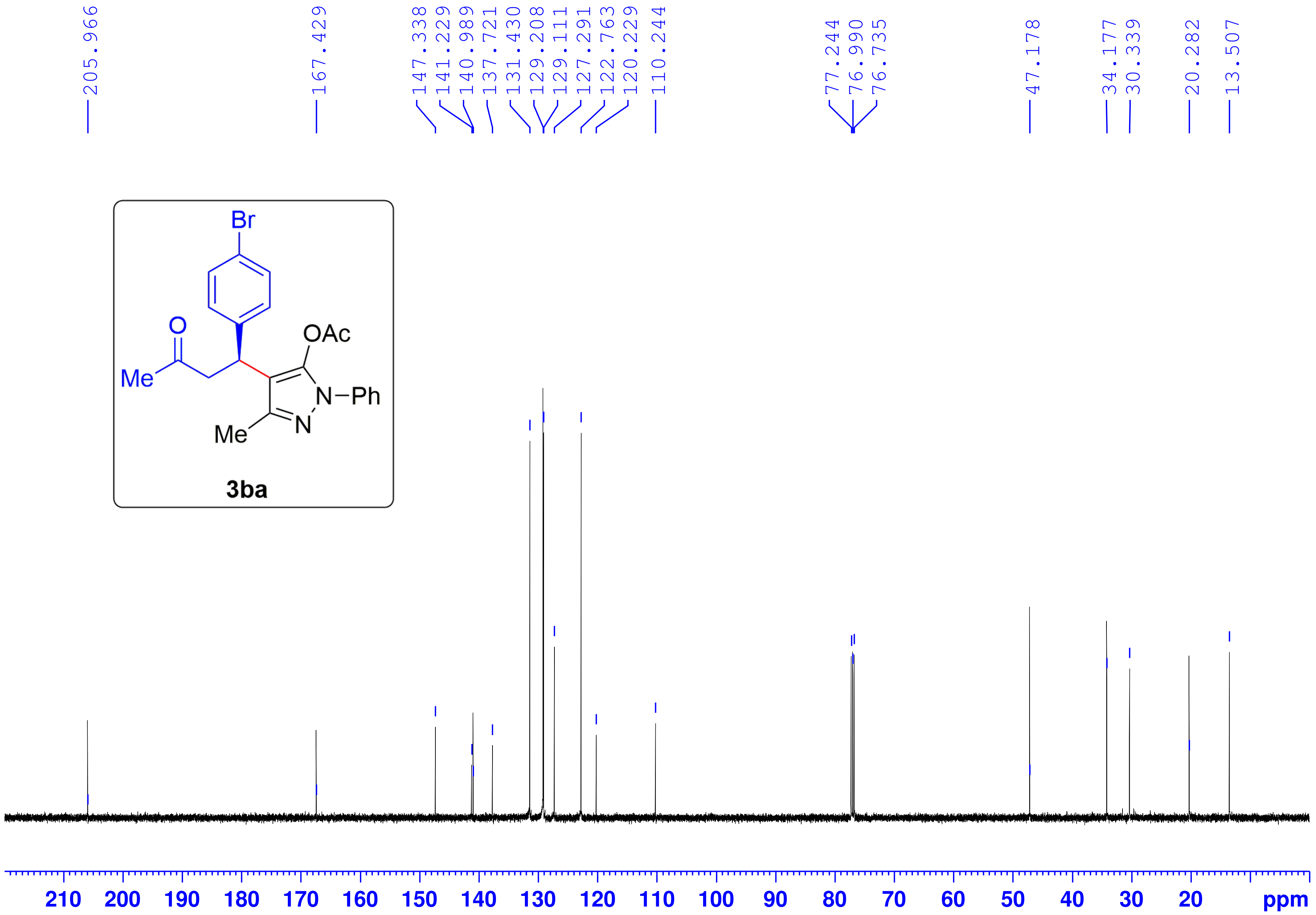
205.966
167.429
147.338
141.229
140.989
137.721
131.430
129.208
129.111
127.291
122.763
120.229
110.244
77.244
76.990
76.735
47.178
34.177
30.339
20.282
13.507
Br
O
OAc
Me
N−Ph
N
Me
3ba
210
200
190
180
170
160
150
140
130
120
110
100
90
80
70
60
50
40
30
20
ppm

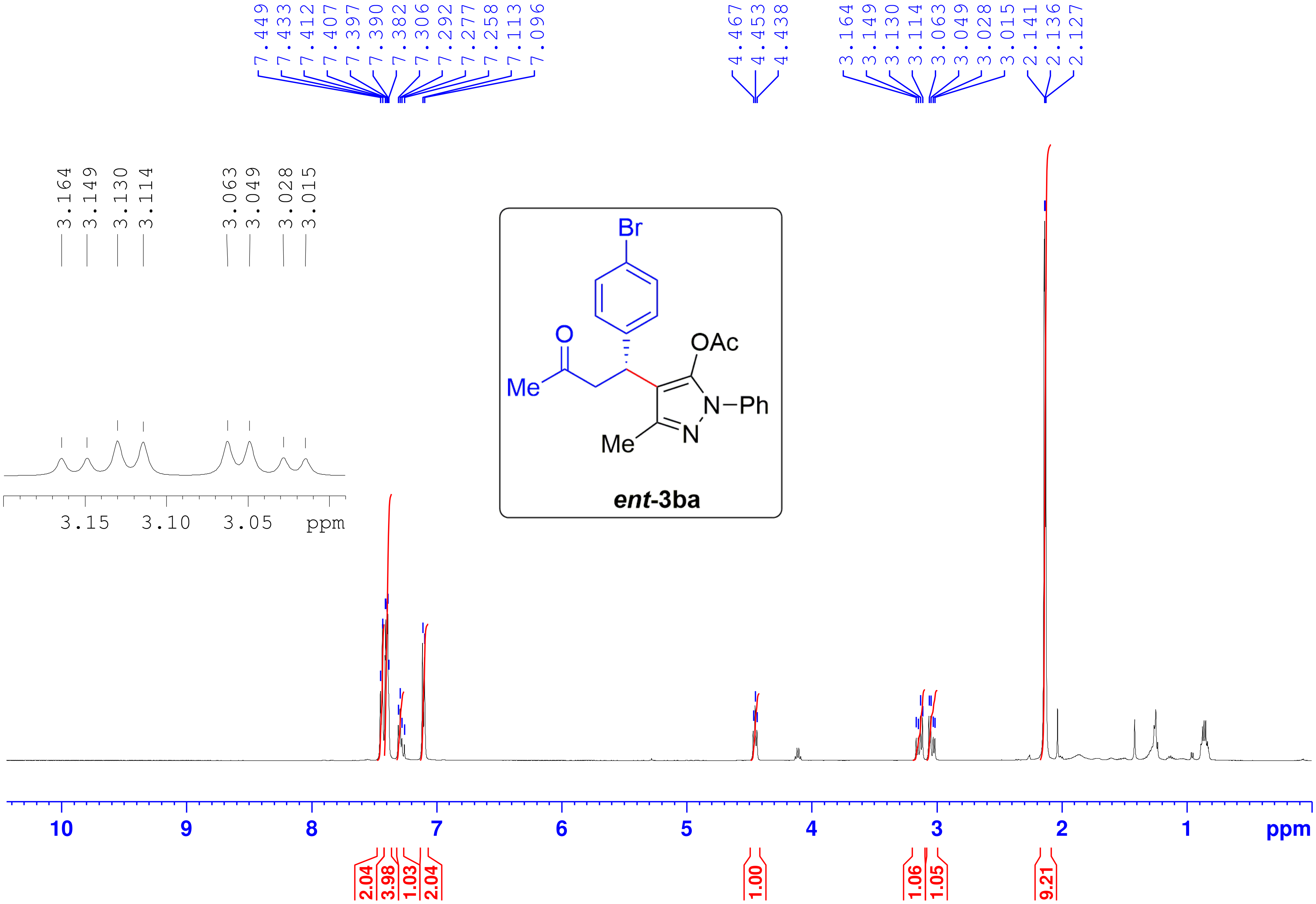

7.449 7.433 7.412 7.407 7.397 7.390 7.382 7.306 7.292 7.277 7.258 7.113 7.096
4.467 4.453 4.438
3.164 3.149 3.130 3.114 3.063 3.049 3.028 3.015 2.141 2.136 2.127
3.164 3.149 3.130 3.114 3.063 3.049 3.028 3.015
3.15 3.10 3.05 ppm
Br
O
OAc
Me
N−Ph
N
Me
ent-3ba
10 9 8 7 6 5 4 3 2 1 ppm
2.04 3.98 1.03 2.04 1.00 1.06 1.05 9.21

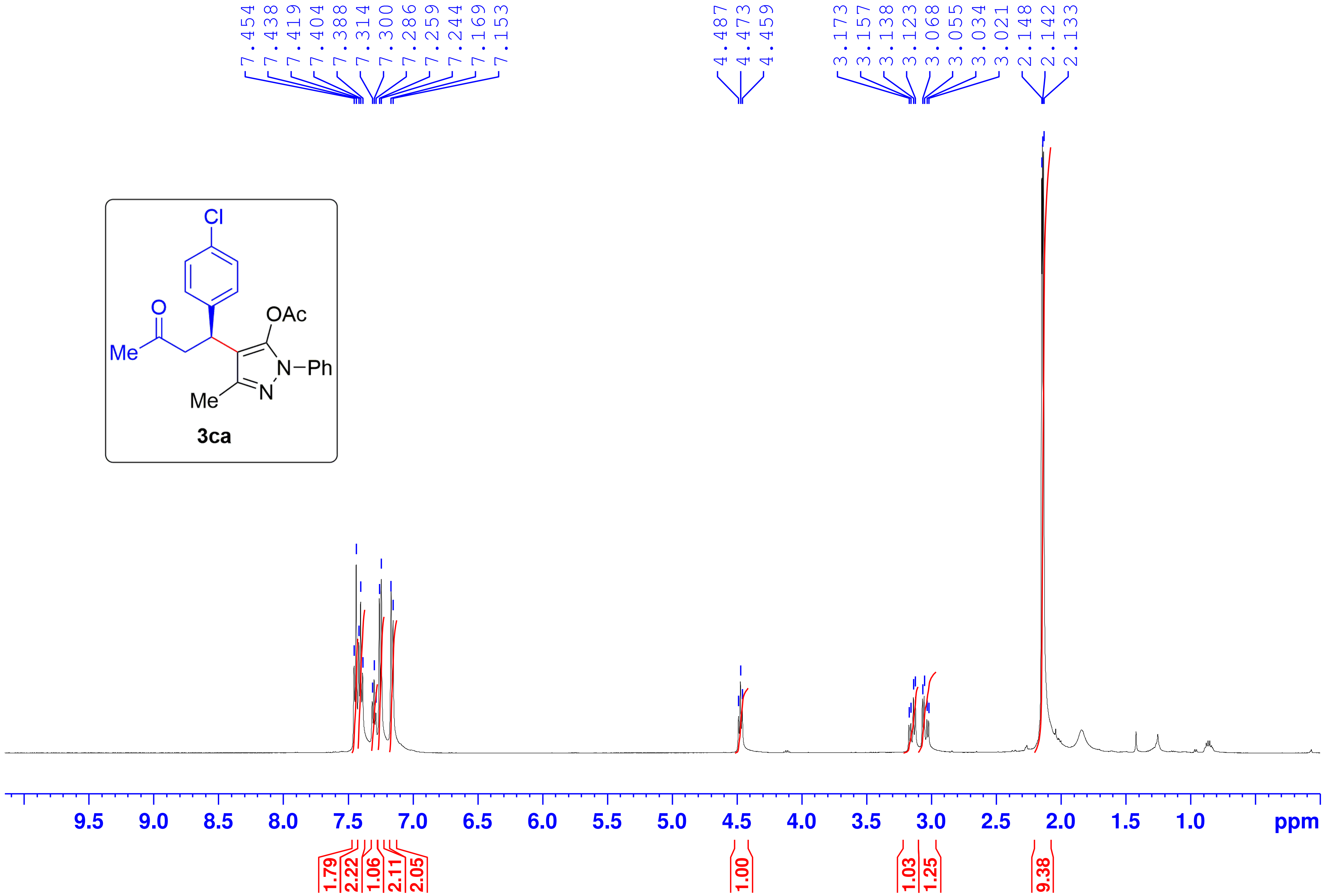
7.454
7.438
7.419
7.404
7.388
7.314
7.300
7.286
7.259
7.244
7.169
7.153
4.487
4.473
4.459
3.173
3.157
3.138
3.123
3.068
3.055
3.034
3.021
2.148
2.142
2.133
Cl
O
OAc
Me
N−Ph
Me
N
3ca
9.5
9.0
8.5
8.0
7.5
7.0
6.5
6.0
5.5
5.0
4.5
4.0
3.5
3.0
2.5
2.0
1.5
1.0
ppm
1.79
2.22
1.06
2.11
2.05
1.00
1.03
1.25
9.38

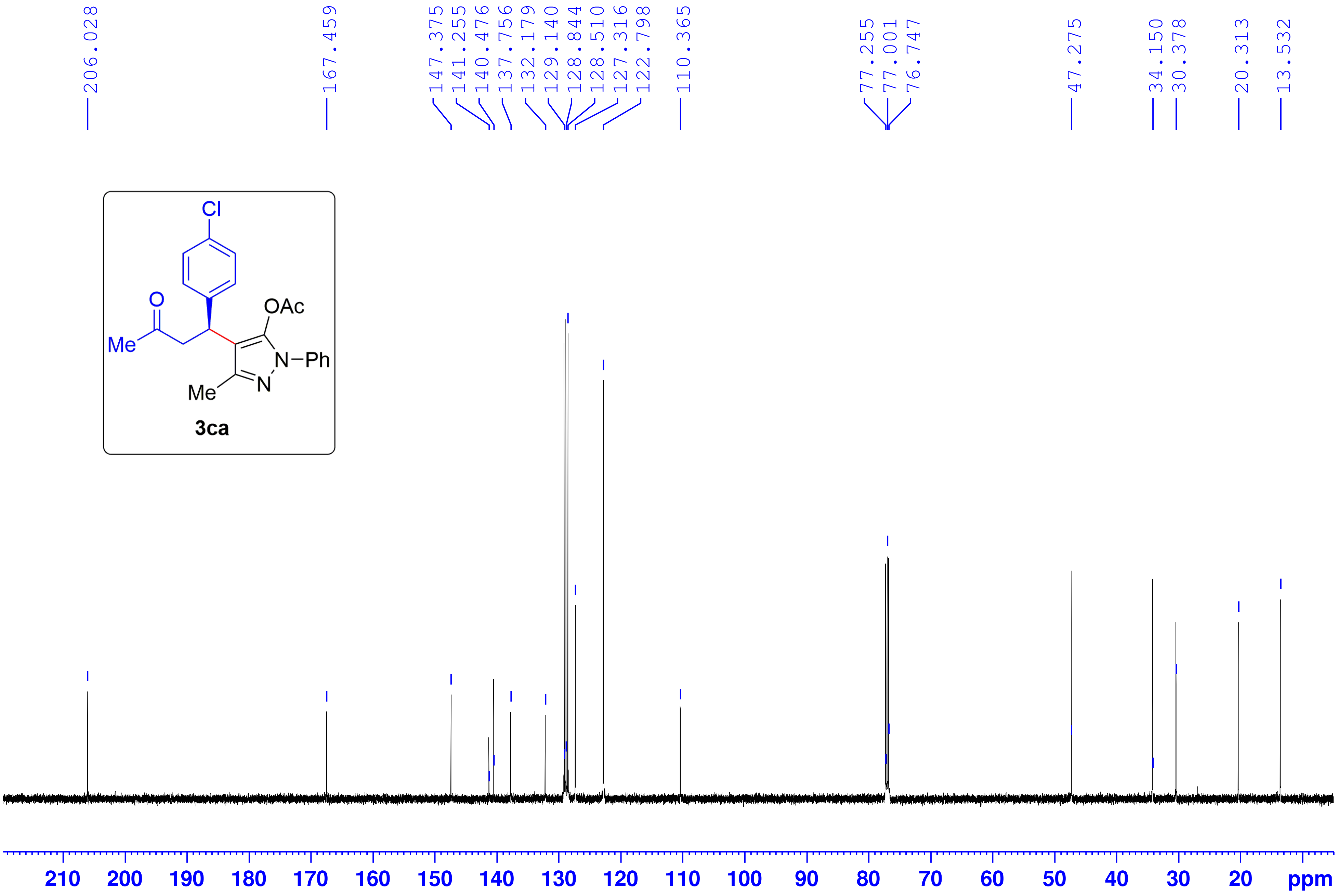
206.028
167.459
147.375
141.255
140.476
137.756
132.179
129.140
128.844
128.510
127.316
122.798
110.365
77.255
77.001
76.747
47.275
34.150
30.378
20.313
13.532
210
200
190
180
170
160
150
140
130
120
110
100
90
80
70
60
50
40
30
20
ppm
Cl
O
OAc
Me
N−Ph
N
Me

3ca

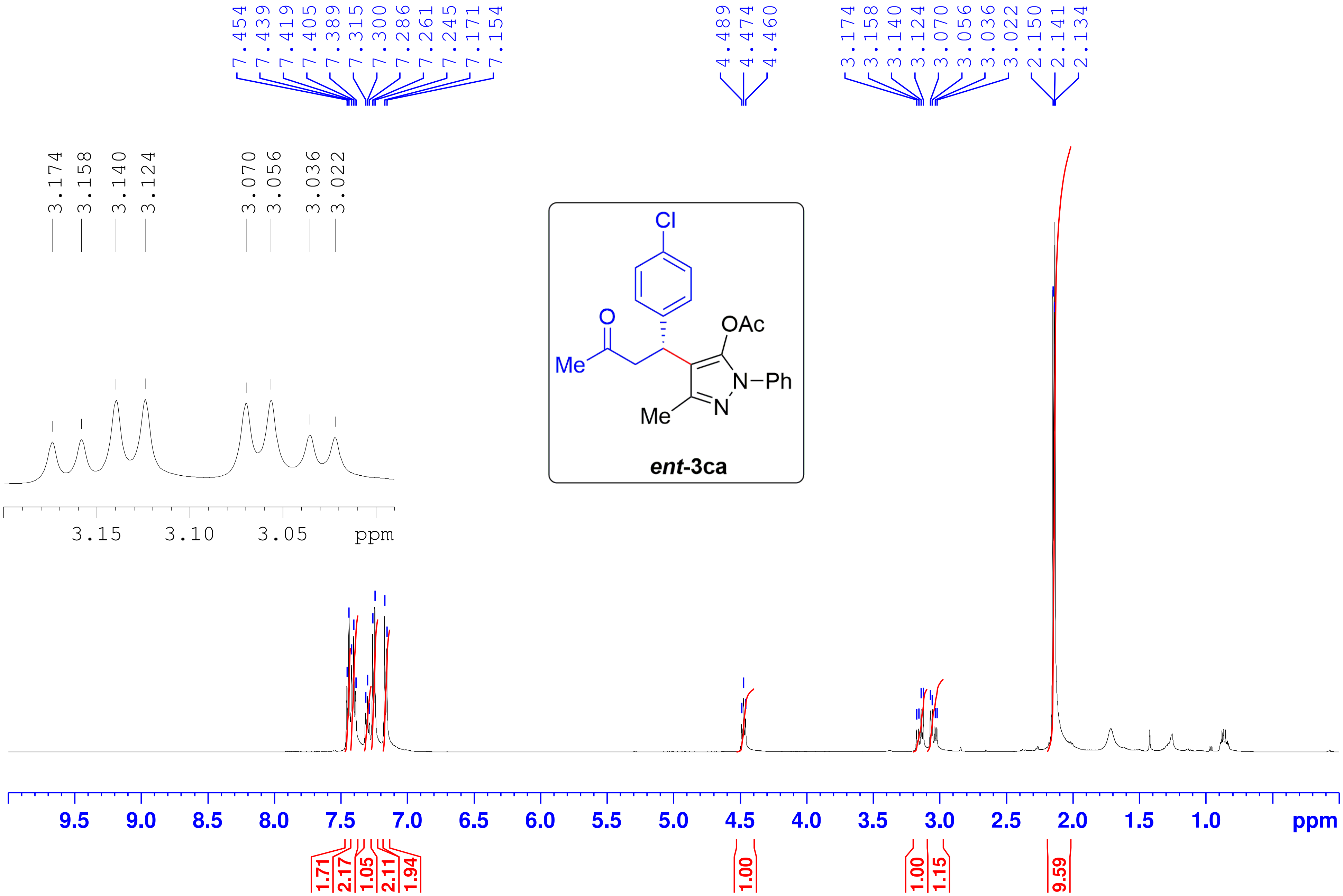
7.454
7.439
7.419
7.405
7.389
7.315
7.300
7.286
7.261
7.245
7.171
7.154
4.489
4.474
4.460
3.174
3.158
3.140
3.124
3.070
3.056
3.036
3.022
2.150
2.141
2.134
3.15
3.10
3.05
ppm
Cl
O
OAc
Me
N−Ph
N
Me
ent-3ca
9.5
9.0
8.5
8.0
7.5
7.0
6.5
6.0
5.5
5.0
4.5
4.0
3.5
3.0
2.5
2.0
1.5
1.0
ppm
1.71
2.17
1.05
2.11
1.94
1.00
1.00
1.15
9.59

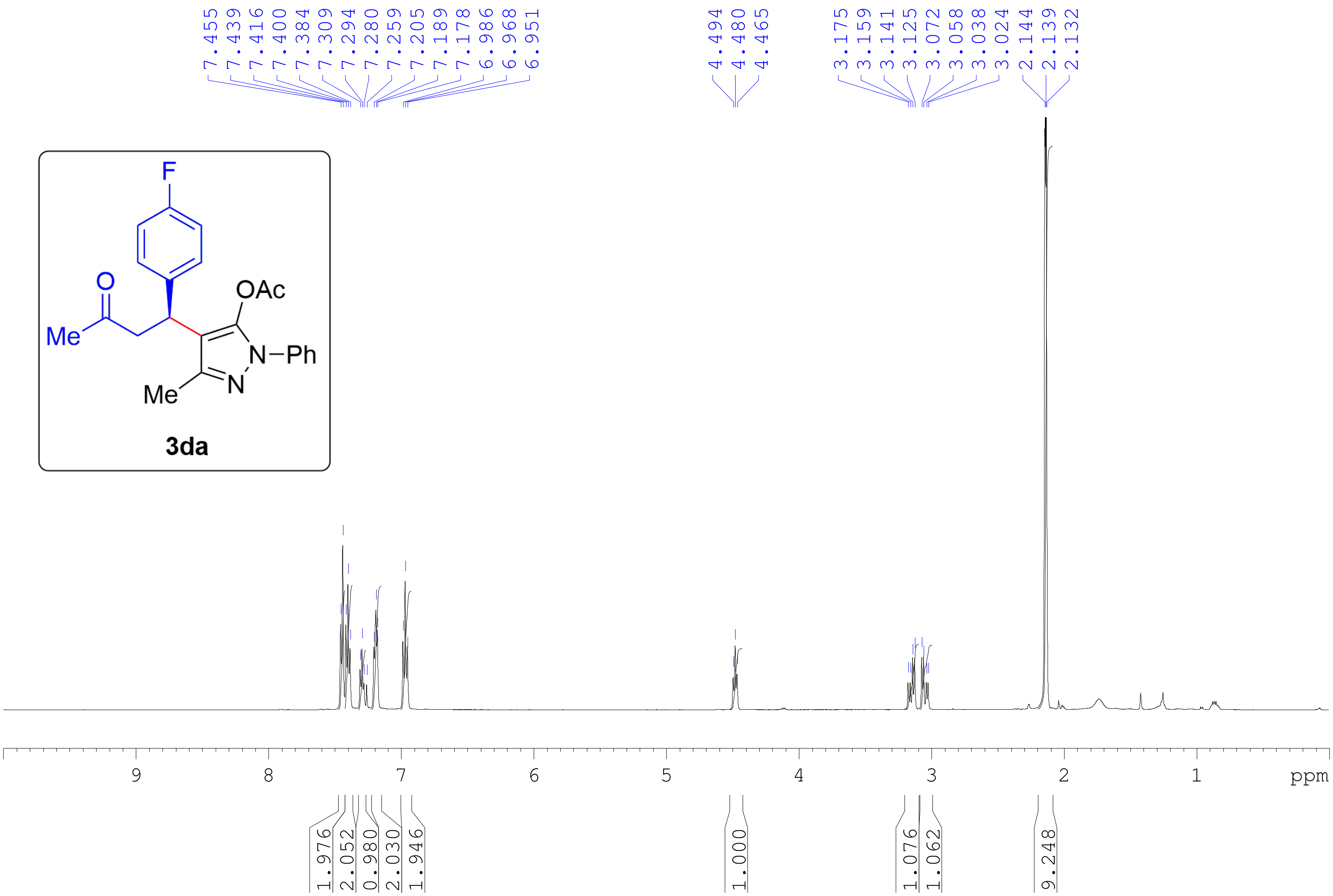
7.455
7.439
7.416
7.400
7.384
7.309
7.294
7.280
7.259
7.205
7.189
7.178
6.986
6.968
6.951
4.494
4.480
4.465
3.175
3.159
3.141
3.125
3.072
3.058
3.038
3.024
2.144
2.139
2.132
F
O
OAc
Me
N–Ph
N
Me
3da
9
8
7
6
5
4
3
2
1
ppm
1.976
2.052
0.980
2.030
1.946
1.000
1.076
1.062
9.248

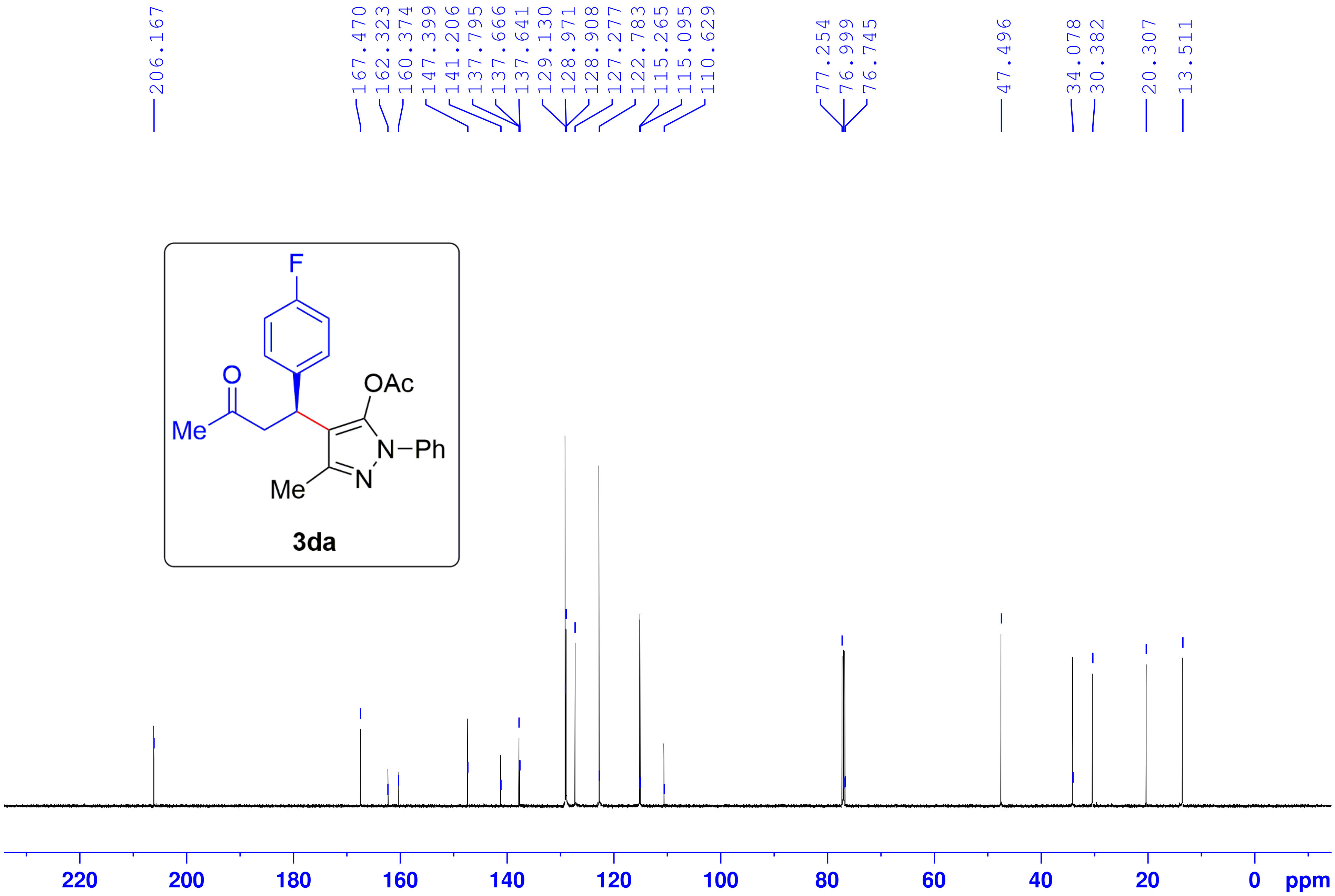
206.167
167.470
162.323
160.374
147.399
141.206
137.795
137.666
137.641
129.130
128.971
128.908
127.277
122.783
115.265
115.095
110.629
77.254
76.999
76.745
47.496
34.078
30.382
20.307
13.511
F
O
OAc
Me
N−Ph
N
Me
3da
220
200
180
160
140
120
100
80
60
40
20
0
ppm

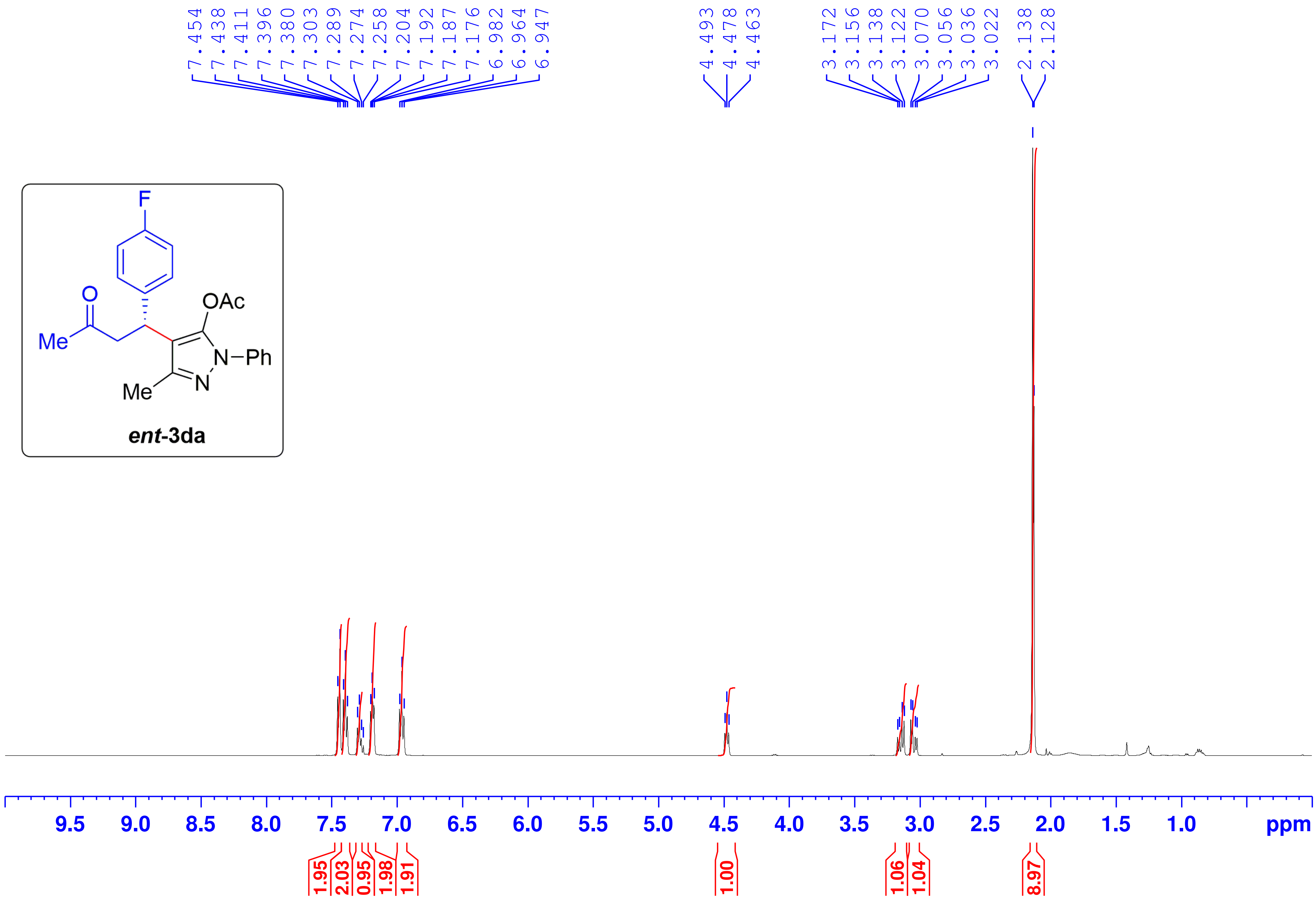
7.454
7.438
7.411
7.396
7.380
7.303
7.289
7.274
7.258
7.204
7.192
7.187
7.176
6.982
6.964
6.947
4.493
4.478
4.463
3.172
3.156
3.138
3.122
3.070
3.056
3.036
3.022
2.138
2.128
F
O
OAc
Me
N−Ph
N
Me
ent-3da
9.5
9.0
8.5
8.0
7.5
7.0
6.5
6.0
5.5
5.0
4.5
4.0
3.5
3.0
2.5
2.0
1.5
1.0
ppm
1.95
2.03
0.95
1.98
1.91
1.00
1.06
1.04
8.97

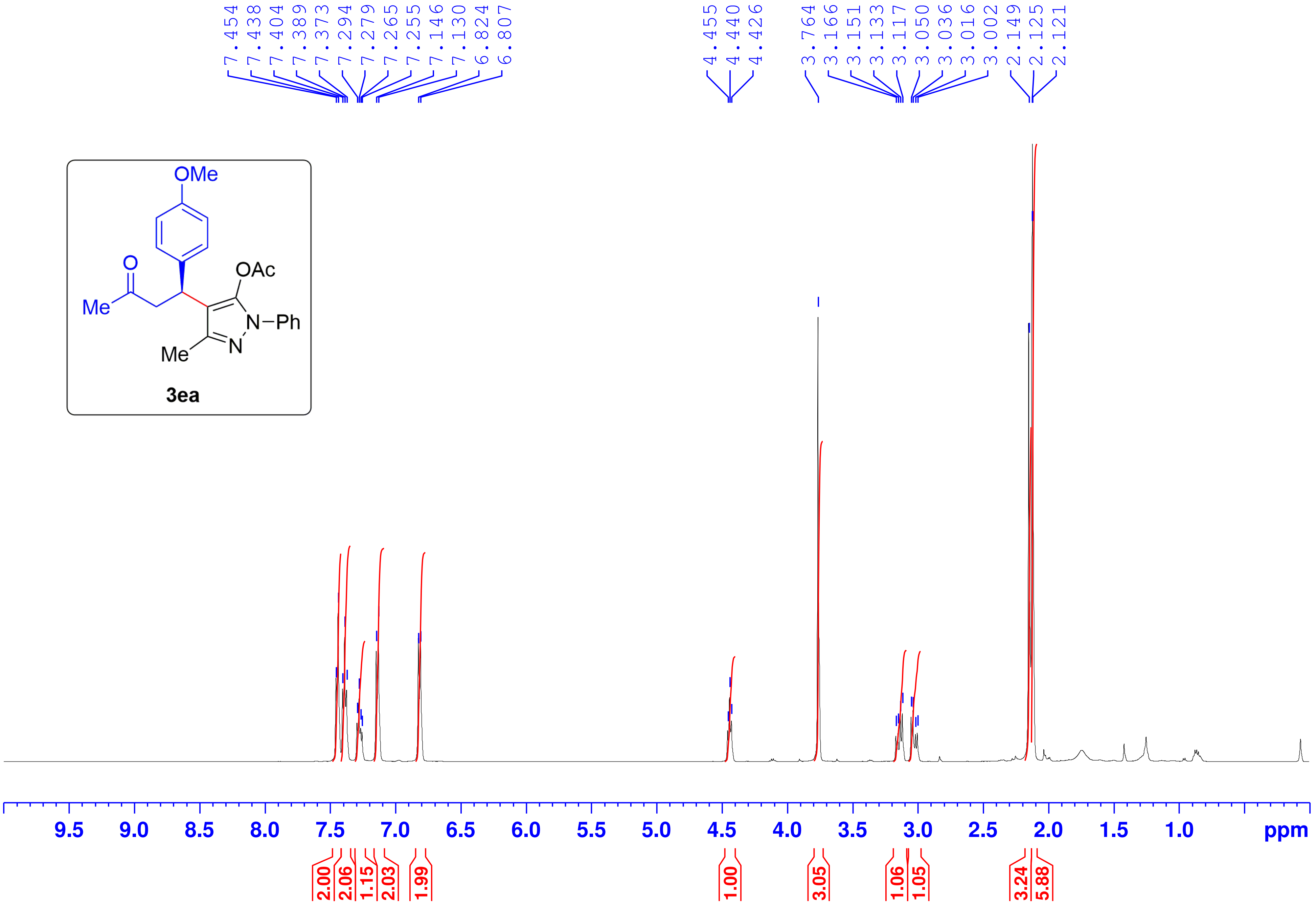
OMe
O
OAc
Me
N−Ph
N
Me
7.454
7.438
7.404
7.389
7.373
7.294
7.279
7.265
7.255
7.146
7.130
6.824
6.807
4.455
4.440
4.426
3.764
3.166
3.151
3.133
3.117
3.050
3.036
3.016
3.002
2.149
2.125
2.121
9.5
9.0
8.5
8.0
7.5
7.0
6.5
6.0
5.5
5.0
4.5
4.0
3.5
3.0
2.5
2.0
1.5
1.0
ppm
2.00
2.06
1.15
2.03
1.99
1.00
3.05
1.06
1.05
3.24
5.88

3ea

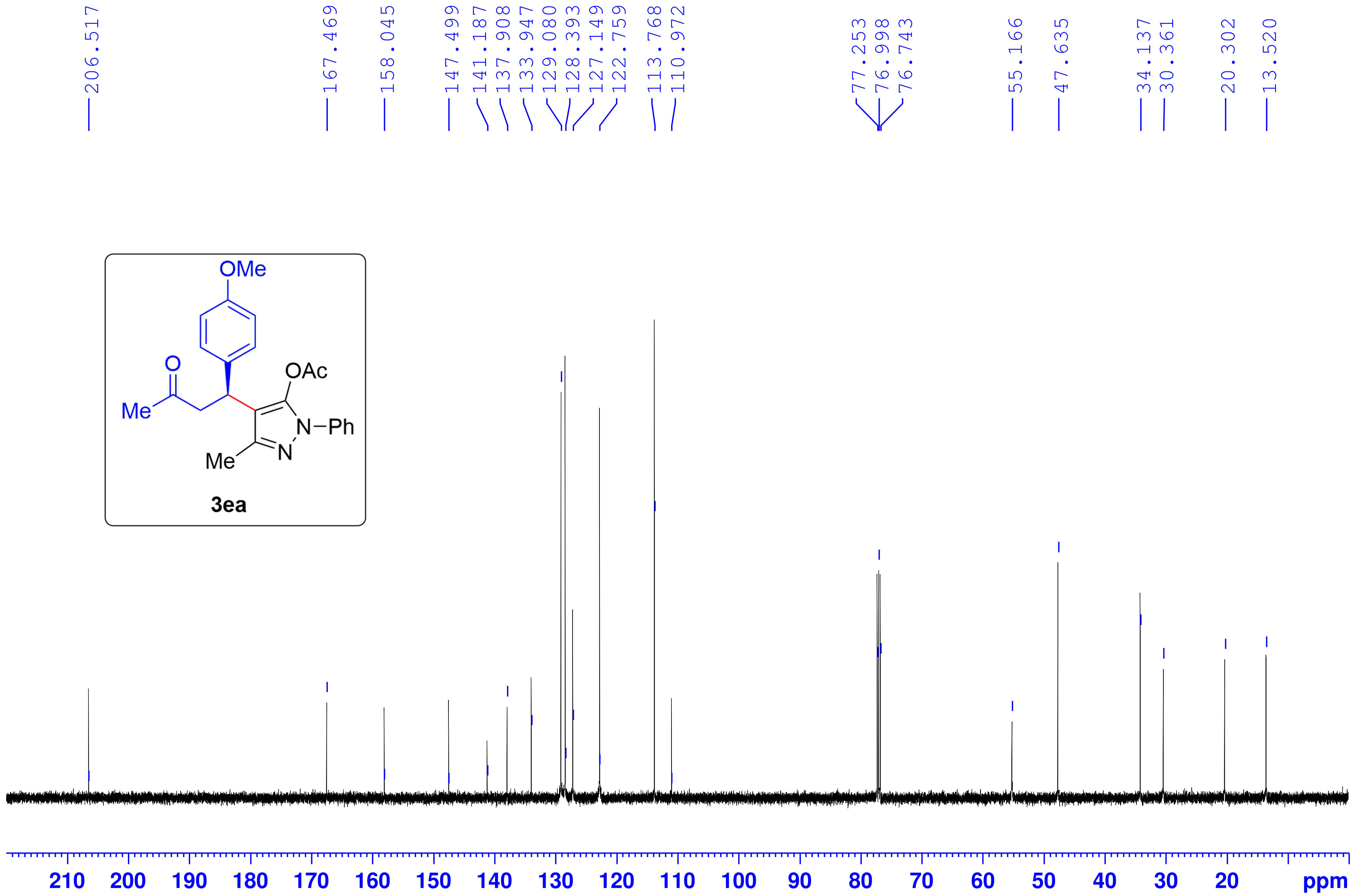
206.517
167.469
158.045
147.499
141.187
137.908
133.947
129.080
128.393
127.149
122.759
113.768
110.972
77.253
76.998
76.743
55.166
47.635
34.137
30.361
20.302
13.520
OMe
O
Me
OAc
N−Ph
N
Me
3ea
210 200 190 180 170 160 150 140 130 120 110 100 90 80 70 60 50 40 30 20 ppm

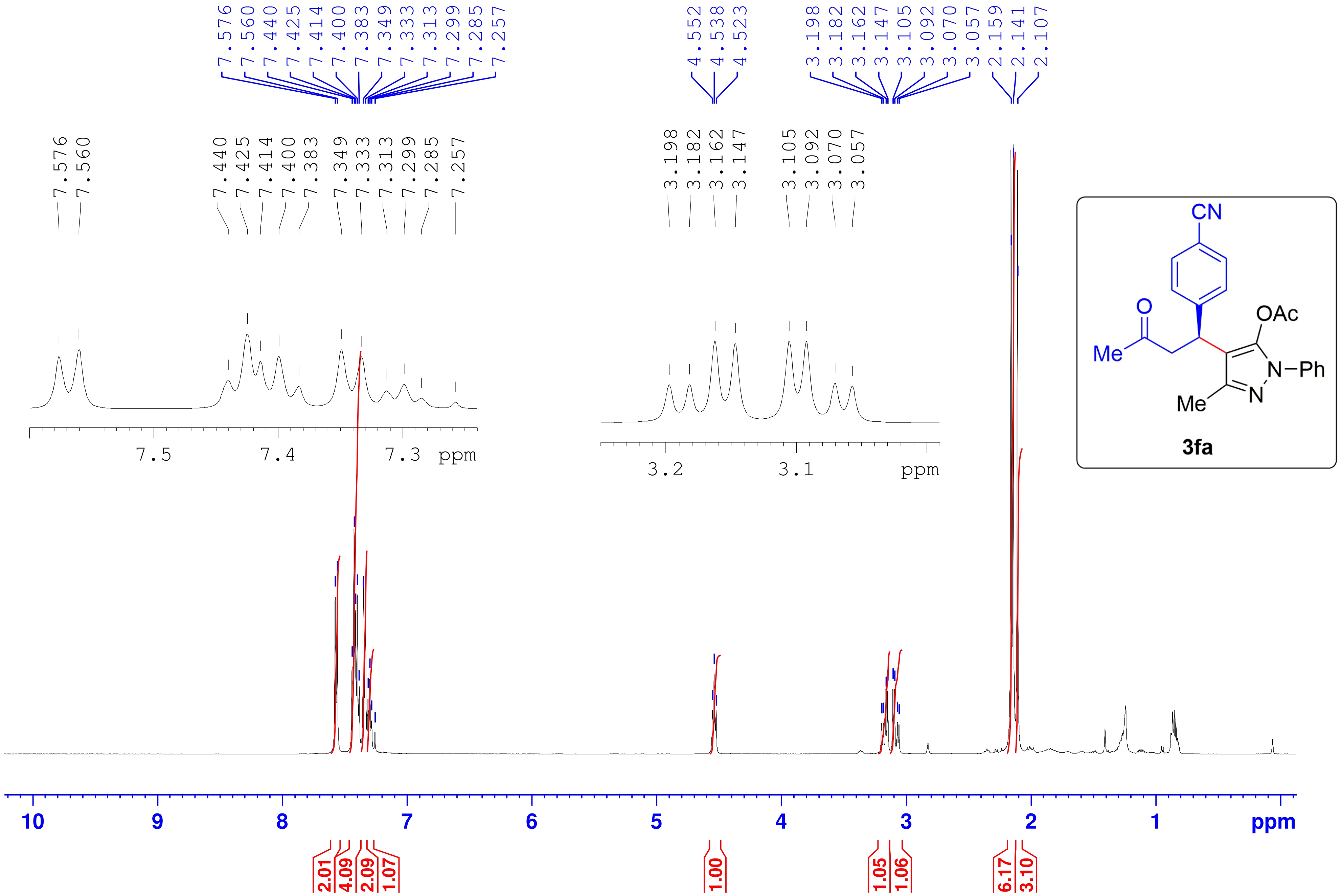
7.576
7.560
7.440
7.425
7.414
7.400
7.383
7.349
7.333
7.313
7.299
7.285
7.257
4.552
4.538
4.523
3.198
3.182
3.162
3.147
3.105
3.092
3.070
3.057
2.159
2.141
2.107
7.5
7.4
7.3
ppm
3.2
3.1
ppm
CN
O
OAc
Me
N−Ph
Me
N
10
9
8
7
6
5
4
3
2
1
ppm
2.01
4.09
2.09
1.07
1.00
1.05
1.06
6.17
3.10

3fa

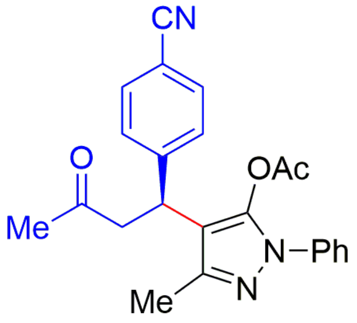
CN
O
Me
OAc
N–Ph
N
Me

3fa

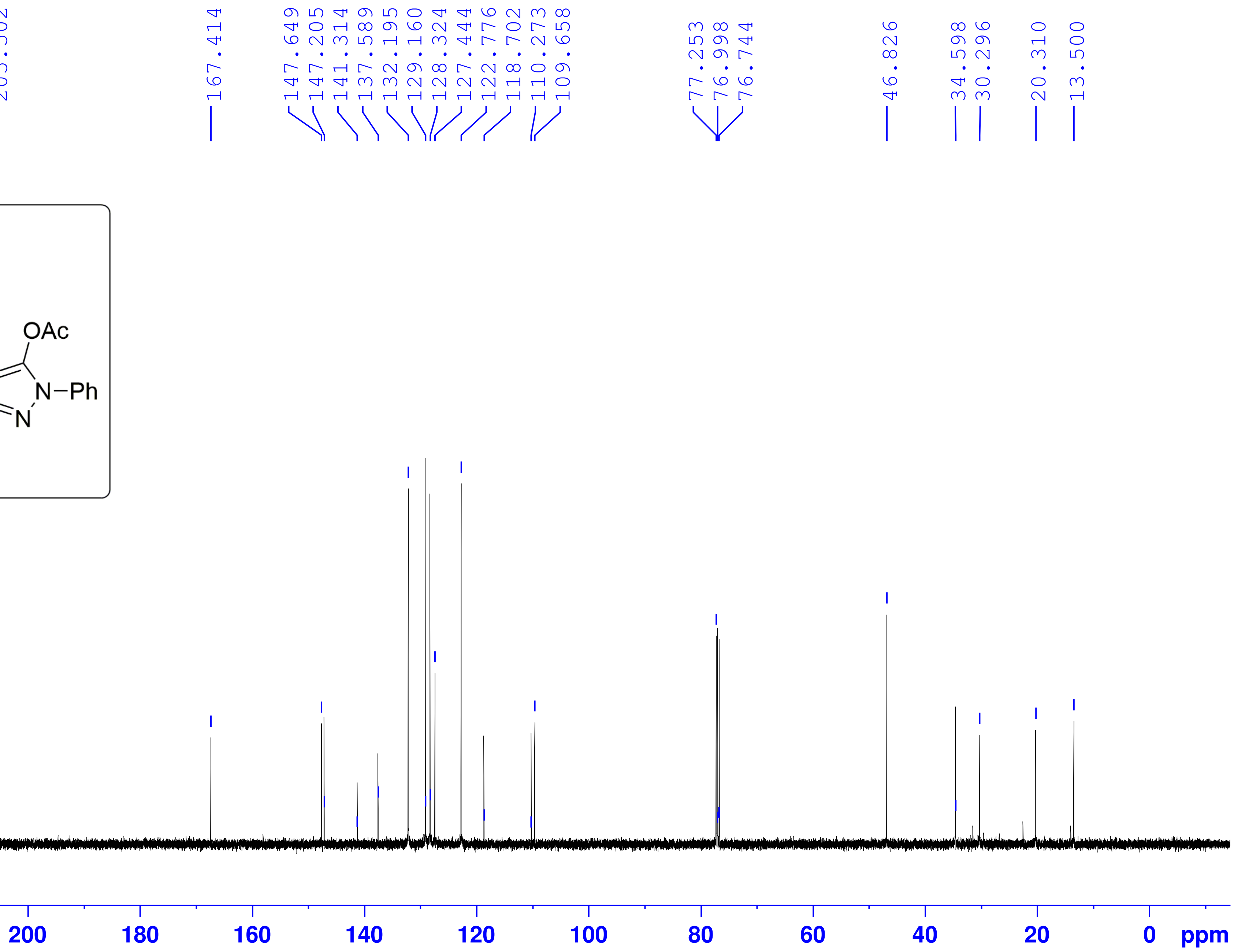
205.502
167.414
147.649
147.205
141.314
137.589
132.195
129.160
128.324
127.444
122.776
118.702
110.273
109.658
77.253
76.998
76.744
46.826
34.598
30.296
20.310
13.500
220
200
180
160
140
120
100
80
60
40
20
0
ppm

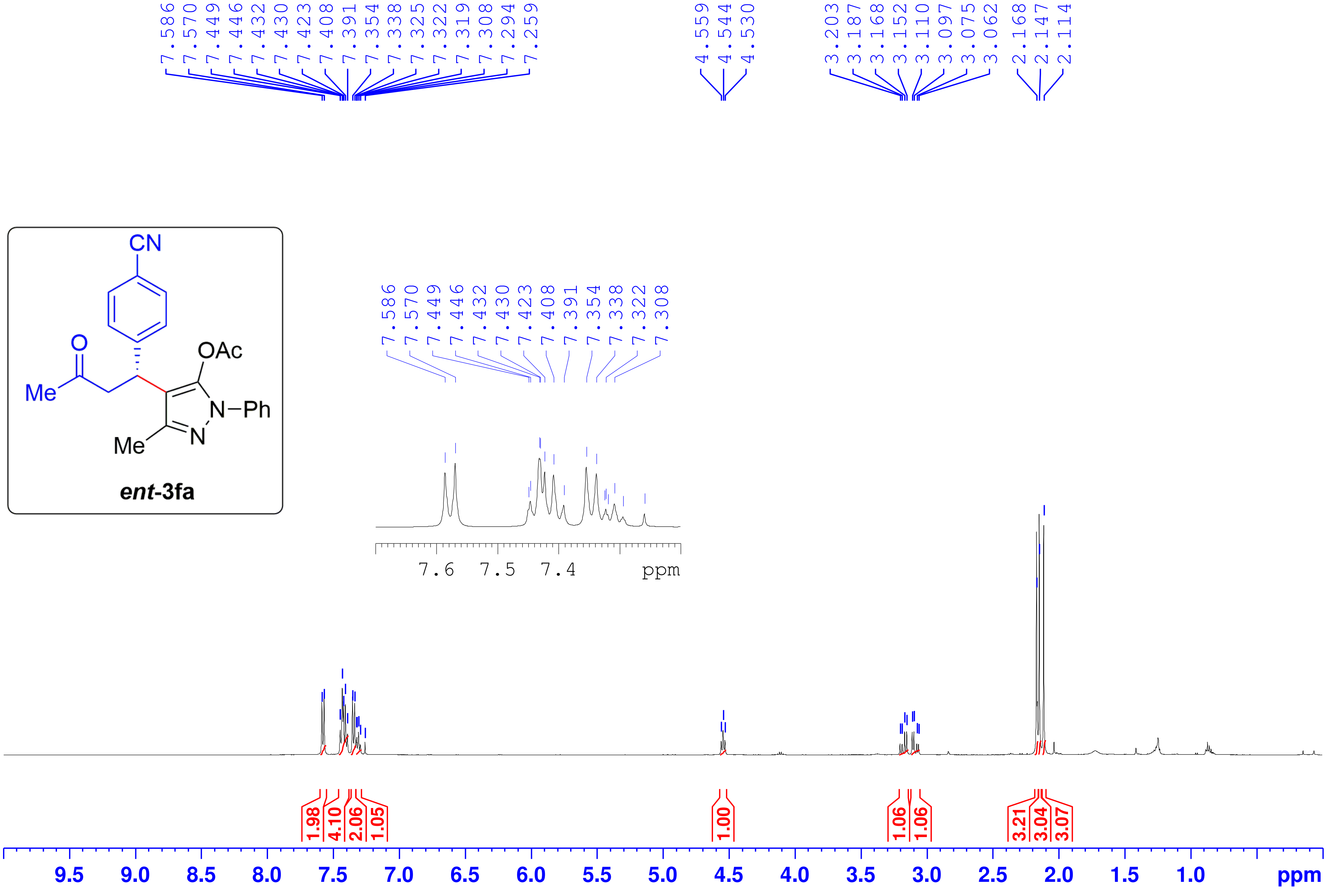
7.586
7.570
7.449
7.446
7.432
7.430
7.423
7.408
7.391
7.354
7.338
7.325
7.322
7.319
7.308
7.294
7.259
4.559
4.544
4.530
3.203
3.187
3.168
3.152
3.110
3.097
3.075
3.062
2.168
2.147
2.114
CN
O
OAc
Me
N−Ph
N
Me
ent-3fa
7.6
7.5
7.4
ppm
1.98
4.10
2.06
1.05
1.00
1.06
1.06
3.21
3.04
3.07
9.5
9.0
8.5
8.0
7.5
7.0
6.5
6.0
5.5
5.0
4.5
4.0
3.5
3.0
2.5
2.0
1.5
1.0
ppm

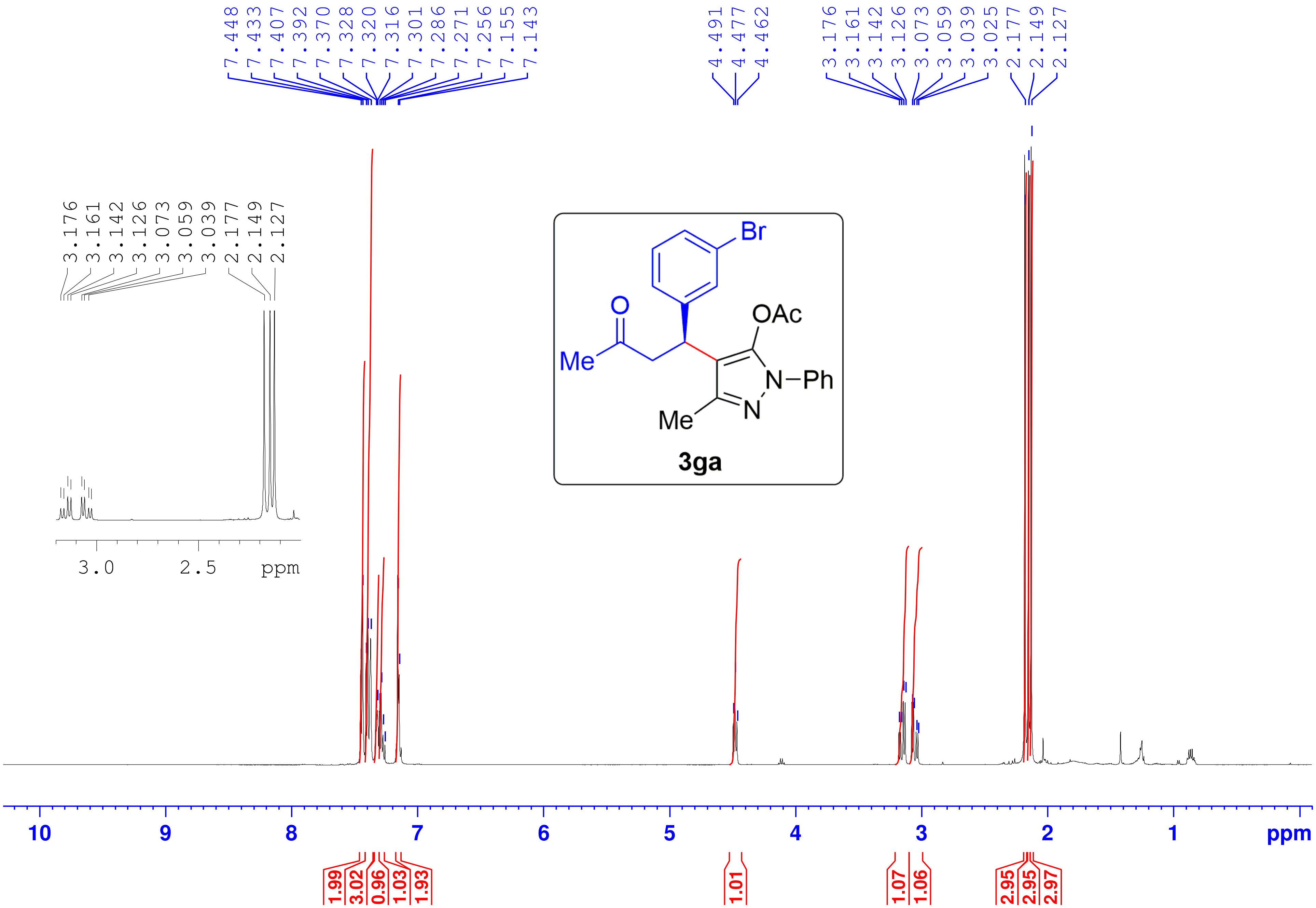

7.448
7.433
7.407
7.392
7.370
7.328
7.320
7.316
7.301
7.286
7.271
7.256
7.155
7.143
4.491
4.477
4.462
3.176
3.161
3.142
3.126
3.073
3.059
3.039
3.025
2.177
2.149
2.127
Br
O
OAc
Me
N−Ph
N
Me
3ga
3.0
2.5
ppm
10
9
8
7
6
5
4
3
2
1
ppm
1.99
3.02
0.96
1.03
1.93
1.01
1.07
1.06
2.95
2.95
2.97

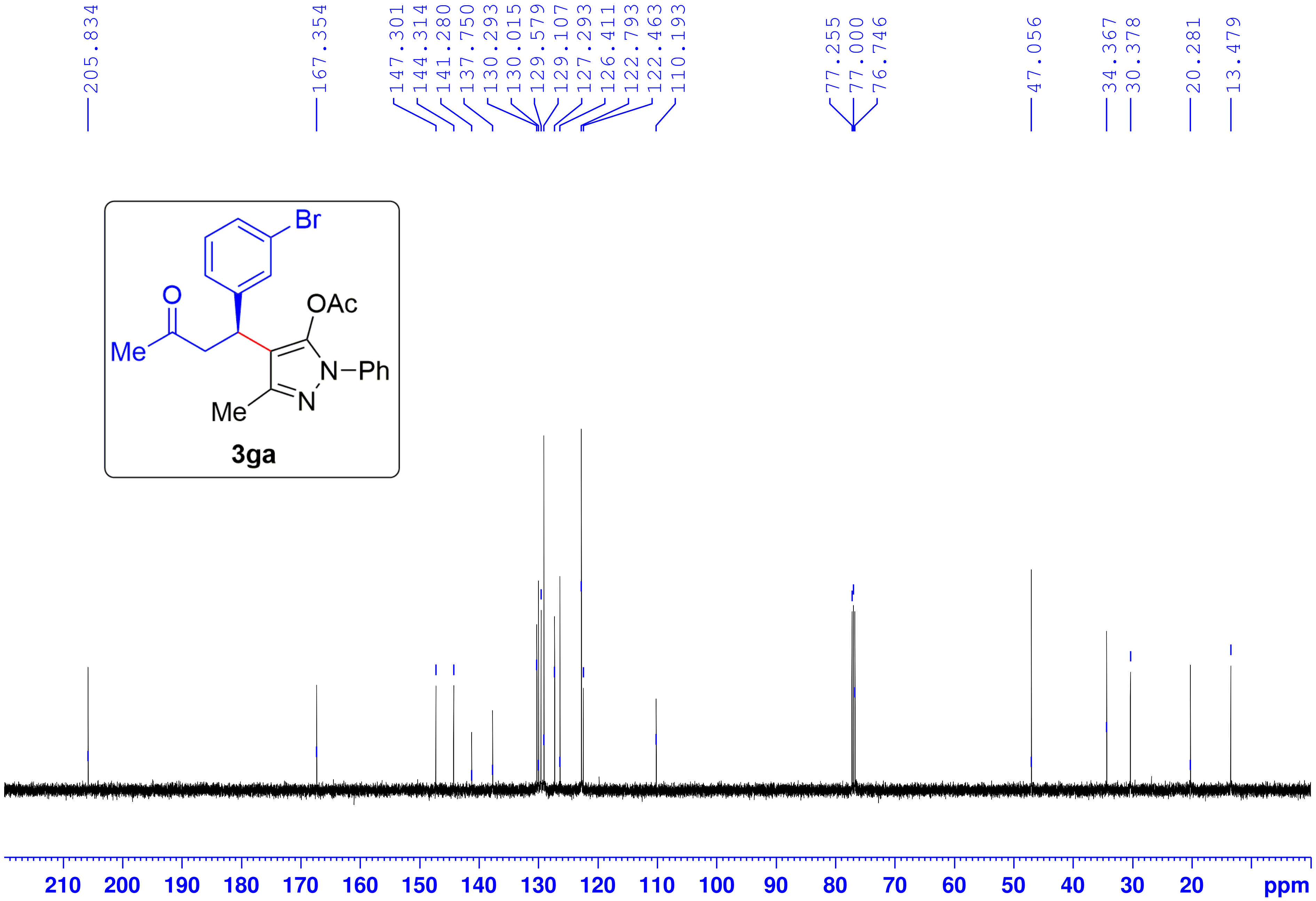
205.834
167.354
147.301
144.314
141.280
137.750
130.293
130.015
129.579
129.107
127.293
126.411
122.793
122.463
110.193
77.255
77.000
76.746
47.056
34.367
30.378
20.281
13.479
Br
O
OAc
Me
N−Ph
N
Me
3ga
210
200
190
180
170
160
150
140
130
120
110
100
90
80
70
60
50
40
30
20
ppm

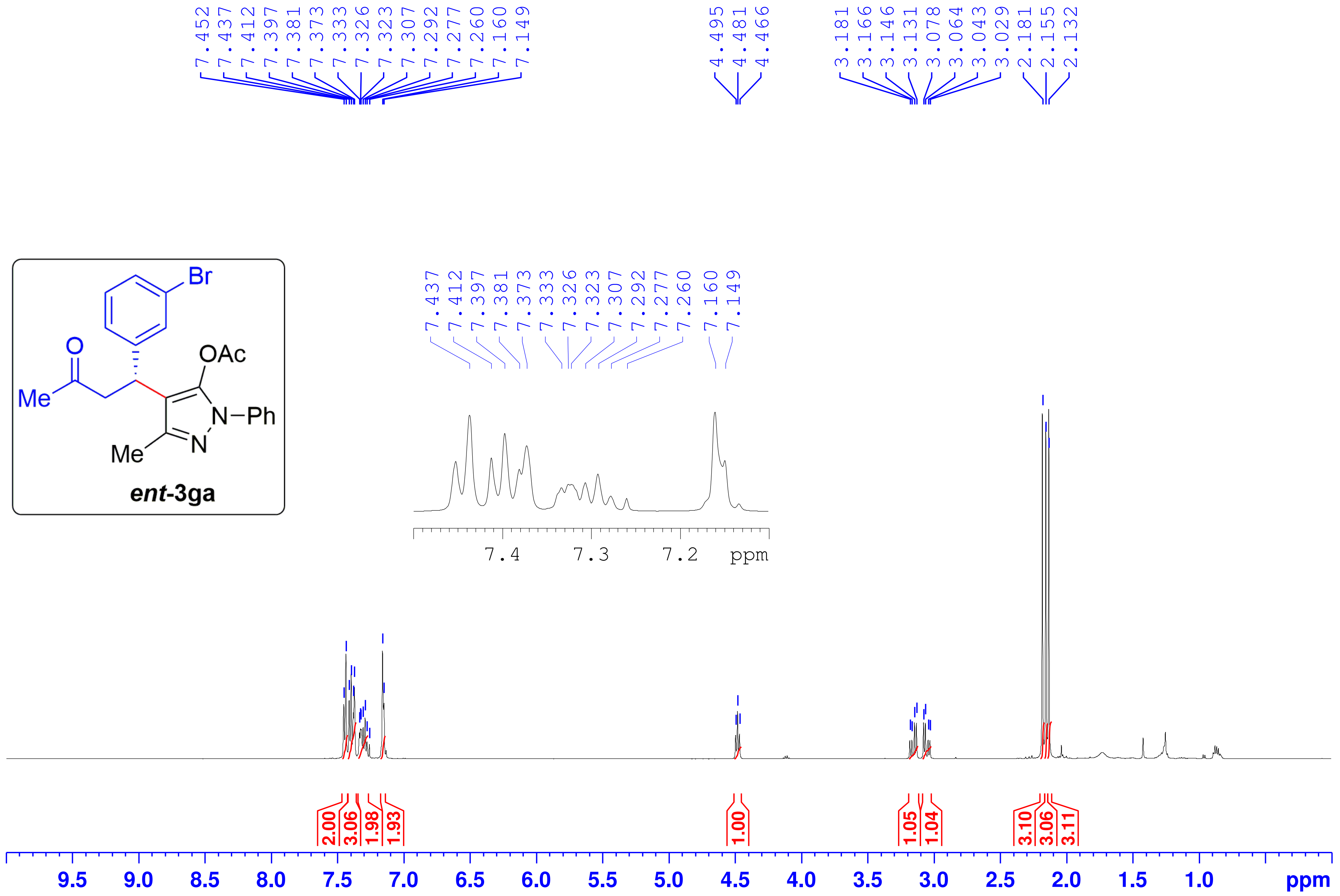
7.452
7.437
7.412
7.397
7.381
7.373
7.333
7.326
7.323
7.307
7.292
7.277
7.260
7.160
7.149
4.495
4.481
4.466
3.181
3.166
3.146
3.131
3.078
3.064
3.043
3.029
2.181
2.155
2.132
Br
O
OAc
Me
N−Ph
N
Me
ent-3ga
7.4
7.3
7.2
ppm
2.00
3.06
1.98
1.93
1.00
1.05
1.04
3.10
3.06
3.11
9.5
9.0
8.5
8.0
7.5
7.0
6.5
6.0
5.5
5.0
4.5
4.0
3.5
3.0
2.5
2.0
1.5
1.0
ppm

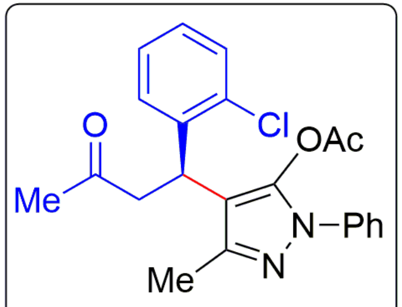
O
Me
Cl
OAc
N−Ph
N
Me

3ha

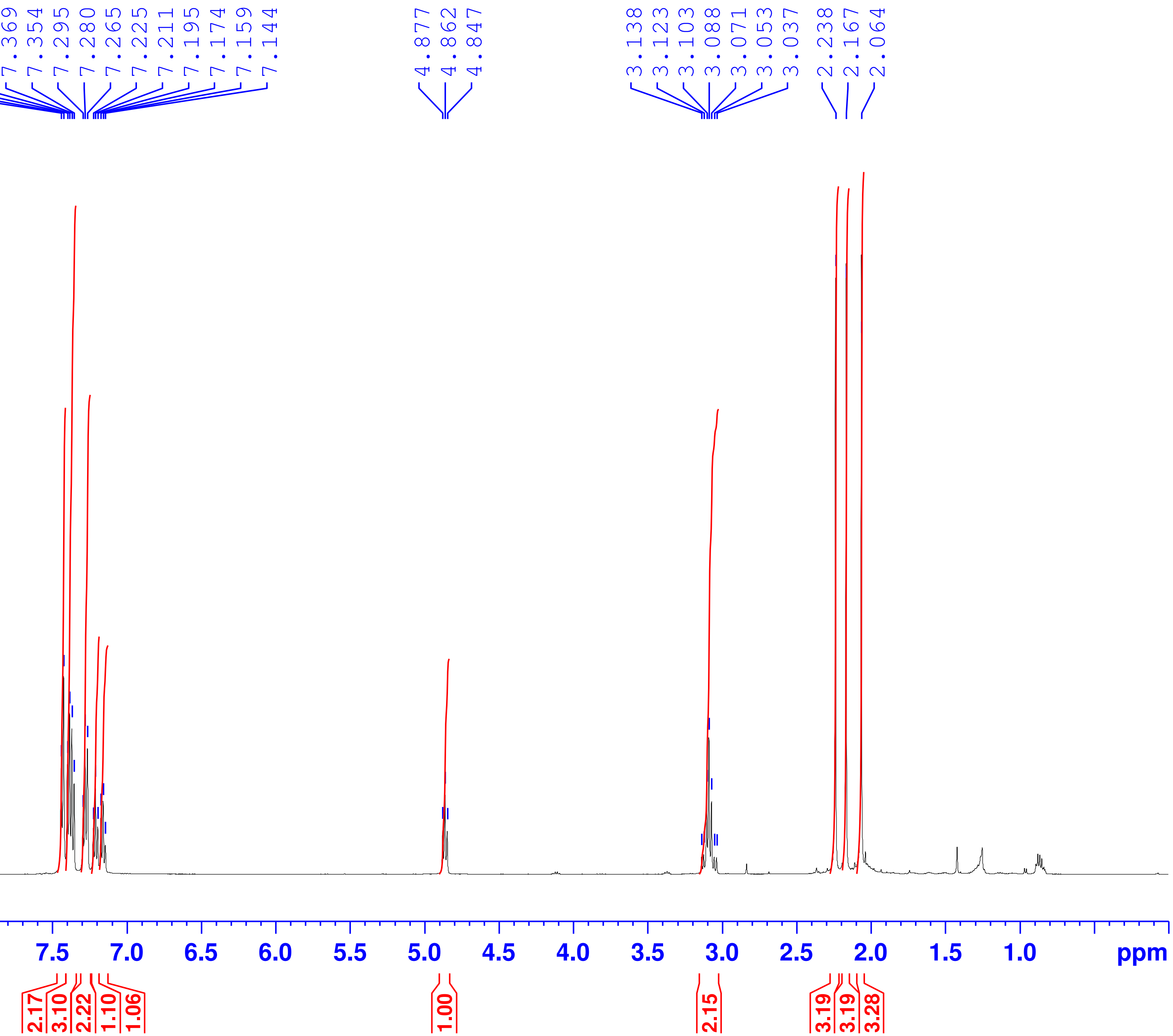
7.441
7.424
7.400
7.385
7.369
7.354
7.295
7.280
7.265
7.225
7.211
7.195
7.174
7.159
7.144
4.877
4.862
4.847
3.138
3.123
3.103
3.088
3.071
3.053
3.037
2.238
2.167
2.064
9.5
9.0
8.5
8.0
7.5
7.0
6.5
6.0
5.5
5.0
4.5
4.0
3.5
3.0
2.5
2.0
1.5
1.0
ppm
2.17
3.10
2.22
1.10
1.06
1.00
2.15
3.19
3.19
3.28

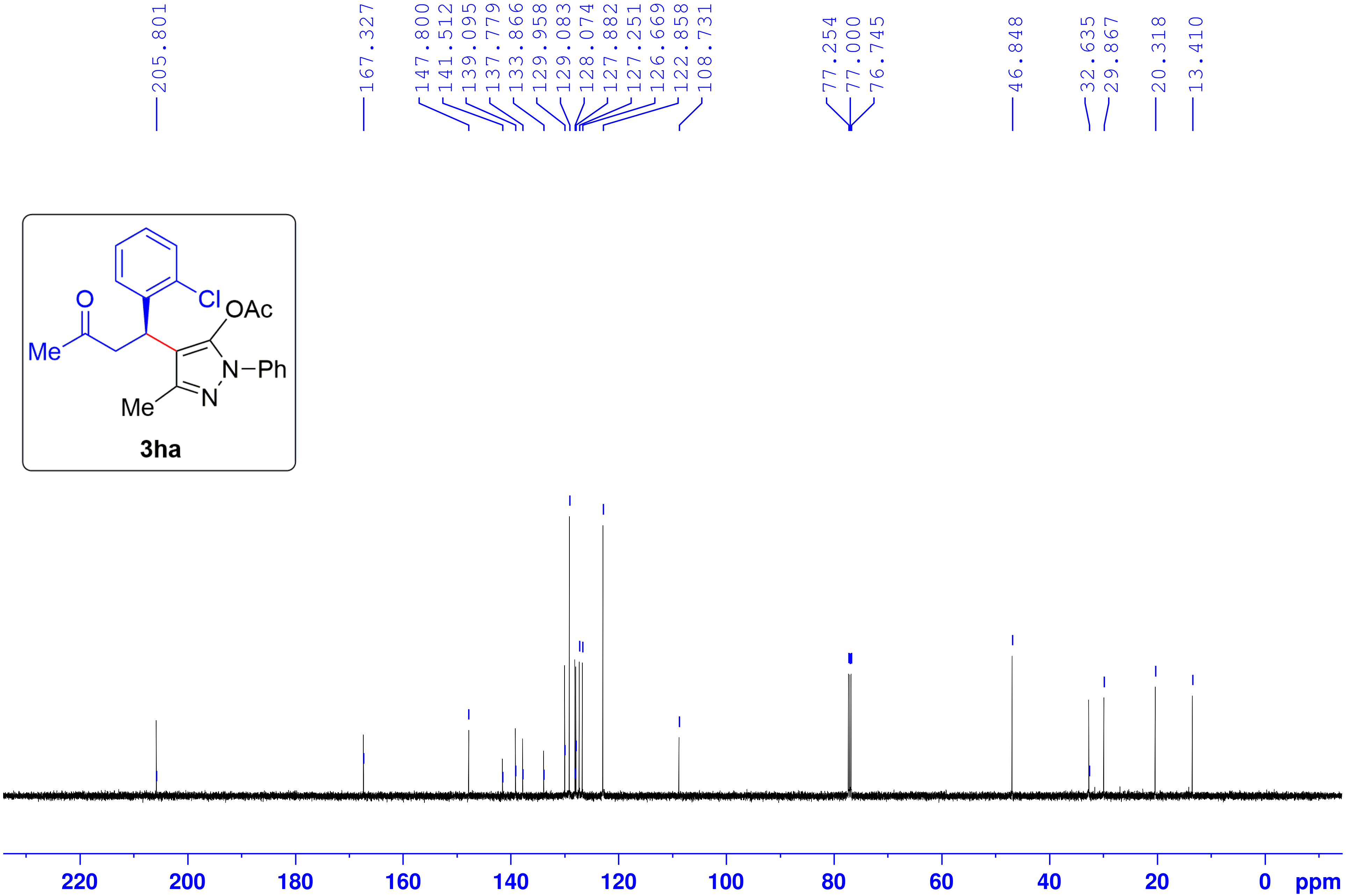
205.801
167.327
147.800
141.512
139.095
137.779
133.866
129.958
129.083
128.074
127.882
127.251
126.669
122.858
108.731
77.254
77.000
76.745
46.848
32.635
29.867
20.318
13.410
O
Cl
OAc
Me
N−Ph
N
Me
3ha
220
200
180
160
140
120
100
80
60
40
20
0
ppm

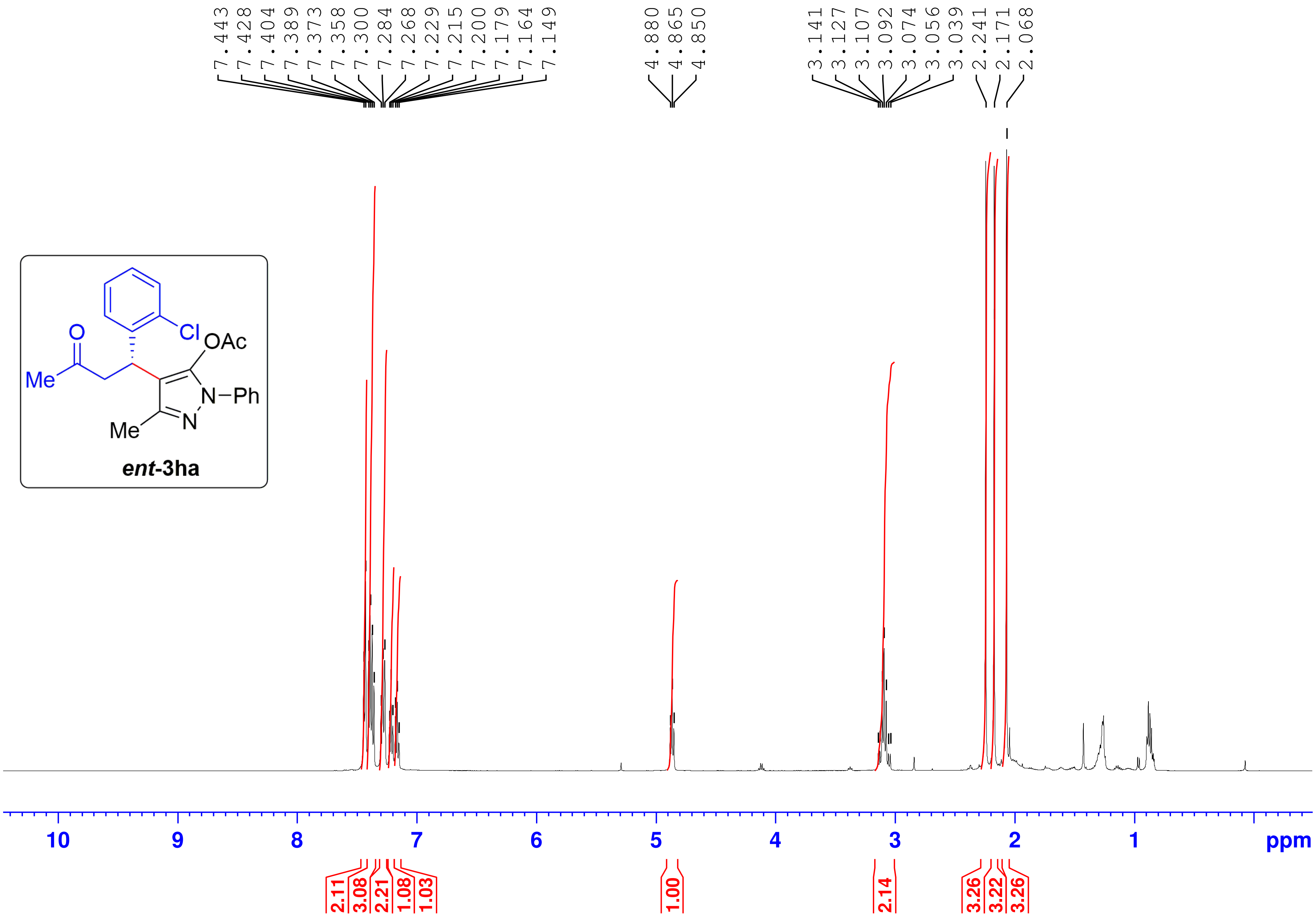
7.443
7.428
7.404
7.389
7.373
7.358
7.300
7.284
7.268
7.229
7.215
7.200
7.179
7.164
7.149
4.880
4.865
4.850
3.141
3.127
3.107
3.092
3.074
3.056
3.039
2.241
2.171
2.068
O
Cl
OAc
Me
N−Ph
N
Me
ent-3ha
10
9
8
7
6
5
4
3
2
1
ppm
2.11
3.08
2.21
1.08
1.03
1.00
2.14
3.26
3.22
3.26

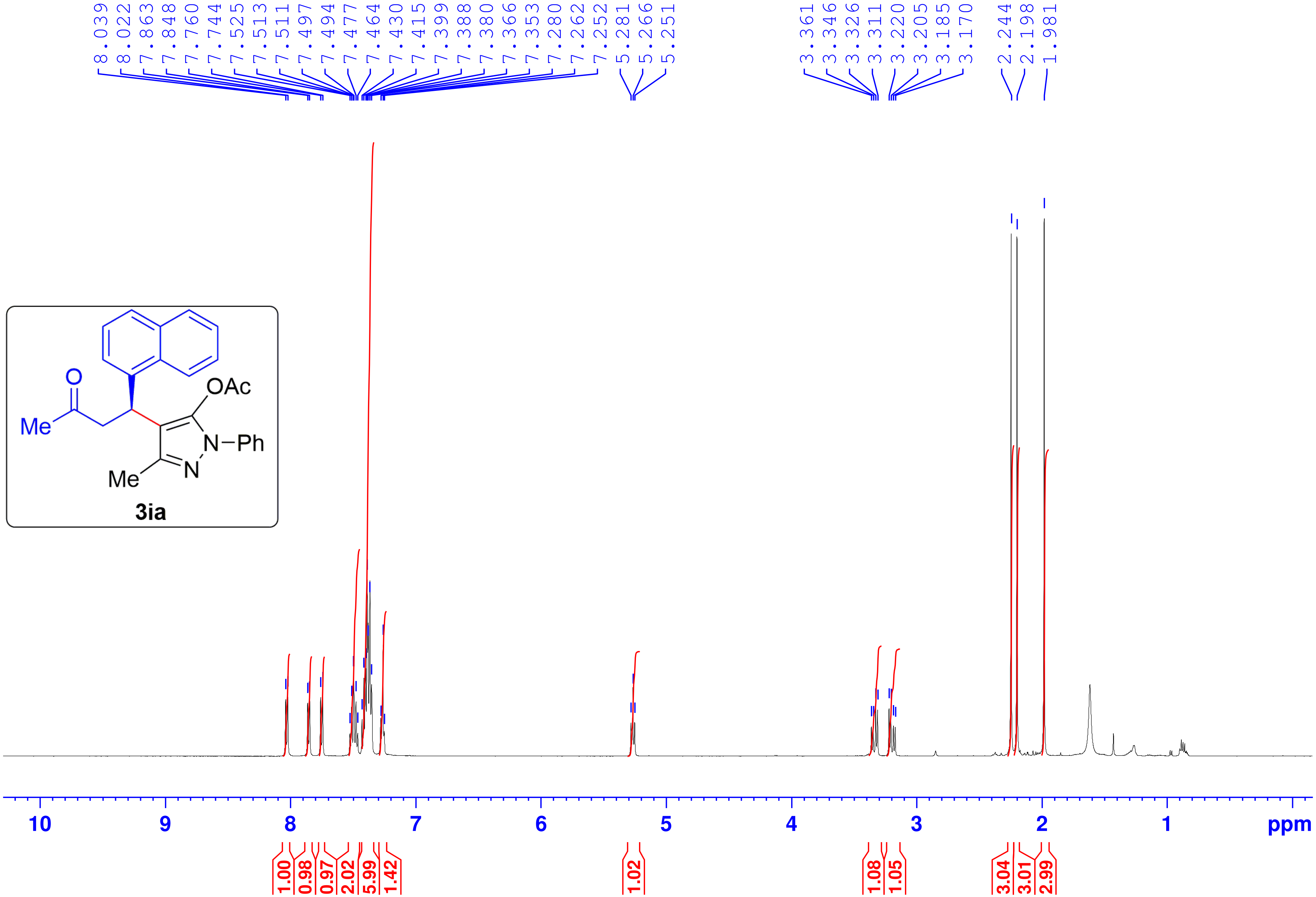
8.039
8.022
7.863
7.848
7.760
7.744
7.525
7.513
7.511
7.497
7.494
7.477
7.464
7.430
7.415
7.399
7.388
7.380
7.366
7.353
7.280
7.262
7.252
5.281
5.266
5.251
3.361
3.346
3.326
3.311
3.220
3.205
3.185
3.170
2.244
2.198
1.981
OAc
Me
N−Ph
N
Me
3ia
10
9
8
7
6
5
4
3
2
1
ppm
1.00
0.98
0.97
2.02
5.99
1.42
1.02
1.08
1.05
3.04
3.01
2.99

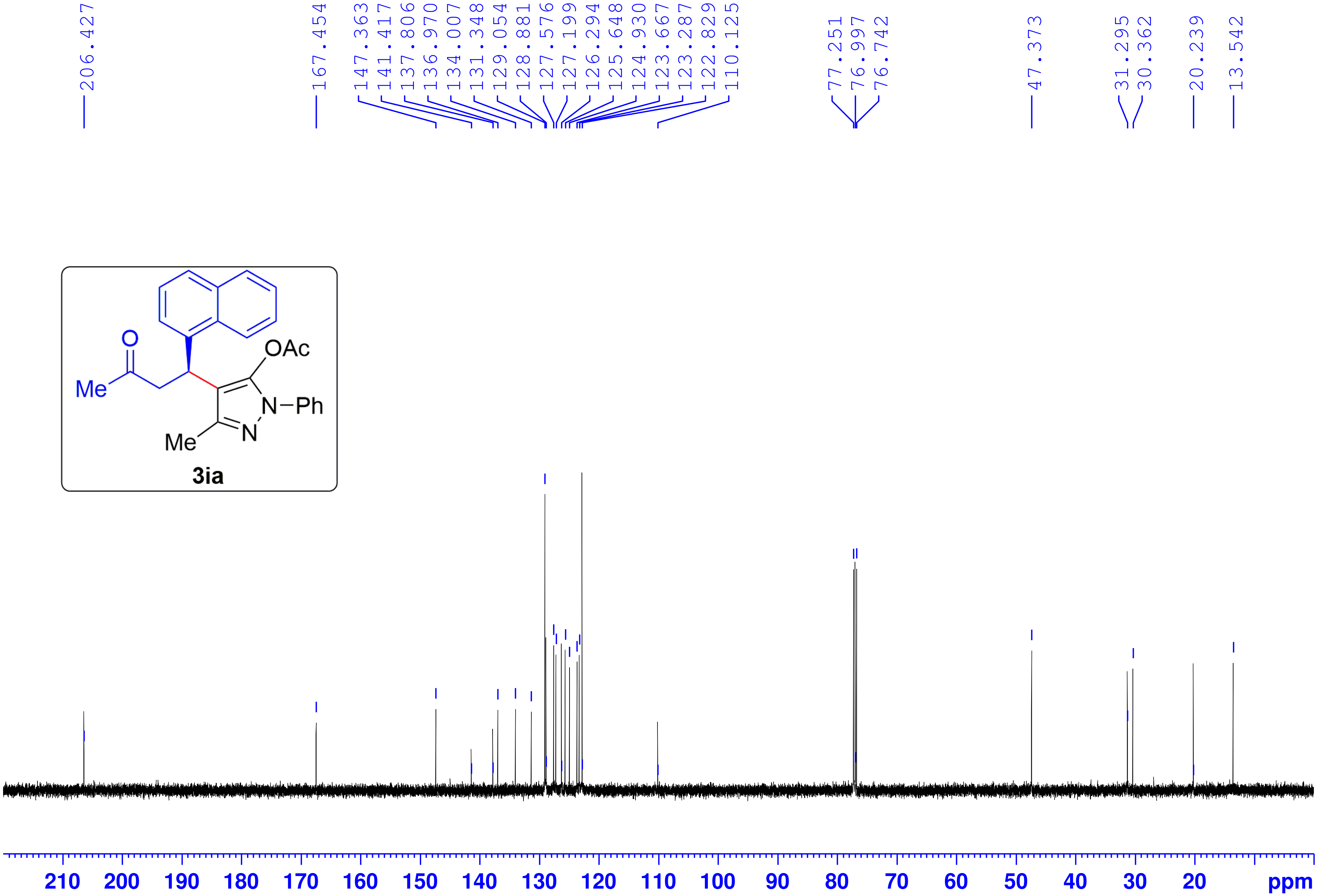

206.427
167.454
147.363
141.417
137.806
136.970
134.007
131.348
129.054
128.881
127.576
127.199
126.294
125.648
124.930
123.667
123.287
122.829
110.125
77.251
76.997
76.742
47.373
31.295
30.362
20.239
13.542
210
200
190
180
170
160
150
140
130
120
110
100
90
80
70
60
50
40
30
20
ppm
O
Me
OAc
N−Ph
N
Me

3ia

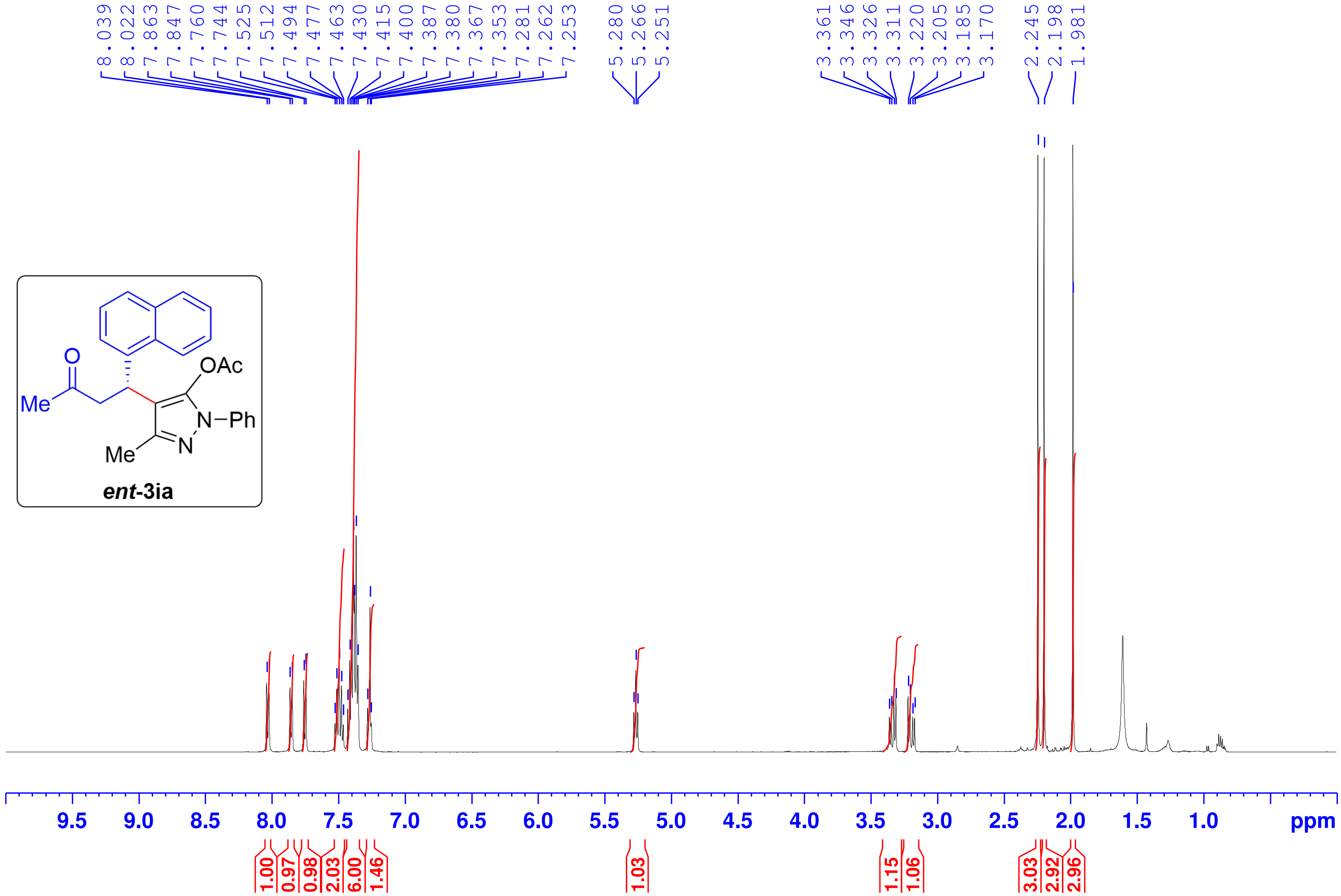
8.039
8.022
7.863
7.847
7.760
7.744
7.525
7.512
7.494
7.477
7.463
7.430
7.415
7.400
7.387
7.380
7.367
7.353
7.281
7.262
7.253
5.280
5.266
5.251
3.361
3.346
3.326
3.311
3.220
3.205
3.185
3.170
2.245
2.198
1.981
O
OAc
Me
N−Ph
N
Me
ent-3ia
9.5
9.0
8.5
8.0
7.5
7.0
6.5
6.0
5.5
5.0
4.5
4.0
3.5
3.0
2.5
2.0
1.5
1.0
ppm
1.00
0.97
0.98
2.03
6.00
1.46
1.03
1.15
1.06
3.03
2.92
2.96

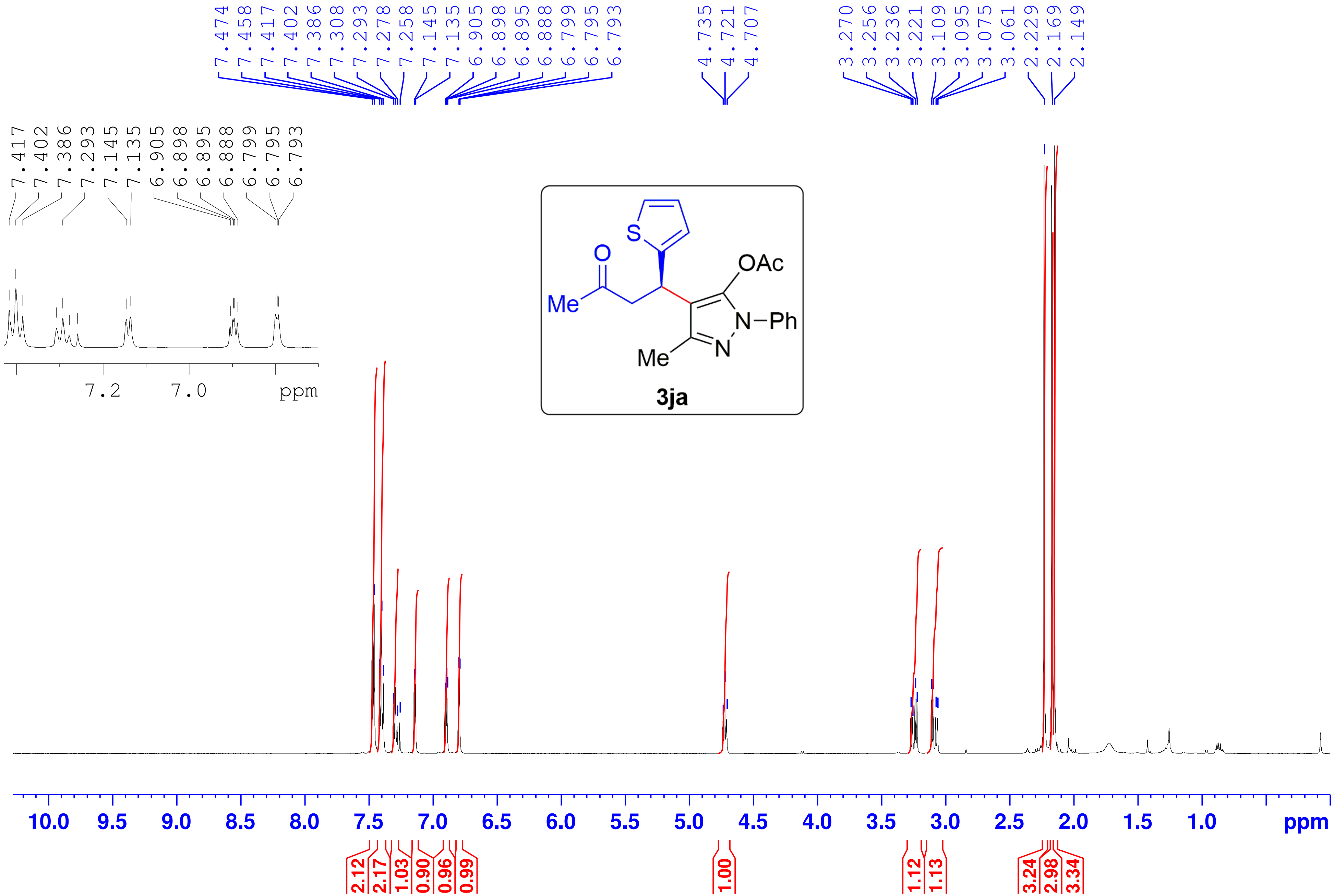

3ja

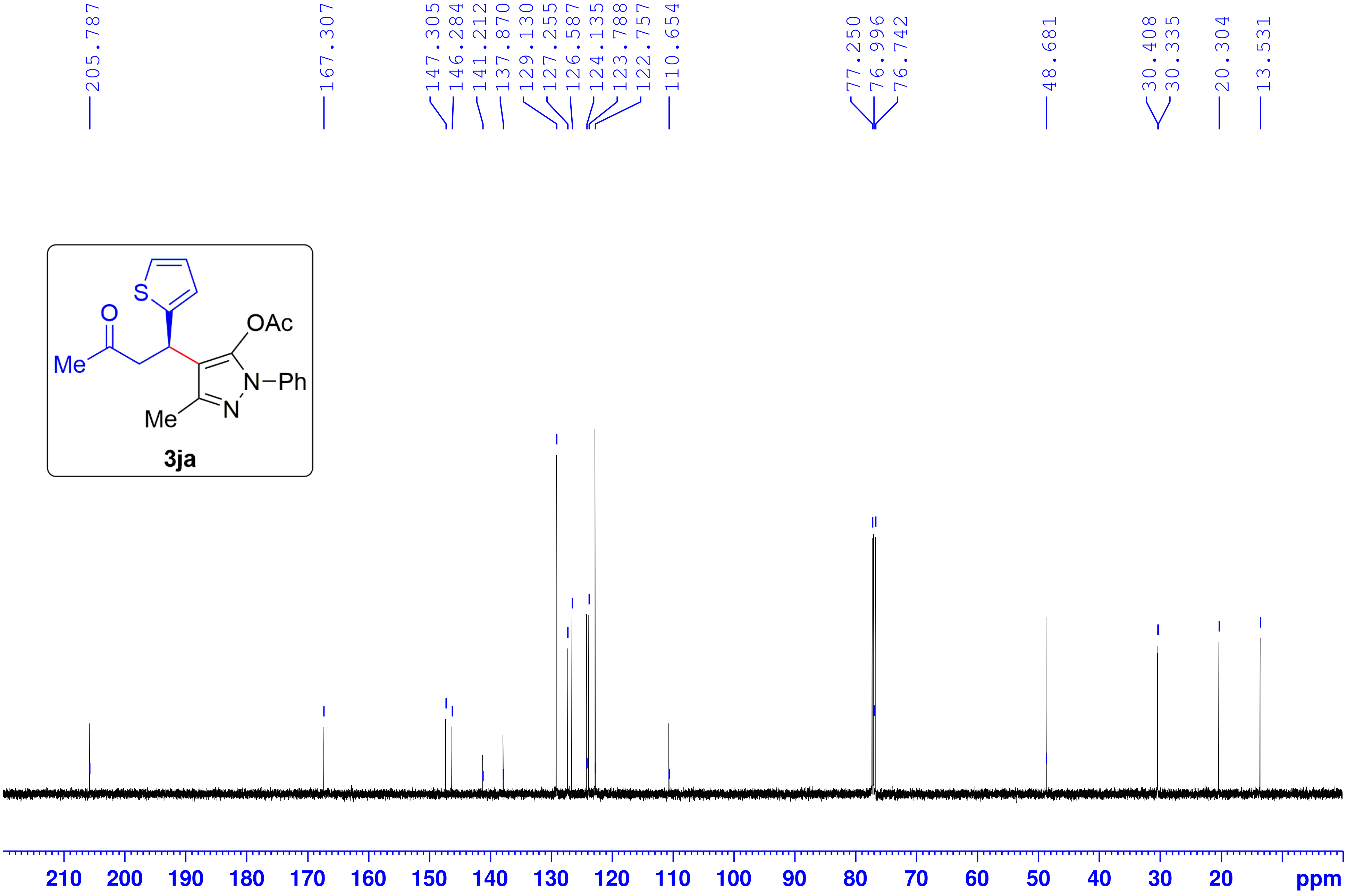
205.787
167.307
147.305
146.284
141.212
137.870
129.130
127.255
126.587
124.135
123.788
122.757
110.654
77.250
76.996
76.742
48.681
30.408
30.335
20.304
13.531
S
O
OAc
Me
N−Ph
N
Me
3ja
210 200 190 180 170 160 150 140 130 120 110 100 90 80 70 60 50 40 30 20 ppm

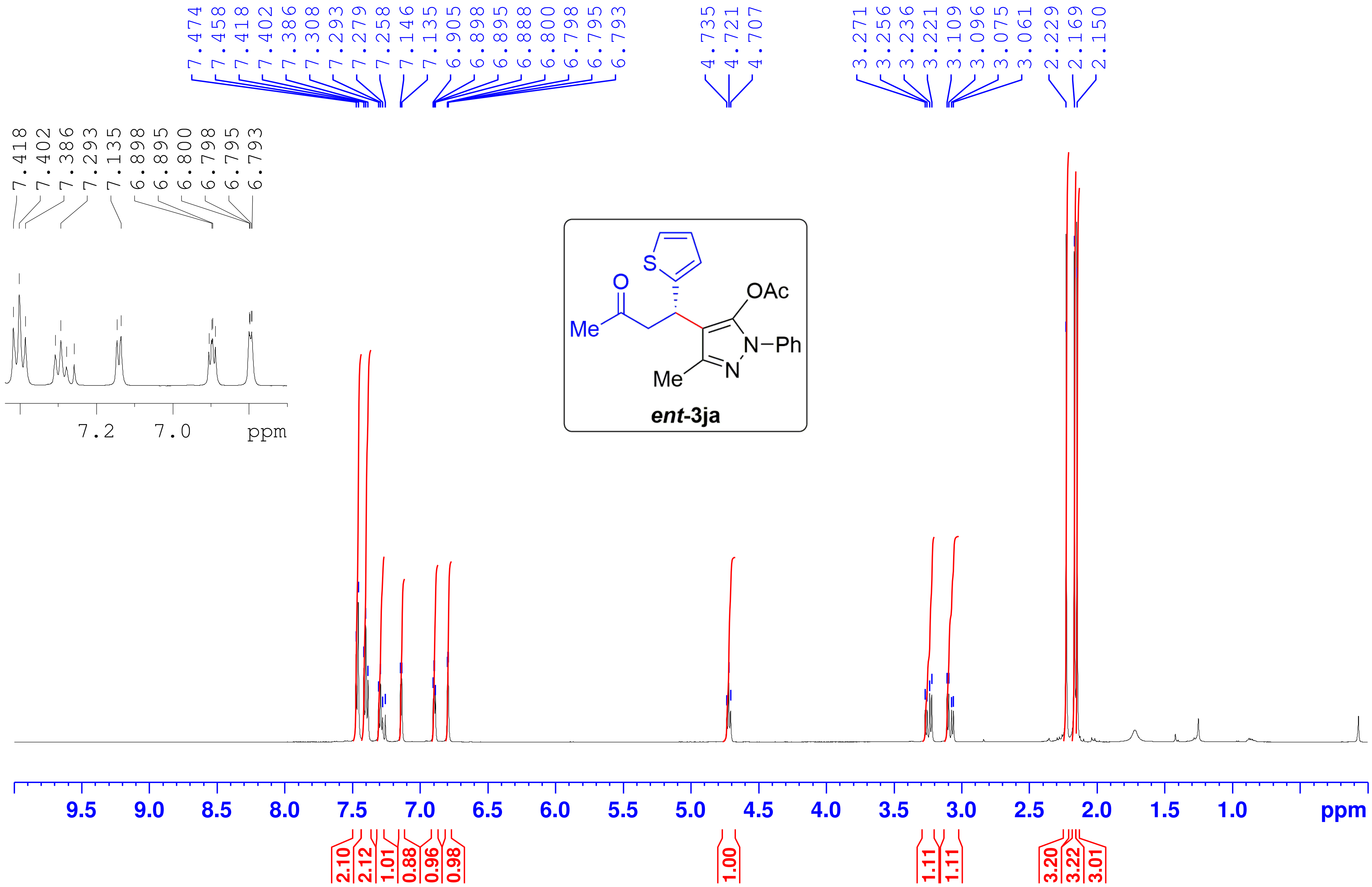

7.474 7.458 7.418 7.402 7.386 7.308 7.293 7.279 7.258 7.146 7.135 6.905 6.898 6.895 6.888 6.800 6.798 6.795 6.793
4.735 4.721 4.707
3.271 3.256 3.236 3.221 3.109 3.096 3.075 3.061 2.229 2.169 2.150
7.418 7.402 7.386 7.293 7.135 6.898 6.895 6.800 6.798 6.795 6.793
7.2
7.0
ppm
O
S
OAc
Me
N−Ph
Me
N
ent-3ja
9.5
9.0
8.5
8.0
7.5
7.0
6.5
6.0
5.5
5.0
4.5
4.0
3.5
3.0
2.5
2.0
1.5
1.0
ppm
2.10 2.12 1.01 0.88 0.96 0.98
1.00
1.11 1.11
3.20 3.22 3.01

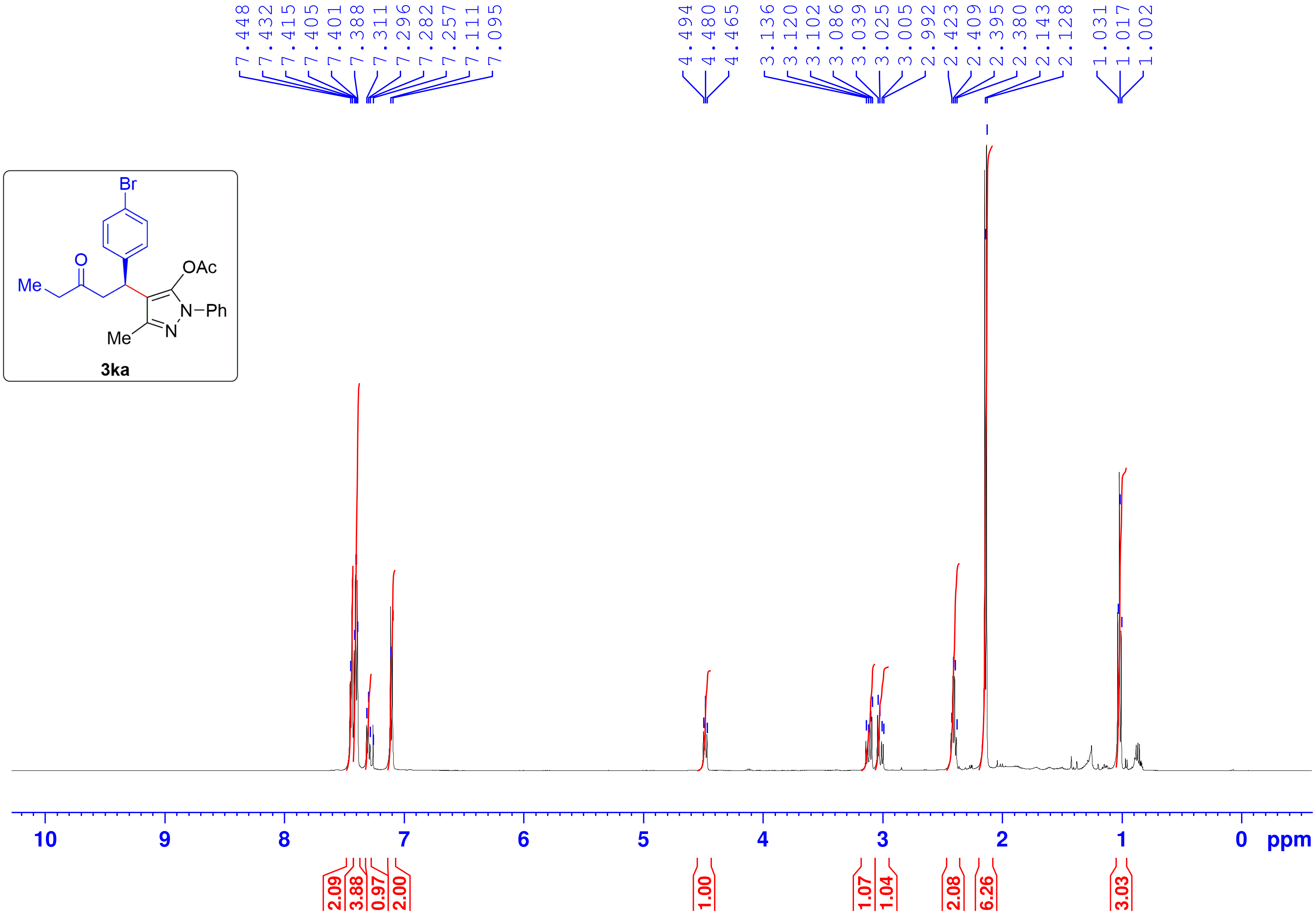

7.448
7.432
7.415
7.405
7.401
7.388
7.311
7.296
7.282
7.257
7.111
7.095
4.494
4.480
4.465
3.136
3.120
3.102
3.086
3.039
3.025
3.005
2.992
2.423
2.409
2.395
2.380
2.143
2.128
1.031
1.017
1.002
Br
O
OAc
Me
N–Ph
N
Me
3ka
10
9
8
7
6
5
4
3
2
1
0
ppm
2.09
3.88
0.97
2.00
1.00
1.07
1.04
2.08
6.26
3.03

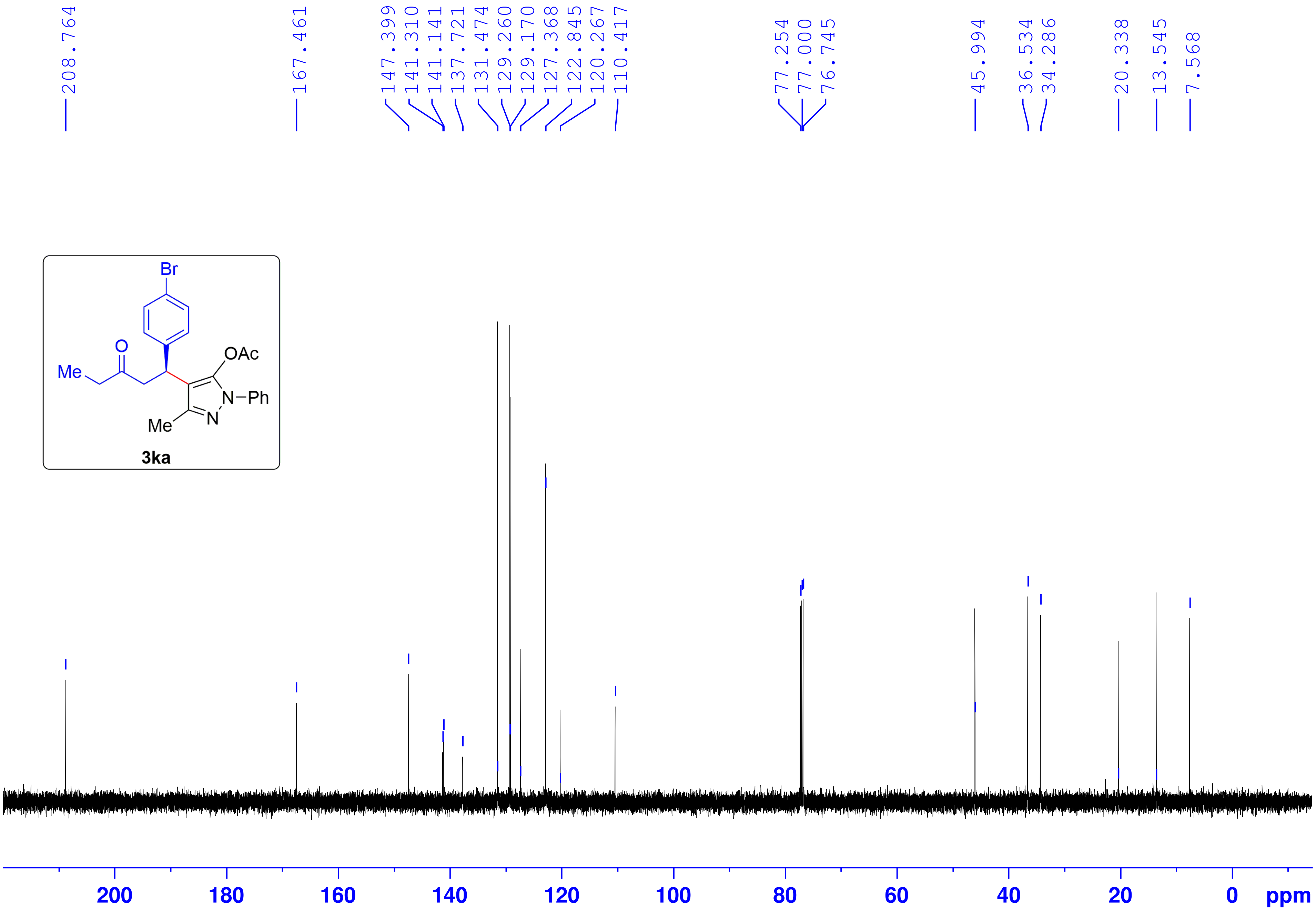
208.764
167.461
147.399
141.310
141.141
137.721
131.474
129.260
129.170
127.368
122.845
120.267
110.417
77.254
77.000
76.745
45.994
36.534
34.286
20.338
13.545
7.568
Br
O
Me
OAc
N−Ph
N
Me
3ka
200
180
160
140
120
100
80
60
40
20
0
ppm

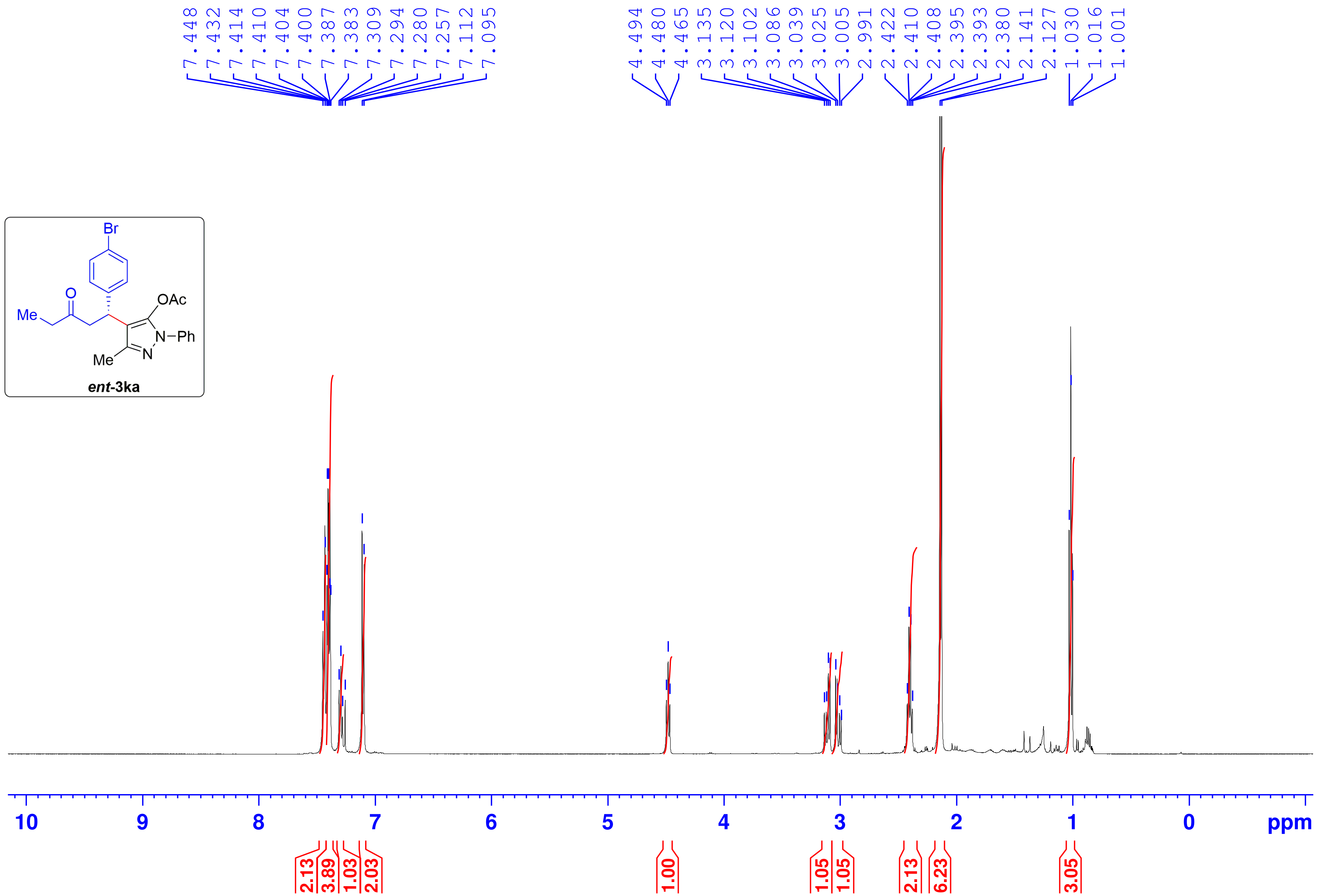
7.448 7.432 7.414 7.410 7.404 7.400 7.387 7.383 7.309 7.294 7.280 7.257 7.112 7.095
4.494 4.480 4.465 3.135 3.120 3.102 3.086 3.039 3.025 3.005 2.991 2.422 2.410 2.408 2.395 2.393 2.380 2.141 2.127 1.030 1.016 1.001
Br
O
OAc
Me
N−Ph
N
Me
ent-3ka
2.13 3.89 1.03 2.03 1.00 1.05 1.05 2.13 6.23 3.05
10 9 8 7 6 5 4 3 2 1 0 ppm

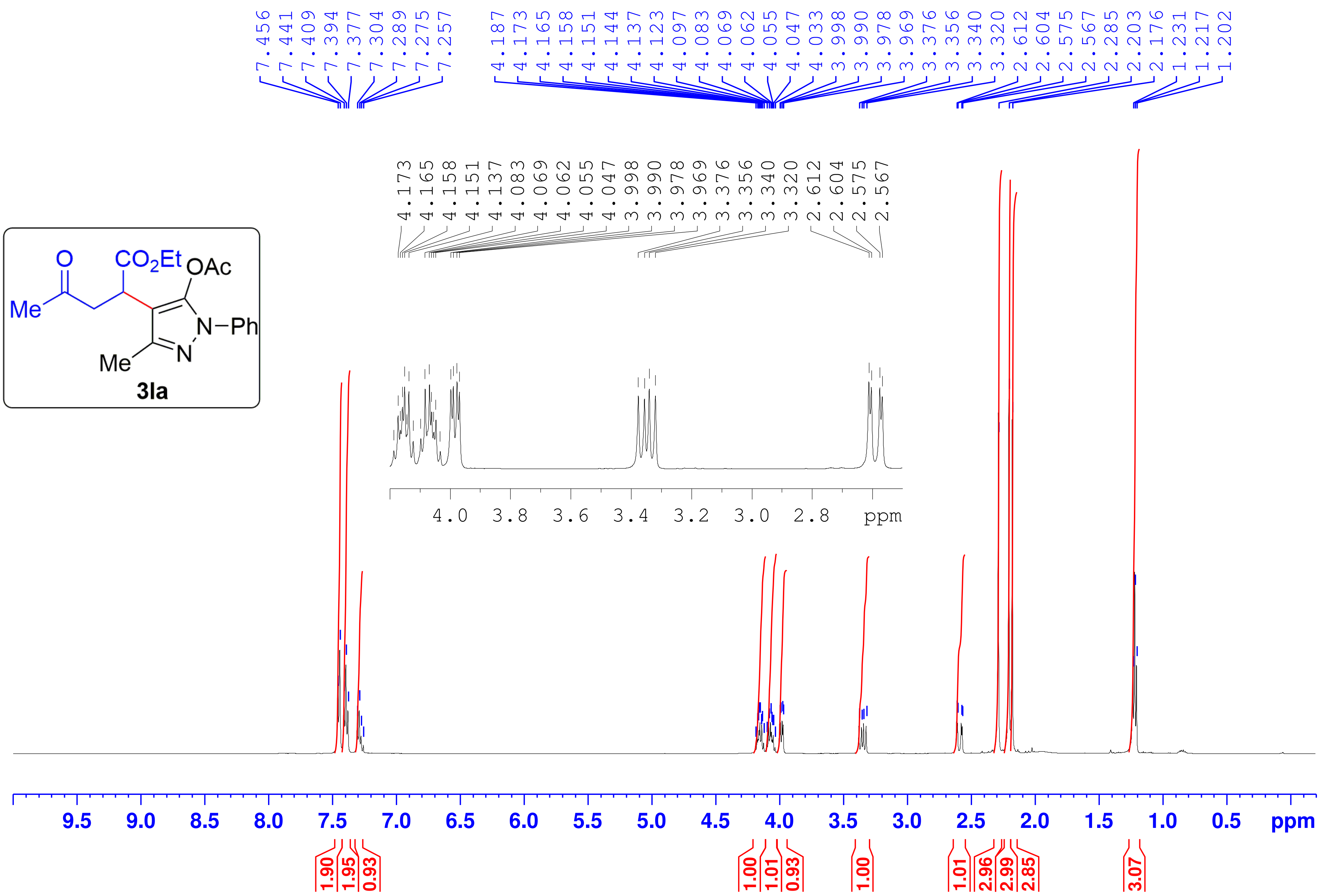

7.456
7.441
7.409
7.394
7.377
7.304
7.289
7.275
7.257
4.187
4.173
4.165
4.158
4.151
4.144
4.137
4.123
4.097
4.083
4.069
4.062
4.055
4.047
4.033
3.998
3.990
3.978
3.969
3.376
3.356
3.340
3.320
2.612
2.604
2.575
2.567
2.285
2.203
2.176
1.231
1.217
1.202
O
CO_2Et
OAc
Me
N–Ph
N
Me
3la
ppm
1.90
1.95
0.93
1.00
1.01
0.93
1.00
1.01
2.96
2.99
2.85
3.07

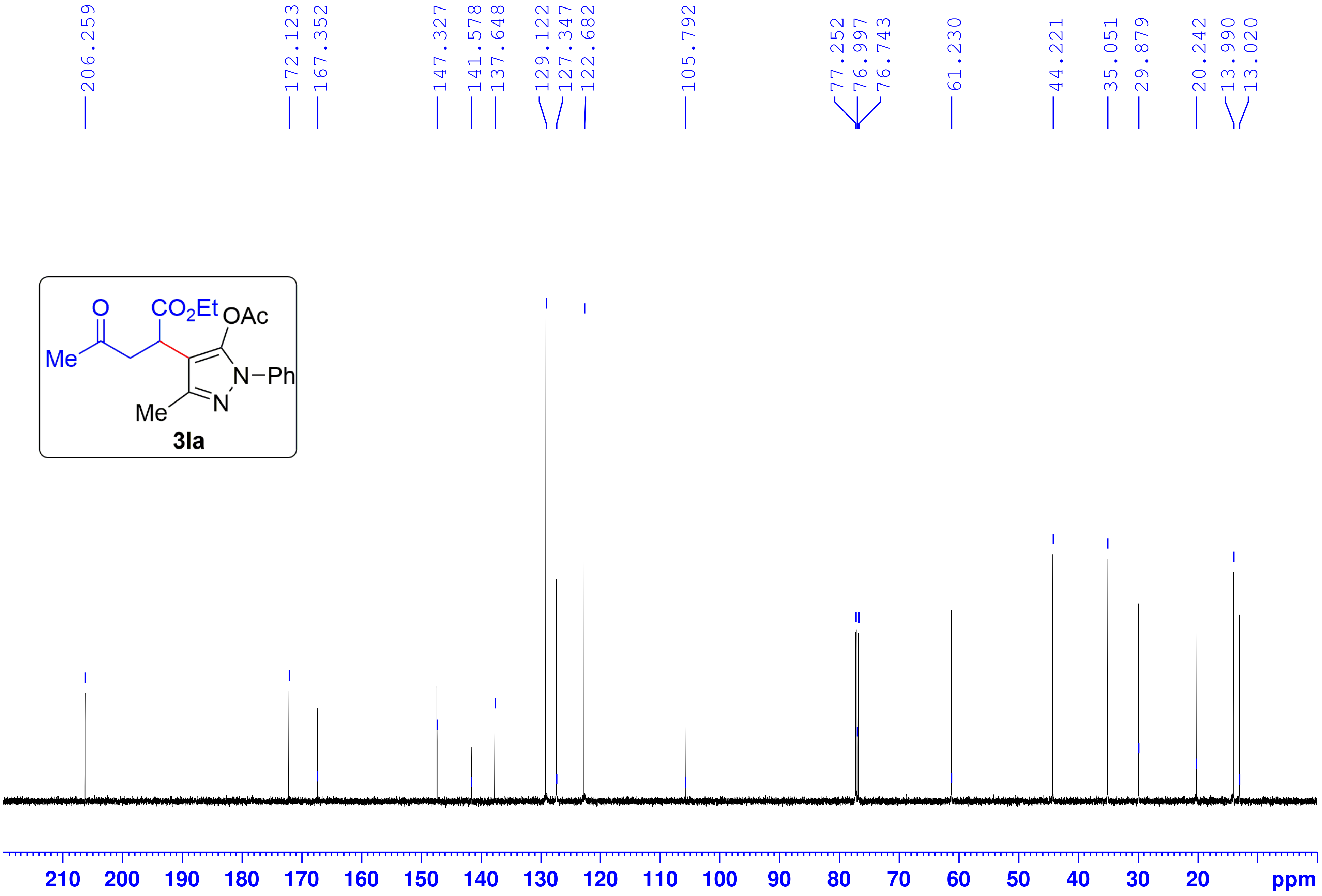
206.259
172.123
167.352
147.327
141.578
137.648
129.122
127.347
122.682
105.792
77.252
76.997
76.743
61.230
44.221
35.051
29.879
20.242
13.990
13.020
O
CO_2Et
OAc
Me
N−Ph
N
Me
3la
210
200
190
180
170
160
150
140
130
120
110
100
90
80
70
60
50
40
30
20
ppm

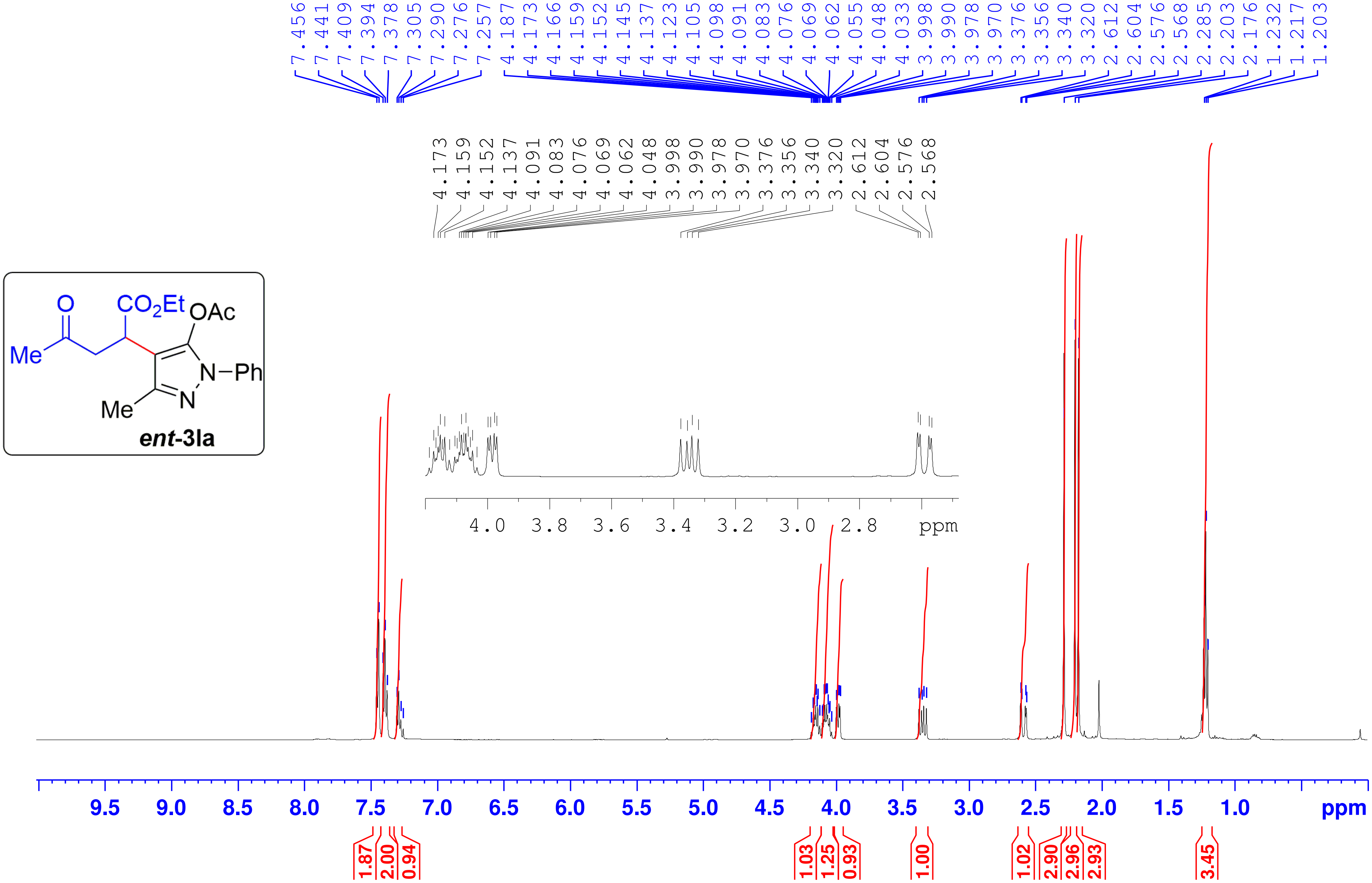

7.456
7.441
7.409
7.394
7.378
7.305
7.290
7.276
7.257
4.187
4.173
4.166
4.159
4.152
4.145
4.137
4.123
4.105
4.098
4.091
4.083
4.076
4.069
4.062
4.055
4.048
4.033
3.998
3.990
3.978
3.970
3.376
3.356
3.340
3.320
2.612
2.604
2.576
2.568
2.285
2.203
2.176
1.232
1.217
1.203
ent-3la
ppm
1.87
2.00
0.94
1.03
1.25
0.93
1.00
1.02
2.90
2.96
2.93
3.45

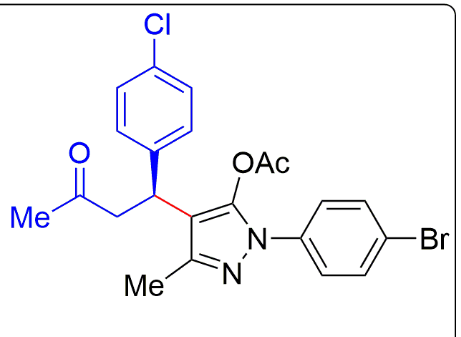

3cb

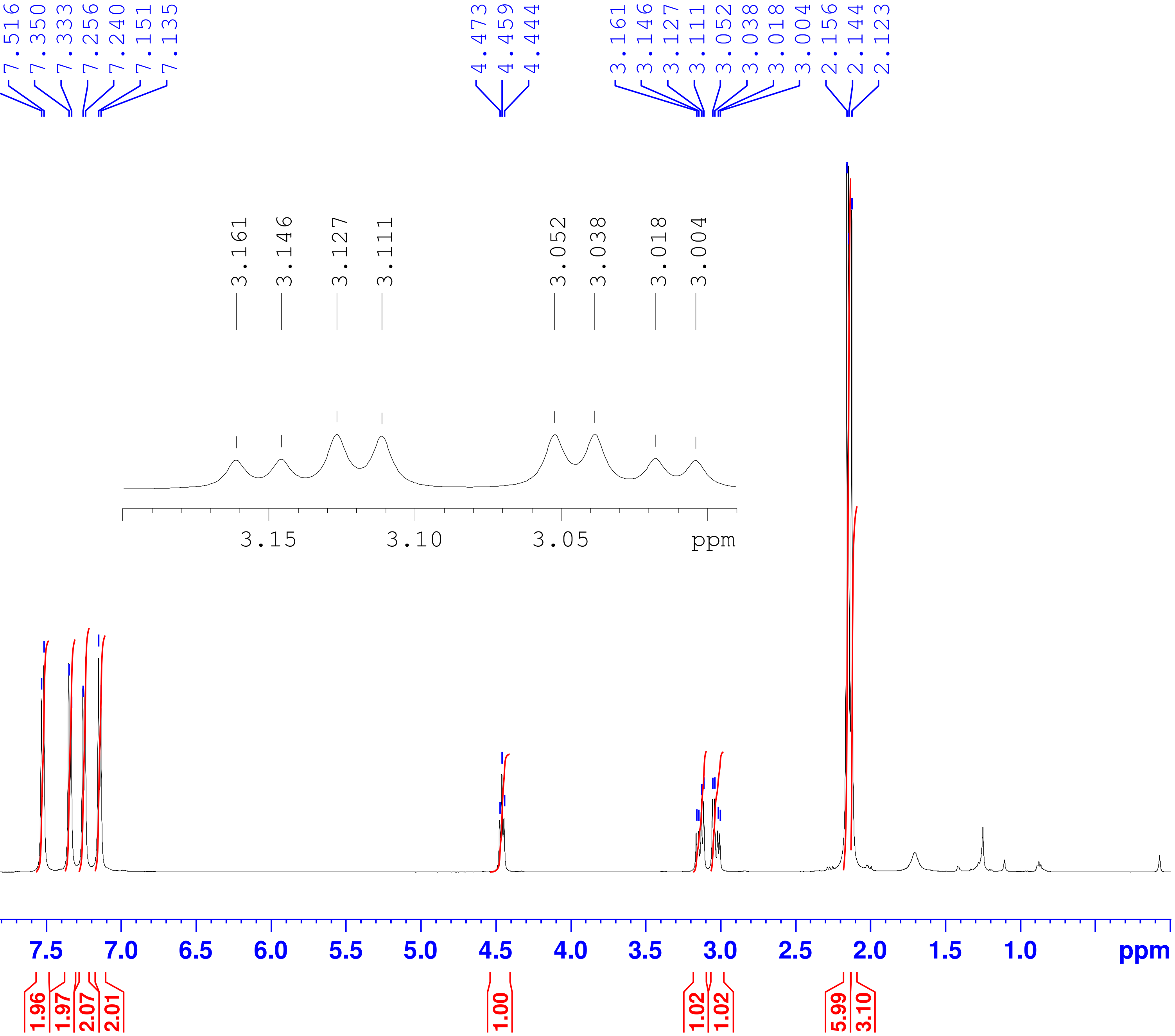

7.533
7.516
7.350
7.333
7.256
7.240
7.151
7.135
4.473
4.459
4.444
3.161
3.146
3.127
3.111
3.052
3.038
3.018
3.004
2.156
2.144
2.123
3.15
3.10
3.05
ppm
10.0
9.5
9.0
8.5
8.0
7.5
7.0
6.5
6.0
5.5
5.0
4.5
4.0
3.5
3.0
2.5
2.0
1.5
1.0
ppm
1.96
1.97
2.07
2.01
1.00
1.02
1.02
5.99
3.10

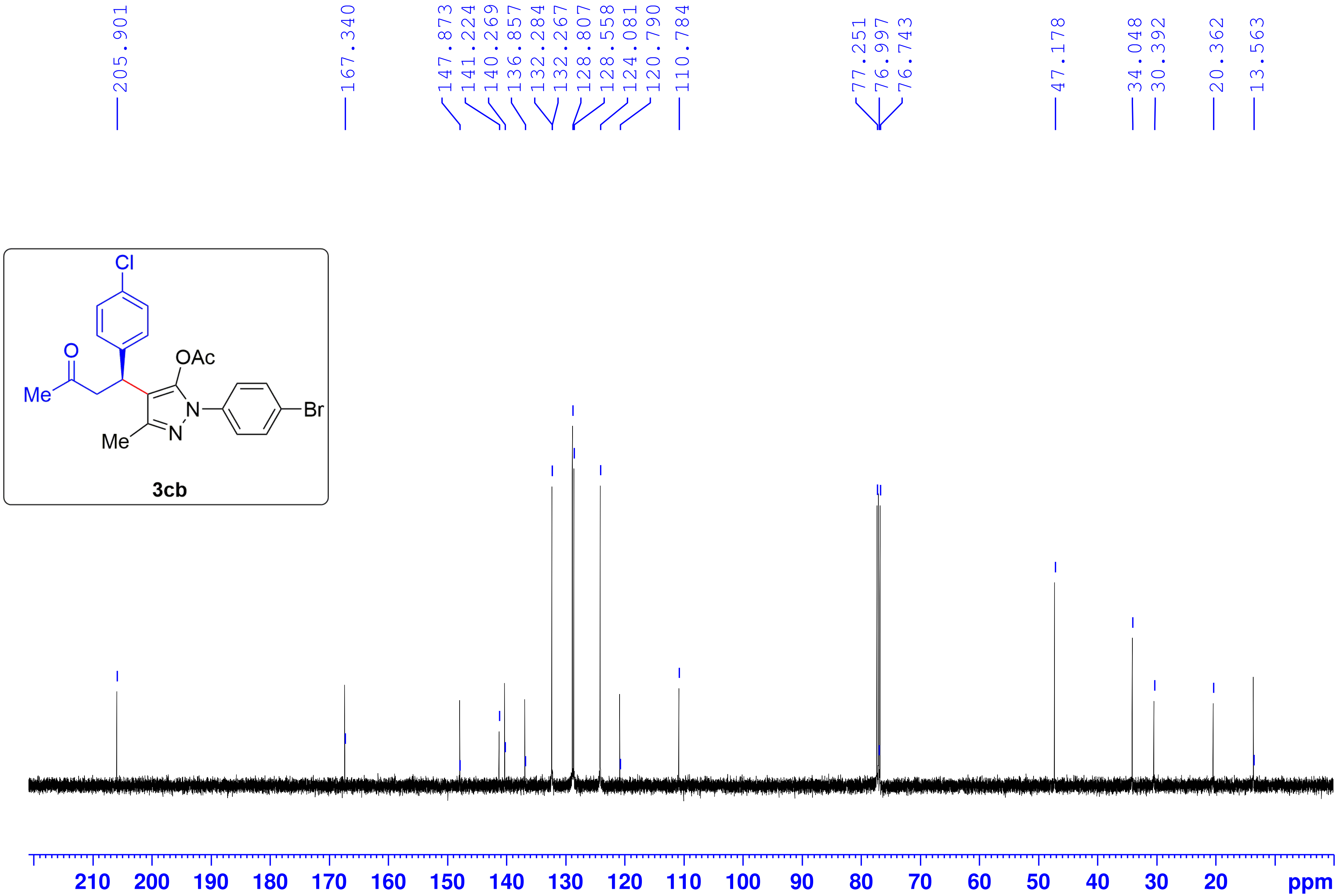
205.901
167.340
147.873
141.224
140.269
136.857
132.284
132.267
128.807
128.558
124.081
120.790
110.784
77.251
76.997
76.743
47.178
34.048
30.392
20.362
13.563
Cl
O
OAc
Me
N
Br
Me
N
3cb
210
200
190
180
170
160
150
140
130
120
110
100
90
80
70
60
50
40
30
20
ppm

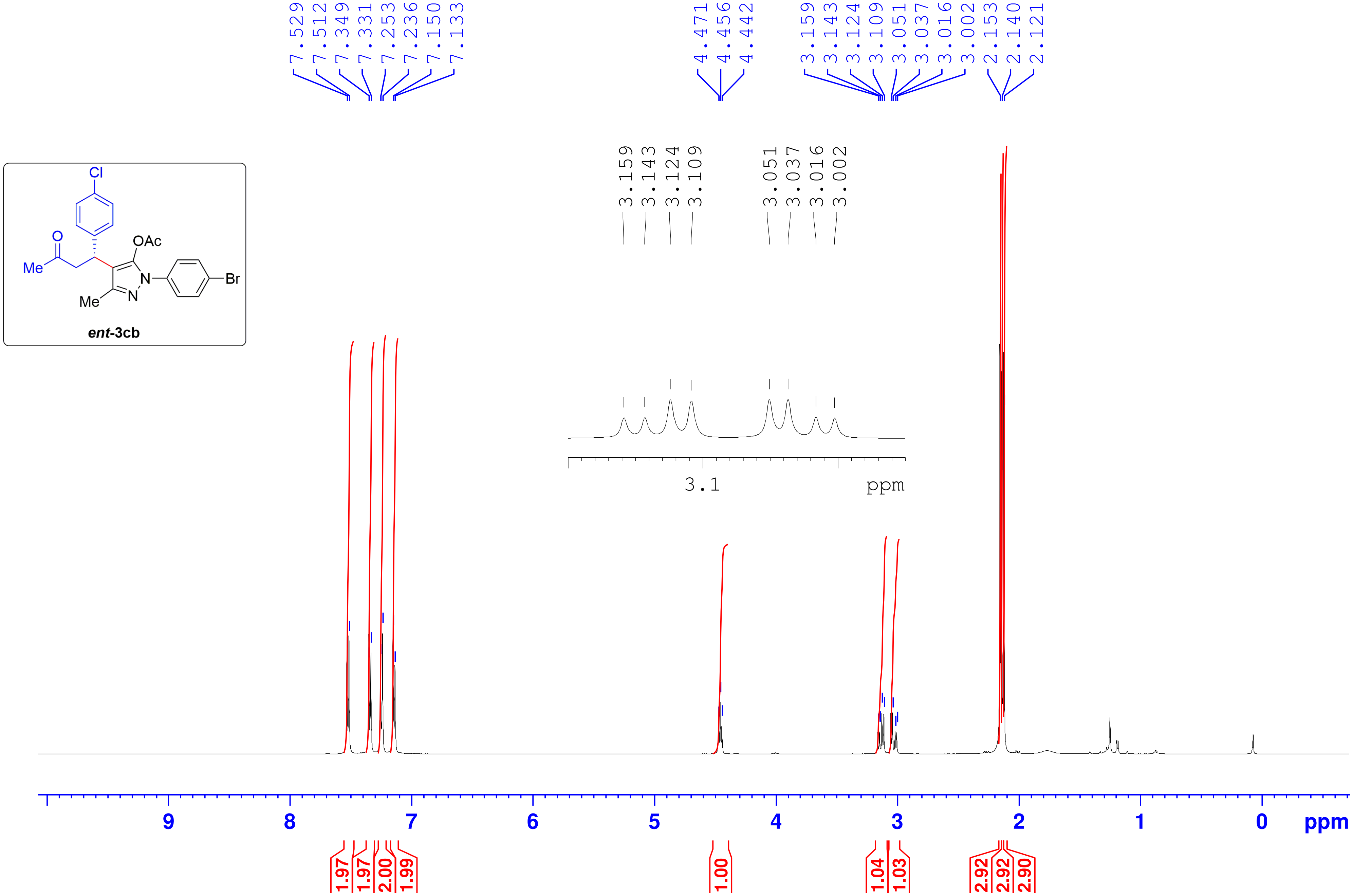
Cl
O
OAc
Me
N
Br
Me
N
ent-3cb
7.529
7.512
7.349
7.331
7.253
7.236
7.150
7.133
4.471
4.456
4.442
3.159
3.143
3.124
3.109
3.051
3.037
3.016
3.002
2.153
2.140
2.121
3.1
ppm
9
8
7
6
5
4
3
2
1
0
ppm
1.97
1.97
2.00
1.99
1.00
1.04
1.03
2.92
2.92
2.90

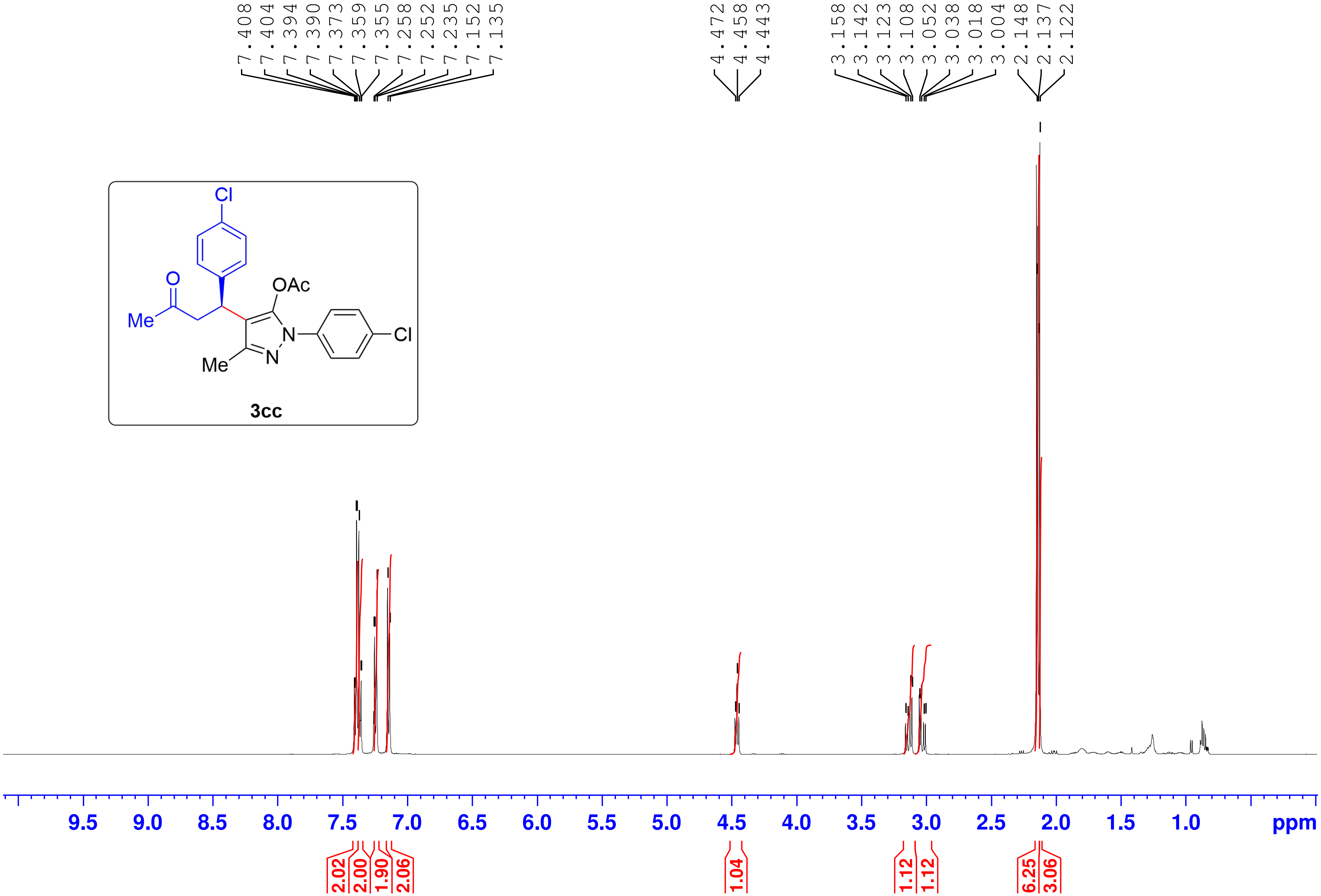

7.408
7.404
7.394
7.390
7.373
7.359
7.355
7.258
7.252
7.235
7.152
7.135
4.472
4.458
4.443
3.158
3.142
3.123
3.108
3.052
3.038
3.018
3.004
2.148
2.137
2.122
Cl
O
OAc
Me
N
N
Cl
Me
9.5
9.0
8.5
8.0
7.5
7.0
6.5
6.0
5.5
5.0
4.5
4.0
3.5
3.0
2.5
2.0
1.5
1.0
ppm
2.02
2.00
1.90
2.06
1.04
1.12
1.12
6.25
3.06

3cc

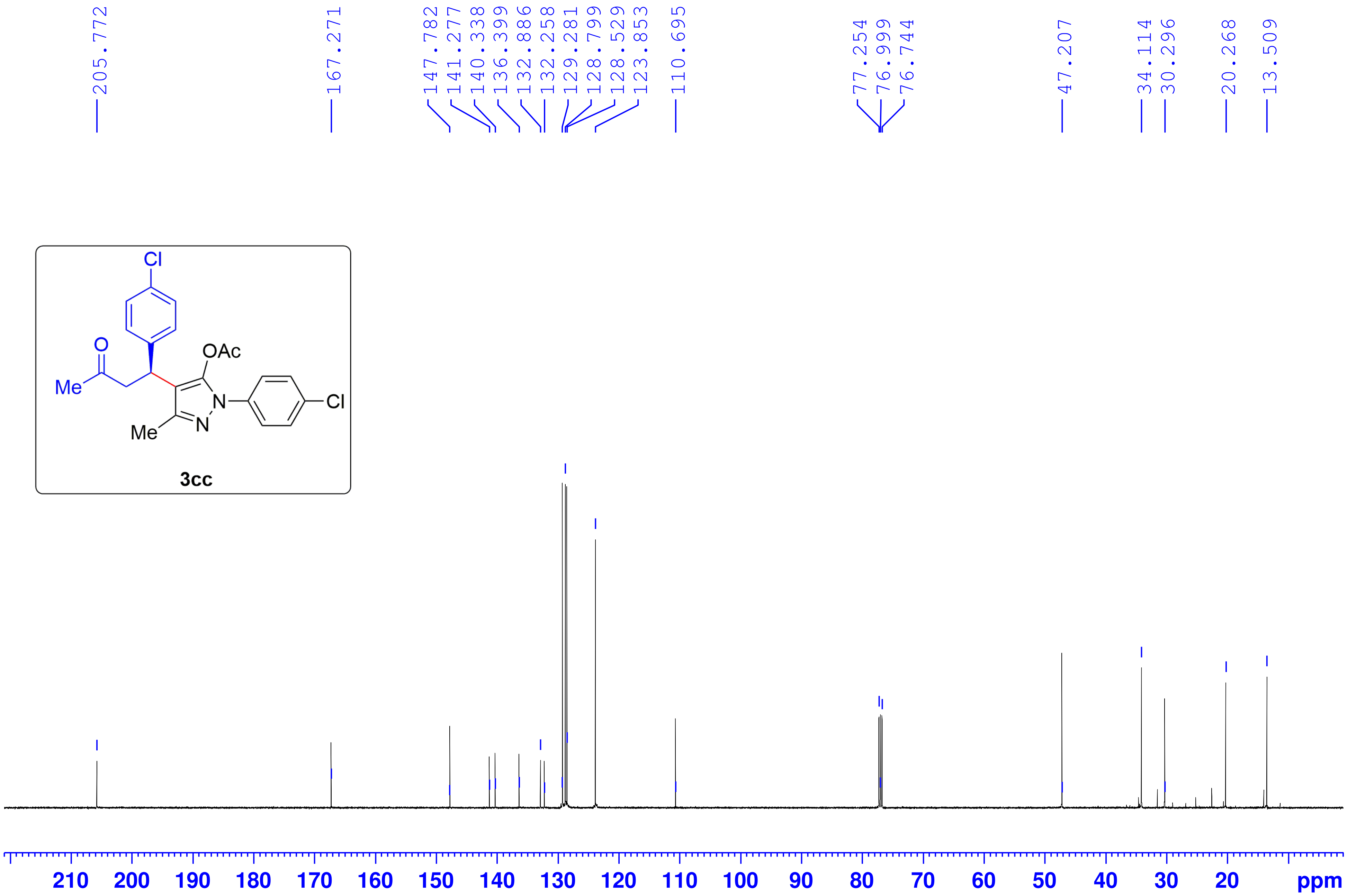
205.772
167.271
147.782
141.277
140.338
136.399
132.886
132.258
129.281
128.799
128.529
123.853
110.695
77.254
76.999
76.744
47.207
34.114
30.296
20.268
13.509
Cl
O
OAc
Me
N
Cl
Me
N
3cc
210
200
190
180
170
160
150
140
130
120
110
100
90
80
70
60
50
40
30
20
ppm

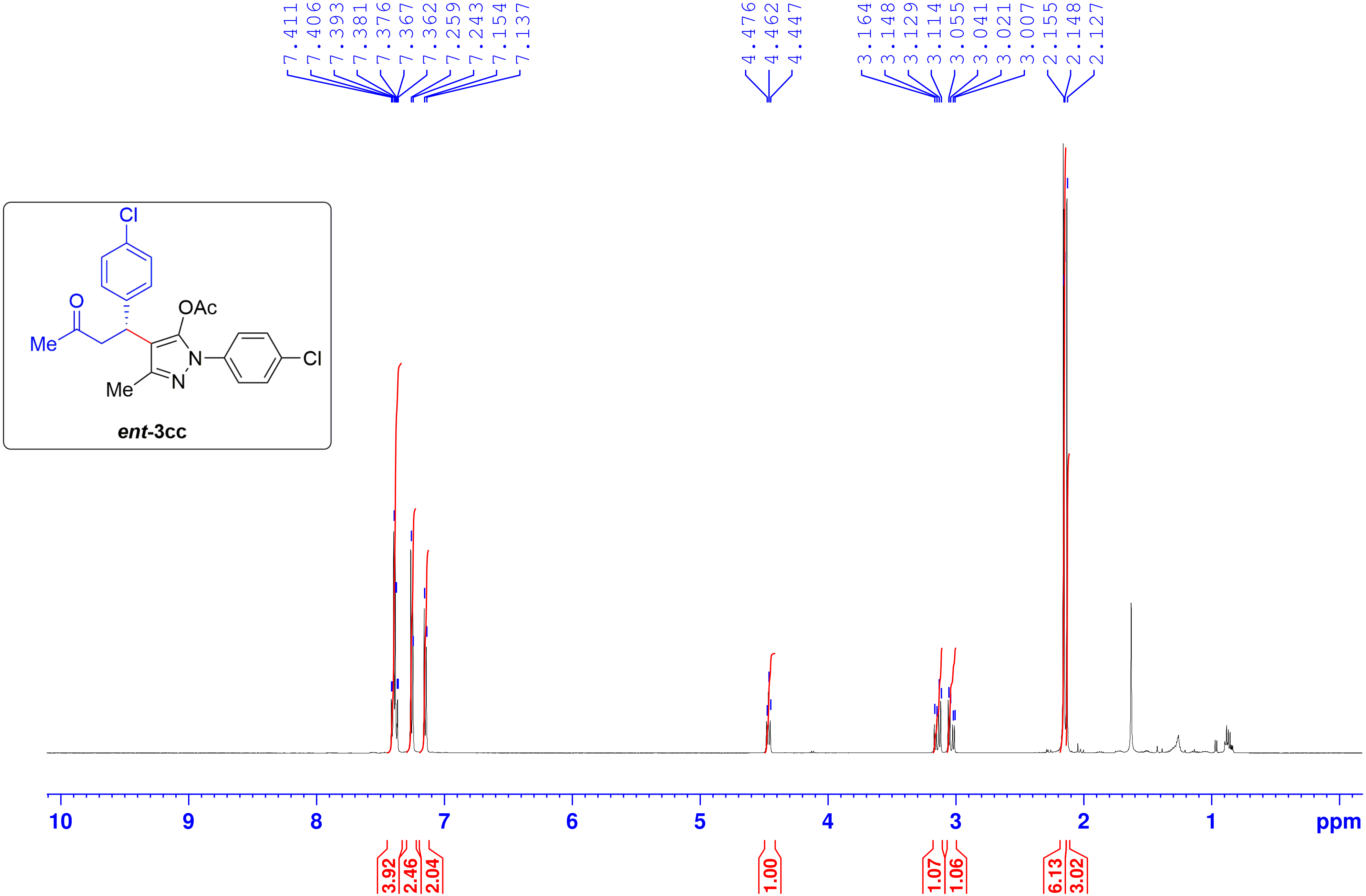

ent-**3cc**

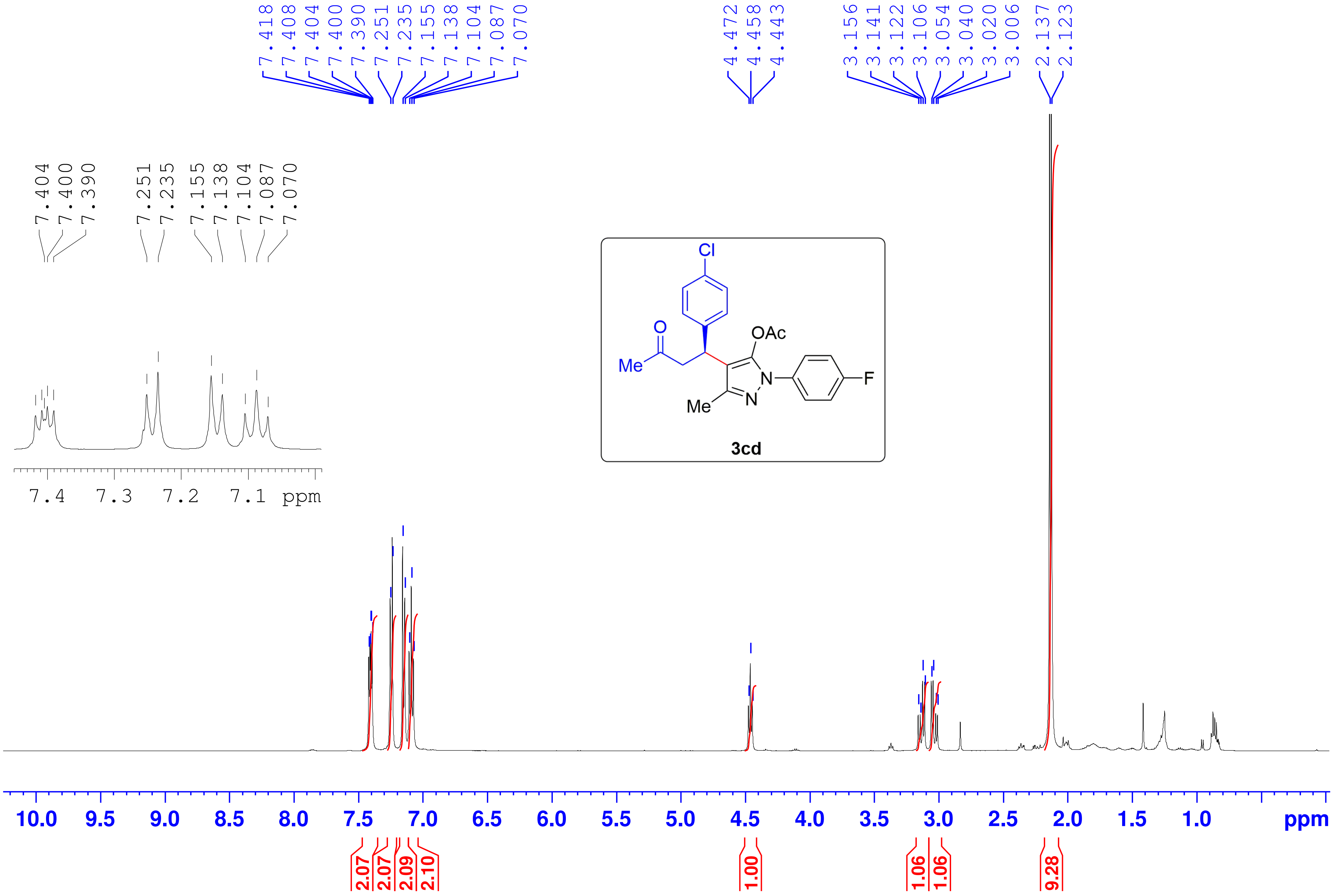

7.418 7.408 7.404 7.400 7.390 7.251 7.235 7.155 7.138 7.104 7.087 7.070
4.472 4.458 4.443
3.156 3.141 3.122 3.106 3.054 3.040 3.020 3.006
2.137 2.123
7.404 7.400 7.390 7.251 7.235 7.155 7.138 7.104 7.087 7.070
7.4 7.3 7.2 7.1 ppm
Cl
O
Me
OAc
N
F
Me
N
3cd
10.0 9.5 9.0 8.5 8.0 7.5 7.0 6.5 6.0 5.5 5.0 4.5 4.0 3.5 3.0 2.5 2.0 1.5 1.0 ppm
2.07 2.07 2.09 2.10 1.00 1.06 1.06 9.28

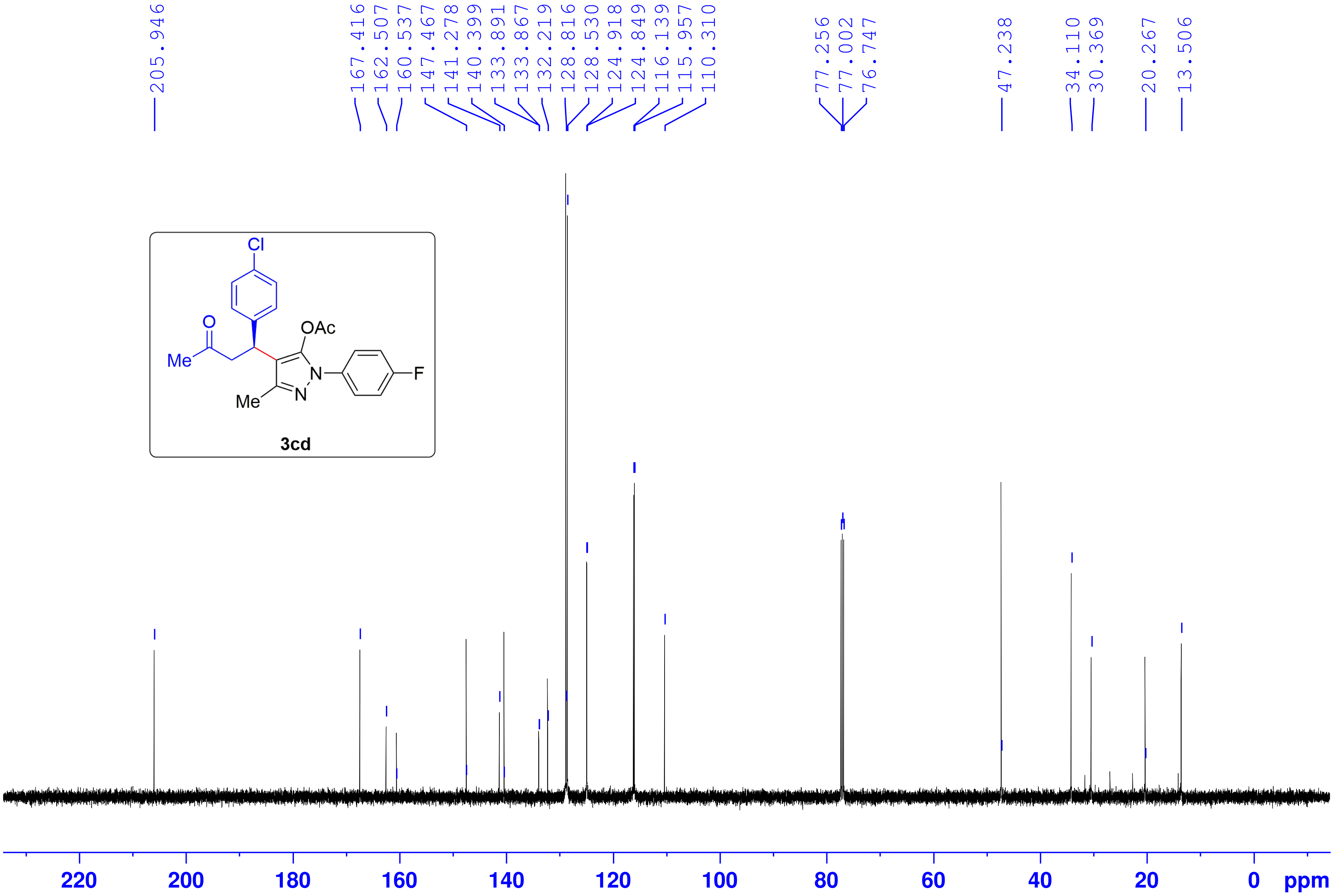

205.946
167.416
162.507
160.537
147.467
141.278
140.399
133.891
133.867
132.219
128.816
128.530
124.918
124.849
116.139
115.957
110.310
77.256
77.002
76.747
47.238
34.110
30.369
20.267
13.506
Cl
O
OAc
Me
N
F
Me
N
3cd
220
200
180
160
140
120
100
80
60
40
20
0
ppm

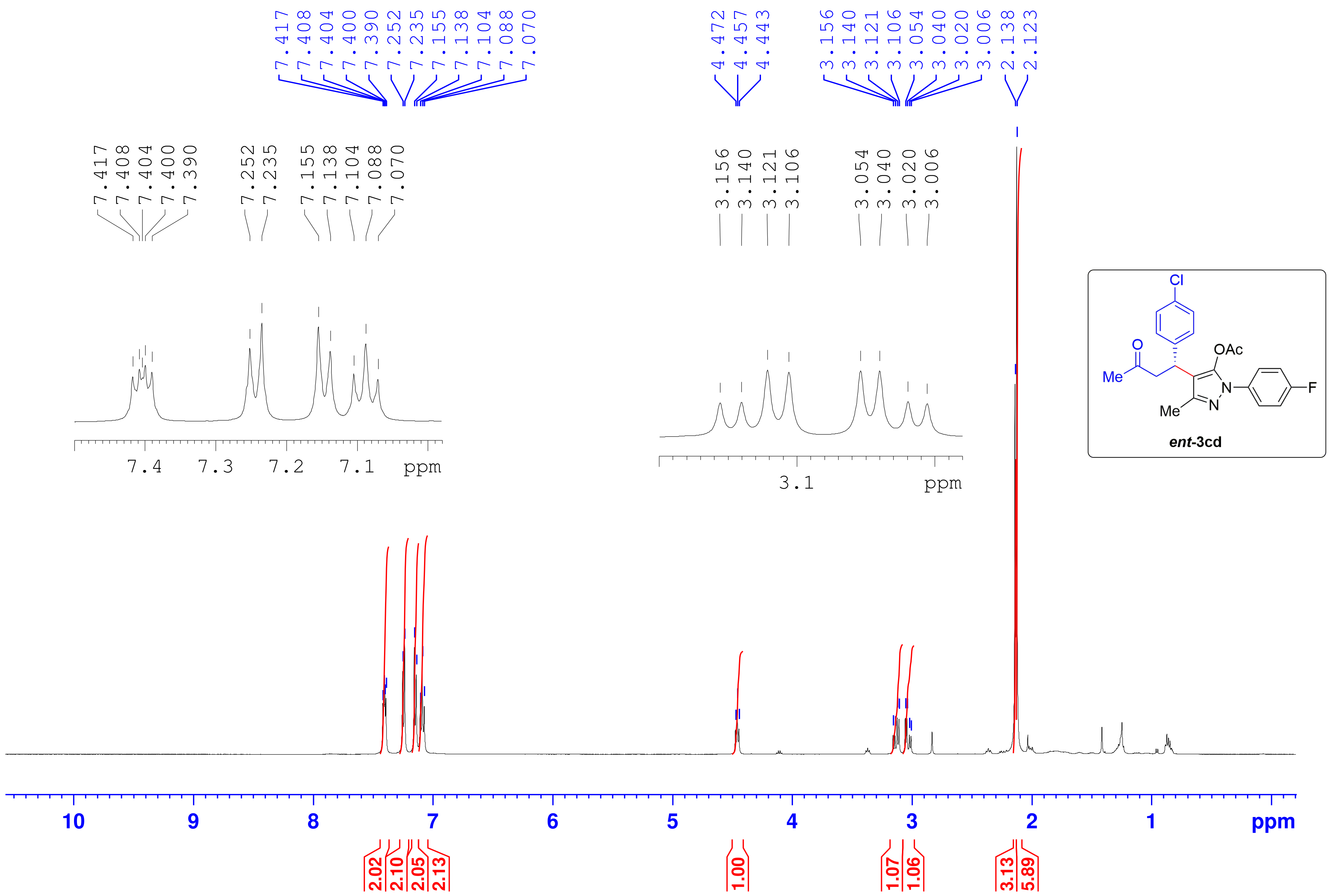
7.417
7.408
7.404
7.400
7.390
7.252
7.235
7.155
7.138
7.104
7.088
7.070
4.472
4.457
4.443
3.156
3.140
3.121
3.106
3.054
3.040
3.020
3.006
2.138
2.123
Cl
O
Me
OAc
N
N
F
Me
ent-3cd
2.02
2.10
2.05
2.13
1.00
1.07
1.06
3.13
5.89
ppm

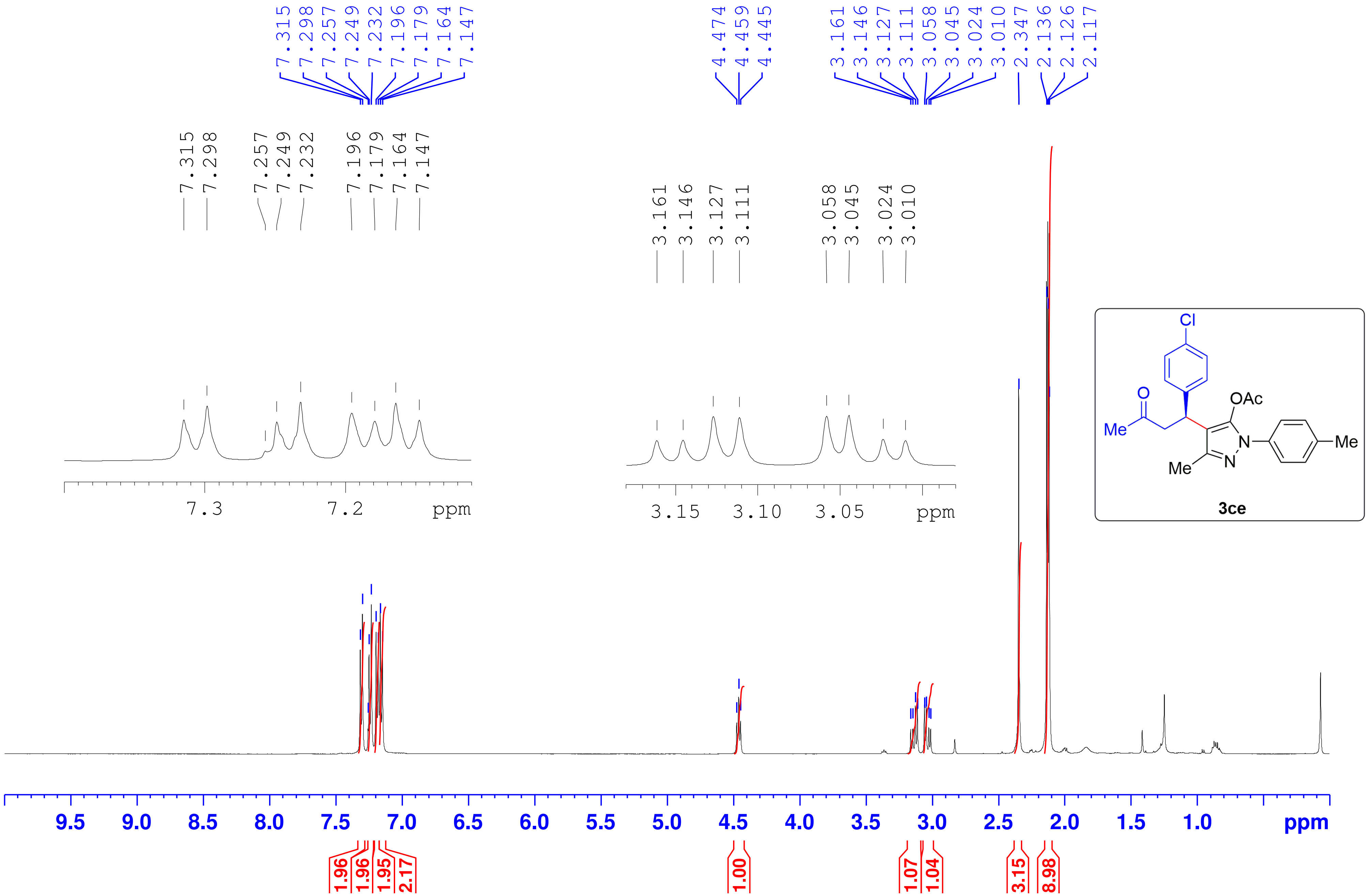

3ce
ppm

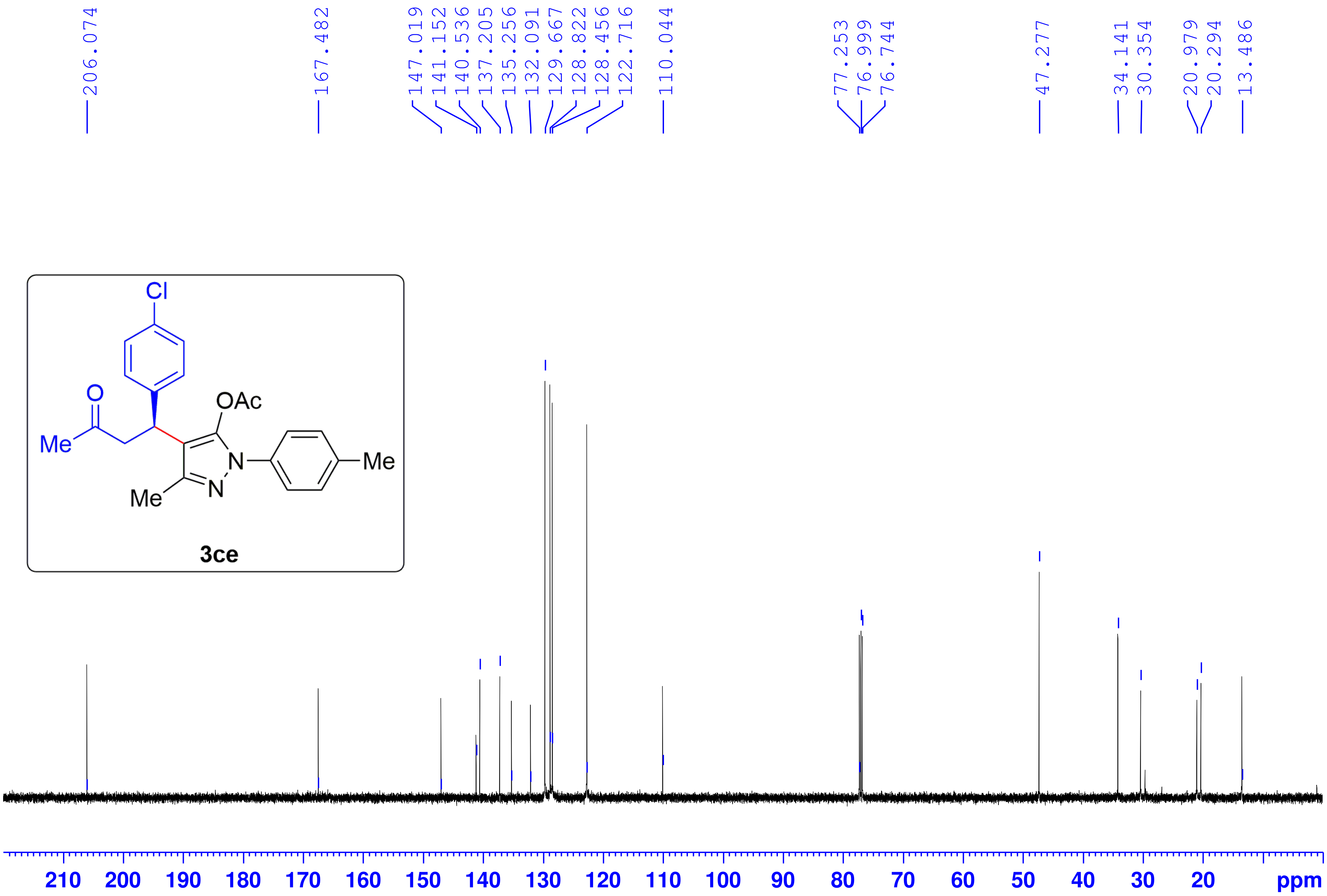
206.074
167.482
147.019
141.152
140.536
137.205
135.256
132.091
129.667
128.822
128.456
122.716
110.044
77.253
76.999
76.744
47.277
34.141
30.354
20.979
20.294
13.486
Cl
O
OAc
Me
N
N
Me
Me
3ce
210
200
190
180
170
160
150
140
130
120
110
100
90
80
70
60
50
40
30
20
ppm

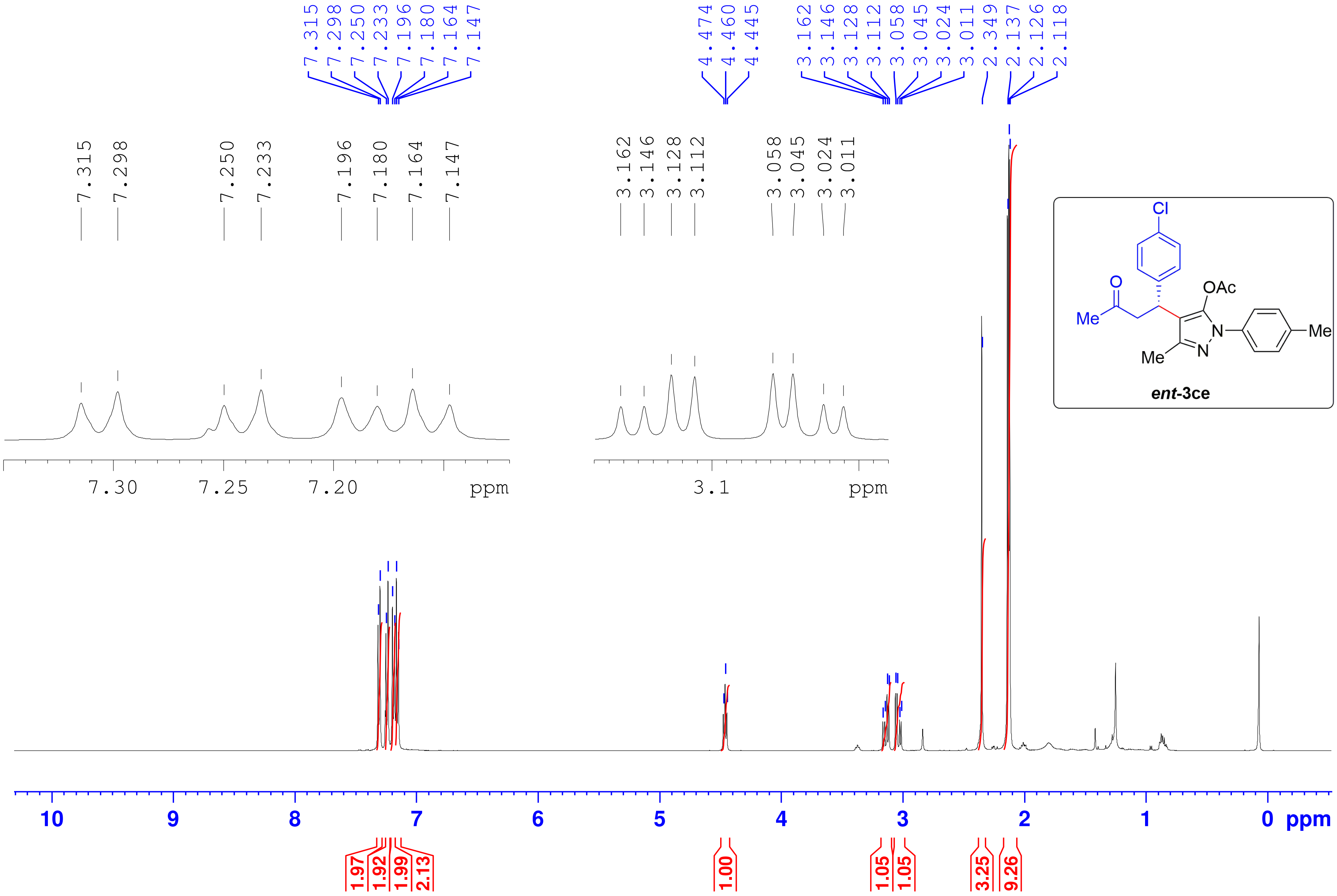
7.315 7.298 7.250 7.233 7.196 7.180 7.164 7.147
4.474 4.460 4.445
3.162 3.146 3.128 3.112 3.058 3.045 3.024 3.011 2.349 2.137 2.126 2.118
7.30 7.25 7.20 ppm
3.1 ppm
10 9 8 7 6 5 4 3 2 1 0 ppm
1.97 1.92 1.99 2.13 1.00 1.05 1.05 3.25 9.26
Cl
O
OAc
Me
N
Me
Me
N
ent-3ce

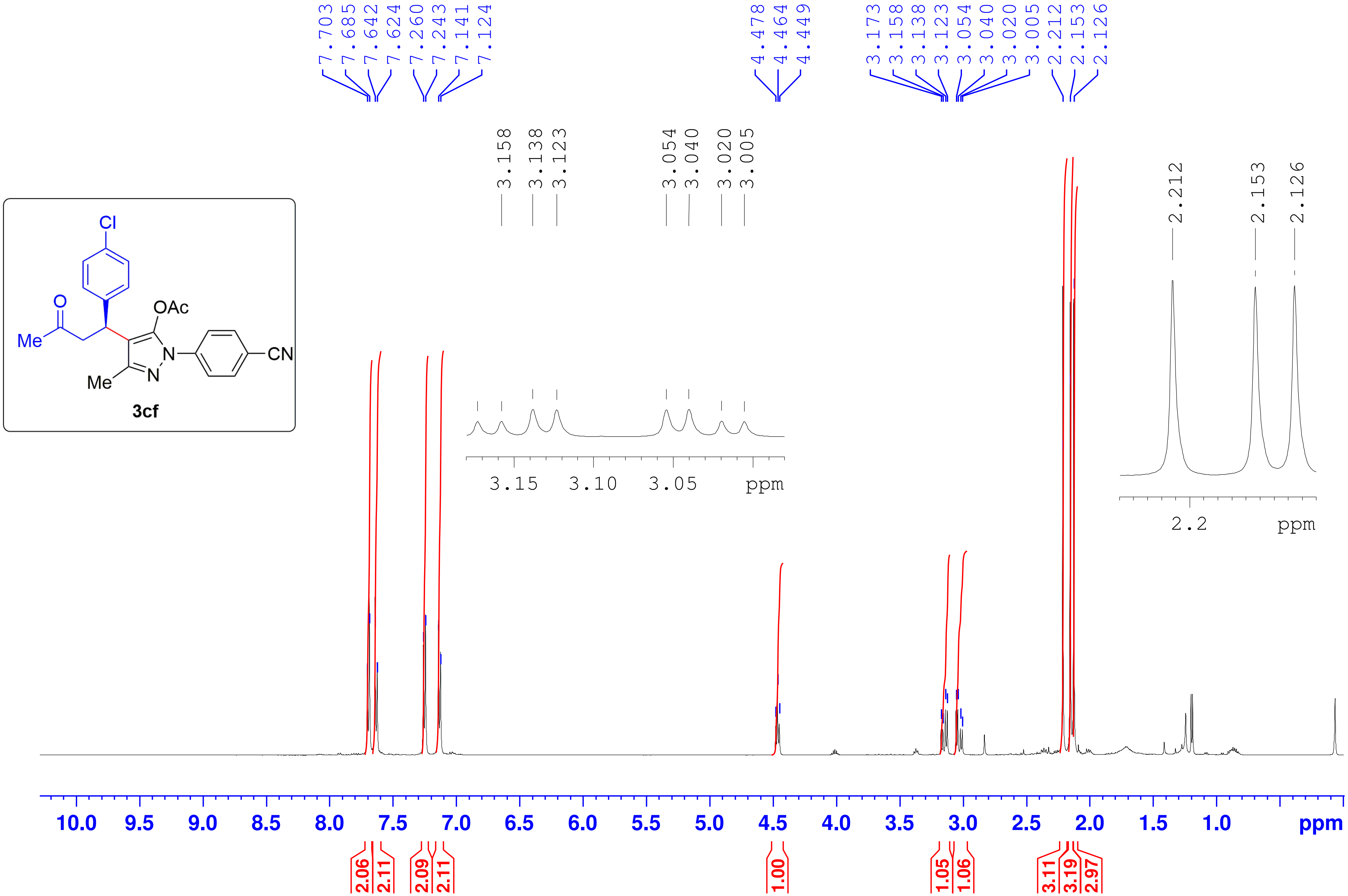
7.703
7.685
7.642
7.624
7.260
7.243
7.141
7.124
4.478
4.464
4.449
3.173
3.158
3.138
3.123
3.054
3.040
3.020
3.005
2.212
2.153
2.126
Cl
O
Me
OAc
N
N
Me
CN
3cf
3.15
3.10
3.05
ppm
2.2
ppm
10.0
9.5
9.0
8.5
8.0
7.5
7.0
6.5
6.0
5.5
5.0
4.5
4.0
3.5
3.0
2.5
2.0
1.5
1.0
ppm
2.06
2.11
2.09
2.11
1.00
1.05
1.06
3.11
3.19
2.97

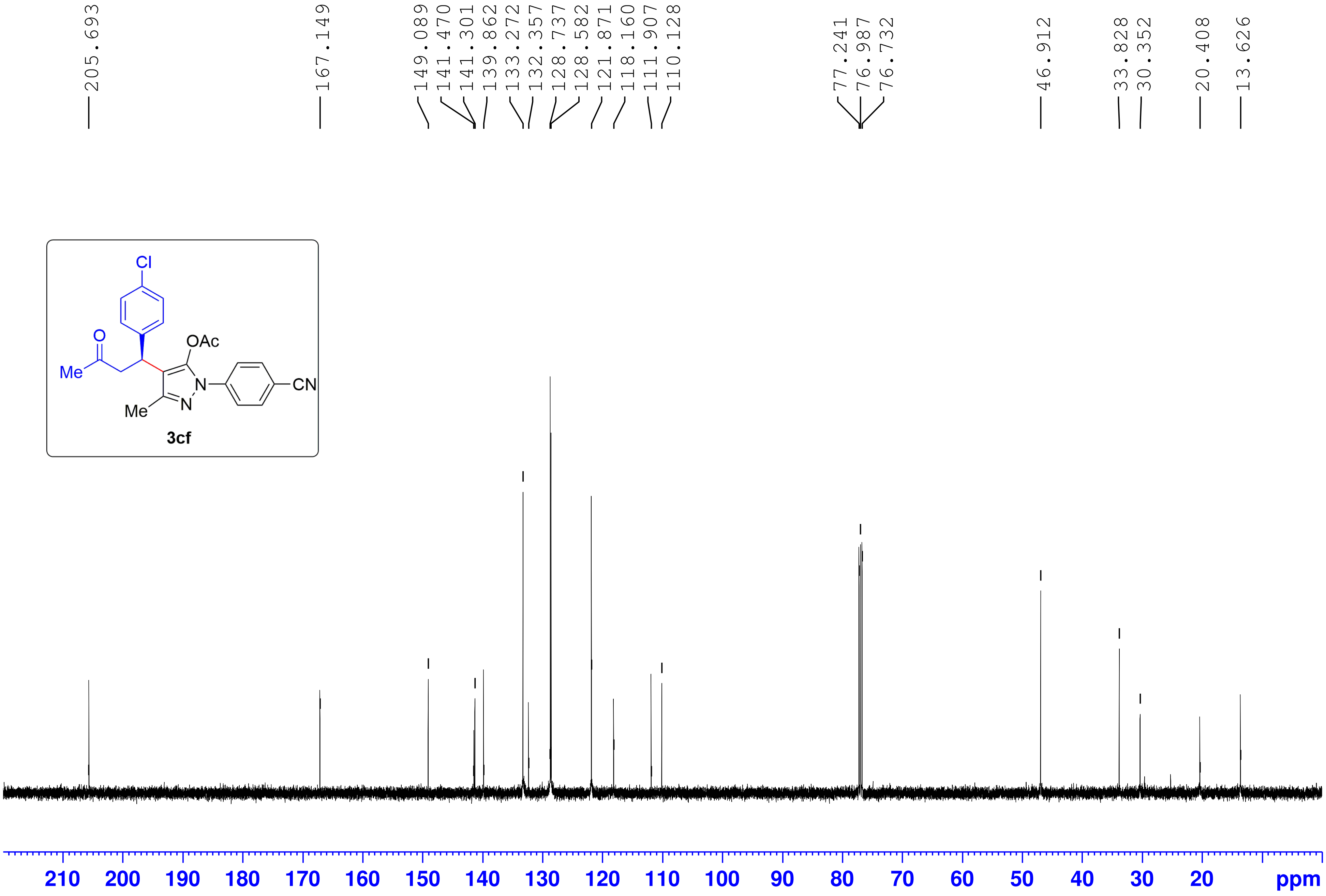
205.693
167.149
149.089
141.470
141.301
139.862
133.272
132.357
128.737
128.582
121.871
118.160
111.907
110.128
77.241
76.987
76.732
46.912
33.828
30.352
20.408
13.626
210 200 190 180 170 160 150 140 130 120 110 100 90 80 70 60 50 40 30 20 ppm
Cl
O
OAc
Me
N
CN
Me
N

3cf

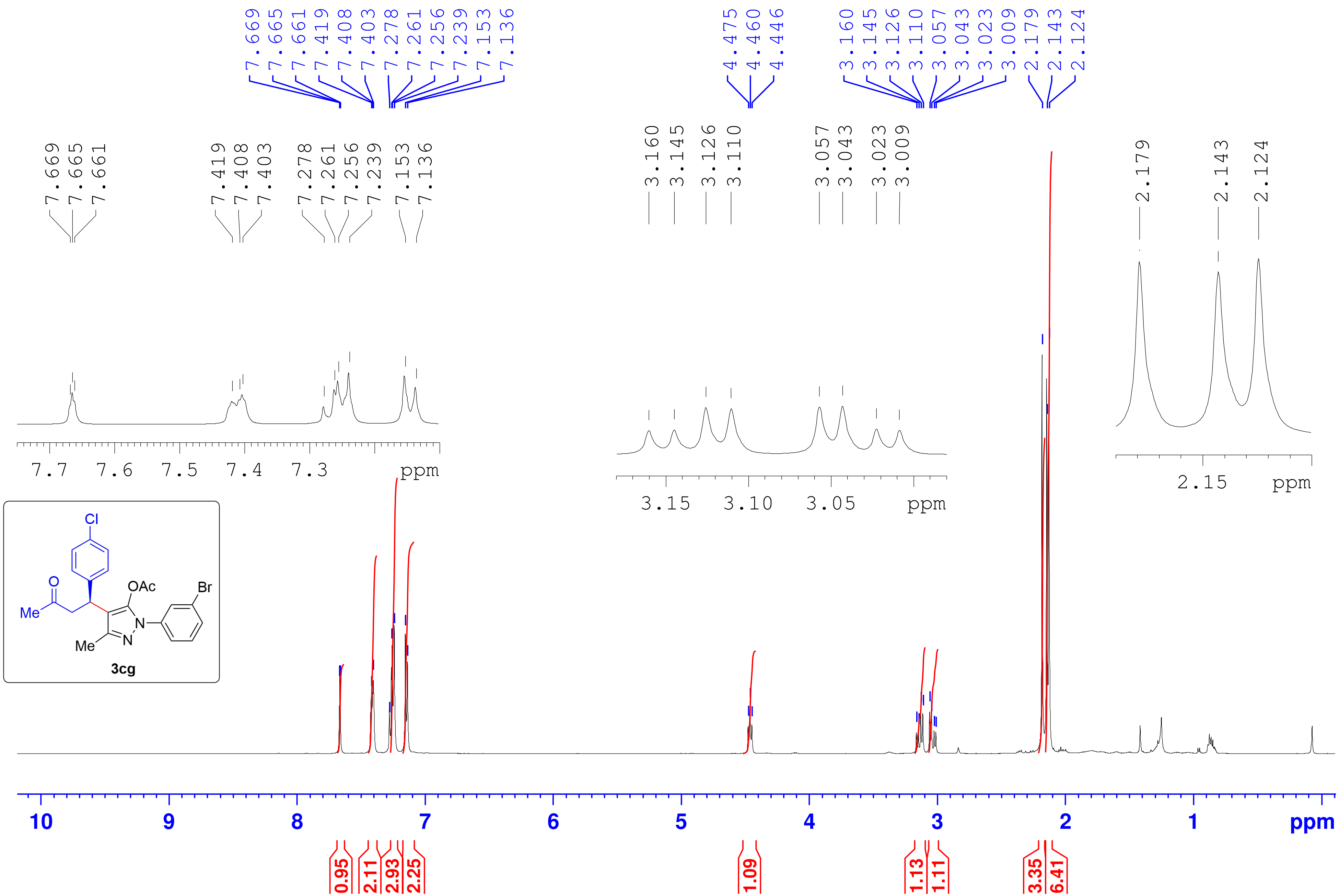
7.669
7.665
7.661
7.419
7.408
7.403
7.278
7.261
7.256
7.239
7.153
7.136
4.475
4.460
4.446
3.160
3.145
3.126
3.110
3.057
3.043
3.023
3.009
2.179
2.143
2.124
Cl
OAc
Br
O
Me
N
N
Me
3cg
ppm
0.95
2.11
2.93
2.25
1.09
1.13
1.11
3.35
6.41

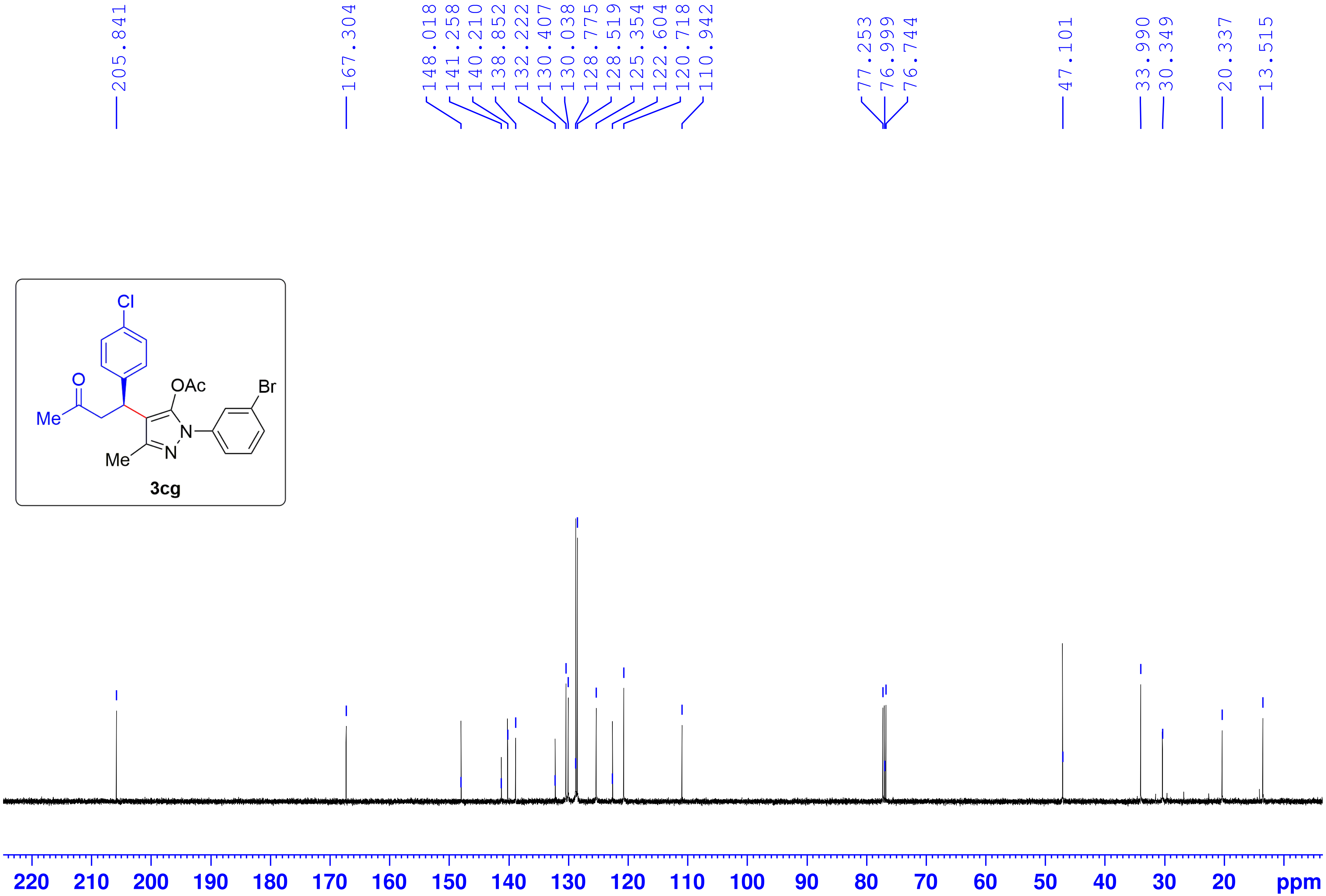
205.841
167.304
148.018
141.258
140.210
138.852
132.222
130.407
130.038
128.775
128.519
125.354
122.604
120.718
110.942
77.253
76.999
76.744
47.101
33.990
30.349
20.337
13.515
Cl
O
OAc
Br
Me
N
N
Me
3cg
220
210
200
190
180
170
160
150
140
130
120
110
100
90
80
70
60
50
40
30
20
ppm

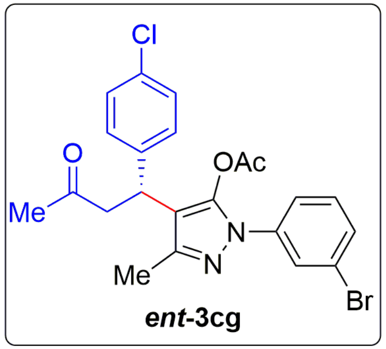
Cl
O
OAc
Me
N
N
Me
Br
ent-3cg

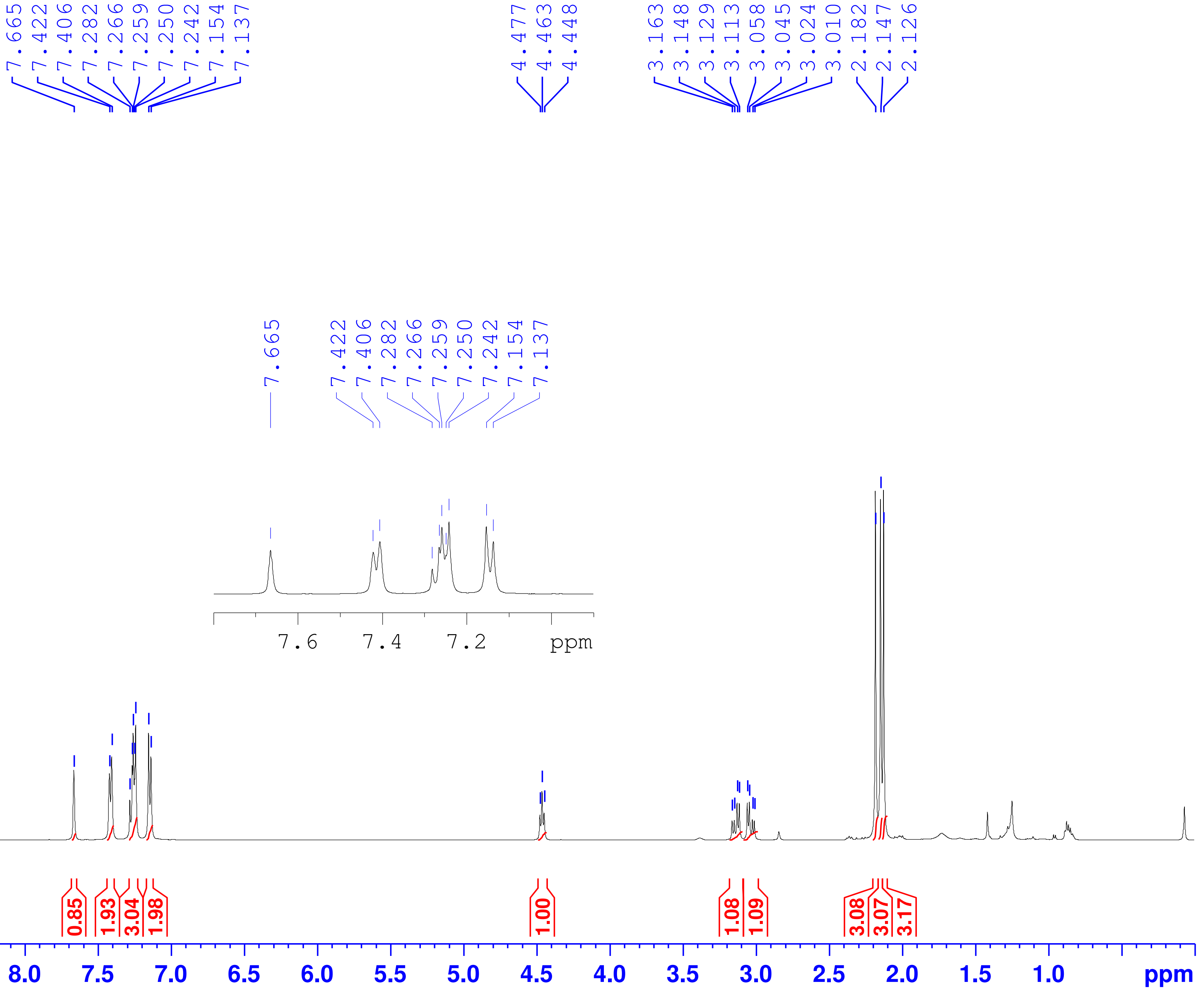
7.665
7.422
7.406
7.282
7.266
7.259
7.250
7.242
7.154
7.137
4.477
4.463
4.448
3.163
3.148
3.129
3.113
3.058
3.045
3.024
3.010
2.182
2.147
2.126
7.6
7.4
7.2
ppm
0.85
1.93
3.04
1.98
1.00
1.08
1.09
3.08
3.07
3.17
9.5
9.0
8.5
8.0
7.5
7.0
6.5
6.0
5.5
5.0
4.5
4.0
3.5
3.0
2.5
2.0
1.5
1.0
ppm

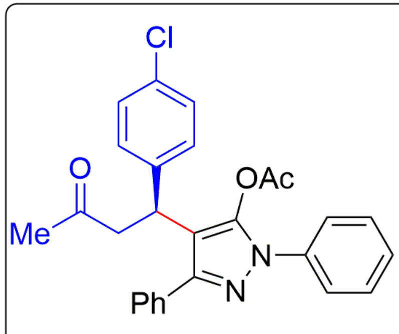
Cl
O
OAc
Me
N
N
Ph

3ch

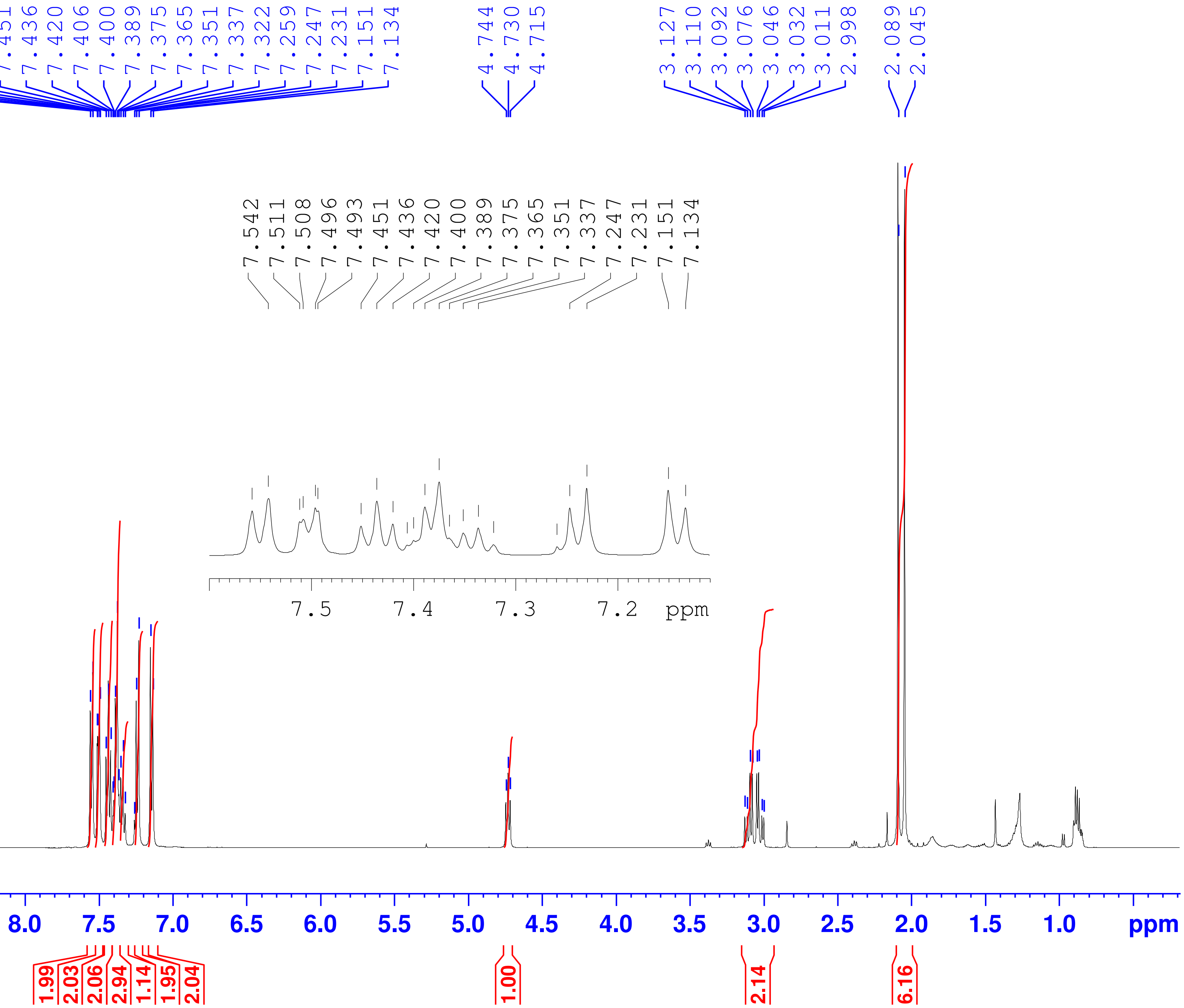
7.558
7.542
7.511
7.508
7.496
7.493
7.451
7.436
7.420
7.406
7.400
7.389
7.375
7.365
7.351
7.337
7.322
7.259
7.247
7.231
7.151
7.134
4.744
4.730
4.715
3.127
3.110
3.092
3.076
3.046
3.032
3.011
2.998
2.089
2.045
7.5
7.4
7.3
7.2
ppm
9.5
9.0
8.5
8.0
7.5
7.0
6.5
6.0
5.5
5.0
4.5
4.0
3.5
3.0
2.5
2.0
1.5
1.0
ppm
1.99
2.03
2.06
2.94
1.14
1.95
2.04
1.00
2.14
6.16

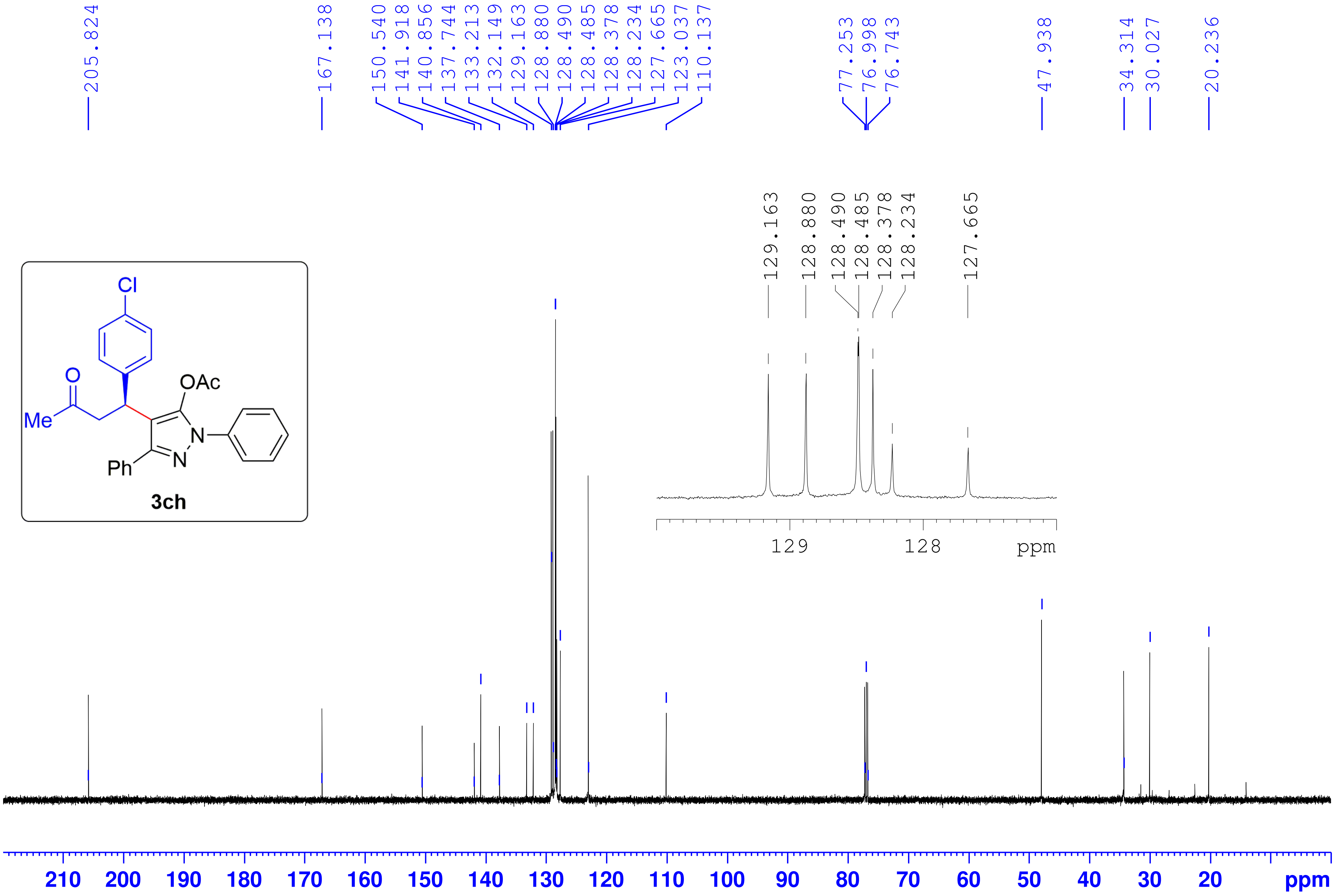
205.824
167.138
150.540
141.918
140.856
137.744
133.213
132.149
129.163
128.880
128.490
128.485
128.378
128.234
127.665
123.037
110.137
77.253
76.998
76.743
47.938
34.314
30.027
20.236
129
128
ppm
Cl
O
OAc
Me
N
N
Ph
210
200
190
180
170
160
150
140
130
120
110
100
90
80
70
60
50
40
30
20
ppm

3ch

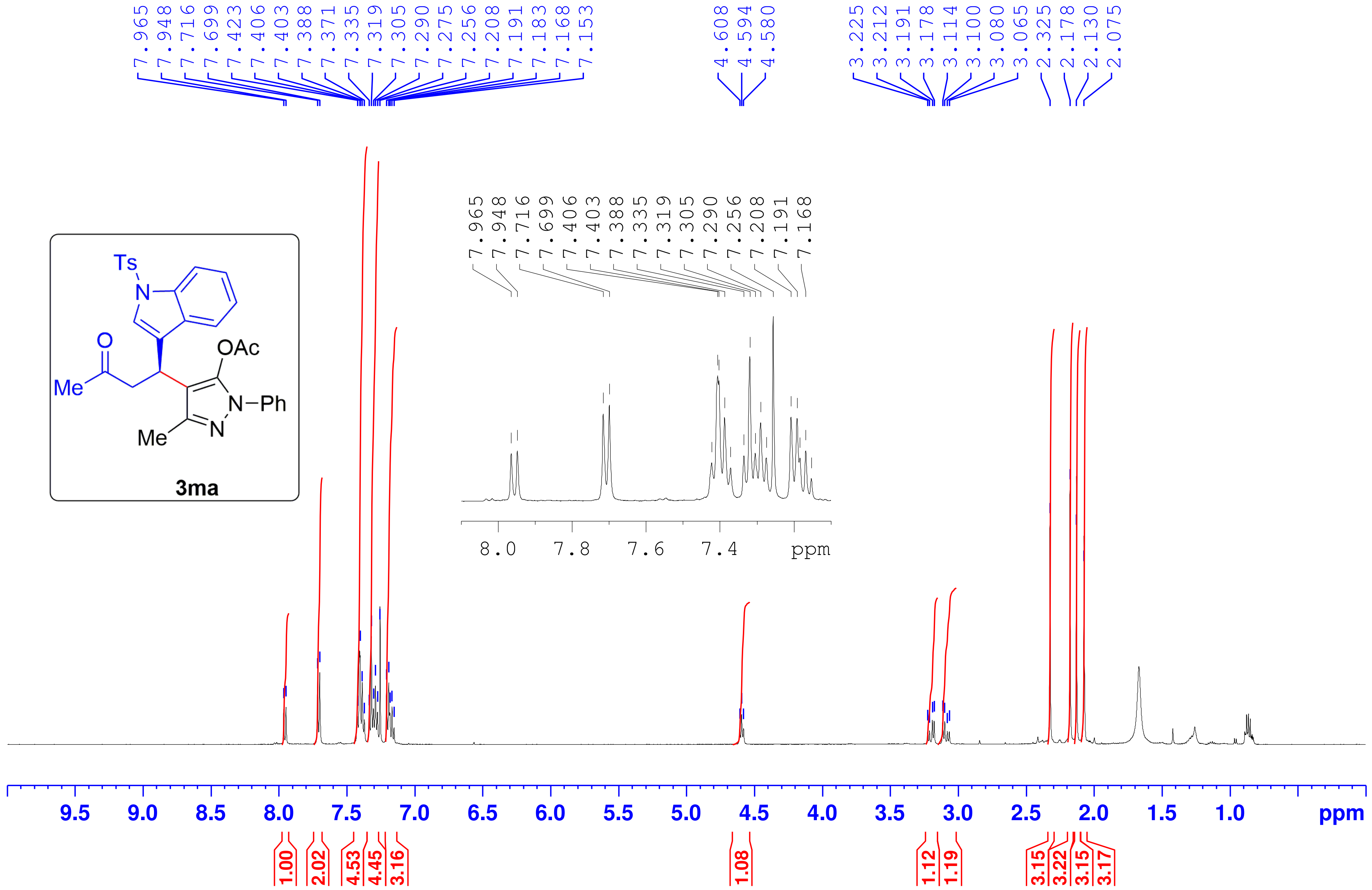

7.965
7.948
7.716
7.699
7.423
7.406
7.403
7.388
7.371
7.335
7.319
7.305
7.290
7.275
7.256
7.208
7.191
7.183
7.168
7.153
4.608
4.594
4.580
3.225
3.212
3.191
3.178
3.114
3.100
3.080
3.065
2.325
2.178
2.130
2.075
Ts
N
O
OAc
Me
N−Ph
Me
N
3ma
8.0
7.8
7.6
7.4
ppm
9.5
9.0
8.5
8.0
7.5
7.0
6.5
6.0
5.5
5.0
4.5
4.0
3.5
3.0
2.5
2.0
1.5
1.0
ppm
1.00
2.02
4.53
4.45
3.16
1.08
1.12
1.19
3.15
3.22
3.15
3.17

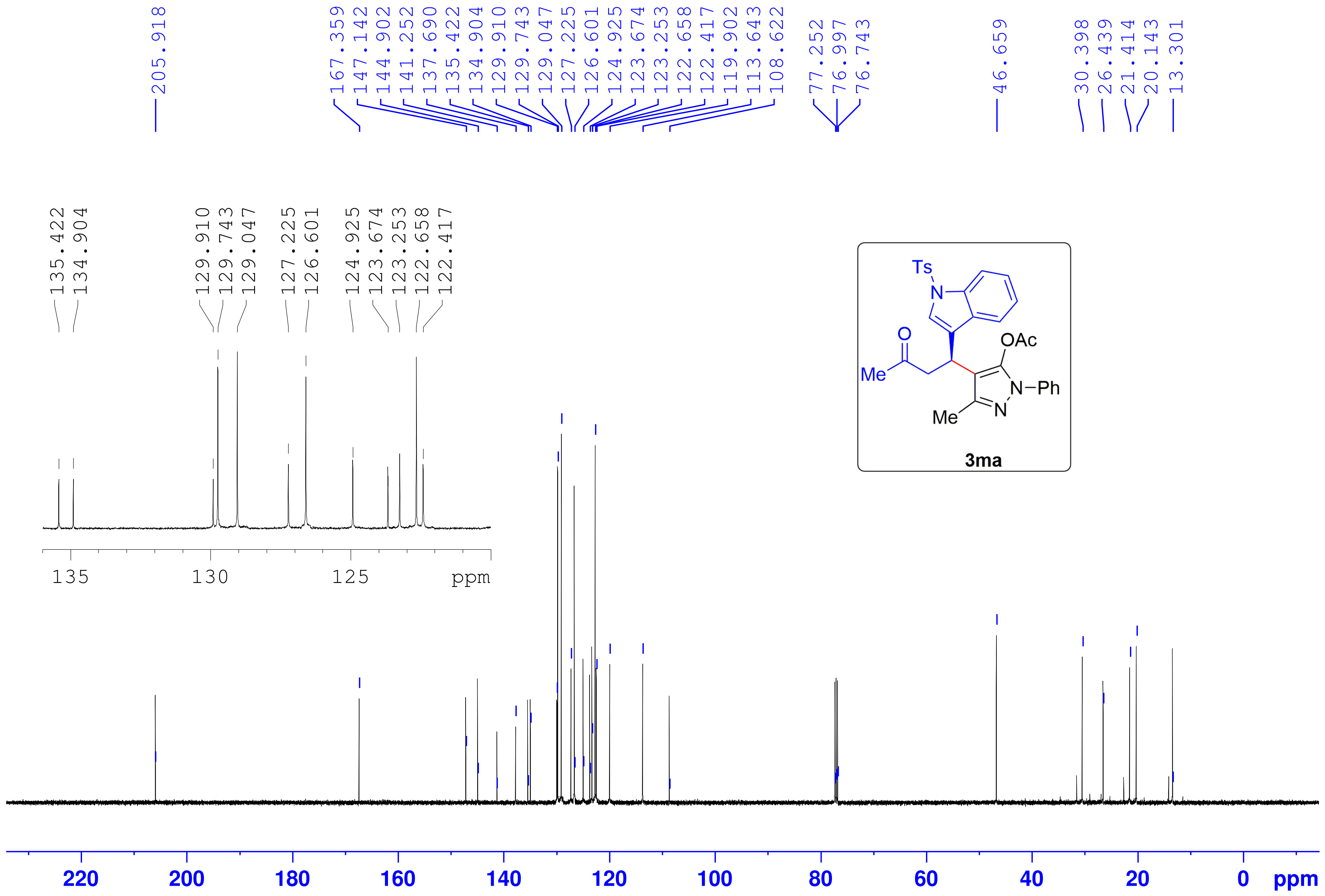

205.918
167.359
147.142
144.902
141.252
137.690
135.422
134.904
129.910
129.743
129.047
127.225
126.601
124.925
123.674
123.253
122.658
122.417
119.902
113.643
108.622
77.252
76.997
76.743
46.659
30.398
26.439
21.414
20.143
13.301
135.422
134.904
129.910
129.743
129.047
127.225
126.601
124.925
123.674
123.253
122.658
122.417
135
130
125
ppm
Ts
N
O
Me
OAc
N–Ph
N
Me
3ma
220
200
180
160
140
120
100
80
60
40
20
0
ppm

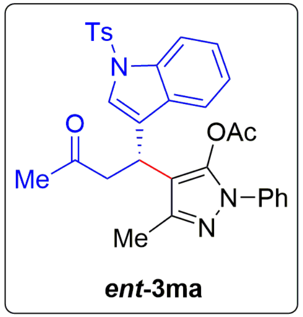
Ts
N
O
OAc
Me
N−Ph
Me
N

ent-**3ma**

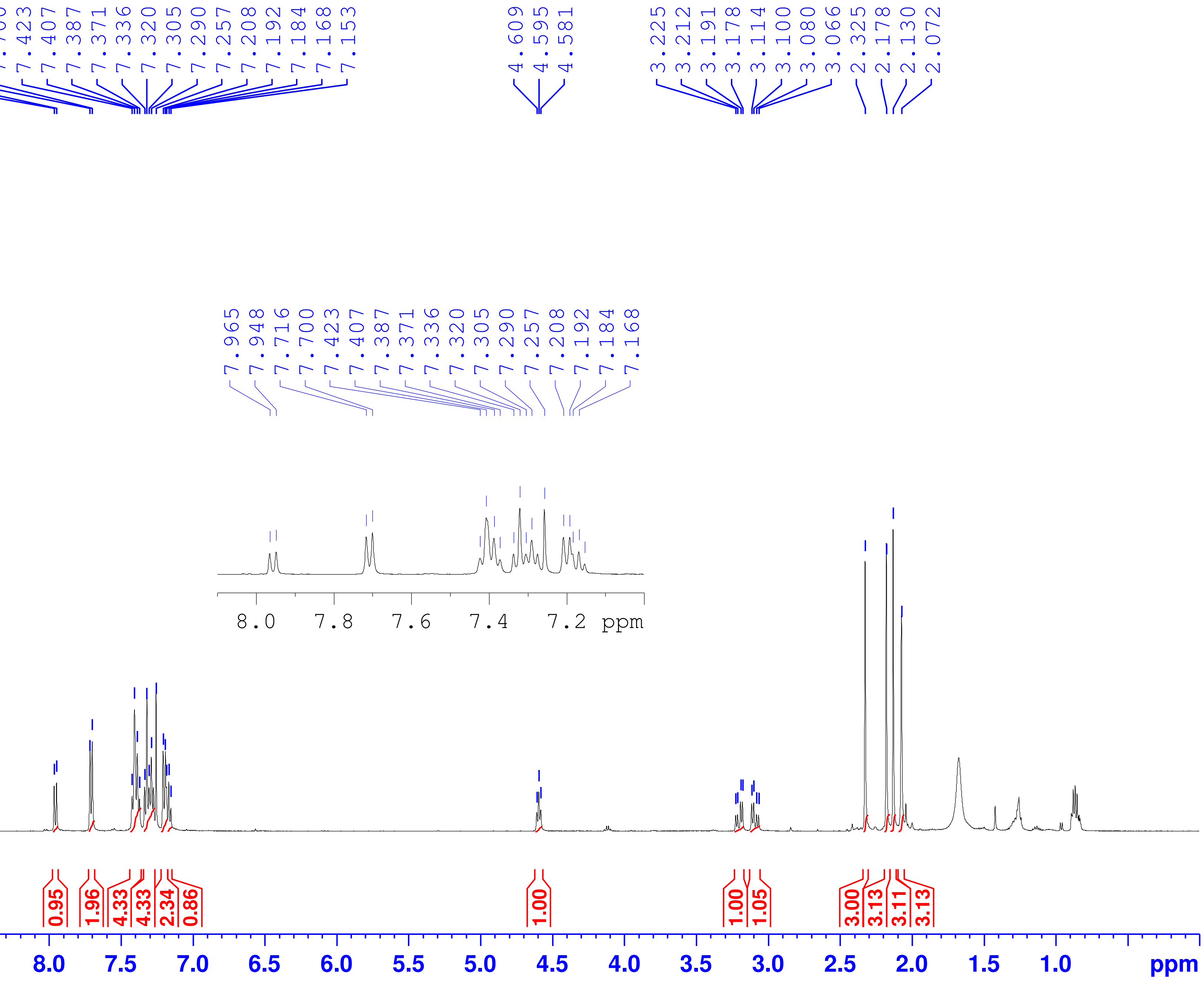
7.965
7.948
7.716
7.700
7.423
7.407
7.387
7.371
7.336
7.320
7.305
7.290
7.257
7.208
7.192
7.184
7.168
7.153
4.609
4.595
4.581
3.225
3.212
3.191
3.178
3.114
3.100
3.080
3.066
2.325
2.178
2.130
2.072
8.0
7.8
7.6
7.4
7.2
ppm
0.95
1.96
4.33
4.33
2.34
0.86
1.00
1.00
1.05
3.00
3.13
3.11
3.13
9.5
9.0
8.5
8.0
7.5
7.0
6.5
6.0
5.5
5.0
4.5
4.0
3.5
3.0
2.5
2.0
1.5
1.0
ppm

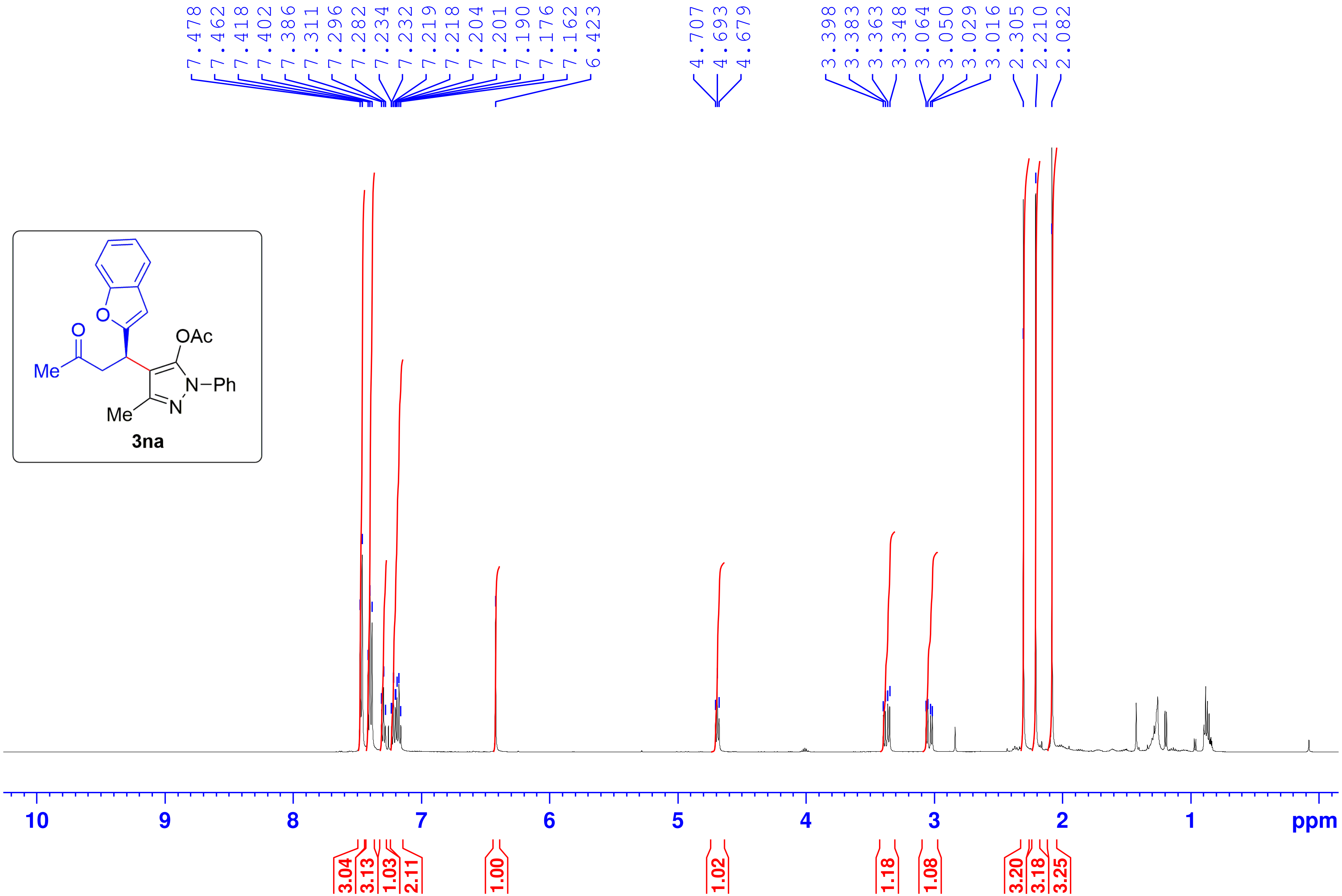
7.478
7.462
7.418
7.402
7.386
7.311
7.296
7.282
7.234
7.232
7.219
7.218
7.204
7.201
7.190
7.176
7.162
6.423
4.707
4.693
4.679
3.398
3.383
3.363
3.348
3.064
3.050
3.029
3.016
2.305
2.210
2.082
OAc
O
O
Me
N−Ph
N
Me
3na
10
9
8
7
6
5
4
3
2
1
ppm
3.04
3.13
1.03
2.11
1.00
1.02
1.18
1.08
3.20
3.18
3.25

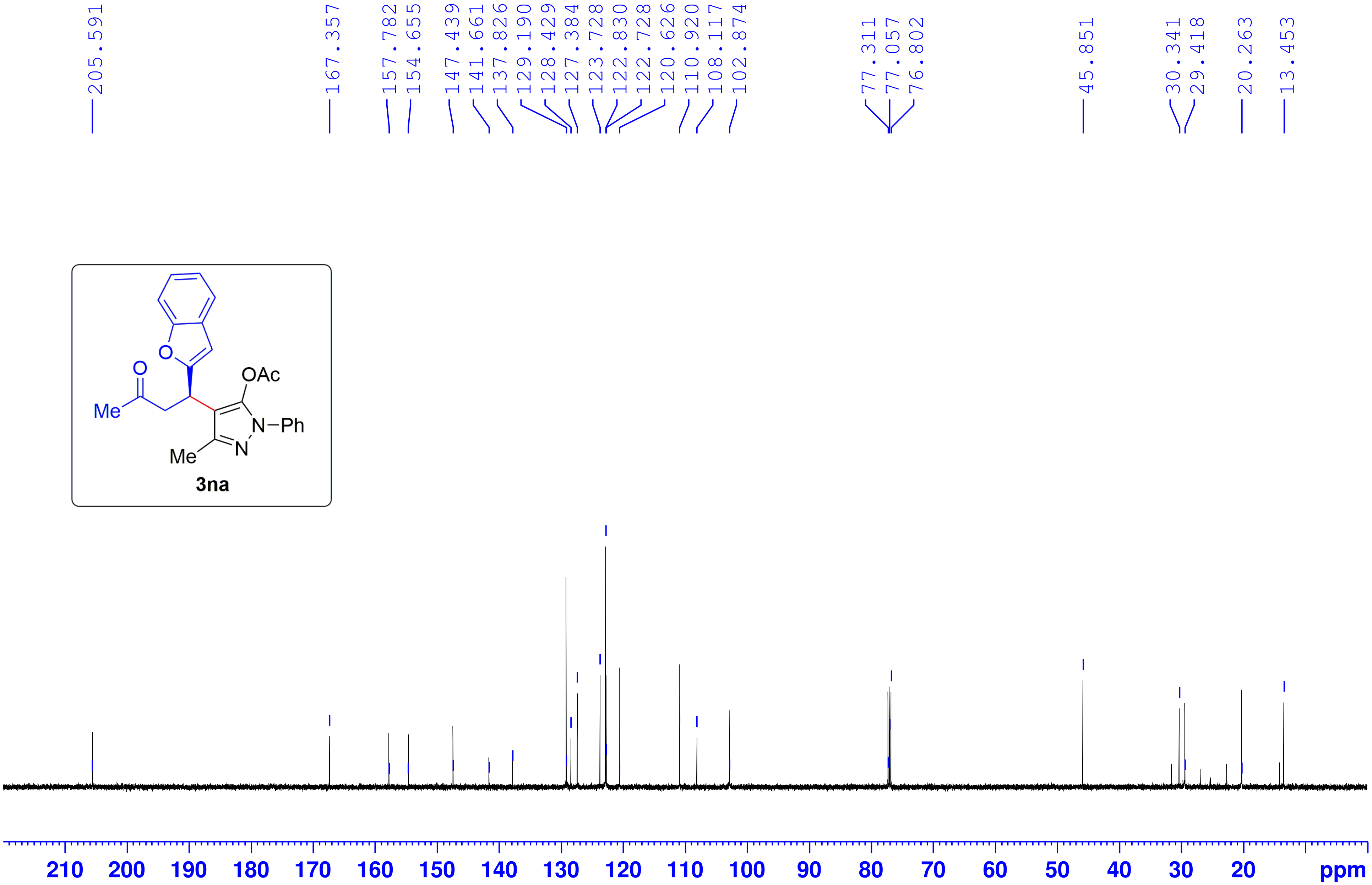
205.591
167.357
157.782
154.655
147.439
141.661
137.826
129.190
128.429
127.384
123.728
122.830
122.728
120.626
110.920
108.117
102.874
77.311
77.057
76.802
45.851
30.341
29.418
20.263
13.453
O
O
OAc
Me
N−Ph
N
Me
3na
210
200
190
180
170
160
150
140
130
120
110
100
90
80
70
60
50
40
30
20
ppm

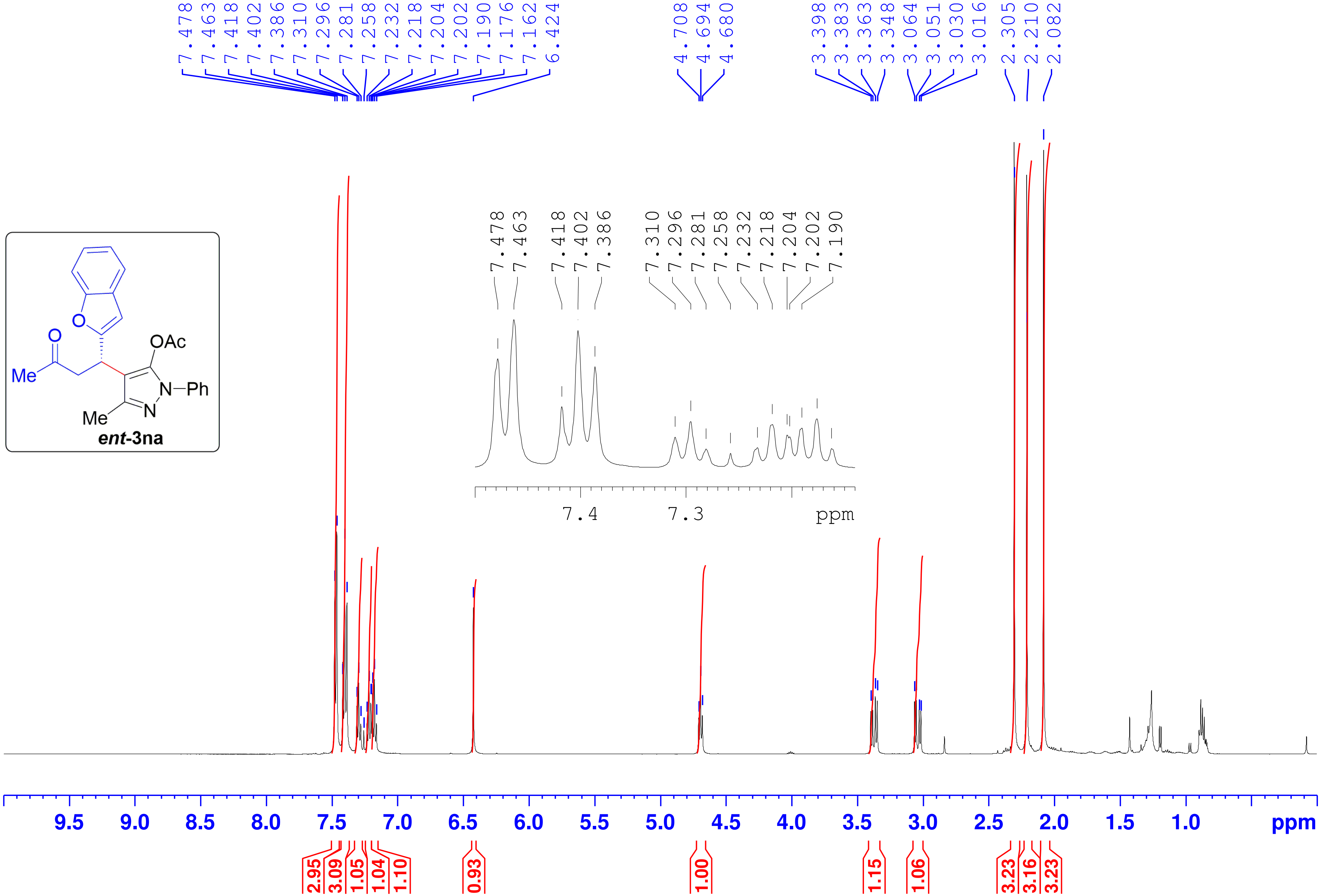
7.478 7.463 7.418 7.402 7.386 7.310 7.296 7.281 7.258 7.232 7.218 7.204 7.202 7.190 7.176 7.162 6.424
4.708 4.694 4.680
3.398 3.383 3.363 3.348 3.064 3.051 3.030 3.016 2.305 2.210 2.082
7.478 7.463 7.418 7.402 7.386 7.310 7.296 7.281 7.258 7.232 7.218 7.204 7.202 7.190
7.4
7.3
ppm
O
OAc
Me
N–Ph
N
O
Me
ent-3na
9.5 9.0 8.5 8.0 7.5 7.0 6.5 6.0 5.5 5.0 4.5 4.0 3.5 3.0 2.5 2.0 1.5 1.0
ppm
2.95 3.09 1.05 1.04 1.10 0.93 1.00 1.15 1.06 3.23 3.16 3.23
